# Comparative genomics and phylogenomics of the *Ralstonia solanacearum* Moko ecotype and its symptomatological variants

**DOI:** 10.1590/1678-4685-GMB-2022-0038

**Published:** 2022-12-02

**Authors:** Ana Karolina Leite Pais, Leandro Victor Silva dos Santos, Greecy Mirian Rodrigues Albuquerque, Antonio Roberto Gomes de Farias, Wilson José Silva, Valdir de Queiroz Balbino, Adriano Márcio Freire Silva, Marco Aurelio Siqueira da Gama, Elineide Barbosa de Souza

**Affiliations:** 1 Universidade Federal Rural de Pernambuco (UFRPE), Departamento de Agronomia, Recife, PE, Brazil.; 2 Universidade Federal de Pernambuco (UFPE), Departamento de Genética, Recife, PE, Brazil.; 3 Universidade Federal de Alagoas (UFAL), Departamento de Agronomia, Maceió, AL, Brazil.; 4 Universidade Federal Rural de Pernambuco (UFRPE), Departamento de Biologia, Recife, PE, Brazil.

**Keywords:** Genomics, *Musa* spp, Sergipe facies, Bugtok, symptomatology

## Abstract

Banana tree bacterial wilt is caused by the *Ralstonia solanacearum* Moko ecotype. These strains vary in their symptom progression in banana, and are classified as typical Moko variants (phylotype IIA and IIB strains from across Central and South America), Bugtok variant (Philippines), and Sergipe facies (the states of Sergipe and Alagoas, Brazil). This study used comparative genomic and phylogenomic approaches to identify a correlation between the symptom progression of the Moko ecotypes based on the analysis of 23 available genomes. Average nucleotide identity and *in silico* DNA-DNA hybridization revealed a high correlation (>96% and >78%, respectively) between the genomes of Moko variants. Pan-genome analysis identified 21.3% of inheritable regions between representatives of the typical Moko and Sergipe facies variants, which could be traced to an abundance of exclusive homolog clusters. Moko ecotype genomes shared 1,951 orthologous genes, but representatives with typical symptoms did not display unique orthologues. Moreover, Bugtok disease and Sergipe facies genomes did not share any unique genes, suggesting convergent evolution to a shared symptom progression. Overall, genomic and phylogenomic analyses were insufficient to differentiate the Moko variants based on symptom progression.

## Introduction

The *Ralstonia solanacearum* Moko ecotype was first described by Schomburgk during his travels to British Guiana in 1840-1844, and gained economic importance after it devastated banana plantations of the ‘Moko’ cultivar (*Musa* ABB, Bluggoe subgroup) on the island of Trinidad in the early Twentieth century (Sequeira, 1998). ‘Ecotype’ describes a group highly adapted to its host, and the Moko ecotype is a polyphyletic group intimately associated with the *Musa* spp. (Fegan and Prior, 2006; Ailloud et al., 2015).

The pathological outcome caused by the Moko ecotype depends on which of the three variants is involved: Moko, Bugtok, or Sergipe facies (Albuquerque et al., 2014; Blomme et al., 2017). The typical symptoms of Moko, which is the most widespread variant, begin in the rhizomes and then move towards the pseudostem, vascular darkening is observed in the central region, the leaves turn yellow and wilt, the fruits become deformed (Albuquerque *et al*., 2014), and finally the whole plant wilts (Sequeira, 1998). In the Philippines, the sequevar IIB-3 of *R. solanacearum* attacks the cultivars ‘Saba’ (*Musa* BBB) and ‘Cardaba’ (*Musa* ABB), causing a disease called Bugtok or Tapurok (Roperos, 1965; Soguilon et al., 1995), with infection starting in the inflorescence due to inoculation by vector insects (Blomme *et al*., 2017). Bugtok symptoms are restricted to the stalk, rachis, and fruits, together with an occasional reddish-brown discoloration of the vascular tissue of the pseudostem, which rarely extends to the rhizome (Blomme *et al*., 2017). Sergipe facies occurs only in northeastern Brazil, in the states of Sergipe and Alagoas, where it is associated with sequevar IIA-53 of *R. solanacearum*. The symptomatological picture is similar to that of Bugtok, but the fruits are uneven, ripen prematurely (Albuquerque *et al*., 2014), and display external necrosis (Albuquerque *et al*., 2021a,b), although both are a result of inoculation by insects vectors. 

In spite of Moko ecotype identification (Albuquerque et al., 2014, 2021a) and genomic characterization of representative strains by Silva et al. (2020) and Pais et al. (2021), no genomic studies have looked the different symptomatology caused by the *R. solanacearum* Moko ecotype. Therefore, the objective of this study was to investigate the representatives of the Moko ecotype that induce different symptoms in banana trees using comparative genomics and phylogenomic approaches.

## Material and Methods

### Genomic sequences

The genomes *of R. solanacearum* available from the National Center for Biotechnology Information (NCBI) at January 2021 were filtered to obtain a final dataset containing only representatives of the three symptomatological variants of the Moko ecotype. These included the ‘M’ group causing typical Moko symptoms (19 genomes of sequevars IIA-6, IIA-24, IIB-3, IIB-4, and IIB-25), the ‘B’ group causing Bugtok symptoms (two genomes of sequevar IIB-3), and the ‘SE’ group causing Sergipe facies (two genomes of sequevar IIA-53). The origin and characteristics of the genomes used in this study are listed in Table 1. Those defined as Bugtok genomes are all representatives of the phylotype/sequevar IIB-3 that originated in the Philippines.

**Table 1-:** Characteristics of *Ralstonia solanacearum* Moko ecotype strains and its symptomatological variants used in this study.

Strain^a^	Sequevar	Origen	Level^b^	N50	Size (pb)	Access
B50^M^	IIA-24	Peru	Scaffold	5596066	5.596.07	GCF_000825785.1
CFBP1416^M^	IIB-3	Costa Rica	Scaffold	5744274	5.744.27	GCF_000825925.1
CCRMRs277^M^	IIA-24	Brazil	Chromosome	3549795	5.636.326	GCA_014210395.1
CCRMRs287 ^M^	IIB-4	Brazil	Chromosome	3512030	5.444.697	GCA_014210375.1
CCRMRs304 ^M^	IIA-24	Brazil	Chromosome	3549663	5.645.239	GCA_014210335.1
CCRMRsB7 ^M^	IIB-25	Brazil	Chromosome	3716474	5.854.658	GCA_014210345.1
Grenada 9-1 ^M^	IIA-6	Grenada	Scaffold	5479463	5.479.46	GCF_000825845.1
IBSBF1900 ^M^	IIA-24	Brazil	Scaffold	5812604	5.812.6	GCF_001373275.1
Po82 ^M^	IIB-4	Mexico	Complete	3481091	5.430.26	GCF_000215325.1
UW163 ^M^	IIB-4	Peru	Complete	3509932	5.596.24	GCF_001587135.1
UW179 ^M^	IIB-4	Colombia	Scaffold	5426414	5.426.41	GCF_000825805.1
UW181 ^M^	IIA-6	Venezuela	Scaffold	5436691	5.436.69	GCF_001373315.1
UA-1609 ^M^	IIB-4	Colombia	Scaffold	5068052	5.068.05	GCF_003860765.1
UA-1617 ^M^	IIB-4	Colombia	Scaffold	5362479	5.362.48	GCF_003860745.1
UA-1579 ^M^	IIB-4	Colombia	Scaffold	5081426	5.081.43	GCF_003860725.1
UA-1591 ^M^	IIB-4	Colombia	Scaffold	5351985	5.351.98	GCF_003860705.1
UA-1611 ^M^	IIA-6	Colombia	Scaffold	5195693	5.195.69	GCF_003860685.1
UA-1612 ^M^	IIA-6	Colombia	Scaffold	5003359	5.003.36	GCF_003860665.1
10314 ^M^	II	Philippines	Contig	36872	5.458.21	GCF_008271875.1
CIP417^B^	IIB-3	Philippines	Scaffold	5523709	5.523.71	GCF_000825825.1
MOLK2^B^	IIB-3	Philippines	Contig	30467	5.551.88	GCF_000212635.3
SFC^SE^	IIA-53	Brazil	Chromosome	3656700	5.7134.71	GCF_003590625.1
IBSBF2570^SE^	IIA-53	Brazil	Chromosome	3630670	5.722.671	GCF_003590585.1

a M, typical symptoms of Moko; B, Bugtok symptoms; SE, symptoms of Sergipe facies.

b Chromosome, scaffold, and contig are incomple genomes.

### *Comparative genomics of the* R. solanacearum *Moko ecotype*

The average nucleotide identity (ANI) and tetranucleotide signature (TETRA) correlation indices were calculated using the pyani 0.2.7 Python3 module (Pritchard et al., 2016). The ANI was calculated by global alignment of the MUMmer algorithm (ANIm; Kurtz et al., 2004). *In silico* DNA-DNA hybridization (*is*DDH) values were calculated using the Genome-to-Genome Distance Calculator platform 2.1 (Meier-Kolthoff et al., 2013) by applying formula 2 for incomplete genomes. Accordingly, *is*DDH estimates were based on identities/high-scoring pair length. The similarity matrices obtained by ANIm and *is*DDH were converted into a heatmap using the Morpheus platform.

### *Pan-genome and phylogenetic analysis of the* R. solanacearum *Moko ecotype*

Pan-genome analysis of strains was performed in Roary v. 3.13.0 (Page et al., 2015) using genome sequences obtained from RefSeq/NCBI. The resulting orthologous genes were classified as core (genes common to all genomes), softcore (genes contained in 95% of genomes), shell (moderately conserved genes present in various genomes), and clouds (rare genes present in only a few genomes) according to the default settings of the software. The functions and descriptions of gene clusters were acquired from the UniProt platform (The UniProt Consortium, 2021). The core gene pool was automatically aligned using MAFFT v. 7.3102 (Katoh and Standley, 2013) and implemented in Roary using the *-mafft* flag. This created a multi-FASTA nucleotide sequence alignment of all core genes*.* The phylogenomic tree of the core genes was constructed using multi-FASTA alignment with the maximum likelihood method in IQ-TREE v. 2.0.4 (Nguyen et al., 2015). ModelFinder (Kalyaanamoorthy et al., 2017) was employed to select the best evolutionary model. Node support was determined by ultrafast bootstrap (Minh et al., 2013) with 100,000 repetitions. The maximum likelihood tree was viewed in Figtree v.1.4.4 (Rambaut, 2009). IQ-TREE v. 2.0.4 was used to construct the phylogeny matrix summarizing the presence/absence of a gene with the same bootstrap configuration and following the same process described earlier for visualization.

## Results

### Genomic sequences

The analyses were performed with the 23 genomes available in this group, however some limitations were found regarding the level of assembly and representation of Bugtok disease and Sergipe facies, since both contained only two representatives (Table 1).

### *Comparative genomics of the* R. solanacearum *Moko ecotype*

ANIm analysis revealed an average similarity of 97.6% among the 23 genomes of the *R. solanacearum* Moko ecotype (Table 2). A strong similarity was observed in the Moko genomes of the three symptomatic variants (Figure S1). Specifically, ‘M’ genomes presented 97.3% similarity with ‘B’ and 97.4% with ‘SE’ genomes; whereas ‘B’ genomes presented 96.3% similarity with their ‘SE’ counterparts. TETRA values confirmed the elevated similarity (99.9%-100%) between the genomes (Table 2).

**Table 2 -:** Estimates of ANIm, TETRA, and *is*DDH for *Ralstonia solanacearum* Moko ecotype strains and its symptomatological variants.

Species	ANIm		Tetra		***is*DDH**	
	Average value	Variation	Average value	Variation	Average value	Variation
Among *R. solanacearum* ecotype Moko strains	97.6	96.1 - 100	99.9	99.9 - 100	78.8	66.1 - 100
Among *R. solanacearum* phylotype IIA strains	99.4	98.9 - 100	99.9	99.9 - 100	94	88.9 - 99.9
Among *R. solanacearum* phylotype IIB strains	98.7	97.4 - 100	99.9	99.9 - 100	87.8	76.6 - 100
Phylotype IIA - phylotype IIB	96.2	96.1 - 96.4	99.9	99.7 - 99.9	67.1	66.1 - 68.6
Among *R. solanacearum* Moko strains^a^	97.7	96.1 - 100	99.9	99.7 - 100	73.2	66.1 - 100
Among *R. solanacearum* Bugtok strains^a^	100	99.9 - 100	99.9	99.9 - 100	99.8	99.5 - 99.7
Among *R. solanacearum* Sergipe facies strains^a^	100	^b^	100	^b^	99.9	99.9 - 100
Moko - Bugtok^a^	97.3	96.2 - 100	99.9	99.7 - 99.9	77.2	66.8 - 66.8
Moko - Sergipe facies^a^	97.4	96.1 - 99.1	99.9	99.8 - 99.9	67.6	66.1 - 91.9
Bugtok - Sergipe facies	96.3	^b^	99.9	^b^	67.7	67.4 - 68.1

a Moko, typical symptoms of Moko; Bugtok, symptoms recorded in the Philippines; Sergipe facies, symptoms documented in Brazil.

b No variation.

Based on *is*DDH results, the genomes of the Moko ecotype presented an average similarity of 78.8%, with a variation of 66.1%-100% (Table 2). The mean values for phylotypes IIA and IIB were 94% and 87.8%, respectively, with a similarity of 67.1% among them. Group ‘M’ presented 73.2% similarity, whereas groups ‘B’ and ‘SE’ exhibited more than 99% similarity. Intergroup comparison revealed 77.2% and 67.3% similarity between ‘M’ and ‘B’ or ‘SE’ groups, respectively, as well as 67.7% similarity between ‘B’ and ‘SE’ groups (Table 2).

A heatmap was constructed with the means calculated by the ANIm and *is*DDH of the Moko ecotype (Figure S1). Accordingly, the strains were grouped into two large phylotypes (IIA and IIB), which were further divided into four subgroups: IIA(α), IIA(β), IIB(α), and IIB(β). ‘M’ genomes were present mainly in subgroups IIA(α) and IIB(β), although some genomes of this group were detected also in other subgroups. ‘B’ genomes displayed significant similarity and constituted a group together with some ‘M’ genomes. Similar results were observed for ‘SE’ genomes.

### *Pan-genome and multilocus sequence analysis of the* R. solanacearum *Moko ecotype*

**Figure 1-:**
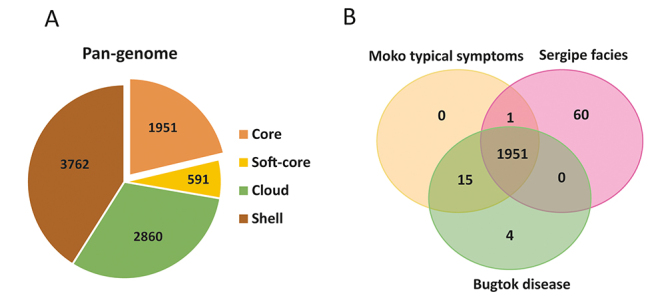
Pan-genome representation of the *Ralstonia solanacearum* Moko ecotype generated by Roary software. (A) Gene categories (core, softcore, shell, and cloud) present in genomes were identified with 90% percent identity. (B) Venn diagram showing clusters present in the genome of *R. solanacearum* Moko ecotype strains and its symptomatological variants. Strains of Moko ecotype causing Bugtok symptoms (CIP417 and Molk2), Sergipe facies (IBSBF2570 and SFC), and typical symptoms of Moko (all other strains) are shown.

Pan-genome analyses revealed the presence of 9,164 clusters of information*,* of which 1,951 were identified as core genes (Figure 1A and Table S1). A total of 3,308 clusters were found among representatives with typical Moko symptoms, but none were common to all representatives (Figure 1B and Table S2). ‘B’ genomes contained 135 clusters, of which two insertions and two unknown sequences were detected in this group. (Tables S2 and S3). The identified gene clusters included the transposase families IS3 and IS5, as well as two proteins of unknown function (one containing the domain DUF4158 plus a hypothetical protein). ‘SE’ genomes revealed 113 clusters, of which 60 were present in all representatives of this group and were related to biological processes, molecular functions, relationship with binding molecules, the type three secretion system (T3SS), insertion sequences, and CRISPR (Tables S2 and S3).

‘M’ and ‘B’ genomes shared 15 unique clusters, while ‘M’ and ‘SE’ genomes shared only one cluster (Figure 1B and Tables S2 and S3). The information shared between the genomes associated with typical Moko and Bugtok symptoms related to biological processes, molecular functions, and cellular components. The genome associated with Sergipe facies symptoms shared information related to molecular function and/or biological processes. No clusters were shared exclusively between the Bugtok and Sergipe facies genomes (Figure 1B and Table S3).

**Figure 2-:**
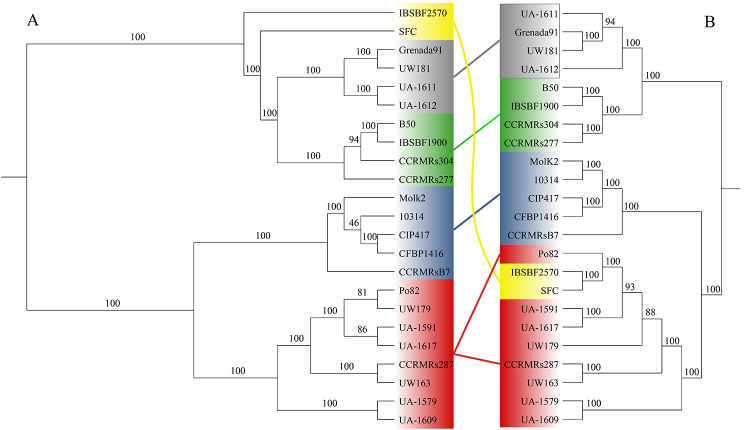
(A) Maximum likelihood phylogenetic tree of core gene sequences annotated for the *Ralstonia solanacearum* Moko ecotype genomes and its symptomatological variants. (B) Maximum likelihood phylogenetic tree based on the presence or absence of orthologous genes of *R. solanacearum* Moko ecotype genomes and its symptomatological variants. * Subgroup IIA(α) is shown in green, IIA(β) in black, IIB(α) in blue, and IIB(β) in red. Strains of Sergipe facies (IBSBF2570 and SFC, IIA- β) are shown yellow. Strains of Moko ecotype causing Bugtok symptoms (CIP417 and Molk2), Sergipe facies (IBSBF2570 and SFC), and typical symptoms of Moko (all other strains) are shown.

The phylogenomic trees showed strong bootstrap support in all branches, indicating a robust phylogeny for *R. solanacearum.* As indicated by ANIm and *is*DDH*,* the phylogenomic tree based on core genes distinguished clearly the two phylotypes, IIA and IIB, and the four subgroups IIA(α), IIA(β), IIB(α), and IIB(β) (Figure 2A). However, inference based on the presence and absence of gene clusters could not group the genomes of the Moko ecotype in the same clusters as done previously in this work, because the genomes IBSBF2570 and SFC (phylotype IIA) did not group with other representatives of subgroup IIA(β) (Figure 2B).

## Discussion

Based on these results, the ANIm and *is*DDH analyses were not sufficient to discriminate between strains with different Moko ecotype symptoms (Figure S1). However, the genomes of Bugtok disease and Sergipe facies variants presented high similarity between their representatives, proving a high genomic homogeneity within these groups (Table 2). Thus, the ANIm and *is*DDH analyses proved that the similarity of symptoms did not correspond with greater genomic proximity between organisms.

However, the pan-genome of the strain of typical Moko symptoms, Bugtok disease, and Sergipe facies variants shared 1,951 homologous genes, or 21.3% of inheritable regions for the entire Moko ecotype. Based on this result and the hypotheses proposed by Ailloud et al. (2015), we conclude that the Moko ecotype may have inherited pathogenic traits from a recent common ancestor by sharing some homologous genes. Ailloud *et al*. (2015) evaluated groups of pathogens highly adapted to hosts, which included representatives of the *R. solanacearum* Moko strains. These strains were characterized by the absence of exclusive homologous regions, suggesting that this ecotype might have arisen from the convergent evolution of several strains, which led to the ability to infect banana trees.

For *R. solanacearum* representatives with typical Moko symptoms, no clusters of homologous genes were found. Instead, Sergipe facies representatives contained the largest number (113) of exclusive clusters, of which 51.1% were observed in both strains, indicating greater diversity within the group. This observation may be related to the high rate of mutation necessary to ensure the prevalence of this trait in the environment, considering that it is the most recently reported symptomatological group (Albuquerque et al., 2014; Silva et al., 2020). Within the exclusive clusters, 13.3% were associated with T3SS and 10% with insertion sequences, which could favor various genetic rearrangements. *R. solanacearum* uses a T3SS to deliver effector proteins, which manipulate the host physiology to increase pathogen success. Insertion sequences are mobile genetic elements that are commonly present in bacteria. They can lead to gene activation or repression, as well as DNA rearrangements, resulting in deletions, inversions, and amplification of genes (Chandler and Mahillon, 2002). These insertion sequences demonstrate the process of horizontal transfer of genetic information, known to be an important mechanism for the evolution of the bacterial genome, as evidenced in Blood Disease Bacterium (BDB; Remenant et al., 2011), corroborating this process in the genomes of Sergipe facies. 

Strains with typical Moko and Bugtok symptoms not were differentiated by phylogenetic analyses of the endoglucanase (*egl*) gene (Fegan and Prior, 2006). The sequence of the *egl* genes is used to determine a phylogenetic relationship among isolates of *R. solanacearum*, differentiating them by sequevar. However, the results obtained in the current study identified four unique ortholog clusters in strains representative of Bugtok symptoms, which can be used to distinguish these two symptomatological conditions. The identified gene clusters included two transposase families, as well as two proteins of unknown function. Various insertion sequence families have been identified among *R. solanacearum*, most of them are scattered throughout the single strains. In addition, closely related strains tend to have similar insertion sequence patterns (Gonçalves et al., 2020).

Phylogenetic analysis of the genus *Ralstonia* successfully distinguished strains from phylotypes IIA and IIB (Zhang and Qiu, 2016). Phylotype IIA has been reported to be highly recombinogenic and diverse, with ongoing species expansion. In contrast, multilocus sequence analysis of nine loci has suggested the almost clonal character of phylotype IIB (Wicker et al., 2012). The description of phylotype IIA as recombinogenic and diverse may hint at the behavior of the genomes of Sergipe facies strains (IIA-53) observed in both phylogenomic analyses (Figure 2B) performed in the present study. In contrast, Bugtok strains (IIB-3) showed strong genetic similarity, which seems to confirm previous descriptions of phylotype IIB by Wicker *et al.* (2012). Finally, it is important to highlight that the analyses carried out in this study may have limitations due to the low quality of some genomes and the discrepancy in the number of representatives of the symptomatological group.

### Conclusions

The genome of the *R. solanacearum* Moko ecotype are strains that present high genomic similarity, chiefly among variants expressing Sergipe facies and Bugtok symptoms. Here, pan-genome analysis identified 21.3% of inheritable regions among representatives of the Moko ecotype, and the symptomatological Sergipe facies variant stood out for presenting the largest number of clusters of exclusive homologues. Accordingly, the similarity among symptomatic cases of Bugtok disease and Sergipe facies does not correspond with genomic or phylogenomic properties. Other approaches, possibly focusing on pathogenicity, virulence, and ecological factors, should be employed to determine a common denominator of different Moko ecotype symptoms. For example, little is known about the interactions between bacterial strains and insects responsible for the transmission of Bugtok and Sergipe facies. Knowing that both diseases occur by infection via inflorescence, this point may help understand the peculiarities of Moko pathogenesis and its symptoms.

## Supplementary material

The following online material is available for this article:

Table S1 - Core gene clusters obtained by pan-genome analysis of the *Ralstonia solanacearum* Moko ecotype and its symptomatological variants.

Table S2 - Clusters obtained by pan-genome analysis of the *Ralstonia solanacearum* Moko ecotype and its symptomatological variants.

Table S3 - Exclusive and shared gene clusters obtained by pan-genome analysis of the *Ralstonia solanacearum* Moko ecotype and its symptomatological variants.

Figure S1 - Heatmap generated from *in silico* DNA-DNA hybridization and average nucleotide identity using the MUMmer algorithm of *Ralstonia solanacearum* Moko ecotype genomes and its symptomatological variants. *The upper triangle refers to the values of *is*DDH and the lower triangle to the values of ANIm.
