# Supplementary material for: Comparative genomics and phylogenomics of the Ralstonia solanacearum Moko ecotype and its symptomatological variants
Source: Genet Mol Biol. 2022 Dec 2;45(4):e20220038. doi: 10.1590/1678-4685-GMB-2022-0038 (PMC9731368; doi:10.1590/1678-4685-GMB-2022-0038)
Supplement: Table S1 - [file 1415-4757-GMB-45-4-e20220038-s1.pdf]

Genetics and Molecular Biology
https://doi.org/10.1590/1678-4685-GMB-2022-0038

Supplementary material to "Comparative genomics and phylogenomics of the *Ralstonia solanacearum* Moko ecotype and its symptomatological variants"

Table S1 - Core gene clusters obtained by pan-genome analysis of the *Ralstonia solanacearum* Moko ecotype and its symptomatological variants.

Gene	Annotation
AQR24_RS04505	efflux RND transporter periplasmic adaptor subunit
cheB	chemotaxis response regulator protein-glutamate methylesterase
pdxH	pyridoxamine 5'-phosphate oxidase
C2L97_RS16060	AMP-binding protein
C2I38_RS16310	HAD-IA family hydrolase
C2I38_RS16350	homoserine O-acetyltransferase
EIH09_RS06400	YbdD/YjiX family protein
dnaA	chromosomal replication initiator protein DnaA
RALGR_RS09115	2-dehydropantoate 2-reductase
HXP37_04555	response regulator transcription factor
RALW1_RS02690	alpha/beta hydrolase
EIH10_RS02715	LysR family transcriptional regulator
AQR21_RS16635	LysR family transcriptional regulator
RALW1_RS02615	short chain dehydrogenase
rsmG	16S rRNA (guanine(527)-N(7))-methyltransferase RsmG
C2L97_RS00650	alpha/beta hydrolase
UW163_RS04860	hypothetical protein
RSMK_RS04000	GFA family protein
EIH14_RS21230	cupin domain-containing protein
hyi	hydroxypyruvate isomerase
AQR24_RS13110	ureidoglycolate lyase
C2I33_RS04545	hypothetical protein
soxX	sulfur oxidation c-type cytochrome SoxX
RSMK_RS03220	2-hydroxyacid dehydrogenase
EIH12_RS06010	general secretion pathway protein GspM
gspH	prepilin-type N-terminal cleavage/methylation domain-containing protein
EIH09_RS15185	DUF4437 domain-containing protein
HXP36_03085	NAD(P)H dehydrogenase
EIH10_RS01460	hypothetical protein
HXP36_03245	hypothetical protein

Gene	Annotation
AQR21_RS22705	glutathione S-transferase
EIH11_RS09290	MipA/OmpV family protein
RALFB_RS12085	response regulator transcription factor
infA	translation initiation factor IF-1
rpoA	DNA-directed RNA polymerase subunit alpha
cyaY	iron donor protein CyaY
RSPO_RS02465	penicillin-binding protein 1A
pilQ	type IV pilus secretin PilQ
UW163_RS06690	OmpW family protein
gltD	glutamate synthase subunit beta
mlaD	outer membrane lipid asymmetry maintenance protein MlaD
RALW1_RS23125	UbiX family flavin prenyltransferase
HXP37_07065	hypothetical protein
RALCI_RS08985	oxidoreductase
AQR24_RS10060	CYTH domain-containing protein
EIH10_RS14360	ABC transporter substrate-binding protein
pfkB	1-phosphofructokinase
RALB5_RS09320	D-alanine--D-alanine ligase
EIH14_RS20305	esterase
RSPO_RS03475	chorismate lyase
RALB5_RS11090	phosphatidylglycerophosphatase A
EIH13_RS06110	ribonuclease N
RALGR_RS03925	TonB-dependent siderophore receptor
RSMK_RS01195	DUF1624 domain-containing protein
EIH14_RS20085	MarR family transcriptional regulator
mnmA	tRNA 2-thiouridine(34) synthase MnmA
RALB5_RS20850	Zn-dependent hydrolase
ubiA	4-hydroxybenzoate octaprenyltransferase
HXP37_08065	pyrroline-5-carboxylate reductase
RALGR_RS04455	type IV pilin protein
RALFB_RS11135	prepilin-type N-terminal cleavage/methylation domain-containing protein
HXP37_08100	PilW family protein
HXP34_04360	hypothetical protein
RALFB_RS16920	deoxynucleoside kinase
RALW1_RS11520	DNA-3-methyladenine glycosylase 2 family protein
miaA	tRNA (adenosine(37)-N6)-dimethylallyltransferase MiaA
murB	UDP-N-acetylmuramate dehydrogenase
RALW1_RS11395	methylated-DNA--[protein]-cysteine S-methyltransferase
phbB	acetoacetyl-CoA reductase

Gene	Annotation
HXP35_04735	MFS transporter
HXP34_04835	zinc metallopeptidase
dapA	4-hydroxy-tetrahydrodipicolinate synthase
EIH12_RS02545	LysR family transcriptional regulator
EIH11_RS08325	MBL fold metallo-hydrolase
radC	JAB domain-containing protein
RALB5_RS05895	GNAT family N-acetyltransferase
RSMK_RS00350	sensor domain-containing diguanylate cyclase
RALCI_RS05505	5-carboxymethyl-2-hydroxymuconate Delta-isomerase
AQR21_RS18540	cobalamin biosynthesis protein CbiX
cobU	bifunctional adenosylcobinamide kinase/adenosylcobinamide-phosphate guanylyltransferase
EIH13_RS09160	pantoate--beta-alanine ligase
EIH12_RS12865	4Fe-4S binding protein
C2I33_RS18280	formate dehydrogenase subunit gamma
RALB5_RS20055	class I SAM-dependent methyltransferase
EIH14_RS07690	replication-associated recombination protein A
RALB5_RS05675	peptidase S53
RSMK_RS11445	HlyC/CorC family transporter
RALW1_RS19940	amino acid ABC transporter permease
pobA	4-hydroxybenzoate 3-monooxygenase
HXP35_05885	homoserine kinase
RALFB_RS07980	NAD(P)/FAD-dependent oxidoreductase
C2L97_RS05915	MBL fold metallo-hydrolase
AQR24_RS17430	LysR family transcriptional regulator
EIH13_RS15175	hypothetical protein
EIH11_RS06690	multidrug effflux MFS transporter
RSMK_RS11980	DUF2968 domain-containing protein
EIH13_RS02020	oxygen-independent coproporphyrinogen III oxidase-like protein
EIH12_RS03035	NAD(P)-dependent alcohol dehydrogenase
AQR24_RS12045	hypothetical protein
AQR21_RS12590	LysR family transcriptional regulator
EIH09_RS16900	solute-binding protein
EIH11_RS06990	DUF924 domain-containing protein
RSPO_RS06630	DUF2818 family protein
C2I38_RS06810	acyl-CoA dehydrogenase
RALFB_RS24400	hypothetical protein
C2L97_RS07190	polyketide cyclase
C2L97_RS07290	peroxidase-related enzyme
RALFB_RS04885	universal stress protein

Gene	Annotation
C2I33_RS18800	zinc-dependent alcohol dehydrogenase family protein
C2L97_RS07735	sulfite exporter TauE/SafE family protein
HXP34_07790	FKBP-type peptidyl-prolyl cis-trans isomerase
RALFB_RS15415	amidohydrolase family protein
C2I33_RS18095	TM2 domain-containing protein
RALB5_RS16405	NUDIX hydrolase
EIH13_RS08490	quinone-dependent dihydroorotate dehydrogenase
RALB5_RS16440	RNA-binding transcriptional accessory protein
phaC	class I poly(R)-hydroxyalkanoic acid synthase
HXP34_08155	tRNA-dihydrouridine synthase
EIH13_RS08355	2-C-methyl-D-erythritol 2,4-cyclodiphosphate synthase
pgi	glucose-6-phosphate isomerase
HXP36_10495	ABC transporter ATP-binding protein
RALW1_RS17240	acyl-CoA dehydrogenase
EIH14_RS05895	folate-binding protein YgfZ
group_1383	polyhydroxyalkanoate depolymerase
EIH13_RS22725	(2Fe-2S)-binding protein
RALGR_RS01470	MarR family transcriptional regulator
EIH11_RS15520	heavy metal translocating P-type ATPase
RSPO_RS09965	HAD family hydrolase
ntrC	nitrogen regulation protein NR(I)
RALW1_RS09120	competence/damage-inducible protein A
RALFB_RS10105	NCS1 family nucleobase:cation symporter-1
HXP36_12415	carbohydrate porin
EIH09_RS02165	antibiotic biosynthesis monooxygenase
HXP37_12610	sigma-54-dependent Fis family transcriptional regulator
RSMK_RS07740	cysteine--tRNA ligase
cysE	serine O-acetyltransferase
HXP34_10145	AEC family transporter
earP	elongation factor P maturation arginine rhamnosyltransferase EarP
moaA	GTP 3',8-cyclase MoaA
HXP36_13240	molybdopterin molybdenumtransferase MoeA
rmuC	DNA recombination protein RmuC
EIH13_RS12430	D-glycerate dehydrogenase
HXP36_13260	sodium:proton antiporter
iscX	Fe-S cluster assembly protein IscX
HXP37_11725	LacI family transcriptional regulator
RALB5_RS03805	cytochrome c
C2I38_RS11600	GNAT family N-acetyltransferase

Gene	Annotation
UW163_RS15700	alanyl-tRNA editing protein
HXP35_10790	glutathione S-transferase
EIH11_RS10880	hypothetical protein
UW163_RS15850	ribosome-associated protein
mog	molybdopterin adenylyltransferase
rimM	ribosome maturation factor RimM
EIH13_RS10200	hypothetical protein
EIH11_RS10715	D-amino acid dehydrogenase
HXP34_10935	DUF1049 domain-containing protein
EIH13_RS10345	DUF2059 domain-containing protein
EIH12_RS09585	glycine zipper 2TM domain-containing protein
EIH14_RS13490	LysR family transcriptional regulator
C2I38_RS12470	alkene reductase
EIH10_RS03335	hypothetical protein
EIH09_RS11245	purine nucleoside permease
EIH13_RS14625	membrane protein
EIH12_RS02635	ankyrin repeat domain-containing protein
msrA	peptide-methionine (S)-S-oxide reductase MsrA
AQR24_RS05760	DUF3318 domain-containing protein
RSPO_RS12575	nucleoside 2-deoxyribosyltransferase
tolA	cell envelope integrity protein TolA
RALGR_RS02125	hypothetical protein
EIH13_RS19900	DUF4337 domain-containing protein
HXP37_00755	NADH:flavin oxidoreductase/NADH oxidase
RALFB_RS14410	YkgJ family cysteine cluster protein
RALB5_RS05520	TolC family outer membrane protein
group_1473	lipopolysaccharide heptosyltransferase I
ruvX	Holliday junction resolvase RuvX
RSPO_RS12950	deoxyribodipyrimidine photo-lyase
HXP36_15090	AAA family ATPase
C2I33_RS17430	hypothetical protein
UW163_RS01445	AzlD domain-containing protein
RALW1_RS15855	DUF2889 domain-containing protein
recX	recombination regulator RecX
RALGR_RS20340	DUF4212 domain-containing protein
RALGR_RS02635	M48 family metallopeptidase
C2L97_RS14120	histidine phosphatase family protein
EIH14_RS02540	DUF3025 domain-containing protein
UW163_RS02000	glycerate kinase

Gene	Annotation
RSPO_RS14185	peptidase C39
C2I33_RS20380	hypothetical protein
RALB5_RS13620	glycosyltransferase family 2 protein
RALGR_RS03940	outer membrane lipoprotein carrier protein LolA
AQR24_RS23120	hypothetical protein
EIH10_RS10165	low specificity L-threonine aldolase
RALB5_RS00890	DJ-1/PfpI family protein
C2L97_RS14765	pirin family protein
HXP36_16550	SURF1 family protein
coxB	cytochrome c oxidase subunit II
RALFB_RS24720	DUF2244 domain-containing protein
RALGR_RS17730	SDR family oxidoreductase
RALW1_RS23185	ABC transporter ATP-binding protein
C2L97_RS15605	HlyD family efflux transporter periplasmic adaptor subunit
AQR24_RS24465	M48 family metallopeptidase
C2I38_RS15905	acyl-CoA dehydrogenase
HXP37_03550	hypothetical protein
RSMK_RS20915	phage tail protein
thiC	phosphomethylpyrimidine synthase ThiC
RALFB_RS02185	hypothetical protein
RSPO_RS15725	aspartate aminotransferase family protein
C2I38_RS16655	MarR family transcriptional regulator
blaOXA	class D beta-lactamase
EIH12_RS19995	carbohydrate porin
EIH14_RS08020	YciI family protein
RALGR_RS18895	hypothetical protein
HXP37_16435	YhfC family intramembrane metalloprotease
RALCI_RS22300	lysine N(6)-hydroxylase/L-ornithine N(5)-oxygenase family protein
EIH11_RS02345	hypothetical protein
RALW1_RS21495	hypothetical protein
C2I33_RS15235	efflux RND transporter periplasmic adaptor subunit
C2L97_RS23650	LysR family transcriptional regulator
C2I33_RS11615	aldehyde dehydrogenase family protein
EIH13_RS19230	amino acid ABC transporter permease
EIH09_RS14610	M81 family metallopeptidase
RSMK_RS05685	cytochrome oxidase subunit III
C2I38_RS23685	phosphogluconate dehydratase
UW163_RS17350	hypothetical protein
EIH09_RS06705	sensor domain-containing diguanylate cyclase

Gene	Annotation
RALFB_RS14745	multidrug/biocide efflux PACE transporter
AQR21_RS03020	U32 family peptidase
C2I33_RS02880	zinc-binding protein
C2L97_RS18685	NnrS family protein
RSPO_RS18340	hypothetical protein
RALB5_RS01155	NAD(P)H-dependent oxidoreductase
cobM	precorrin-4 C(11)-methyltransferase
RSMK_RS14795	amino acid ABC transporter permease
waaC	lipopolysaccharide heptosyltransferase I
AQR24_RS24435	acyltransferase
UW163_RS19265	helix-turn-helix transcriptional regulator
RALGR_RS18610	DUF962 domain-containing protein
atpC	F0F1 ATP synthase subunit epsilon
HXP36_20260	aldehyde dehydrogenase (NADP(+))
C2I33_RS21945	leucine-rich repeat domain-containing protein
UW163_RS19515	helix-turn-helix transcriptional regulator
RALGR_RS07065	FecR family protein
HXP34_18010	type III secretion protein HrpB7
RSMK_RS16095	DUF3141 domain-containing protein
group_1660	enoyl-ACP reductase FabI
EIH13_RS03330	hypothetical protein
RALCI_RS15015	response regulator transcription factor
RALB5_RS04420	helix-turn-helix transcriptional regulator
RSMK_RS00180	cobalamin-binding protein
AQR21_RS12150	DUF692 domain-containing protein
HXP34_18870	TolC family protein
C2I33_RS04875	efflux RND transporter periplasmic adaptor subunit
RALB5_RS18185	hypothetical protein
fdhD	formate dehydrogenase accessory sulfurtransferase FdhD
EIH10_RS20325	glycoside hydrolase family 32 protein
EIH11_RS05380	DUF2968 domain-containing protein
HXP36_21730	Flp family type IVb pilin
EIH10_RS03840	MdtA/MuxA family multidrug efflux RND transporter periplasmic adaptor subunit
HXP34_19695	hypothetical protein
RSMK_RS17435	alpha/beta hydrolase
HXP36_22335	pyridoxal phosphate-dependent aminotransferase
RALFB_RS24080	helix-turn-helix transcriptional regulator
HXP36_22525	serine hydrolase
mdtD	DHA2 family efflux MFS transporter permease subunit

Gene	Annotation
C2I33_RS06825	ABC transporter ATP-binding protein
RALFB_RS12845	ABC transporter ATP-binding protein
HXP37_21745	hypothetical protein
EIH14_RS09890	hypothetical protein
RALB5_RS03550	hypothetical protein
C2I38_RS19420	arginase family protein
tynA	primary-amine oxidase
HXP37_22805	NAD(P)-dependent alcohol dehydrogenase
AQR24_RS19005	alcohol dehydrogenase catalytic domain-containing protein
EIH14_RS16920	winged helix-turn-helix transcriptional regulator
EIH12_RS21720	ferrous iron transport protein A
C2I38_RS17315	hypothetical protein
EIH12_RS21870	hypothetical protein
AQR21_RS10675	MATE family efflux transporter
prpR	propionate catabolism operon regulatory protein PrpR
RALGR_RS22190	hypothetical protein
C2I38_RS16840	TetR/AcrR family transcriptional regulator
RALFB_RS21730	peroxidase-related enzyme
C2I33_RS18060	acyl-CoA thioesterase
EIH13_RS11550	TetR/AcrR family transcriptional regulator
HXP35_10665	recombinase
RSPO_RS12510	IclR family transcriptional regulator
EIH14_RS13165	NCS2 family permease
EIH12_RS15380	M48 family metallopeptidase
RALCI_RS18815	TolC family protein
group_1892	N-acetyl-gamma-glutamyl-phosphate reductase
C2I33_RS21775	hypothetical protein
group_1909	3,4-dihydroxy-2-butanone-4-phosphate synthase
C2I38_RS23550	nitronate monooxygenase
RALGR_RS10710	PhoPQ-regulated protein
HXP37_14085	winged helix-turn-helix transcriptional regulator
sctL	type III secretion system stator protein SctL
UW163_RS20715	MarR family transcriptional regulator
amt	ammonium transporter
AQR21_RS17150	MFS transporter
bluB	5,6-dimethylbenzimidazole synthase
EIH14_RS16325	response regulator
RSPO_RS15760	DUF4390 domain-containing protein
EIH09_RS06150	peptide deformylase

Gene	Annotation
EIH13_RS17870	LysM peptidoglycan-binding domain-containing protein
rodA	rod shape-determining protein RodA
HXP34_00105	rod shape-determining protein
RSPO_RS15305	hypothetical protein
gatC	Asp-tRNA(Asn)/Glu-tRNA(Gln) amidotransferase subunit GatC
HXP37_03855	class I SAM-dependent rRNA methyltransferase
HXP37_03870	transcriptional repressor
RALFB_RS00330	ABC transporter substrate-binding protein
EIH12_RS09185	copper chaperone PCu(A)C
EIH12_RS09170	zf-HC2 domain-containing protein
gabT	4-aminobutyrate--2-oxoglutarate transaminase
RALB5_RS06490	NAD-dependent succinate-semialdehyde dehydrogenase
metW	methionine biosynthesis protein MetW
group_2091	phasin family protein
HXP36_00960	sel1 repeat family protein
EIH10_RS06855	histidine kinase
EIH09_RS06430	response regulator
HXP36_01330	membrane protein
EIH09_RS20050	Rrf2 family transcriptional regulator
UW163_RS04420	sensor histidine kinase
kdpF	K(+)-transporting ATPase subunit F
EIH11_RS12660	hypothetical protein
EIH09_RS22945	helix-turn-helix transcriptional regulator
rlmJ	23S rRNA (adenine(2030)-N(6))-methyltransferase RlmJ
HXP37_04540	HU family DNA-binding protein
C2I33_RS16100	sensor histidine kinase
cueR	Cu(I)-responsive transcriptional regulator
EIH12_RS05615	ABC transporter substrate-binding protein
C2I33_RS16190	branched-chain amino acid ABC transporter permease
RSMK_RS03825	TSUP family transporter
C2I38_RS00560	ABC transporter substrate-binding protein
C2I33_RS10775	ATP synthase subunit I
atpB	F0F1 ATP synthase subunit A
HXP35_01025	DUF3562 domain-containing protein
gstA	glutathione transferase GstA
EIH10_RS19610	LysR family transcriptional regulator
AQR21_RS06380	hypothetical protein
C2I38_RS00865	LysR family transcriptional regulator
HXP34_01265	GntR family transcriptional regulator

Gene	Annotation
soxY	thiosulfate oxidation carrier protein SoxY
soxA	sulfur oxidation c-type cytochrome SoxA
AQR21_RS22595	MarR family transcriptional regulator
C2I38_RS01660	ferritin-like domain-containing protein
RALW1_RS16400	N-acetyltransferase
gspE	type II secretion system ATPase GspE
gspD	type II secretion system secretin GspD
AQR21_RS14495	general secretion pathway protein GspL
gspK	type II secretion system minor pseudopilin GspK
HXP35_02190	M20/M25/M40 family metallo-hydrolase
C2I38_RS01930	DUF1484 domain-containing protein
AQR24_RS16770	cation transporter
RALCI_RS22655	hypothetical protein
HXP34_02395	DUF2160 domain-containing protein
AQR21_RS11470	carbohydrate ABC transporter permease
C2L97_RS02135	ABC transporter ATP-binding protein
rplJ	50S ribosomal protein L10
EIH09_RS23895	DUF2135 domain-containing protein
UW163_RS06395	class I SAM-dependent methyltransferase
rplB	50S ribosomal protein L2
rplF	50S ribosomal protein L6
EIH11_RS20985	divalent-cation tolerance protein CutA
C2I38_RS02490	YihA family ribosome biogenesis GTP-binding protein
C2I33_RS02090	ATP-binding protein
RALW1_RS02725	PilN domain-containing protein
pilO	type 4a pilus biogenesis protein PilO
EIH12_RS06935	deoxyguanosinetriphosphate triphosphohydrolase
EIH09_RS15375	tripartite tricarboxylate transporter substrate binding protein
HXP34_02865	VacJ family lipoprotein
RALCI_RS12650	phospholipid-binding protein MlaC
HXP36_03870	STAS domain-containing protein
AQR24_RS09205	NAAT family transporter
EIH12_RS06805	membrane protein
C2I33_RS02335	sensor histidine kinase
RSMK_RS02325	Nif3-like dinuclear metal center hexameric protein
AQR21_RS03740	cytochrome c1
RALB5_RS19655	ClpXP protease specificity-enhancing factor
cyoC	cytochrome o ubiquinol oxidase subunit III
HXP36_04255	cytochrome o ubiquinol oxidase subunit IV

Gene	Annotation
mdoH	glucans biosynthesis glucosyltransferase MdoH
EIH09_RS15670	hypothetical protein
grxD	Grx4 family monothiol glutaredoxin
HXP35_03215	flagellar protein FliT
HXP36_04345	lytic transglycosylase domain-containing protein
EIH10_RS18200	DsbC family protein
C2I38_RS03000	FUSC family protein
trpD	anthranilate phosphoribosyltransferase
C2I33_RS24435	phosphoglycolate phosphatase
rpe	ribulose-phosphate 3-epimerase
HXP34_03295	hypothetical protein
EIH14_RS13565	murein transglycosylase A
paaI	hydroxyphenylacetyl-CoA thioesterase PaaI
argE	acetylornithine deacetylase
HXP37_07270	molybdopterin oxidoreductase family protein
RALFB_RS07000	MHS family MFS transporter
HXP34_03430	porin
EIH10_RS14475	cell division protein FtsQ/DivIB
C2I38_RS03305	M23 family metallopeptidase
secA	preprotein translocase subunit SecA
RALW1_RS08620	NUDIX domain-containing protein
yacG	DNA gyrase inhibitor YacG
RALCI_RS17100	dephospho-CoA kinase
RALFB_RS13065	HlyC/CorC family transporter
HXP37_07480	hypothetical protein
ampD	1,6-anhydro-N-acetylmuramyl-L-alanine amidase AmpD
prmA	50S ribosomal protein L11 methyltransferase
aroQ	type II 3-dehydroquinate dehydratase
HXP35_03775	TlpA family protein disulfide reductase
C2I33_RS03765	hypothetical protein
UW163_RS07745	16S rRNA (uracil(1498)-N(3))-methyltransferase
group_2268	outer membrane protein assembly factor BamE
C2L97_RS03705	class I SAM-dependent RNA methyltransferase
C2L97_RS03725	class I SAM-dependent methyltransferase
HXP36_05145	NUDIX hydrolase
EIH10_RS15035	cytochrome b
EIH11_RS19745	polyisoprenoid-binding protein
secD	protein translocase subunit SecD
queA	tRNA preQ1(34) S-adenosylmethionine ribosyltransferase-isomerase QueA

Gene	Annotation
recG	ATP-dependent DNA helicase RecG
yddG	drug/metabolite DMT transporter permease
RSPO_RS03900	pilus assembly protein
HXP34_04265	cupin domain-containing protein
C2L97_RS04020	VOC family protein
RALCI_RS02430	CidA/LrgA family protein
C2I38_RS04080	FAD-binding protein
EIH11_RS08930	mechanosensitive ion channel family protein
aroG	3-deoxy-7-phosphoheptulonate synthase AroG
rrtA	rhombosortase
hutH	histidine ammonia-lyase
AQR24_RS01275	hypothetical protein
grpE	nucleotide exchange factor GrpE
rnk	nucleoside diphosphate kinase regulator
RALB5_RS04545	energy-coupling factor ABC transporter ATP-binding protein
panB	3-methyl-2-oxobutanoate hydroxymethyltransferase
HXP36_05655	HAD-IB family hydrolase
EIH12_RS06340	AI-2E family transporter
EIH13_RS05435	MCE family protein
C2I38_RS04350	TetR/AcrR family transcriptional regulator
murJ	murein biosynthesis integral membrane protein MurJ
argG	argininosuccinate synthase
xerD	site-specific tyrosine recombinase XerD
C2I33_RS09765	biopolymer transporter ExbD
EIH11_RS08445	sulfite exporter TauE/SafE family protein
C2L97_RS04570	hypothetical protein
C2I38_RS04630	DNA-3-methyladenine glycosylase 2 family protein
EIH11_RS08355	gamma-glutamyltransferase family protein
HXP36_06125	DUF4126 domain-containing protein
lspA	lipoprotein signal peptidase
ileS	isoleucine--tRNA ligase
C2I38_RS04840	bifunctional riboflavin kinase/FAD synthetase
HXP37_08935	mechanosensitive ion channel family protein
EIH14_RS19040	carboxylating nicotinate-nucleotide diphosphorylase
nadB	L-aspartate oxidase
EIH13_RS09425	branched-chain amino acid ABC transporter substrate-binding protein
EIH10_RS12085	DUF934 domain-containing protein
lptG	LPS export ABC transporter permease LptG
HXP35_05250	EVE domain-containing protein

Gene	Annotation
C2I33_RS23975	hypothetical protein
EIH11_RS18100	ParA family protein
AQR21_RS08070	outer membrane protein assembly factor
RSMK_RS00105	DUF3305 domain-containing protein
C2L97_RS05210	4Fe-4S dicluster domain-containing protein
EIH11_RS18000	superoxide dismutase family protein
AQR24_RS06890	sigma-70 family RNA polymerase sigma factor
hemC	hydroxymethylbilane synthase
HXP37_09460	PepSY domain-containing protein
EIH13_RS08945	MFS transporter
pelF	DUF3492 domain-containing protein
RALW1_RS19935	amino acid ABC transporter permease
EIH12_RS22870	ABC transporter substrate-binding protein
EIH14_RS12330	3-oxoacid CoA-transferase subunit A
pcaD	3-oxoadipate enol-lactonase
pcaC	4-carboxymuconolactone decarboxylase
EIH13_RS14845	hypothetical protein
EIH14_RS12220	membrane protein
polA	DNA polymerase I
HXP35_05905	NAD(P)/FAD-dependent oxidoreductase
C2L97_RS05850	sulfurtransferase
EIH13_RS14920	DMT family transporter
RALFB_RS08005	exodeoxyribonuclease VII small subunit
HXP35_05965	GatB/YqeY domain-containing protein
EIH10_RS01765	DEAD/DEAH box helicase
EIH11_RS06510	pirin family protein
HXP36_07455	sulfurtransferase
C2L97_RS05975	glycosyltransferase
RSPO_RS05900	hypothetical protein
rsfS	ribosome silencing factor
EIH11_RS06625	SDR family oxidoreductase
HXP36_07570	methylglyoxal synthase
EIH10_RS01615	radical SAM protein
AQR24_RS03660	ABC transporter permease
RSMK_RS11970	TRAP transporter substrate-binding protein
pcp	pyroglutamyl-peptidase I
rph	ribonuclease PH
RALW1_RS14410	hypothetical protein
uraD	2-oxo-4-hydroxy-4-carboxy-5-ureidoimidazoline decarboxylase

Gene	Annotation
EIH11_RS06940	TMEM165/GDT1 family protein
guaD	guanine deaminase
xdhA	xanthine dehydrogenase small subunit
RALFB_RS09925	LysR family transcriptional regulator
EIH13_RS02260	LysR family transcriptional regulator
EIH09_RS16925	LysE family translocator
C2L97_RS06560	TIGR00730 family Rossman fold protein
EIH13_RS02315	RDD family protein
C2I33_RS05175	DUF3619 family protein
EIH14_RS11440	zinc-binding dehydrogenase
RALFB_RS09780	triose-phosphate isomerase
AQR21_RS13925	glutathione S-transferase
EIH11_RS19715	GntR family transcriptional regulator
truA	tRNA pseudouridine(38-40) synthase TruA
EIH14_RS04845	phosphoribosylanthranilate isomerase
hemP	hemin uptake protein HemP
RSMK_RS12675	glutamate racemase
HXP37_11430	ABC transporter substrate-binding protein
HXP37_11445	hypothetical protein
EIH09_RS20475	DUF4743 domain-containing protein
C2I38_RS07590	DUF2892 domain-containing protein
carA	glutamine-hydrolyzing carbamoyl-phosphate synthase small subunit
EIH13_RS07535	DUF4149 domain-containing protein
folP	dihydropteroate synthase
pstA	phosphate ABC transporter permease PstA
sixA	phosphohistidine phosphatase SixA
C2I33_RS13365	MBL fold metallo-hydrolase
RALW1_RS12730	hypothetical protein
EIH12_RS00295	O-acetylhomoserine aminocarboxypropyltransferase
HXP35_07760	SDR family oxidoreductase
EIH12_RS00350	alpha/beta hydrolase
HXP36_09830	RidA family protein
EIH10_RS05565	bifunctional (p)ppGpp synthetase/guanosine-3',5'-bis(diphosphate) 3'-pyrophosphohydrolase
infC	translation initiation factor IF-3
UW163_RS15075	integration host factor subunit alpha
C2I38_RS08100	aldose epimerase
dinB	DNA polymerase IV
AQR24_RS11090	hypothetical protein
phaP	phasin family protein

Gene	Annotation
RSMK_RS13695	histone deacetylase
EIH09_RS04605	DMT family transporter
EIH12_RS00550	(Fe-S)-binding protein
C2I38_RS08245	iron-sulfur cluster-binding protein
C2I38_RS08285	hypothetical protein
trkD	potassium transporter Kup
EIH09_RS04490	RluA family pseudouridine synthase
phaR	polyhydroxyalkanoate synthesis repressor PhaR
rimO	30S ribosomal protein S12 methylthiotransferase RimO
RALFB_RS03865	carboxymuconolactone decarboxylase family protein
hpnD	presqualene diphosphate synthase HpnD
clpX	ATP-dependent Clp protease ATP-binding subunit ClpX
purL	phosphoribosylformylglycinamidine synthase
HXP35_08580	ABC transporter substrate-binding protein
msrB	peptide-methionine (R)-S-oxide reductase MsrB
EIH13_RS08090	branched-chain amino acid ABC transporter substrate-binding protein
RALGR_RS22610	ABC transporter ATP-binding protein
EIH10_RS05385	glycoside hydrolase family 28 protein
C2I38_RS08720	3-hydroxyacyl-CoA dehydrogenase
C2L97_RS08710	SDR family oxidoreductase
RALB5_RS18495	PaaI family thioesterase
mltG	endolytic transglycosylase MltG
AQR24_RS19925	TatD family hydrolase
EIH11_RS14520	glutathione S-transferase family protein
EIH13_RS22710	MFS transporter
RSMK_RS09125	sulfite exporter TauE/SafE family protein
HXP34_09110	hypothetical protein
AQR21_RS05650	cbb3-type cytochrome oxidase subunit 3
ccoN	cytochrome-c oxidase, cbb3-type subunit I
HXP37_13085	hypothetical protein
lpdA	dihydrolipoyl dehydrogenase
AQR24_RS19340	hypothetical protein
C2I33_RS10295	hypothetical protein
HXP37_12995	EI24 domain-containing protein
C2I38_RS10240	sterol desaturase family protein
RSPO_RS10020	YncE family protein
iolB	5-deoxy-glucuronate isomerase
EIH13_RS11330	Rieske 2Fe-2S domain-containing protein
UW163_RS12870	TIM barrel protein

Gene	Annotation
rnr	ribonuclease R
HXP34_09405	phosphoribosyltransferase
RSMK_RS08810	ATP phosphoribosyltransferase regulatory subunit
EIH11_RS15235	DUF2065 domain-containing protein
hflK	FtsH protease activity modulator HflK
hflX	GTPase HflX
C2I33_RS07605	tetratricopeptide repeat protein
hisS	histidine--tRNA ligase
ndk	nucleoside-diphosphate kinase
rlmD	23S rRNA (uracil(1939)-C(5))-methyltransferase RlmD
EIH09_RS02195	ABC transporter ATP-binding protein
RALB5_RS20440	ABC transporter substrate-binding protein
AQR21_RS01640	YbaB/EbfC family nucleoid-associated protein
EIH14_RS10390	MATE family efflux transporter
EIH11_RS14990	glutamate--tRNA ligase
AQR24_RS16355	ABC transporter ATP-binding protein
RSPO_RS10510	hypothetical protein
HXP35_09900	peptidyl-prolyl cis-trans isomerase
EIH10_RS00265	UDP-2,3-diacylglucosamine diphosphatase
dkgB	2,5-didehydrogluconate reductase DkgB
mutS	DNA mismatch repair protein MutS
EIH10_RS00195	peptidylprolyl isomerase
EIH13_RS11855	cupin domain-containing protein
EIH11_RS04185	MBL fold metallo-hydrolase
C2I38_RS10905	DUF4166 domain-containing protein
C2I38_RS10915	alpha/beta hydrolase
ftsB	cell division protein FtsB
RSMK_RS18840	helix-turn-helix transcriptional regulator
HXP37_12145	LysR family transcriptional regulator
EIH14_RS22035	FadR family transcriptional regulator
RALGR_RS20455	response regulator transcription factor
uvrC	excinuclease ABC subunit UvrC
nagZ	beta-N-acetylhexosaminidase
RSPO_RS11010	holo-ACP synthase
EIH13_RS12285	DUF4845 domain-containing protein
EIH09_RS01445	sugar dehydratase
HXP36_13145	anti-sigma factor
fabF	beta-ketoacyl-ACP synthase II
group_2624	3-oxoacyl-ACP reductase FabG

Gene	Annotation
group_2626	septum formation protein Maf
EIH11_RS04695	SAM-dependent methyltransferase
EIH13_RS12385	Rieske (2Fe-2S) protein
prfB	peptide chain release factor 2
EIH14_RS22365	sugar ABC transporter substrate-binding protein
phaZ	polyhydroxyalkanoate depolymerase
rsxB	electron transport complex subunit RsxB
nth	endonuclease III
RSMK_RS19315	DMT family transporter
AQR24_RS08840	VWA domain-containing protein
C2L97_RS11640	chromate transporter
EIH12_RS01390	chromate transporter
egtD	L-histidine N(alpha)-methyltransferase
RALB5_RS11645	TerC family protein
HXP35_10905	threonine dehydratase
pmbA	metalloprotease PmbA
RSPO_RS11585	hypothetical protein
C2I33_RS15735	twin-arginine translocation signal domain-containing protein
RALB5_RS11585	CoA pyrophosphatase
EIH13_RS10195	sorbosone dehydrogenase family protein
RALW1_RS09570	TM2 domain-containing protein
mltB	lytic murein transglycosylase B
HXP35_11080	UDP-glucose/GDP-mannose dehydrogenase family protein
C2I38_RS11990	histidinol-phosphate transaminase
EIH14_RS04980	OmpA family protein
EIH14_RS11955	nicotinate-nucleotide adenylyltransferase
C2I33_RS08410	endoglucanase
RALCI_RS11060	NUDIX hydrolase
RALCI_RS11050	4-oxalocrotonate tautomerase
HXP37_00255	LysR family transcriptional regulator
RALFB_RS02500	hypothetical protein
RALGR_RS07685	TonB-dependent receptor
HXP36_14405	DUF2214 family protein
HXP36_07585	hypothetical protein
AQR21_RS16085	class I SAM-dependent methyltransferase
kynB	arylformamidase
kynA	tryptophan 2,3-dioxygenase
C2I33_RS00925	DUF883 family protein
RALB5_RS11235	ATP-binding cassette domain-containing protein

Gene	Annotation
fetB	iron export ABC transporter permease subunit FetB
EIH12_RS11330	DNA/RNA non-specific endonuclease
C2I33_RS00990	YeeE/YedE family protein
queC	7-cyano-7-deazaguanine synthase QueC
tolB	Tol-Pal system protein TolB
ybgC	tol-pal system-associated acyl-CoA thioesterase
HXP34_11660	6,7-dimethyl-8-ribityllumazine synthase
C2L97_RS12945	lytic transglycosylase domain-containing protein
EIH10_RS07990	NAD-dependent succinate-semialdehyde dehydrogenase
EIH13_RS19855	TetR/AcrR family transcriptional regulator
RALB5_RS05525	sulfurtransferase
waaA	3-deoxy-D-manno-octulosonic acid transferase
AQR21_RS06160	glycosyltransferase family 2 protein
RALFB_RS24635	glycosyltransferase family 2 protein
EIH11_RS18470	1-acyl-sn-glycerol-3-phosphate acyltransferase
pyrR	bifunctional pyr operon transcriptional regulator/uracil phosphoribosyltransferase PyrR
RSPO_RS12945	16S rRNA pseudouridine(516) synthase
RALFB_RS18235	purine-binding chemotaxis protein CheW
AQR24_RS09270	response regulator
EIH09_RS10590	rubredoxin
hemL	glutamate-1-semialdehyde 2,1-aminomutase
EIH11_RS18370	Flp family type IVb pilin
UW163_RS01045	hypothetical protein
C2L97_RS13235	CpaF family protein
EIH13_RS01160	glutathione S-transferase family protein
HXP36_15330	TonB-dependent receptor
EIH10_RS15270	hypothetical protein
RALW1_RS17115	threonylcarbamoyl-AMP synthase
AQR24_RS08140	phosphoribosylaminoimidazolesuccinocarboxamide synthase
pyk	pyruvate kinase
HXP36_15480	zinc-finger domain-containing protein
C2L97_RS13870	FAD-dependent monooxygenase
RALB5_RS20640	response regulator transcription factor
RALCI_RS12405	DUF748 domain-containing protein
RALB5_RS20655	serine/threonine protein kinase
HXP35_12680	hypothetical protein
ybeY	rRNA maturation RNase YbeY
RALCI_RS20360	glycine--tRNA ligase subunit beta
gmhB	D-glycero-beta-D-manno-heptose 1,7-bisphosphate 7-phosphatase

Gene	Annotation
rsmA	16S rRNA (adenine(1518)-N(6)/adenine(1519)-N(6))- dimethyltransferase RsmA
AQR21_RS20285	AzlD domain-containing protein
ruvC	crossover junction endodeoxyribonuclease RuvC
HXP36_15905	hypothetical protein
EIH12_RS07310	OsmC family protein
RALGR_RS04175	ABC transporter ATP-binding protein/permease
RALB5_RS13535	FAD/FMN-binding oxidoreductase
HXP34_12945	DedA family protein
EIH13_RS21665	hypothetical protein
UW163_RS02215	hypothetical protein
C2I33_RS20370	AMP-binding protein
fabG	3-oxoacyl-ACP reductase FabG
HXP36_16210	membrane protein
RALB5_RS00885	adenine phosphoribosyltransferase
EIH14_RS02155	ribbon-helix-helix domain-containing protein
RSPO_RS14355	potassium transporter
lptA	lipopolysaccharide transport periplasmic protein LptA
RALFB_RS01055	HPr kinase/phosphorylase
rapZ	RNase adapter RapZ
mutY	A/G-specific adenine glycosylase
mutM	bifunctional DNA-formamidopyrimidine glycosylase/DNA-(apurinic or apyrimidinic site) lyase
RSMK_RS22130	tetratricopeptide repeat protein
lolB	lipoprotein localization protein LolB
coaD	pantetheine-phosphate adenylyltransferase
maiA	maleylacetoacetate isomerase
ntrB	nitrate ABC transporter permease
RALCI_RS19825	heme A synthase
moeB	molybdopterin-synthase adenylyltransferase MoeB
gshA	glutamate--cysteine ligase
HXP37_02710	TlpA family protein disulfide reductase
RALCI_RS15805	alpha/beta hydrolase
HXP37_02800	ABC transporter ATP-binding protein
EIH13_RS20380	hypothetical protein
C2I38_RS15190	glutathione peroxidase
AQR21_RS12495	response regulator
UW163_RS02990	entericidin A/B family lipoprotein
HXP35_13945	DUF1840 domain-containing protein
EIH11_RS21670	thiol:disulfide interchange protein DsbA/DsbL
RALGR_RS22785	MBL fold metallo-hydrolase

Gene	Annotation
AQR21_RS12885	rhodanese-like domain-containing protein
HXP34_13910	glyoxylate/hydroxypyruvate reductase A
C2I33_RS20940	hypothetical protein
AQR21_RS09725	LysE family translocator
HXP35_14215	RtcB family protein
EIH13_RS23270	sigma 54-dependent transcriptional regulator
EIH11_RS16965	cupin domain-containing protein
AQR21_RS02700	MFS transporter
glmS	glutamine--fructose-6-phosphate transaminase (isomerizing)
RALGR_RS17720	UbiA family prenyltransferase
RALW1_RS05280	FAD-binding oxidoreductase
rocD	ornithine--oxo-acid transaminase
AQR21_RS00900	nitroreductase family protein
HXP37_03480	AraC family transcriptional regulator
C2I38_RS15850	phosphatase PAP2 family protein
RALFB_RS19480	glutathione S-transferase
RSMK_RS20910	phage tail protein
HXP34_14475	alkaline phosphatase
C2L97_RS15940	complex I NDUFA9 subunit family protein
EIH11_RS01650	hypothetical protein
HXP37_16270	SCO family protein
RALCI_RS20740	divalent metal cation transporter
RSMK_RS06440	YbhB/YbcL family Raf kinase inhibitor-like protein
EIH14_RS08035	ABC transporter ATP-binding protein
HXP36_17405	branched-chain amino acid ABC transporter permease
RSMK_RS06395	ABC transporter substrate-binding protein
galU	UTP--glucose-1-phosphate uridylyltransferase GalU
HXP35_15030	ParB/RepB/Spo0J family partition protein
EIH09_RS20805	ParA family protein
RALW1_RS20125	NCS2 family permease
EIH12_RS19845	RNA polymerase factor sigma-54
HXP35_15135	hypothetical protein
EIH11_RS02055	cytochrome c oxidase subunit II
EIH11_RS02100	MarR family transcriptional regulator
EIH09_RS21035	DMT family transporter
EIH14_RS08340	NAD(P)-dependent oxidoreductase
RSMK_RS06075	xylose ABC transporter ATP-binding protein
AQR21_RS11995	hypothetical protein
RSMK_RS05785	FAD-binding oxidoreductase

Gene	Annotation
AQR21_RS12035	cystathionine gamma-synthase family protein
RALCI_RS01895	helix-turn-helix transcriptional regulator
RALW1_RS04560	transporter substrate-binding domain-containing protein
EIH10_RS11300	LysR family transcriptional regulator
RALFB_RS19990	LysR family transcriptional regulator
C2I38_RS23725	cytochrome c oxidase subunit IV
RALB5_RS22770	hypothetical protein
C2L97_RS23365	PadR family transcriptional regulator
C2L97_RS23355	efflux transporter outer membrane subunit
RSPO_RS22830	response regulator transcription factor
EIH14_RS17230	HAMP domain-containing histidine kinase
cadR	Cd(II)/Pb(II)-responsive transcriptional regulator
RALW1_RS13660	threo-3-hydroxy-L-aspartate ammonia-lyase
C2I33_RS02805	RidA family protein
UW163_RS18025	DUF488 family protein
narJ	nitrate reductase molybdenum cofactor assembly chaperone
narI	respiratory nitrate reductase subunit gamma
mobB	molybdopterin-guanine dinucleotide biosynthesis protein B
EIH09_RS06935	iron-containing redox enzyme family protein
RALB5_RS01175	SGNH/GDSL hydrolase family protein
paaB	1,2-phenylacetyl-CoA epoxidase subunit B
paaC	phenylacetate-CoA oxygenase subunit PaaC
AQR21_RS04300	GTP-binding protein
RALGR_RS17120	ferredoxin protein
EIH11_RS15850	HoxN/HupN/NixA family nickel/cobalt transporter
EIH13_RS15815	amino acid ABC transporter permease
HXP36_20120	Crp/Fnr family transcriptional regulator
AQR21_RS21710	hypothetical protein
EIH12_RS04450	MFS transporter
EIH12_RS04530	sigma-70 family RNA polymerase sigma factor
EIH12_RS04570	hypothetical protein
sctU	type III secretion system export apparatus subunit SctU
AQR21_RS09910	HrpB1 family type III secretion system apparatus protein
UW163_RS19630	type III secretion protein HrpB2
EIH10_RS13545	hypothetical protein
EIH11_RS11160	tyrosine-type recombinase/integrase
RALFB_RS24480	phosphate acetyltransferase
EIH12_RS07850	lipoprotein
RALCI_RS14910	hypothetical protein

Gene	Annotation
EIH11_RS20755	DegT/DnrJ/EryC1/StrS aminotransferase family protein
EIH11_RS20750	acetyltransferase
EIH10_RS17100	heparinase II/III-family protein
RALW1_RS00830	glycosyltransferase family 4 protein
HXP36_21065	multidrug MFS transporter
RALCI_RS14990	polysaccharide biosynthesis tyrosine autokinase
RALB5_RS08815	low molecular weight phosphotyrosine protein phosphatase
EIH14_RS20930	multidrug MFS transporter
EIH14_RS20940	hypothetical protein
EIH13_RS03715	GNAT family N-acetyltransferase
RALCI_RS01535	Lrp/AsnC family transcriptional regulator
C2L97_RS22520	DUF861 domain-containing protein
EIH14_RS01370	DUF2282 domain-containing protein
RALGR_RS00380	APC family permease
AQR24_RS15435	patatin-like phospholipase family protein
ptsP	phosphoenolpyruvate--protein phosphotransferase
HXP36_21720	pilus assembly protein
AQR24_RS05145	amino acid ABC transporter substrate-binding protein
modB	molybdate ABC transporter permease subunit
UW163_RS20825	sulfate/molybdate ABC transporter ATP-binding protein
EIH09_RS12735	universal stress protein
acpA	acid phosphatase
RALB5_RS21150	MFS transporter
AQR21_RS13020	amino acid deaminase
AQR21_RS13005	RidA family protein
UW163_RS21000	response regulator
C2L97_RS20125	entericidin A/B family lipoprotein
C2I33_RS14875	Fic family protein
nirD	nitrite reductase small subunit NirD
EIH11_RS03990	LysR family transcriptional regulator
RALCI_RS00095	sulfite exporter TauE/SafE family protein
fumC	class II fumarate hydratase
C2I33_RS06895	type IV pilin protein
HXP34_19980	pilus assembly protein
HXP36_22640	pilus assembly protein
group_3107	type IV pilus modification protein PilV
EIH09_RS00805	polyamine aminopropyltransferase
EIH10_RS13115	alkylphosphonate utilization protein
EIH09_RS00885	YggT family protein

Gene	Annotation
AQR21_RS02370	RNA polymerase sigma factor FliA
flhB	flagellar type III secretion system protein FlhB
EIH13_RS13605	TetR/AcrR family transcriptional regulator
group_3116	protein phosphatase CheZ
cheD	chemoreceptor glutamine deamidase CheD
RALW1_RS04165	response regulator
flhC	flagellar transcriptional regulator FlhC
EIH12_RS05960	hypothetical protein
RALCI_RS17515	ABC transporter substrate-binding protein
C2L97_RS19345	SgcJ/EcaC family oxidoreductase
EIH09_RS01105	response regulator transcription factor
HXP36_23140	TolC family protein
EIH14_RS17050	flavodoxin family protein
ggt	gamma-glutamyltransferase
C2I38_RS17700	SDR family oxidoreductase
UW163_RS22750	PadR family transcriptional regulator
AQR24_RS16650	hypothetical protein
ahpF	alkyl hydroperoxide reductase subunit F
RALW1_RS07005	hypothetical protein
cysC	adenylyl-sulfate kinase
AQR24_RS19975	type II toxin-antitoxin system RelB/DinJ family antitoxin
modA	molybdate ABC transporter substrate-binding protein
EIH14_RS15685	CHAD domain-containing protein
HXP36_05245	diaminopropionate ammonia-lyase
tatA	Sec-independent protein translocase subunit TatA
HXP36_06050	LysR family transcriptional regulator
EIH13_RS06365	RNA pyrophosphohydrolase
RALGR_RS12285	LysR family transcriptional regulator
RALB5_RS02670	ABC transporter ATP-binding protein
C2I38_RS07020	hypothetical protein
folC	bifunctional tetrahydrofolate synthase/dihydrofolate synthase
EIH13_RS08240	peptidylprolyl isomerase
AQR24_RS18410	DUF1439 domain-containing protein
dacB	D-alanyl-D-alanine carboxypeptidase/D-alanyl-D-alanine-endopeptidase
lipA	lipoyl synthase
RALB5_RS12250	MCE family protein
EIH13_RS15845	cobalt-precorrin-7 (C(5))-methyltransferase
EIH12_RS22025	acetyl-CoA C-acyltransferase family protein
AQR24_RS13700	response regulator transcription factor

Gene	Annotation
UW163_RS21050	cytochrome ubiquinol oxidase subunit I
mprA	MprA protease, GlyGly-CTERM protein-sorting domain-containing form
AQR24_RS20285	glycoside hydrolase
AQR24_RS16445	helix-turn-helix transcriptional regulator
RSMK_RS19525	ATP-binding cassette domain-containing protein
mqo	malate dehydrogenase (quinone)
EIH09_RS17695	anion transporter
EIH13_RS13185	aminotransferase class III-fold pyridoxal phosphate-dependent enzyme
acnD	Fe/S-dependent 2-methylisocitrate dehydratase AcnD
UW163_RS08610	hypothetical protein
AQR21_RS02995	ABC transporter substrate-binding protein
RSMK_RS05775	catalase
rpoD	RNA polymerase sigma factor RpoD
RSMK_RS17685	hypothetical protein
RALGR_RS10810	YnfA family protein
groL	chaperonin GroEL
dsbD	protein-disulfide reductase DsbD
HXP36_22950	hypothetical protein
pgk	phosphoglycerate kinase
EIH14_RS07860	RidA family protein
HXP34_06685	NADH-quinone oxidoreductase subunit G
HXP35_14095	MBL fold metallo-hydrolase
UW163_RS16965	efflux transporter outer membrane subunit
RALGR_RS20495	alpha/beta hydrolase
EIH14_RS14330	acyltransferase family protein
C2I33_RS04700	LuxR family transcriptional regulator
RALB5_RS22835	hypothetical protein
HXP36_03385	ABC transporter ATP-binding protein
HXP35_02840	transposase
C2L97_RS03795	glutathione S-transferase
RALB5_RS13915	YggS family pyridoxal phosphate-dependent enzyme
RALCI_RS02565	hypothetical protein
HXP36_06160	YdcF family protein
AQR24_RS09400	hypothetical protein
AQR21_RS08145	hypothetical protein
EIH14_RS11560	ABC transporter permease
group_497	phasin family protein
RALCI_RS19010	ATP-binding cassette domain-containing protein
AQR21_RS08390	amino acid permease

Gene	Annotation
mnmC	bifunctional tRNA (5-methylaminomethyl-2-thiouridine)(34)-methyltransferase MnmD/FAD-dependent 5-carboxymethylaminomethyl-2-thiouridine(34) oxidoreductase MnmC
HXP36_10450	BolA family transcriptional regulator
EIH13_RS07925	ankyrin repeat domain-containing protein
RALFB_RS21970	HAMP domain-containing protein
HXP37_00160	SDR family NAD(P)-dependent oxidoreductase
RALCI_RS11095	helix-turn-helix transcriptional regulator
EIH11_RS19000	DUF3999 domain-containing protein
EIH09_RS10980	3-deoxy-7-phosphoheptulonate synthase
group_544	molybdopterin-guanine dinucleotide biosynthesis protein B
nudB	dihydroneopterin triphosphate diphosphatase
EIH13_RS04285	hotdog family protein
RALCI_RS19870	YchJ family protein
C2I33_RS20520	nucleotidyltransferase domain-containing protein
EIH09_RS20640	H-NS histone family protein
cheZ	protein phosphatase CheZ
RALW1_RS25855	LysR family transcriptional regulator
HXP35_16745	anaerobic ribonucleoside-triphosphate reductase activating protein
RSMK_RS05275	2-hydroxychromene-2-carboxylate isomerase
RALW1_RS01515	amino acid ABC transporter ATP-binding protein
EIH14_RS04465	transporter substrate-binding domain-containing protein
EIH14_RS03710	NAD(P)-dependent oxidoreductase
EIH10_RS13325	lytic transglycosylase domain-containing protein
EIH12_RS23240	NAD(P)/FAD-dependent oxidoreductase
EIH09_RS21550	carbohydrate kinase
RALFB_RS10790	exopolysaccharide phosphotransferase
RALCI_RS17885	helix-turn-helix domain-containing protein
EIH11_RS03400	ABC transporter ATP-binding protein
EIH12_RS21115	cysteine synthase A
EIH14_RS11925	DMT family transporter
EIH09_RS16815	SDR family NAD(P)-dependent oxidoreductase
dprA	DNA-protecting protein DprA
mrdA	penicillin-binding protein 2
EIH10_RS06930	hypothetical protein
C2I33_RS02540	DUF3617 domain-containing protein
EIH10_RS03030	hypothetical protein
AQR24_RS04955	DUF2272 domain-containing protein
RALW1_RS08050	SET domain-containing protein-lysine N-methyltransferase
C2I38_RS00640	DUF3298 domain-containing protein

Gene	Annotation
EIH11_RS10370	hypothetical protein
RALCI_RS01690	hypothetical protein
RALB5_RS15620	hypothetical protein
msrP	protein-methionine-sulfoxide reductase catalytic subunit MsrP
EIH11_RS20880	shikimate kinase
EIH12_RS10840	uracil-DNA glycosylase
HXP37_07275	aminopeptidase
HXP34_03700	DMT family transporter
EIH12_RS08600	DUF2147 domain-containing protein
pilV	type IV pilus modification protein PilV
C2L97_RS04180	thioredoxin family protein
EIH13_RS05450	GNAT family N-acetyltransferase
EIH13_RS05430	DUF3313 domain-containing protein
EIH14_RS19335	oxidoreductase
AQR21_RS17455	hypothetical protein
RALW1_RS13405	DMT family transporter
cobC	alpha-ribazole phosphatase
EIH12_RS20830	MipA/OmpV family protein
UW163_RS09725	hypothetical protein
uraH	hydroxyisourate hydrolase
HXP37_10540	DUF1330 domain-containing protein
HXP35_06585	membrane protein
RSMK_RS12430	pyridoxal phosphate-dependent aminotransferase
AQR21_RS15585	YdcF family protein
EIH10_RS19850	EAL domain-containing protein
RSMK_RS13735	hypothetical protein
C2L97_RS08510	arylesterase
pdhA	pyruvate dehydrogenase (acetyl-transferring) E1 component subunit alpha
rpmC	50S ribosomal protein L29
minE	cell division topological specificity factor MinE
rplS	50S ribosomal protein L19
RALGR_RS11370	sugar phosphate isomerase/epimerase
AQR24_RS01995	F0F1 ATP synthase subunit epsilon
pilW	type IV pilus biogenesis/stability protein PilW
rpsO	30S ribosomal protein S15
iscU	Fe-S cluster assembly scaffold IscU
rplN	50S ribosomal protein L14
RSPO_RS14710	P-II family nitrogen regulator
UW163_RS22795	hypothetical protein

Gene	Annotation
apaG	Co2+/Mg2+ efflux protein ApaG
C2L97_RS03740	NAD(P) transhydrogenase subunit alpha
EIH14_RS19390	Trm112 family protein
AQR21_RS03335	4a-hydroxytetrahydrobiopterin dehydratase
ccoS	cbb3-type cytochrome oxidase assembly protein CcoS
RALB5_RS05210	YajQ family cyclic di-GMP-binding protein
rpmI	50S ribosomal protein L35
C2I38_RS16435	cold-shock protein
HXP35_12225	co-chaperone GroES
C2L97_RS13205	Flp family type IVb pilin
EIH10_RS16645	RidA family protein
C2L97_RS11700	hypothetical protein
EIH10_RS10780	dihydroneopterin aldolase
rplP	50S ribosomal protein L16
rpsG	30S ribosomal protein S7
RSPO_RS14685	HPr family phosphocarrier protein
glnK	P-II family nitrogen regulator
hfq	RNA chaperone Hfq
ftsA	cell division protein FtsA
RALB5_RS06100	molecular chaperone Tir
RSMK_RS18665	HU family DNA-binding protein
fabI	enoyl-ACP reductase FabI
glnA	type I glutamate--ammonia ligase
C2I33_RS00625	dihydrofolate reductase
minD	septum site-determining protein MinD
rplD	50S ribosomal protein L4
rpsS	30S ribosomal protein S19
mtgA	monofunctional biosynthetic peptidoglycan transglycosylase
rpsT	30S ribosomal protein S20
nusB	transcription antitermination factor NusB
EIH13_RS06690	hypothetical protein
dksA	RNA polymerase-binding protein DksA
rplT	50S ribosomal protein L20
RSMK_RS07560	hypothetical protein
rpmJ	50S ribosomal protein L36
EIH14_RS06860	CoA transferase subunit B
RALB5_RS21610	Lrp/AsnC family transcriptional regulator
rpsI	30S ribosomal protein S9
ybgF	tol-pal system protein YbgF

Gene	Annotation
ompR	two-component system response regulator OmpR
zapA	cell division protein ZapA
rpsM	30S ribosomal protein S13
rplU	50S ribosomal protein L21
rpsH	30S ribosomal protein S8
rpmB	50S ribosomal protein L28
ilvN	acetolactate synthase small subunit
HXP36_05360	DNA starvation/stationary phase protection protein
acpP	acyl carrier protein
mscL	large conductance mechanosensitive channel protein MscL
HXP35_02955	phosphoribosyl-ATP diphosphatase
C2I33_RS12805	GNAT family N-acetyltransferase
rplR	50S ribosomal protein L18
clpP	ATP-dependent Clp endopeptidase proteolytic subunit ClpP
erpA	iron-sulfur cluster insertion protein ErpA
pncB	nicotinate phosphoribosyltransferase
C2I38_RS03525	membrane protein
sctS	type III secretion system export apparatus subunit SctS
group_8192	chemotaxis protein CheY
rplO	50S ribosomal protein L15
group_8194	peptide chain release factor 2
rpmA	50S ribosomal protein L27
RSPO_RS19730	acyloxyacyl hydrolase
HXP35_11360	sulfurtransferase TusA family protein
C2I38_RS14230	polymer-forming cytoskeletal protein
clpS	ATP-dependent Clp protease adapter ClpS
RALFB_RS06500	DUF3460 family protein
rplE	50S ribosomal protein L5
HXP36_01905	hypothetical protein
rpmG	50S ribosomal protein L33
EIH12_RS06720	hypothetical protein
nrdR	transcriptional repressor NrdR
rpmD	50S ribosomal protein L30
ahpC	peroxiredoxin
mreD	rod shape-determining protein MreD
nuoK	NADH-quinone oxidoreductase subunit NuoK
rpoE	RNA polymerase sigma factor RpoE
fur	ferric iron uptake transcriptional regulator
rplM	50S ribosomal protein L13

Gene	Annotation
hrcA	heat-inducible transcriptional repressor HrcA
HXP34_13380	twin transmembrane helix small protein
EIH10_RS15145	branched-chain amino acid transaminase
EIH14_RS01730	sigma-54-dependent Fis family transcriptional regulator
EIH10_RS14910	leucine--tRNA ligase
acnA	aconitate hydratase AcnA
HXP34_10560	DUF1841 family protein
C2I33_RS05085	NADH-quinone oxidoreductase subunit B
HXP37_21825	flagellar synthesis regulator flen protein
hisI	phosphoribosyl-AMP cyclohydrolase
adk	adenylate kinase
fabD	ACP S-malonyltransferase
gpmA	2,3-diphosphoglycerate-dependent phosphoglycerate mutase
phoB	phosphate regulon transcriptional regulatory protein PhoB
RSMK_RS22105	50S ribosomal protein L25/general stress protein Ctc
secF	protein translocase subunit SecF
EIH10_RS08260	flavin reductase family protein
EIH09_RS10920	serine hydroxymethyltransferase
trpB	tryptophan synthase subunit beta
C2L97_RS03270	peroxiredoxin
AQR24_RS19970	type II toxin-antitoxin system YafQ family toxin
trmD	tRNA (guanosine(37)-N1)-methyltransferase TrmD
EIH11_RS04290	CTP synthase
RSPO_RS12970	response regulator
RALFB_RS15920	response regulator
phoU	phosphate signaling complex protein PhoU
gmk	guanylate kinase
EIH10_RS00295	acetyl-CoA carboxylase carboxyltransferase subunit alpha
argJ	bifunctional glutamate N-acetyltransferase/amino-acid acetyltransferase ArgJ
HXP37_04640	branched-chain amino acid ABC transporter permease
C2I33_RS03740	energy transducer TonB
nuoF	NADH-quinone oxidoreductase subunit NuoF
cysD	sulfate adenylyltransferase subunit CysD
RALGR_RS10290	ABC transporter substrate-binding protein
hflC	protease modulator HflC
EIH13_RS03485	Gfo/Idh/MocA family oxidoreductase
AQR21_RS10035	PAS domain-containing sensor histidine kinase
C2I33_RS23645	DNA topoisomerase III
C2I38_RS10875	tryptophan--tRNA ligase

Gene	Annotation
kdsA	3-deoxy-8-phosphooctulonate synthase
AQR24_RS00745	class II aldolase/adducin family protein
RSPO_RS11300	aspartate/tyrosine/aromatic aminotransferase
ttcA	tRNA 2-thiocytidine(32) synthetase TtcA
RALB5_RS04670	hypothetical protein
HXP37_20935	glutaminase
HXP35_04705	superoxide dismutase
HXP36_15975	Glu/Leu/Phe/Val dehydrogenase
EIH12_RS11485	ABC transporter permease
EIH13_RS18250	hypothetical protein
metG	methionine--tRNA ligase
plsY	glycerol-3-phosphate 1-O-acyltransferase PlsY
wrbA	NAD(P)H:quinone oxidoreductase
HXP37_01960	glutamate/aspartate ABC transporter substrate-binding protein
RALCI_RS10145	PLP-dependent aminotransferase family protein
RSMK_RS02330	Do family serine endopeptidase
EIH12_RS14725	sugar ABC transporter permease
argB	acetylglutamate kinase
pxpA	5-oxoprolinase subunit PxpA
ftsL	cell division protein FtsL
RALGR_RS02585	methionine adenosyltransferase
AQR21_RS13535	NADH-quinone oxidoreductase subunit D
C2L97_RS06165	DUF969 domain-containing protein
HXP35_10835	type IIA DNA topoisomerase subunit B
RALFB_RS05570	endonuclease/exonuclease/phosphatase family protein
C2I38_RS00800	UvrD-helicase domain-containing protein
EIH14_RS04830	tryptophan synthase subunit alpha
C2L97_RS04165	RNA-binding S4 domain-containing protein
EIH14_RS07600	heme biosynthesis protein HemY
EIH14_RS13260	DsbA family protein
EIH09_RS09800	glutamate-5-semialdehyde dehydrogenase
C2I38_RS03955	hydrogen peroxide-inducible genes activator
group_8292	UTP--glucose-1-phosphate uridylyltransferase GalU
sucD	succinate--CoA ligase subunit alpha
RALCI_RS08395	PTS sugar transporter subunit IIA
EIH14_RS20490	lytic transglycosylase domain-containing protein
fabH	ketoacyl-ACP synthase III
rpsA	30S ribosomal protein S1
AQR21_RS16005	hypothetical protein

Gene	Annotation
C2I33_RS24495	butyryl-CoA dehydrogenase
RALB5_RS00560	transporter substrate-binding domain-containing protein
C2L97_RS00155	amino acid ABC transporter substrate-binding protein
hisB	imidazoleglycerol-phosphate dehydratase HisB
AQR24_RS24140	DUF1304 domain-containing protein
EIH13_RS03470	sugar transferase
ispH	4-hydroxy-3-methylbut-2-enyl diphosphate reductase
RALFB_RS19020	F0F1 ATP synthase subunit B
EIH11_RS08125	ABC transporter ATP-binding protein
RALW1_RS19600	FMN-binding glutamate synthase family protein
UW163_RS00790	hypothetical protein
eno	phosphopyruvate hydratase
RALB5_RS09315	UDP-N-acetylmuramate--L-alanine ligase
gatB	Asp-tRNA(Asn)/Glu-tRNA(Gln) amidotransferase subunit GatB
HXP34_19210	organic hydroperoxide resistance protein
secG	preprotein translocase subunit SecG
EIH11_RS08150	peptidylprolyl isomerase
RALW1_RS07115	phage holin family protein
RALB5_RS00905	KpsF/GutQ family sugar-phosphate isomerase
tolQ	protein TolQ
leuD	3-isopropylmalate dehydratase small subunit
trmB	tRNA (guanosine(46)-N7)-methyltransferase TrmB
AQR24_RS11495	DUF502 domain-containing protein
RALCI_RS02625	S-(hydroxymethyl)glutathione dehydrogenase/class III alcohol dehydrogenase
gyrB	DNA topoisomerase (ATP-hydrolyzing) subunit B
atpG	F0F1 ATP synthase subunit gamma
RALCI_RS17050	UDP-3-O-acyl-N-acetylglucosamine deacetylase
C2L97_RS05025	DNA polymerase III subunit chi
RALGR_RS18480	membrane protein
rplV	50S ribosomal protein L22
RALGR_RS11765	DNA polymerase III subunit beta
RALCI_RS02255	protein-L-isoaspartate O-methyltransferase
RALFB_RS21040	LysR family transcriptional regulator
RALCI_RS03440	acyl-CoA thioesterase
RSMK_RS17010	LysR family transcriptional regulator
C2I38_RS06415	class 1 fructose-bisphosphatase
secB	protein-export chaperone SecB
purM	phosphoribosylformylglycinamidine cyclo-ligase
AQR21_RS17865	aspartate carbamoyltransferase catalytic subunit

Gene	Annotation
AQR21_RS11830	aspartate kinase
greB	transcription elongation factor GreB
pxpB	5-oxoprolinase subunit PxpB
rpsN	30S ribosomal protein S14
EIH09_RS09415	carbon-nitrogen hydrolase family protein
HXP35_19650	permease
EIH11_RS02175	LacI family transcriptional regulator
lptC	LPS export ABC transporter periplasmic protein LptC
UW163_RS16440	ABC transporter ATP-binding protein
rnc	ribonuclease III
RALGR_RS02325	fumarate hydratase
RSPO_RS16080	D-2-hydroxyacid dehydrogenase family protein
RSMK_RS12065	NADP-dependent malic enzyme
HXP34_13745	5-methyltetrahydrofolate--homocysteine methyltransferase
AQR24_RS02765	methionine ABC transporter ATP-binding protein
leuC	3-isopropylmalate dehydratase large subunit
mraZ	division/cell wall cluster transcriptional repressor MraZ
RSMK_RS09055	2-oxoglutarate dehydrogenase E1 component
EIH12_RS22000	acetyl-CoA C-acetyltransferase
RSMK_RS12530	hypothetical protein
bamC	outer membrane protein assembly factor BamC
ubiB	ubiquinone biosynthesis regulatory protein kinase UbiB
nuoE	NADH-quinone oxidoreductase subunit NuoE
C2I33_RS16310	DUF2863 family protein
petA	ubiquinol-cytochrome c reductase iron-sulfur subunit
HXP34_09920	gamma carbonic anhydrase family protein
EIH09_RS20625	nitric-oxide reductase large subunit
C2I38_RS18740	nitrate reductase subunit alpha
bamD	outer membrane protein assembly factor BamD
lipB	lipoyl(octanoyl) transferase LipB
yajC	preprotein translocase subunit YajC
C2L97_RS06610	acetolactate synthase 3 catalytic subunit
C2I38_RS07980	MFS transporter
rlmB	23S rRNA (guanosine(2251)-2'-O)-methyltransferase RlmB
C2L97_RS03645	glyoxalase/bleomycin resistance/extradiol dioxygenase family protein
nuoH	NADH-quinone oxidoreductase subunit NuoH
HXP34_04565	membrane protein
EIH12_RS13160	ABC transporter ATP-binding protein
glmM	phosphoglucosamine mutase

Gene	Annotation
purU	formyltetrahydrofolate deformylase
accC	acetyl-CoA carboxylase biotin carboxylase subunit
argC	N-acetyl-gamma-glutamyl-phosphate reductase
rpiA	ribose-5-phosphate isomerase RpiA
hisF	imidazole glycerol phosphate synthase subunit HisF
EIH10_RS03270	slipin family protein
C2I38_RS03655	barstar family protein
rfbD	dTDP-4-dehydrorhamnose reductase
RALFB_RS09445	response regulator
ppx	exopolyphosphatase
HXP35_21035	HlyD family type I secretion periplasmic adaptor subunit
EIH13_RS15135	quinone oxidoreductase
ssb	single-stranded DNA-binding protein
HXP36_08840	heme-binding protein
C2L97_RS19630	branched-chain amino acid ABC transporter permease
EIH11_RS08625	pyrimidine/purine nucleoside phosphorylase
rsmH	16S rRNA (cytosine(1402)-N(4))-methyltransferase RsmH
flhA	flagellar biosynthesis protein FlhA
AQR24_RS09215	histidinol-phosphate transaminase
sdhD	succinate dehydrogenase, hydrophobic membrane anchor protein
C2I33_RS15070	response regulator transcription factor
RSMK_RS22555	amino acid ABC transporter permease
HXP37_02470	ANTAR domain-containing response regulator
EIH12_RS05535	ParA family protein
EIH09_RS06070	multifunctional CCA addition/repair protein
wecC	UDP-N-acetyl-D-mannosamine dehydrogenase
EIH14_RS11500	RNA polymerase sigma factor
C2L97_RS03445	ribonucleotide-diphosphate reductase subunit beta
cysM	cysteine synthase CysM
RALW1_RS09335	isovaleryl-CoA dehydrogenase
trxB	thioredoxin-disulfide reductase
surE	5'/3'-nucleotidase SurE
C2I33_RS18825	DUF1488 domain-containing protein
leuB	3-isopropylmalate dehydrogenase
C2L97_RS13490	hypothetical protein
hppD	4-hydroxyphenylpyruvate dioxygenase
der	ribosome biogenesis GTPase Der
RALFB_RS24235	uroporphyrinogen decarboxylase
HXP34_05195	sulfate adenylyltransferase subunit 1

Gene	Annotation
RALCI_RS04380	inositol monophosphatase
aroE	shikimate dehydrogenase
EIH09_RS17005	2-isopropylmalate synthase
carB	carbamoyl-phosphate synthase large subunit
EIH11_RS12515	hypothetical protein
purD	phosphoribosylamine--glycine ligase
EIH10_RS16005	enoyl-CoA hydratase/isomerase family protein
HXP35_14335	GtrA family protein
rfbB	dTDP-glucose 4,6-dehydratase
HXP34_06715	NADH-quinone oxidoreductase subunit M
aspS	aspartate--tRNA ligase
RSMK_RS02445	ABC transporter ATP-binding protein
RALFB_RS08235	rhodanese-like domain-containing protein
C2I33_RS24475	methylcrotonoyl-CoA carboxylase
sucC	ADP-forming succinate--CoA ligase subunit beta
UW163_RS01325	hypothetical protein
HXP36_05880	MotA/TolQ/ExbB proton channel family protein
HXP36_15390	hypothetical protein
RALCI_RS20275	peroxiredoxin
iscR	Fe-S cluster assembly transcriptional regulator IscR
EIH09_RS17130	NUDIX hydrolase
rpoH	RNA polymerase sigma factor RpoH
lptF	LPS export ABC transporter permease LptF
AQR21_RS15550	helix-turn-helix transcriptional regulator
secY	preprotein translocase subunit SecY
HXP37_09355	dCTP deaminase
EIH10_RS02465	NADH-quinone oxidoreductase subunit J
HXP36_12405	CoA transferase
AQR21_RS18040	succinate dehydrogenase flavoprotein subunit
rpsK	30S ribosomal protein S11
RALCI_RS01355	acetoacetate decarboxylase
EIH12_RS14240	lipid carrier protein
HXP35_06020	flavodoxin family protein
UW163_RS14990	response regulator transcription factor
AQR24_RS03415	glutamate 5-kinase
motA	flagellar motor stator protein MotA
UW163_RS06840	iron ABC transporter permease
rpsC	30S ribosomal protein S3
RALW1_RS04215	response regulator transcription factor

Gene	Annotation
group_8474	ferric iron uptake transcriptional regulator
cyoA	ubiquinol oxidase subunit II
C2L97_RS03225	phospho-N-acetylmuramoyl-pentapeptide- transferase
RSPO_RS13410	fructose-bisphosphate aldolase class II
rplL	50S ribosomal protein L7/L12
rbfA	30S ribosome-binding factor RbfA
HXP34_12355	DUF2946 family protein
ychF	redox-regulated ATPase YchF
C2I33_RS20785	C40 family peptidase
rho	transcription termination factor Rho
AQR21_RS12490	response regulator transcription factor
EIH10_RS02420	NADH-quinone oxidoreductase subunit A
C2L97_RS02725	ABC transporter ATP-binding protein
RSPO_RS00590	F0F1 ATP synthase subunit alpha
EIH10_RS18625	TerC family protein
RSMK_RS00115	OmpA family protein
obgE	GTPase ObgE
EIH11_RS12355	nuclear transport factor 2 family protein
glyQ	glycine--tRNA ligase subunit alpha
UW163_RS10385	serine/threonine protein kinase
HXP36_06285	acetylornithine transaminase
lysS	lysine--tRNA ligase
EIH09_RS08660	aminotransferase
HXP37_20380	HAD-IA family hydrolase
RSPO_RS03615	CopD family protein
serS	serine--tRNA ligase
C2I38_RS14155	FAD-dependent oxidoreductase
zwf	glucose-6-phosphate dehydrogenase
AQR24_RS24720	hypothetical protein
UW163_RS02800	ferredoxin
EIH14_RS02470	acetyl-CoA C-acyltransferase
hslV	ATP-dependent protease subunit HslV
HXP35_06175	ABC transporter permease
EIH12_RS16070	LysR family transcriptional regulator
ubiE	bifunctional demethylmenaquinone methyltransferase/2-methoxy-6-polyprenyl-1,4-benzoquinol methylase UbiE
rng	ribonuclease G
C2I33_RS02550	response regulator transcription factor
sctR	EscR/YscR/HrcR family type III secretion system export apparatus protein
ilvC	ketol-acid reductoisomerase

Gene	Annotation
ptsN	PTS IIA-like nitrogen regulatory protein PtsN
ribD	bifunctional diaminohydroxyphosphoribosylaminopyrimidine deaminase/5-amino-6-(5-phosphoribosylamino)uracil reductase RibD
rplQ	50S ribosomal protein L17
dnaK	molecular chaperone DnaK
RSMK_RS19720	MetQ/NlpA family ABC transporter substrate-binding protein
raiA	ribosome-associated translation inhibitor RaiA
argF	ornithine carbamoyltransferase
pheS	phenylalanine--tRNA ligase subunit alpha
C2I38_RS02135	carbohydrate ABC transporter substrate-binding protein
rplX	50S ribosomal protein L24
EIH12_RS04810	response regulator
RALGR_RS03975	membrane protein
C2I38_RS00610	F0F1 ATP synthase subunit delta
AQR21_RS10250	substrate-binding domain-containing protein
UW163_RS08420	tetratricopeptide repeat protein
EIH10_RS10465	cytochrome c oxidase subunit 3
rpsE	30S ribosomal protein S5
asd	aspartate-semialdehyde dehydrogenase
gyrA	DNA gyrase subunit A
atpD	F0F1 ATP synthase subunit beta
glmU	UDP-N-acetylglucosamine diphosphorylase/glucosamine-1-phosphate N-acetyltransferase
flhD	flagellar transcriptional regulator FlhD
rlmH	23S rRNA (pseudouridine(1915)-N(3))-methyltransferase RlmH
can	carbonate dehydratase
C2I33_RS19570	succinate dehydrogenase iron-sulfur subunit
RSPO_RS06965	acetyl-CoA carboxylase carboxyltransferase subunit beta
bamE	outer membrane protein assembly factor BamE
EIH12_RS12915	segregation/condensation protein A
rfbA	glucose-1-phosphate thymidylyltransferase RfbA
upp	uracil phosphoribosyltransferase
purB	adenylosuccinate lyase
UW163_RS08900	hybrid sensor histidine kinase/response regulator
HXP37_09070	CysB family HTH-type transcriptional regulator
C2L97_RS02655	ATP phosphoribosyltransferase
HXP35_13940	sensor histidine kinase
C2L97_RS13130	YqgE/AlgH family protein
prs	ribose-phosphate pyrophosphokinase
HXP36_24705	AMP nucleosidase
adhP	alcohol dehydrogenase AdhP

Gene	Annotation
HXP37_07050	hypothetical protein
EIH11_RS21885	YbeD family protein
trpC	indole-3-glycerol phosphate synthase TrpC
aceE	pyruvate dehydrogenase (acetyl-transferring), homodimeric type
HXP34_02205	iron permease
ftsW	putative lipid II flippase FtsW
EIH13_RS14010	L-serine ammonia-lyase
lpxO	lipid A hydroxylase LpxO
dnaE	DNA polymerase III subunit alpha
C2I33_RS12475	DUF1488 domain-containing protein
C2I33_RS05815	branched-chain amino acid ABC transporter permease
HXP34_05110	ABC transporter ATP-binding protein
EIH10_RS06155	malate dehydrogenase
ffh	signal recognition particle protein
gltK	glutamate/aspartate ABC transporter permease GltK
RSPO_RS04030	NAD kinase
EIH10_RS07380	U32 family peptidase
EIH12_RS17320	peptidyl-prolyl cis-trans isomerase
RALB5_RS02545	FKBP-type peptidyl-prolyl cis-trans isomerase
iscA	iron-sulfur cluster assembly protein IscA
purH	bifunctional phosphoribosylaminoimidazolecarboxamide formyltransferase/IMP cyclohydrolase
gltA	citrate (Si)-synthase
AQR24_RS22795	rhodanese-like domain-containing protein
ilvA	threonine ammonia-lyase, biosynthetic
gap	type I glyceraldehyde-3-phosphate dehydrogenase
rplW	50S ribosomal protein L23
lon	endopeptidase La
msbA	lipid A export permease/ATP-binding protein MsbA
group_8629	phosphate ABC transporter substrate-binding protein PstS
EIH12_RS22345	hypothetical protein
EIH14_RS19110	LLM class flavin-dependent oxidoreductase
nirK	nitrite reductase, copper-containing
RALW1_RS06680	hypothetical protein
EIH11_RS07465	flavohemoprotein
RSMK_RS00590	hypothetical protein
EIH10_RS13295	bifunctional nicotinamide-nucleotide adenylyltransferase/Nudix hydroxylase
tatB	Sec-independent protein translocase subunit TatB
atpE	F0F1 ATP synthase subunit C
AQR24_RS06990	Smr/MutS family protein

Gene	Annotation
HXP35_14045	dienelactone hydrolase family protein
RSPO_RS04270	patatin-like phospholipase family protein
greA	transcription elongation factor GreA
RALCI_RS04455	GTP cyclohydrolase I FolE2
RALFB_RS18490	LysR family transcriptional regulator
RSPO_RS12515	acyl-CoA dehydrogenase
HXP36_22670	membrane protein
RALGR_RS04070	DUF971 domain-containing protein
trxA	thioredoxin
EIH12_RS22080	HAMP domain-containing protein
C2L97_RS15330	acetyl/propionyl/methylcrotonyl-CoA carboxylase subunit alpha
RALW1_RS21100	DUF3079 domain-containing protein
cobO	cob(I)yrinic acid a,c-diamide adenosyltransferase
HXP34_15430	bb3-type cytochrome oxidase subunit IV
AQR24_RS02085	DUF3820 family protein
C2I33_RS17670	MoxR family ATPase
EIH10_RS06050	CvpA family protein
ppk1	polyphosphate kinase 1
EIH10_RS06590	MarR family transcriptional regulator
tsaD	tRNA (adenosine(37)-N6)-threonylcarbamoyltransferase complex transferase subunit TsaD
EIH11_RS18185	SIMPL domain-containing protein
gatA	Asp-tRNA(Asn)/Glu-tRNA(Gln) amidotransferase subunit GatA
C2I33_RS19485	urocanate hydratase
aroB	3-dehydroquinate synthase
AQR24_RS07050	IclR family transcriptional regulator
RALGR_RS14220	Fis family transcriptional regulator
EIH09_RS08975	GntP family permease
ilvD	dihydroxy-acid dehydratase
AQR21_RS08290	PAS domain-containing protein
EIH10_RS05745	hypothetical protein
HXP35_04655	bile acid:sodium symporter
RALW1_RS12170	polyprenyl synthetase family protein
mpl	UDP-N-acetylmuramate:L-alanyl-gamma-D-glutamyl- meso-diaminopimelate ligase
prpB	methylisocitrate lyase
RSPO_RS06990	ABC transporter permease
serB	phosphoserine phosphatase SerB
EIH11_RS05415	toxin-activating lysine-acyltransferase
EIH12_RS20910	DUF427 domain-containing protein
RALCI_RS05475	nitrite/sulfite reductase

Gene	Annotation
ugpA	sn-glycerol-3-phosphate ABC transporter permease UgpA
rpsQ	30S ribosomal protein S17
C2I38_RS15060	O-acetyl-ADP-ribose deacetylase
RALW1_RS15005	sugar ABC transporter substrate-binding protein
HXP36_00295	LysR family transcriptional regulator
RSPO_RS07815	CoA transferase subunit A
EIH10_RS10555	3-hydroxybutyryl-CoA dehydrogenase
EIH11_RS08205	phosphoribosylglycinamide formyltransferase
C2I33_RS09075	bifunctional (p)ppGpp synthetase/guanosine-3',5'-bis(diphosphate) 3'-pyrophosphohydrolase
dut	dUTP diphosphatase
HXP37_18215	ATP-grasp domain-containing protein
C2I38_RS11380	RluA family pseudouridine synthase
EIH10_RS17855	branched-chain amino acid ABC transporter permease
UW163_RS18040	ABC transporter ATP-binding protein
UW163_RS03230	helix-turn-helix transcriptional regulator
RALB5_RS09755	BolA family transcriptional regulator
C2I38_RS03320	ATP-binding protein
HXP34_19005	PTS sucrose transporter subunit IIBC
EIH09_RS14040	TetR/AcrR family transcriptional regulator
RSPO_RS19510	TetR family transcriptional regulator
AQR24_RS12295	membrane protein
murA	UDP-N-acetylglucosamine 1-carboxyvinyltransferase
dctA	dicarboxylate/amino acid:cation symporter
EIH12_RS00900	ABC transporter ATP-binding protein
EIH11_RS22870	acetyl-CoA C-acyltransferase
RALCI_RS14670	CoA-binding protein
RALFB_RS04290	efflux RND transporter permease subunit
HXP37_12870	oxidative damage protection protein
metF	methylenetetrahydrofolate reductase [NAD(P)H]
RSMK_RS11055	NAD+ synthase
HXP36_21005	polysaccharide biosynthesis protein
efp	elongation factor P
RALGR_RS11690	long-chain fatty acid--CoA ligase
EIH10_RS01850	ZIP family metal transporter
AQR21_RS19245	hypothetical protein
pal	peptidoglycan-associated lipoprotein Pal
UW163_RS02905	patatin-like phospholipase family protein
lepB	signal peptidase I
C2I38_RS00435	Lrp/AsnC family transcriptional regulator

Gene	Annotation
RSMK_RS01120	hypothetical protein
RALCI_RS06615	DUF3567 domain-containing protein
rimP	ribosome maturation factor RimP
metI	ABC transporter permease
RALFB_RS08245	S41 family peptidase
RALCI_RS09355	arginine/lysine/ornithine decarboxylase
lolA	outer membrane lipoprotein chaperone LolA
ppa	inorganic diphosphatase
RALB5_RS00910	HAD family hydrolase
RSPO_RS02945	aminodeoxychorismate/anthranilate synthase component II
C2L97_RS10905	DUF1330 domain-containing protein
glxR	2-hydroxy-3-oxopropionate reductase
RALW1_RS15245	HU family DNA-binding protein
RSMK_RS18540	DoxX family protein
EIH11_RS10635	integration host factor subunit beta
EIH09_RS04505	DUF465 domain-containing protein
uvrA	excinuclease ABC subunit UvrA
ccoP	cytochrome-c oxidase, cbb3-type subunit III
AQR21_RS01170	cold-shock protein
ftsZ	cell division protein FtsZ
dnaJ	molecular chaperone DnaJ
UW163_RS03475	prohibitin family protein
RALW1_RS06590	DUF2970 domain-containing protein
EIH10_RS09850	class II glutamine amidotransferase
EIH14_RS08115	replication protein RepA
AQR24_RS17030	aminoacyl-tRNA deacylase
AQR24_RS24470	muropeptide transporter
fnr	fumarate/nitrate reduction transcriptional regulator Fnr
EIH11_RS19310	ABC transporter substrate-binding protein
group_8753	translation initiation factor IF-1
ruvB	Holliday junction branch migration DNA helicase RuvB
EIH12_RS19360	3-hydroxybutyrate dehydrogenase
AQR21_RS12140	H-NS histone family protein
hutC	histidine utilization repressor
yejB	microcin C ABC transporter permease YejB
galE	UDP-glucose 4-epimerase GalE
AQR24_RS24460	EamA family transporter
C2I33_RS23160	sodium:solute symporter family protein
EIH09_RS16945	glycosyltransferase family 1 protein

Gene	Annotation
EIH10_RS05070	cystathionine beta-lyase
EIH13_RS05775	type IV pilus twitching motility protein PilT
ispE	4-(cytidine 5'-diphospho)-2-C-methyl-D-erythritol kinase
lysA	diaminopimelate decarboxylase
AQR21_RS17905	sugar ABC transporter
RALFB_RS09885	TetR/AcrR family transcriptional regulator
RALCI_RS21180	DUF1488 domain-containing protein
AQR21_RS03455	hypothetical protein
waaF	lipopolysaccharide heptosyltransferase II
EIH14_RS20150	response regulator
EIH09_RS12680	dodecin family protein
C2I38_RS10355	Gfo/Idh/MocA family oxidoreductase
UW163_RS04515	ABC transporter permease subunit
RALW1_RS00325	hypoxanthine-guanine phosphoribosyltransferase
C2I38_RS22815	aspartate aminotransferase family protein
HXP34_13470	bacterioferritin-associated ferredoxin
C2L97_RS15910	adenosylhomocysteinase
EIH12_RS05765	ATP-binding cassette domain-containing protein
RALW1_RS06765	DNA-binding transcriptional regulator
RSMK_RS13755	leucyl/phenylalanyl-tRNA--protein transferase
tkt	transketolase
EIH13_RS04275	beta-ketoacyl-[acyl-carrier-protein] synthase family protein
C2I33_RS14955	NAD(P)-dependent glycerol-3-phosphate dehydrogenase
pdxJ	pyridoxine 5'-phosphate synthase
pstS	phosphate ABC transporter substrate-binding protein PstS
HXP35_12430	lipase
RALFB_RS24945	LemA family protein
RALB5_RS23555	NAD(P)/FAD-dependent oxidoreductase
HXP36_08020	DNA-directed RNA polymerase subunit omega
EIH12_RS16415	GNAT family N-acetyltransferase
EIH13_RS09875	LysR family transcriptional regulator
iolE	myo-inosose-2 dehydratase
C2I33_RS00530	AsnC family transcriptional regulator
RALFB_RS04100	aromatic ring-hydroxylating dioxygenase subunit alpha
AQR24_RS16415	peptidyl-prolyl cis-trans isomerase
hemB	porphobilinogen synthase
RSMK_RS21965	cytochrome c oxidase assembly protein
C2L97_RS12475	low specificity L-threonine aldolase
RALB5_RS09540	NADPH:quinone oxidoreductase family protein

Gene	Annotation
RALCI_RS05380	GlsB/YeaQ/YmgE family stress response membrane protein
AQR21_RS11350	hypothetical protein
rsgA	ribosome small subunit-dependent GTPase A
EIH14_RS11715	DUF4136 domain-containing protein
HXP36_16755	D-alanyl-D-alanine carboxypeptidase
EIH11_RS11035	malonic semialdehyde reductase
C2L97_RS11355	hypothetical protein
UW163_RS02175	membrane protein
HXP35_21955	carbonic anhydrase
EIH10_RS17210	winged helix-turn-helix transcriptional regulator
EIH09_RS08675	RsmB/NOP family class I SAM-dependent RNA methyltransferase
gspG	type II secretion system major pseudopilin GspG
RALCI_RS10275	CDP-6-deoxy-delta-3,4-glucoseen reductase
ubiD	4-hydroxy-3-polyprenylbenzoate decarboxylase
EIH13_RS00105	biopolymer transporter ExbD
miaB	tRNA (N6-isopentenyl adenosine(37)-C2)-methylthiotransferase MiaB
C2I38_RS02575	pilus assembly protein PilP
feoB	ferrous iron transport protein B
AQR21_RS09975	IclR family transcriptional regulator
pssA	CDP-diacylglycerol--serine O-phosphatidyltransferase
RALGR_RS22400	acyl-CoA thioesterase
RALB5_RS06095	response regulator transcription factor
EIH14_RS06150	winged helix DNA-binding protein
RALCI_RS05850	hypothetical protein
rpsP	30S ribosomal protein S16
recR	recombination protein RecR
EIH09_RS14715	xanthine dehydrogenase family protein subunit M
C2I33_RS07940	hypothetical protein
wecB	UDP-N-acetylglucosamine 2-epimerase (non-hydrolyzing)
pth	aminoacyl-tRNA hydrolase
EIH14_RS06075	porin
RALB5_RS02270	hypothetical protein
EIH11_RS15020	type B 50S ribosomal protein L31
RSMK_RS01325	Lrp/AsnC family transcriptional regulator
AQR21_RS05495	accessory factor UbiK family protein
EIH10_RS17195	helix-turn-helix transcriptional regulator
RALB5_RS20025	SDR family oxidoreductase
zapD	cell division protein ZapD
phoR	phosphate regulon sensor histidine kinase PhoR

Gene	Annotation
EIH12_RS06100	YafY family transcriptional regulator
HXP35_13420	YfhL family 4Fe-4S dicluster ferredoxin
C2I33_RS02795	transcriptional regulator
RALFB_RS21670	DUF192 domain-containing protein
AQR21_RS13235	cytochrome c4
pyrC	dihydroorotase
RALB5_RS18100	transglutaminase family protein
AQR21_RS20120	LysR family transcriptional regulator
C2I38_RS05980	FprA family A-type flavoprotein
nuoI	NADH-quinone oxidoreductase subunit NuoI
EIH14_RS18485	hypothetical protein
ugpE	sn-glycerol-3-phosphate ABC transporter permease UgpE
RALB5_RS02945	ParB/RepB/Spo0J family partition protein
C2I38_RS22415	acyltransferase
EIH11_RS19840	GHKL domain-containing protein
RSMK_RS12285	phosphatidylserine decarboxylase
aceK	bifunctional isocitrate dehydrogenase kinase/phosphatase
RSMK_RS23710	undecaprenyl-diphosphate phosphatase
RALB5_RS09085	aromatic ring-hydroxylating dioxygenase subunit alpha
EIH13_RS19350	response regulator transcription factor
grxC	glutaredoxin 3
UW163_RS16330	DUF2249 domain-containing protein
EIH12_RS14365	carbamoyltransferase
HXP37_00865	symmetrical bis(5'-nucleosyl)-tetraphosphatase
ugpB	sn-glycerol-3-phosphate ABC transporter substrate-binding protein UgpB
RSPO_RS08290	trigger factor
C2I33_RS07650	adenylosuccinate synthase
EIH14_RS20600	DUF721 domain-containing protein
HXP37_12375	4-hydroxy-tetrahydrodipicolinate synthase
RSMK_RS20815	glutathione S-transferase family protein
EIH14_RS06285	peroxiredoxin
tolR	protein TolR
apbC	iron-sulfur cluster carrier protein ApbC
C2L97_RS00420	DUF3717 domain-containing protein
AQR21_RS20675	DegQ family serine endoprotease
RALB5_RS06835	site-specific DNA-methyltransferase
UW163_RS15975	histone deacetylase family protein
EIH10_RS19950	RlmE family RNA methyltransferase
zapE	cell division protein ZapE

Gene	Annotation
pstB	phosphate ABC transporter ATP-binding protein PstB
HXP35_03740	carbohydrate kinase family protein
EIH12_RS06920	glutamate synthase subunit alpha
lysM	peptidoglycan-binding protein LysM
C2I38_RS06390	BcpO-related WXXGXW repeat protein
pilM	pilus assembly protein PilM
RSMK_RS02105	M61 family metallopeptidase
EIH11_RS02485	amino acid ABC transporter permease
EIH13_RS12560	H-NS histone family protein
HXP37_15885	CobD/CbiB family protein
HXP36_17585	RNA polymerase sigma factor
UW163_RS22580	APC family permease
AQR21_RS20950	DegQ family serine endoprotease
rnhA	ribonuclease HI
C2I33_RS23025	four-helix bundle copper-binding protein
C2I33_RS13105	rhodanese-like domain-containing protein
group_8896	methylisocitrate lyase
coq7	2-polyprenyl-3-methyl-6-methoxy-1,4-benzoquinone monooxygenase
C2I38_RS11660	PLP-dependent aminotransferase family protein
EIH10_RS09070	response regulator
RALFB_RS14975	helix-turn-helix domain-containing protein
AQR24_RS02905	EpsG family protein
AQR21_RS05285	antibiotic biosynthesis monooxygenase
RALFB_RS00955	ABC transporter permease
RALFB_RS03890	DUF1800 family protein
HXP34_14395	phage tail protein
C2I33_RS04135	DUF3565 domain-containing protein
AQR21_RS06840	DUF3579 domain-containing protein
bamB	outer membrane protein assembly factor BamB
EIH12_RS07220	3-hydroxyacyl-CoA dehydrogenase/enoyl-CoA hydratase family protein
EIH09_RS01860	threonylcarbamoyl-AMP synthase
rplA	50S ribosomal protein L1
HXP34_18975	TonB-dependent siderophore receptor
UW163_RS03290	patatin-like phospholipase family protein
EIH13_RS02145	GntR family transcriptional regulator
pyrF	orotidine-5'-phosphate decarboxylase
RALGR_RS04270	tyrosine--tRNA ligase
UW163_RS10725	haloacid dehalogenase-like hydrolase
paaK	phenylacetate-CoA oxygenase/reductase subunit PaaK

Gene	Annotation
era	GTPase Era
HXP35_12735	1-acyl-sn-glycerol-3-phosphate acyltransferase
AQR24_RS01830	methionine--tRNA ligase
HXP35_03340	amidohydrolase
UW163_RS10255	DUF979 domain-containing protein
purF	amidophosphoribosyltransferase
RSMK_RS17775	hypothetical protein
EIH12_RS16755	ATP-binding protein
EIH09_RS06330	sigma-70 family RNA polymerase sigma factor
EIH12_RS03255	NADH-quinone oxidoreductase subunit C
sctJ	type III secretion inner membrane ring lipoprotein SctJ
AQR24_RS02140	ABC transporter ATP-binding protein
RALW1_RS06160	enoyl-CoA hydratase
RALGR_RS06790	TraR/DksA family transcriptional regulator
RALB5_RS17800	bifunctional 2-methylcitrate dehydratase/aconitate hydratase
slmA	nucleoid occlusion factor SlmA
C2L97_RS22550	hypothetical protein
AQR21_RS13845	arginyltransferase
folK	2-amino-4-hydroxy-6- hydroxymethyldihydropteridine diphosphokinase
gph	HAD-IA family hydrolase
hscB	Fe-S protein assembly co-chaperone HscB
RSPO_RS23050	MFS transporter
EIH09_RS06965	hypothetical protein
HXP37_18210	proline dehydrogenase
AQR24_RS13515	MFS transporter
UW163_RS03145	hydroxymethylglutaryl-CoA lyase
EIH12_RS02615	YaeQ family protein
EIH10_RS00775	polysaccharide deacetylase family protein
egtB	ergothioneine biosynthesis protein EgtB
C2I38_RS15130	hypothetical protein
AQR21_RS09595	NAD(P)/FAD-dependent oxidoreductase
AQR24_RS11945	SCO family protein
HXP35_13515	protoheme IX farnesyltransferase
kdpE	two-component system response regulator KdpE
rfbC	dTDP-4-dehydrorhamnose 3,5-epimerase
cheY	chemotaxis protein CheY
EIH12_RS03980	YebC/PmpR family DNA-binding transcriptional regulator
queG	tRNA epoxyqueuosine(34) reductase QueG
RALW1_RS09895	Re/Si-specific NAD(P)(+) transhydrogenase subunit alpha

Gene	Annotation
recO	DNA repair protein RecO
C2L97_RS18625	ribonucleoside triphosphate reductase
RALFB_RS14680	Crp/Fnr family transcriptional regulator
C2L97_RS13475	PLP-dependent aminotransferase family protein
bfr	bacterioferritin
RALCI_RS19885	enoyl-CoA hydratase/isomerase family protein
RALGR_RS09430	LysE family translocator
AQR24_RS05970	3-hydroxybutyrate dehydrogenase
EIH14_RS20480	MarC family protein
HXP37_04310	amino acid ABC transporter ATP-binding protein
C2I33_RS13925	hypothetical protein
C2L97_RS11935	prephenate dehydrogenase
AQR24_RS06035	YihY family inner membrane protein
EIH11_RS05540	porin LamB
EIH14_RS06165	ABC transporter ATP-binding protein
RALFB_RS22975	phosphotransferase
pheA	prephenate dehydratase
HXP34_09670	patatin-like phospholipase family protein
lptB	LPS export ABC transporter ATP-binding protein
RALB5_RS18620	CreA family protein
recN	DNA repair protein RecN
C2I33_RS07550	peptidoglycan DD-metalloendopeptidase family protein
RALB5_RS06880	glycine zipper 2TM domain-containing protein
glpD	glycerol-3-phosphate dehydrogenase
RALGR_RS16790	alkaline phosphatase
RSPO_RS10470	ABC transporter substrate-binding protein
ruvA	Holliday junction branch migration protein RuvA
UW163_RS14430	acetyl-CoA C-acyltransferase
RALFB_RS12635	phenylalanine 4-monooxygenase
HXP37_10065	O-antigen ligase family protein
RALB5_RS12115	prolipoprotein diacylglyceryl transferase
RALB5_RS19675	Lrp/AsnC family transcriptional regulator
RALFB_RS02150	nitronate monooxygenase
RALGR_RS21285	methyltransferase domain-containing protein
RALGR_RS16405	TetR/AcrR family transcriptional regulator
RSPO_RS05605	IclR family transcriptional regulator
HXP34_07900	hypothetical protein
C2L97_RS21670	MFS transporter
EIH12_RS16815	glucan biosynthesis protein

Gene	Annotation
EIH09_RS07910	p-hydroxycinnamoyl CoA hydratase/lyase
hrpB	transcriptional regulator HrpB
prfA	peptide chain release factor 1
gcvA	transcriptional regulator GcvA
HXP37_02530	hypothetical protein
C2L97_RS16785	hypothetical protein
HXP35_10130	alpha-E domain-containing protein
C2I38_RS05700	hypothetical protein
group_9005	acetylornithine deacetylase
UW163_RS07185	enoyl-CoA hydratase
HXP35_19415	LysR family transcriptional regulator ArgP
EIH11_RS06315	hypothetical protein
HXP36_09420	helix-turn-helix transcriptional regulator
C2I33_RS02835	diguanylate cyclase
maf	septum formation inhibitor Maf
EIH10_RS05840	hypothetical protein
yaaA	peroxide stress protein YaaA
EIH10_RS03090	IclR family transcriptional regulator
gudD	glucarate dehydratase
EIH13_RS08135	ABC transporter ATP-binding protein
rpmF	50S ribosomal protein L32
RALW1_RS10970	SDR family oxidoreductase
HXP35_05430	formate dehydrogenase accessory sulfurtransferase FdhD
EIH12_RS14370	hypothetical protein
RALW1_RS17445	HAD-IIIA family hydrolase
gcvH	glycine cleavage system protein GcvH
RALCI_RS04690	response regulator transcription factor
HXP36_15345	HD-GYP domain-containing protein
UW163_RS09080	phosphoadenylyl-sulfate reductase
ccsA	cytochrome c biogenesis protein CcsA
garD	galactarate dehydratase
soxZ	thiosulfate oxidation carrier complex protein SoxZ
UW163_RS09915	TIGR00730 family Rossman fold protein
RALB5_RS17355	enoyl-CoA hydratase family protein
HXP36_18640	alpha/beta hydrolase
EIH10_RS22520	transcriptional regulator
EIH12_RS08895	multidrug efflux MFS transporter
UW163_RS04100	phosphoenolpyruvate carboxykinase (GTP)
EIH12_RS18640	YhbY family RNA-binding protein

Gene	Annotation
RSMK_RS07275	DUF1835 domain-containing protein
pgsA	CDP-diacylglycerol--glycerol-3-phosphate 3-phosphatidyltransferase
gspI	type II secretion system minor pseudopilin GspI
AQR24_RS11610	D-tyrosyl-tRNA(Tyr) deacylase
mutL	DNA mismatch repair endonuclease MutL
EIH10_RS13050	branched-chain amino acid ABC transporter permease
EIH12_RS05230	hypothetical protein
C2I38_RS04475	3-hydroxyacyl-CoA dehydrogenase
paaA	1,2-phenylacetyl-CoA epoxidase subunit A
ccsB	c-type cytochrome biogenesis protein CcsB
C2I33_RS14595	MurR/RpiR family transcriptional regulator
HXP34_02195	tripartite tricarboxylate transporter substrate binding protein
C2I38_RS23770	arginase
EIH11_RS04150	MFS transporter
lepA	elongation factor 4
xth	exodeoxyribonuclease III
HXP35_13820	ABC transporter permease
EIH11_RS19925	DUF937 domain-containing protein
HXP37_07915	polyisoprenoid-binding protein
HXP36_01010	dioxygenase
C2L97_RS13210	precorrin-2 dehydrogenase
serC	3-phosphoserine/phosphohydroxythreonine transaminase
AQR24_RS19910	dTMP kinase
cobI	precorrin-2 C(20)-methyltransferase
RALCI_RS05385	response regulator transcription factor
UW163_RS03720	lytic transglycosylase domain-containing protein
nadA	quinolinate synthase NadA
C2L97_RS03385	proline--tRNA ligase
HXP36_05110	aspartate 1-decarboxylase
RSPO_RS08150	3-ketoacyl-ACP reductase
C2I38_RS12995	riboflavin synthase
nuoL	NADH-quinone oxidoreductase subunit L
rfaE2	D-glycero-beta-D-manno-heptose 1-phosphate adenylyltransferase
rfaE1	D-glycero-beta-D-manno-heptose-7-phosphate kinase
ubiG	bifunctional 2-polyprenyl-6-hydroxyphenol methylase/3-demethylubiquinol 3-O-methyltransferase UbiG
secE	preprotein translocase subunit SecE
RSMK_RS03030	peptidase
pilB	type IV-A pilus assembly ATPase PilB
AQR21_RS13915	putative toxin-antitoxin system toxin component, PIN family

Gene	Annotation
RALCI_RS06235	ABC transporter permease
mdoG	glucan biosynthesis protein G
RALB5_RS03035	ABC transporter ATP-binding protein
EIH11_RS21755	fumarylacetoacetate hydrolase family protein
AQR21_RS04675	methionyl-tRNA formyltransferase
RALGR_RS06210	L-lactate permease
EIH09_RS20435	ABC transporter permease
AQR21_RS19845	sugar ABC transporter permease
HXP37_09695	signal peptidase
UW163_RS15945	acyl-CoA dehydrogenase
pyrE	orotate phosphoribosyltransferase
EIH11_RS07895	GTP-binding protein
RALCI_RS18630	LysR family transcriptional regulator
nusG	transcription termination/antitermination protein NusG
ccoO	cytochrome-c oxidase, cbb3-type subunit II
otsA	alpha,alpha-trehalose-phosphate synthase (UDP-forming)
HXP35_02385	DUF1415 family protein
RALW1_RS12845	cytochrome c
RALB5_RS01055	DUF484 family protein
C2L97_RS13435	methyl-accepting chemotaxis protein
cydB	cytochrome d ubiquinol oxidase subunit II
RALCI_RS04495	dienelactone hydrolase family protein
RSMK_RS11905	hypothetical protein
folD	bifunctional methylenetetrahydrofolate dehydrogenase/methenyltetrahydrofolate cyclohydrolase FolD
mnmG	tRNA uridine-5-carboxymethylaminomethyl(34) synthesis enzyme MnmG
RSPO_RS11490	amino acid ABC transporter substrate-binding protein
EIH12_RS22595	fucose/mannose-binding lectin
RALW1_RS19470	glycosyltransferase family 39 protein
EIH09_RS20445	LysR family transcriptional regulator
RALCI_RS12320	MarR family transcriptional regulator
fdx	ISC system 2Fe-2S type ferredoxin
HXP35_15005	sigma-54-dependent Fis family transcriptional regulator
AQR24_RS21355	ABC transporter permease
UW163_RS15080	phenylalanine--tRNA ligase subunit beta
C2I33_RS13955	MFS transporter
AQR21_RS03705	ABC transporter substrate-binding protein
UW163_RS09055	ABC transporter substrate-binding protein
EIH09_RS11420	glutamate--cysteine ligase
UW163_RS16030	(d)CMP kinase

Gene	Annotation
HXP35_03800	RNB domain-containing ribonuclease
EIH13_RS01765	NarK family nitrate/nitrite MFS transporter
kynU	kynureninase
nodI	nodulation factor ABC transporter ATP-binding protein NodI
HXP35_12480	AzlC family ABC transporter permease
AQR21_RS17470	HD-GYP domain-containing protein
metH	methionine synthase
alaS	alanine--tRNA ligase
EIH12_RS03920	glycosyltransferase family 2 protein
RSPO_RS02665	ABC transporter substrate-binding protein
EIH13_RS00995	5-(carboxyamino)imidazole ribonucleotide synthase
C2I33_RS05825	fatty-acid--CoA ligase
tal	transaldolase
HXP36_21000	hypothetical protein
AQR21_RS23100	ABC transporter substrate-binding protein
RALGR_RS04975	sulfite exporter TauE/SafE family protein
ribB	3,4-dihydroxy-2-butanone-4-phosphate synthase
hemN	oxygen-independent coproporphyrinogen III oxidase
mobA	molybdenum cofactor guanylyltransferase MobA
HXP36_16735	sensor histidine kinase
AQR21_RS03270	AMP-binding protein
RALW1_RS10270	flagellar brake protein
HXP37_03775	thioredoxin family protein
HXP36_08180	adenosine deaminase
cpaB	Flp pilus assembly protein CpaB
puuE	allantoinase PuuE
RALCI_RS22195	YicC family protein
C2I33_RS16145	heavy-metal-associated domain-containing protein
rfaD	ADP-glyceromanno-heptose 6-epimerase
adeC	AdeC/AdeK/OprM family multidrug efflux complex outer membrane factor
RSPO_RS21155	EamA family transporter
EIH09_RS17485	hypothetical protein
HXP35_18095	hypothetical protein
sctC	type III secretion system outer membrane ring subunit SctC
HXP35_18700	rhomboid family intramembrane serine protease
HXP36_21105	type III effector protein
RALB5_RS04425	TonB-dependent receptor
C2I33_RS23715	thioesterase
AQR21_RS12160	DoxX family protein

Gene	Annotation
EIH09_RS03680	prepilin peptidase
EIH09_RS12745	DUF1272 domain-containing protein
EIH12_RS04835	cytochrome-c peroxidase
HXP37_21160	diguanylate cyclase
HXP37_21685	DUF350 domain-containing protein
HXP37_22010	HAMP domain-containing histidine kinase
C2L97_RS19250	N-acetylmuramoyl-L-alanine amidase
psd	phosphatidylserine decarboxylase
group_965	pyroglutamyl-peptidase I
EIH11_RS09140	hypothetical protein
UW163_RS10505	urate hydroxylase PuuD
