# Supplementary material for: Comparative genomics and phylogenomics of the Ralstonia solanacearum Moko ecotype and its symptomatological variants
Source: Genet Mol Biol. 2022 Dec 2;45(4):e20220038. doi: 10.1590/1678-4685-GMB-2022-0038 (PMC9731368; doi:10.1590/1678-4685-GMB-2022-0038)
Supplement: Table S2 - [file 1415-4757-GMB-45-4-e20220038-s2.pdf]

Genetics and Molecular Biology
https://doi.org/10.1590/1678-4685-GMB-2022-0038

Supplementary material to “Comparative genomics and phylogenomics of the *Ralstonia solanacearum* Moko ecotype and its symptomatological variants”

Table S2 - Clusters obtained by pan-genome analysis of the *Ralstonia solanacearum* Moko ecotype and its symptomatological variants.

**Moko typical**	
**Gene**	**Annotation**
RALB5_RS00465	cytochrome P450
RALB5_RS08620	oligopeptide transporter, OPT family
RALB5_RS16855	hypothetical protein
tuf	elongation factor Tu
EIH13_RS16810	response regulator
HXP36_08575	LysR family transcriptional regulator
group_650	4-hydroxythreonine-4-phosphate dehydrogenase PdxA
group_1267	AAA family ATPase
EIH14_RS17925	DUF2875 family protein
EIH10_RS05025	hypothetical protein
RALB5_RS10480	pilus assembly protein
dbpA	ATP-dependent RNA helicase DbpA
RALB5_RS04665	hypothetical protein
AQR24_RS13645	TolC family protein
HXP36_22045	type I secretion system permease/ATPase
RSPO_RS00170	CPBP family intramembrane metalloprotease
HXP37_04345	type III effector protein skwp1
RSPO_RS04395	site-2 protease family protein

Moko typical	
**Gene**	**Annotation**
UW163_RS09520	hypothetical protein
EIH11_RS15320	hypothetical protein
AQR24_RS21055	acetyl-CoA carboxylase
EIH09_RS07790	FimV family protein
HXP35_01625	hypothetical protein
group_4207	leucine efflux protein LeuE
HXP34_13615	hypothetical protein
nac	nitrogen assimilation transcriptional regulator
group_4638	acetyl-CoA carboxylase biotin carboxylase subunit
AQR21_RS20405	biotin-dependent carboxyltransferase family protein
group_4640	5-oxoprolinase subunit PxpA
HXP36_22050	HlyD family efflux transporter periplasmic adaptor subunit
group_5577	5-oxoprolinase subunit PxpB
EIH13_RS00500	H-NS histone family protein
HXP35_01730	ABC transporter substrate-binding protein
HXP34_14725	GNAT family N-acetyltransferase
AQR24_RS23400	PaaI family thioesterase
AQR24_RS21940	hypothetical protein
EIH10_RS21285	DUF4123 domain-containing protein
HXP36_01055	hypothetical protein
EIH10_RS08370	hypothetical protein
EIH09_RS20910	hypothetical protein
HXP37_19860	GNAT family N-acetyltransferase
EIH12_RS23260	alpha/beta fold hydrolase
HXP34_00390	hypothetical protein

Moko typical	
**Gene**	**Annotation**
AQR24_RS12225	hemagglutinin
EIH10_RS04940	serine/threonine protein phosphatase
EIH12_RS03415	hypothetical protein
EIH12_RS05110	hypothetical protein
RALGR_RS13435	PAS domain-containing sensor histidine kinase
HXP34_10640	phytanoyl-CoA dioxygenase
EIH10_RS22635	hypothetical protein
RALB5_RS12340	hypothetical protein
AQR24_RS28080	hypothetical protein
AQR24_RS19815	hypothetical protein
RALGR_RS18855	hypothetical protein
RALB5_RS10810	helix-turn-helix transcriptional regulator
HXP35_18010	hypothetical protein
AQR21_RS20380	amino acid ABC transporter permease
HXP35_18385	SagB/ThcOx family dehydrogenase
HXP35_18400	NADH dehydrogenase
EIH10_RS04085	hypothetical protein
EIH10_RS04135	SDR family oxidoreductase
HXP35_18830	hypothetical protein
AQR21_RS12190	Hpt domain-containing protein
HXP35_19440	DcrB-related protein
EIH12_RS04860	DUF3175 domain-containing protein
AQR24_RS07945	hypothetical protein
HXP36_25035	MerR family DNA-binding protein
EIH12_RS18690	NADH:flavin oxidoreductase/NADH oxidase family protein

Moko typical	
**Gene**	**Annotation**
EIH12_RS00795	IS3 family transposase
EIH09_RS19945	hypothetical protein
EIH09_RS09120	hypothetical protein
HXP37_08670	hypothetical protein
UW163_RS09285	DUF3306 domain-containing protein
EIH11_RS23065	hypothetical protein
EIH13_RS07835	hypothetical protein
HXP37_16370	hypothetical protein
EIH09_RS20985	hypothetical protein
RSPO_RS23305	hypothetical protein
EIH09_RS14585	hypothetical protein
RALW1_RS26045	hypothetical protein
EIH09_RS06975	DUF2894 domain-containing protein
EIH11_RS11330	hypothetical protein
RALW1_RS18410	integrase
RALW1_RS18405	endoribonuclease L-PSP
RALB5_RS10310	acylphosphatase
group_4013	septum site-determining protein MinC
AQR24_RS02080	hypothetical protein
HXP35_02200	porin
EIH10_RS23430	hypothetical protein
EIH12_RS14645	hypothetical protein
EIH12_RS14700	hypothetical protein
AQR21_RS16810	collagen-like triple helix repeat-containing protein
AQR21_RS25705	hypothetical protein

Moko typical	
**Gene**	**Annotation**
EIH10_RS01905	hypothetical protein
AQR24_RS03500	type III effector protein
AQR24_RS14690	S49 family peptidase
HXP34_11300	polyisoprenoid-binding protein
RALGR_RS02190	pilus assembly protein
RALB5_RS14115	hypothetical protein
EIH12_RS12295	hypothetical protein
HXP35_12170	hypothetical protein
AQR21_RS16500	hypothetical protein
HXP34_12035	NAD(P)-binding domain-containing protein
AQR21_RS16480	class I SAM-dependent methyltransferase
EIH12_RS12680	hypothetical protein
AQR21_RS17785	Ku protein
AQR24_RS07990	hypothetical protein
HXP35_14065	type II toxin-antitoxin system Phd/YefM family antitoxin
EIH12_RS22445	type II toxin-antitoxin system RelE/ParE family toxin
HXP34_14055	hypothetical protein
AQR21_RS00850	polyphosphate kinase 2 family protein
group_4431	sulfur carrier protein ThiS
RALB5_RS14985	GALA protein
AQR21_RS08400	hypothetical protein
AQR21_RS11130	hypothetical protein
EIH10_RS17740	helix-turn-helix transcriptional regulator
EIH12_RS19740	hypothetical protein
HXP35_15250	hypothetical protein

Moko typical	
**Gene**	**Annotation**
AQR24_RS06480	DUF4139 domain-containing protein
RALGR_RS18715	flagellar brake protein
group_4585	phenylacetate-CoA oxygenase subunit PaaJ
EIH12_RS04475	hypothetical protein
HXP34_18135	SDR family oxidoreductase
EIH12_RS23275	alpha/beta hydrolase
AQR21_RS09820	CBS domain-containing protein
mgtA	magnesium-translocating P-type ATPase
EIH10_RS13910	bifunctional aminoglycoside phosphotransferase/ATP-binding protein
EIH12_RS23250	bifunctional acetate--CoA ligase family protein/GNAT family N-acetyltransferase
EIH10_RS03980	FtsX-like permease family protein
AQR21_RS09850	ABC transporter ATP-binding protein
EIH10_RS03990	biotin/lipoyl-binding protein
fdhF	formate dehydrogenase subunit alpha
RALB5_RS08475	membrane protein
AQR24_RS14120	YgcG family protein
HXP35_18480	lecithin retinol acyltransferase family protein
HXP34_18325	hypothetical protein
RALGR_RS05205	hypothetical protein
group_4670	ATP-dependent metallopeptidase FtsH/Yme1/Tma family protein
AQR24_RS14160	adenosylcobalamin-dependent ribonucleoside-diphosphate reductase
AQR21_RS11730	cytochrome c4
EIH12_RS07740	hypothetical protein
EIH12_RS07750	1-phosphofructokinase family hexose kinase
AQR21_RS25575	hypothetical protein

Moko typical	
**Gene**	**Annotation**
AQR24_RS18090	CPBP family intramembrane metalloprotease
RALGR_RS13095	collagen-like triple helix repeat-containing protein
HXP34_19635	hypothetical protein
EIH12_RS16100	excisionase family DNA-binding protein
AQR21_RS03210	hypothetical protein
RALB5_RS26085	type III effector (Cyclin-like F-box domain)
HXP34_20145	glycine zipper 2TM domain-containing protein
RALGR_RS12395	hypothetical protein
EIH12_RS19295	hypothetical protein
AQR24_RS19100	hypothetical protein
EIH12_RS19605	poly-beta-1,6-N-acetyl-D-glucosamine biosynthesis protein PgaD
AQR21_RS00270	hypothetical protein
EIH10_RS11125	MFS transporter
AQR21_RS02575	hypothetical protein
HXP35_21250	hypothetical protein
HXP34_21105	hypothetical protein
RALB5_RS18740	disulfide bond formation protein B
AQR21_RS10670	4'-phosphopantetheinyl transferase superfamily protein
EIH13_RS13760	hypothetical protein
EIH14_RS14355	hypothetical protein
UW163_RS05175	TlpA family protein disulfide reductase
RSPO_RS01505	tryptophan 2-monooxygenase oxidoreductase
UW163_RS24510	type III effector
RALW1_RS17655	hypothetical protein
UW163_RS06155	hypothetical protein

Moko typical	
**Gene**	**Annotation**
EIH13_RS16685	hypothetical protein
RSPO_RS02940	DUF1311 domain-containing protein
UW163_RS07595	hemagglutinin
UW163_RS25675	hypothetical protein
EIH13_RS05675	helix-turn-helix transcriptional regulator
EIH11_RS23085	hypothetical protein
EIH09_RS09235	metal-binding protein
EIH11_RS06935	hypothetical protein
RALW1_RS10055	hypothetical protein
EIH11_RS19335	hypothetical protein
EIH13_RS12070	alpha/beta hydrolase
HXP37_13460	type III effector protein
HXP37_13500	GALA protein
EIH09_RS03245	hypothetical protein
UW163_RS14245	GALA protein
group_5902	bifunctional nicotinamidase/pyrazinamidase
HXP37_14865	hypothetical protein
RSPO_RS07640	response regulator transcription factor
EIH11_RS13355	flavodoxin family protein
EIH13_RS07395	universal stress protein
UW163_RS15370	universal stress protein
EIH14_RS07940	GNAT family N-acetyltransferase
UW163_RS16635	hypothetical protein
HXP37_16610	methyltransferase domain-containing protein
EIH09_RS20960	4-oxalocrotonate tautomerase family protein

Moko typical	
**Gene**	**Annotation**
EIH11_RS02105	hypothetical protein
EIH11_RS02125	hypothetical protein
EIH13_RS21840	hypothetical protein
EIH09_RS21075	type I toxin-antitoxin system SymE family toxin
RALW1_RS16740	GNAT family N-acetyltransferase
EIH14_RS08485	ShET2/EspL2 family type III secretion system effector toxin
HXP37_16935	hypothetical protein
RSPO_RS22465	hypothetical protein
RSPO_RS22420	hypothetical protein
RSPO_RS18155	hypothetical protein
RALW1_RS13815	flavoprotein
HXP37_18605	hypothetical protein
UW163_RS19200	hypothetical protein
HXP37_19290	hypothetical protein
EIH13_RS02950	hypothetical protein
EIH13_RS03190	hypothetical protein
RALW1_RS13215	SDR family NAD(P)-dependent oxidoreductase
RALW1_RS00790	membrane protein
EIH14_RS23275	hypothetical protein
HXP37_20270	membrane protein
group_6027	Flp pilus assembly protein CpaB
EIH13_RS12585	collagen-like triple helix repeat-containing protein
EIH09_RS12825	glyoxalase/bleomycin resistance/dioxygenase family protein
RALW1_RS10775	NUDIX hydrolase
UW163_RS20845	hypothetical protein

Moko typical	
**Gene**	**Annotation**
EIH11_RS05065	hypothetical protein
HXP37_21210	hypothetical protein
HXP37_21225	DUF2239 family protein
EIH14_RS00515	ankyrin repeat domain-containing protein
EIH14_RS17825	hypothetical protein
EIH09_RS19730	DUF3005 domain-containing protein
RALW1_RS13080	hypothetical protein
RSPO_RS16875	SRPBCC domain-containing protein
UW163_RS23125	hypothetical protein
RSPO_RS16675	hypothetical protein
RSPO_RS21440	protein popC
EIH12_RS07700	LemA family protein
group_684	membrane protein insertase YidC
HXP35_01600	major capsid protein
RALGR_RS05170	hypothetical protein
group_7040	chromate efflux transporter
RALGR_RS07090	hypothetical protein
RALB5_RS18595	hypothetical protein
EIH10_RS13150	hypothetical protein
RALB5_RS02410	HAD family hydrolase
HXP34_01915	hypothetical protein
RALB5_RS18710	hypothetical protein
EIH12_RS07655	efflux transporter outer membrane subunit
UW163_RS21225	type III effector protein
EIH11_RS08370	hypothetical protein

Moko typical	
**Gene**	**Annotation**
RALW1_RS16340	type III effector protein
HXP34_14780	hypothetical protein
AQR24_RS01505	type II toxin-antitoxin system HipA family toxin
AQR21_RS23295	class I SAM-dependent methyltransferase
AQR24_RS05615	S49 family peptidase
EIH12_RS20455	DUF4440 domain-containing protein
AQR24_RS22680	hypothetical protein
HXP34_07205	HAMP domain-containing protein
ligD	DNA ligase D
RALGR_RS25900	hypothetical protein
RALB5_RS08550	amino acid ABC transporter permease
RALW1_RS11085	HARPIN
EIH12_RS04260	HlyD family secretion protein
RALB5_RS17215	MFS transporter
EIH14_RS04670	TetR/AcrR family transcriptional regulator
AQR21_RS19670	NAD(P) transhydrogenase subunit alpha
EIH12_RS20925	gluconokinase
EIH12_RS20935	RraA family protein
EIH13_RS22505	hypothetical protein
HXP34_10060	hypothetical protein
HXP34_11240	nodulation protein NfeD
RALB5_RS14975	hypothetical protein
AQR21_RS05800	class I SAM-dependent methyltransferase
EIH12_RS00135	hypothetical protein
EIH12_RS00140	hypothetical protein

Moko typical	
**Gene**	**Annotation**
HXP34_15820	3-oxoacyl-ACP synthase
RALGR_RS16275	beta-ketoacyl-ACP synthase
group_4496	3-oxoacyl-ACP reductase FabG
HXP34_15835	beta-hydroxyacyl-ACP dehydratase
HXP35_15995	beta-ketoacyl-[acyl-carrier-protein] synthase family protein
AQR21_RS05845	outer membrane lipoprotein carrier protein LolA
HXP35_16010	acyl-CoA thioesterase
RALGR_RS13710	histidine ammonia-lyase
EIH12_RS00190	glycosyltransferase
AQR24_RS10655	AMP-binding protein
EIH10_RS02000	hypothetical protein
EIH12_RS00205	acyl carrier protein
AQR24_RS10670	hypothetical protein
EIH12_RS00215	1-acyl-sn-glycerol-3-phosphate acyltransferase
AQR24_RS10680	beta-ketoacyl synthase chain length factor
HXP34_16045	BON domain-containing protein
RALB5_RS18340	DUF1801 domain-containing protein
EIH10_RS11700	GALA protein
HXP34_17445	MFS transporter
AQR21_RS20375	amino acid ABC transporter ATP-binding protein
AQR24_RS21025	ABC transporter substrate-binding protein
RALGR_RS13125	type II and III secretion system protein family protein
group_4711	type VI secretion system tip protein VgrG
RALGR_RS20655	LacI family transcriptional regulator
EIH12_RS20450	VOC family protein

Moko typical	
**Gene**	**Annotation**
RALGR_RS13380	NAD(P)(+) transhydrogenase (Re/Si-specific) subunit beta
RALGR_RS13370	NAD(P) transhydrogenase subunit alpha
EIH10_RS21200	glucose 1-dehydrogenase
EIH10_RS21220	aspartate transaminase
HXP37_21025	DUF2145 domain-containing protein
EIH12_RS12490	type III effector protein
EIH12_RS20915	L-idonate 5-dehydrogenase
RALGR_RS25565	helix-turn-helix domain-containing protein
EIH11_RS23075	hypothetical protein
EIH12_RS12085	MBL fold metallo-hydrolase
HXP35_01555	hypothetical protein
AQR24_RS24570	IS110 family transposase
HXP34_08175	hypothetical protein
HXP35_08135	PRTRC system ThiF family protein
EIH10_RS02030	MMPL family transporter
AQR21_RS25645	hypothetical protein
AQR24_RS22955	hypothetical protein
EIH13_RS24455	hypothetical protein
EIH10_RS21210	D-glycerate dehydrogenase
EIH09_RS03900	leucine-rich repeat protein
UW163_RS19820	type III effector protein
EIH10_RS17725	hypothetical protein
EIH12_RS15475	hypothetical protein
RALGR_RS17800	universal stress protein
RALGR_RS17405	hypothetical protein

Moko typical	
**Gene**	**Annotation**
EIH12_RS23085	transposase
AQR24_RS23930	tyrosine-type recombinase/integrase
AQR24_RS23390	type III effector protein
EIH10_RS20300	DEAD/DEAH box helicase
RALGR_RS21750	hypothetical protein
RALGR_RS06935	isoprenylcysteine carboxylmethyltransferase family protein
RALB5_RS14320	integrase arm-type DNA-binding domain-containing protein
EIH14_RS06360	hypothetical protein
HXP34_08315	hypothetical protein
EIH11_RS13990	recombinase family protein
RALGR_RS21705	hypothetical protein
AQR21_RS20590	2Fe-2S iron-sulfur cluster binding domain-containing protein
RALB5_RS13440	Rieske 2Fe-2S domain-containing protein
AQR24_RS20905	salicylate hydroxylase
AQR24_RS20900	non-heme iron oxygenase ferredoxin subunit
group_4317	gentisate 1,2-dioxygenase
RALB5_RS13420	fumarylacetoacetate hydrolase family protein
group_4319	maleylacetoacetate isomerase
AQR24_RS20880	porin
UW163_RS05350	hypothetical protein
HXP34_16050	hypothetical protein
RALB5_RS14745	DNA helicase
AQR24_RS19475	DNA-binding protein
AQR21_RS14945	ATP-binding protein
AQR21_RS14950	S8 family peptidase

Moko typical	
**Gene**	**Annotation**
AQR24_RS19460	DNA-binding protein
EIH10_RS15470	hypothetical protein
EIH10_RS14115	pyridoxamine 5'-phosphate oxidase family protein
EIH10_RS21230	acetyl-CoA carboxylase biotin carboxyl carrier protein subunit
AQR21_RS16395	hypothetical protein
HXP37_05450	hypothetical protein
HXP37_00110	hypothetical protein
EIH13_RS07140	YraN family protein
HXP37_05715	hypothetical protein
UW163_RS06120	hypothetical protein
casB	type I-E CRISPR-associated protein Cse2/CasB
EIH09_RS17225	hypothetical protein
EIH13_RS10935	hypothetical protein
EIH09_RS20655	type III effector gala6 protein
HXP37_17795	hypothetical protein
EIH13_RS15985	hypothetical protein
UW163_RS26350	hypothetical protein
UW163_RS20495	Lrp/AsnC ligand binding domain-containing protein
HXP37_20895	aquaporin family protein
EIH09_RS19400	hypothetical protein
RALW1_RS19230	hypothetical protein
RSPO_RS16745	hypothetical protein
RALW1_RS21700	hypothetical protein
HXP37_23600	hypothetical protein
EIH10_RS07580	hypothetical protein

Moko typical	
**Gene**	**Annotation**
EIH12_RS04270	TolC family protein
EIH12_RS04275	TetR family transcriptional regulator
AQR24_RS13355	PHB depolymerase family esterase
AQR24_RS12785	DUF2235 domain-containing protein
EIH12_RS01085	1,4-beta-cellobiosidase
AQR21_RS04045	Lrp/AsnC family transcriptional regulator
RALB5_RS15105	AzlC family ABC transporter permease
AQR24_RS10175	AzlD family protein
EIH12_RS01105	helix-turn-helix transcriptional regulator
AQR24_RS17735	CBS domain-containing protein
RALB5_RS16525	MgtC/SapB family protein
EIH12_RS12130	universal stress protein
RALGR_RS13450	TraR/DksA family transcriptional regulator
RALB5_RS18695	BON domain-containing protein
EIH10_RS20310	Tn3 family transposase
RALGR_RS26305	hypothetical protein
AQR24_RS21140	Tn3 family transposase
AQR24_RS10445	type III effector protein
EIH12_RS23460	universal stress protein
AQR21_RS13055	universal stress protein
EIH12_RS23470	hypothetical protein
RALB5_RS21660	cytochrome b
EIH10_RS23550	hypothetical protein
tnpB	IS66 family insertion sequence element accessory protein TnpB
AQR24_RS21575	DUF1488 domain-containing protein

Moko typical	
**Gene**	**Annotation**
EIH12_RS12070	thymidine phosphorylase family protein
AQR21_RS20365	DNA-binding protein
AQR21_RS20350	hypothetical protein
AQR24_RS21590	Hsp20 family protein
RALGR_RS14700	nucleoside hydrolase
AQR21_RS06575	hypothetical protein
EIH12_RS12105	helix-turn-helix domain-containing protein
AQR24_RS12810	beta-propeller fold lactonase family protein
AQR24_RS18450	hypothetical protein
AQR24_RS21135	tyrosine-type recombinase/integrase
group_92	2-methylcitrate synthase
AQR21_RS20780	DUF2924 domain-containing protein
AQR21_RS14385	SDR family NAD(P)-dependent oxidoreductase
EIH11_RS19450	phage baseplate assembly protein V
EIH10_RS20525	type III effector
group_1192	glutathione-regulated potassium-efflux system protein KefC
group_1363	chorismate synthase
C2I33_RS09715	EAL domain-containing protein
HXP36_01390	DEAD/DEAH box helicase family protein
C2I33_RS08415	Type III effector protein (Skwp5)
AQR21_RS01605	fatty acyl-AMP ligase
EIH12_RS22125	hypothetical protein
AQR24_RS17880	SDR family NAD(P)-dependent oxidoreductase
EIH12_RS15450	type I polyketide synthase
RALGR_RS25420	DUF1911 domain-containing protein

Moko typical	
**Gene**	**Annotation**
AQR24_RS23210	hypothetical protein
HXP36_02345	hypothetical protein
RALB5_RS24460	helix-turn-helix transcriptional regulator
RALB5_RS22420	hypothetical protein
HXP36_07930	tail assembly protein
HXP34_08565	AlpA family transcriptional regulator
AQR24_RS02355	hypothetical protein
HXP35_22060	hypothetical protein
AQR24_RS25050	type III effector protein
HXP34_17605	conjugal transfer protein
AQR24_RS05130	PAAR domain-containing protein
HXP36_24735	acyl-CoA dehydrogenase
RALGR_RS24370	HAD-IIIC family phosphatase
EIH10_RS21670	thioesterase
AQR21_RS00295	glycoside hydrolase family 3 protein
EIH12_RS12480	hypothetical protein
AQR21_RS23485	hypothetical protein
RALB5_RS27770	hypothetical protein
AQR21_RS04070	cytochrome c
EIH12_RS12230	GALA protein
RALGR_RS21005	hypothetical protein
EIH10_RS16890	IS66 family transposase
HXP36_01215	hypothetical protein
HXP35_01780	tetratricopeptide repeat protein
HXP34_08540	toprim domain-containing protein

Moko typical	
**Gene**	**Annotation**
HXP36_07970	hypothetical protein
HXP36_07965	phosphoribosyltransferase
HXP34_10085	aromatic acid/H+ symport family MFS transporter
AQR24_RS20920	LysR family transcriptional regulator
RALB5_RS22245	LacI family transcriptional regulator
EIH10_RS04005	hypothetical protein
HXP34_18415	hypothetical protein
HXP35_18585	DUF29 domain-containing protein
RALB5_RS01900	hypothetical protein
HXP35_18605	DUF2523 domain-containing protein
AQR24_RS18130	phage coat protein
HXP34_18455	hypothetical protein
RALB5_RS01925	hypothetical protein
HXP36_20865	single-stranded DNA-binding protein
AQR24_RS18150	replication protein
AQR21_RS23550	hypothetical protein
RALGR_RS20650	recombinase family protein
EIH13_RS24405	hypothetical protein
EIH09_RS24000	IS1595 family transposase
EIH13_RS24305	hypothetical protein
HXP37_18895	type III effector gala6 protein
EIH09_RS05280	SDR family NAD(P)-dependent oxidoreductase
UW163_RS25260	hypothetical protein
RSPO_RS17805	Type III effector protein (Skwp 4)
AQR21_RS09085	type III effector protein

Moko typical	
**Gene**	**Annotation**
HXP35_18595	hypothetical protein
AQR24_RS02350	helix-turn-helix transcriptional regulator
group_6769	ribose-phosphate pyrophosphokinase
RALGR_RS24710	IS5 family transposase
group_1031	DUF2156 domain-containing protein
RALB5_RS19545	amino acid adenylation domain-containing protein
EIH12_RS22725	DUF1983 domain-containing protein
RALW1_RS23895	hypothetical protein
AQR21_RS23110	hypothetical protein
EIH10_RS20905	BON domain-containing protein
AQR24_RS23425	ATP-binding protein
RALB5_RS22975	MMPL family transporter
AQR21_RS03860	phage virion morphogenesis protein
RALGR_RS26010	BapA prefix-like domain-containing protein
EIH12_RS15400	hypothetical protein
AQR21_RS23470	hypothetical protein
HXP35_01120	hypothetical protein
AQR24_RS26855	hypothetical protein
RALFB_RS05685	WGR domain-containing protein
RALB5_RS14735	MFS transporter
HXP35_21565	Bcr/CflA family efflux MFS transporter
RALB5_RS06985	integrase arm-type DNA-binding domain-containing protein
HXP34_16810	PAAR domain-containing protein
AQR24_RS21500	ogr/Delta-like zinc finger family protein
AQR24_RS27915	saccharopine dehydrogenase NADP-binding domain-containing protein

Moko typical	
**Gene**	**Annotation**
HXP34_20015	DUF4178 domain-containing protein
AQR21_RS26015	porin
AQR24_RS04390	tyrosine-type recombinase/integrase
RALB5_RS22005	TrfA
HXP35_16405	ATP-dependent helicase
HXP34_16625	hypothetical protein
RALB5_RS27430	BON domain-containing protein
AQR24_RS11400	hypothetical protein
HXP34_21280	hypothetical protein
HXP34_21790	AIPR family protein
HXP35_21995	DUF3274 domain-containing protein
HXP36_14165	hypothetical protein
HXP36_24745	hypothetical protein
EIH13_RS06900	hypothetical protein
RSPO_RS23775	hypothetical protein
AQR24_RS02340	hypothetical protein
RALB5_RS14425	hypothetical protein
EIH10_RS21275	DUF3304 domain-containing protein
RALGR_RS25800	hypothetical protein
AQR21_RS02585	DUF1311 domain-containing protein
AQR21_RS21345	hypothetical protein
EIH10_RS21410	tyrosine-type recombinase/integrase
AQR21_RS03795	hypothetical protein
AQR21_RS03845	phage head protein
AQR21_RS03855	hypothetical protein

Moko typical	
**Gene**	**Annotation**
AQR21_RS03870	DUF935 domain-containing protein
AQR21_RS18650	hypothetical protein
AQR21_RS24870	transcriptional regulator
EIH12_RS02705	hypothetical protein
AQR21_RS25770	hypothetical protein
EIH12_RS13310	hypothetical protein
RALGR_RS26025	hypothetical protein
AQR21_RS15280	hypothetical protein
RALGR_RS24885	HNH endonuclease
RALGR_RS26035	hypothetical protein
AQR21_RS24875	AAA family ATPase
EIH10_RS19675	aspartate/glutamate racemase family protein
RALGR_RS24390	DUF1311 domain-containing protein
EIH12_RS19565	hypothetical protein
EIH10_RS23470	hypothetical protein
AQR21_RS17795	hypothetical protein
EIH10_RS19480	filamentous hemagglutinin N-terminal domain-containing protein
EIH10_RS15835	hypothetical protein
EIH10_RS03110	hypothetical protein
EIH10_RS11425	DUF4303 domain-containing protein
EIH10_RS21720	hypothetical protein
EIH12_RS22120	hypothetical protein
AQR24_RS25400	baseplate assembly protein
EIH13_RS08835	hypothetical protein
RALB5_RS01085	GTP-binding protein

Moko typical	
**Gene**	**Annotation**
creD	cell envelope integrity protein CreD
creC	two-component system sensor histidine kinase CreC
creB	two-component system response regulator CreB
HXP34_00385	hypothetical protein
RALB5_RS25765	alginate lyase family protein
HXP35_01405	hypothetical protein
HXP34_01405	sce7726 family protein
HXP35_01415	TIGR04255 family protein
HXP34_01415	hypothetical protein
AQR24_RS28130	hypothetical protein
AQR24_RS13865	hypothetical protein
HXP34_01430	site-specific recombinase resolvase
HXP34_01530	hypothetical protein
HXP34_01535	hypothetical protein
AQR24_RS05660	hypothetical protein
HXP35_01685	hypothetical protein
RALB5_RS24465	DUF1484 domain-containing protein
HXP34_01880	hypothetical protein
HXP35_01895	tyrosinase family protein
HXP34_02030	hypothetical protein
RALB5_RS26170	hypothetical protein
HXP35_03010	porin
HXP35_03895	hypothetical protein
group_4116	glycolate oxidase subunit GlcF
HXP35_04910	hypothetical protein

Moko typical	
**Gene**	**Annotation**
HXP34_04930	DUF3631 domain-containing protein
HXP35_04925	hypothetical protein
AQR24_RS08565	hypothetical protein
AQR24_RS13295	hypothetical protein
group_4141	type I-E CRISPR-associated protein Cse2/CasB
RALB5_RS05625	recombinase family protein
AQR24_RS27985	hypothetical protein
HXP35_05645	zonular occludens toxin
RALB5_RS05640	DUF2523 domain-containing protein
AQR24_RS05490	coat protein
HXP35_05660	phage coat protein
AQR24_RS05475	hypothetical protein
RALB5_RS05665	hypothetical protein
HXP35_05675	hypothetical protein
RALB5_RS14325	AlpA family phage regulatory protein
RALB5_RS22575	hypothetical protein
HXP35_07205	hypothetical protein
HXP34_07245	hypothetical protein
RALB5_RS22560	hypothetical protein
AQR24_RS21510	helix-turn-helix transcriptional regulator
HXP34_07270	hypothetical protein
dcm	DNA cytosine methyltransferase
HXP35_07245	Com family DNA-binding transcriptional regulator
RALB5_RS09935	hypothetical protein
RALB5_RS02830	hypothetical protein

Moko typical	
**Gene**	**Annotation**
HXP34_07725	hypothetical protein
AQR24_RS11270	DUF4433 domain-containing protein
HXP35_08120	macro domain-containing protein
HXP34_08170	hypothetical protein
RALB5_RS08285	hypothetical protein
HXP34_08245	hypothetical protein
AQR24_RS25675	hypothetical protein
AQR24_RS27520	hypothetical protein
HXP34_08435	DUF3596 domain-containing protein
AQR24_RS24970	ogr/Delta-like zinc finger family protein
HXP35_22035	hypothetical protein
HXP35_22040	hypothetical protein
RALB5_RS27370	hypothetical protein
AQR24_RS14815	tyrosine-type recombinase/integrase
HXP35_10190	C40 family peptidase
RALB5_RS00650	DUF3828 domain-containing protein
EIH10_RS23490	hypothetical protein
RALB5_RS22250	hypothetical protein
HXP35_11185	recombinase family protein
RALB5_RS22230	hypothetical protein
RALB5_RS20520	hypothetical protein
HXP34_11705	carbon-nitrogen hydrolase family protein
RALB5_RS27635	hypothetical protein
AQR24_RS11800	phytanoyl-CoA dioxygenase
RALB5_RS18910	type III effector

Moko typical	
**Gene**	**Annotation**
HXP34_12370	O-antigen ligase family protein
RALB5_RS09360	pilin
AQR24_RS08225	pilin
HXP35_12935	tartrate dehydrogenase
HXP34_13980	type III effector
HXP35_14200	hypothetical protein
HXP35_14260	hypothetical protein
RALB5_RS12355	hypothetical protein
AQR24_RS15520	hypothetical protein
AQR24_RS21690	CbrC family protein
AQR24_RS21695	DUF2199 domain-containing protein
HXP34_14715	hypothetical protein
HXP34_15080	copper resistance protein
RALB5_RS23035	nuclear transport factor 2 family protein
RALB5_RS27380	hypothetical protein
RALB5_RS25760	hypothetical protein
AQR24_RS19445	hypothetical protein
HXP35_16390	AAA family ATPase
HXP34_16250	ParB/RepB/Spo0J family partition protein
RALB5_RS12975	hypothetical protein
RALB5_RS12965	hypothetical protein
AQR24_RS22265	TraI domain-containing protein
HXP35_16445	type IV secretion system protein
RALB5_RS12935	conjugal transfer protein
RALB5_RS12930	conjugal transfer protein

Moko typical	
**Gene**	**Annotation**
HXP34_16320	hypothetical protein
AQR24_RS22295	CpaF family protein
AQR24_RS22300	type IV secretion system protein
HXP34_16335	TrbG/VirB9 family P-type conjugative transfer protein
RALB5_RS12905	TrbI/VirB10 family protein
HXP34_16345	lytic transglycosylase
HXP35_16495	PilL-like protein
AQR24_RS22325	lytic transglycosylase domain-containing protein
HXP34_16360	pilus assembly protein
group_4559	type 4b pilus protein PilO2
group_4560	Flp pilus assembly complex ATPase component TadA
RALB5_RS22480	type II secretion system protein
AQR24_RS20850	prepilin type IV pili
HXP34_16395	hypothetical protein
group_4564	shufflon system plasmid conjugative transfer pilus tip adhesin PilV
AQR24_RS20835	CAP domain-containing protein
HXP34_16410	hypothetical protein
RALB5_RS26720	hypothetical protein
AQR24_RS20825	hypothetical protein
HXP35_16985	hypothetical protein
RALB5_RS07160	hypothetical protein
RALB5_RS26770	PAAR domain-containing protein
AQR24_RS20055	hypothetical protein
HXP35_17095	hypothetical protein
AQR24_RS10220	DUF4123 domain-containing protein

Moko typical	
**Gene**	**Annotation**
AQR24_RS23320	hypothetical protein
AQR24_RS16870	type III effector protein, AvrPphF family
RALB5_RS17480	hypothetical protein
RALB5_RS17500	hypothetical protein
AQR24_RS21020	hypothetical protein
polX	DNA polymerase/3'-5' exonuclease PolX
RALB5_RS16995	phosphoribosyltransferase
RALB5_RS17000	hypothetical protein
HXP34_18270	hypothetical protein
AQR24_RS14090	acyl carrier protein
AQR24_RS14095	2-oxo acid dehydrogenase subunit E2
AQR24_RS14100	alpha-ketoacid dehydrogenase subunit beta
group_4663	pyruvate dehydrogenase (acetyl-transferring) E1 component subunit alpha
acsA	acetate--CoA ligase
HXP35_18610	hypothetical protein
AQR24_RS18155	hypothetical protein
RALB5_RS16845	DUF2946 domain-containing protein
HXP34_18795	hypothetical protein
HXP34_19020	Lrp/AsnC ligand binding domain-containing protein
RALB5_RS17020	hypothetical protein
AQR24_RS27155	tyrosine-type recombinase/integrase
AQR24_RS21160	tyrosine-type recombinase/integrase
RALB5_RS19955	tyrosine-type recombinase/integrase
AQR24_RS17280	hypothetical protein
HXP34_20075	hypothetical protein

Moko typical	
**Gene**	**Annotation**
HXP35_20280	hypothetical protein
AQR24_RS28075	transposase
RALB5_RS01235	UDP-glucuronosyltransferase
RALB5_RS27385	hypothetical protein
AQR24_RS27980	hypothetical protein
HXP35_21670	hypothetical protein
RALB5_RS26535	ankyrin repeat domain-containing protein
AQR24_RS04920	discoidin domain-containing protein
RALB5_RS27845	hypothetical protein
HXP34_21925	hypothetical protein
RALB5_RS22950	hypothetical protein
EIH10_RS08445	hypothetical protein
EIH12_RS23945	hypothetical protein
AQR21_RS20960	porin
HXP37_18885	DUF3309 domain-containing protein
RALGR_RS24945	hypothetical protein
EIH13_RS24340	hypothetical protein
AQR24_RS24260	IS3 family transposase
RALGR_RS23470	IS3 family transposase
AQR24_RS11405	TolC family protein
RALGR_RS25960	hypothetical protein
HXP35_17685	hypothetical protein
AQR21_RS13065	heavy metal translocating P-type ATPase
RALGR_RS13970	hypothetical protein
AQR24_RS28150	hypothetical protein

Moko typical	
**Gene**	**Annotation**
EIH14_RS22560	hypothetical protein
RALB5_RS25480	aminotransferase class I/II-fold pyridoxal phosphate-dependent enzyme
RALGR_RS25775	hypothetical protein
EIH12_RS14450	DUF3761 domain-containing protein
RALGR_RS25810	hypothetical protein
EIH10_RS13805	type II toxin-antitoxin system HicA family toxin
EIH12_RS11730	HicB family protein
RALGR_RS25830	hypothetical protein
AQR21_RS05215	DUF2946 domain-containing protein
EIH12_RS23970	hypothetical protein
AQR21_RS24135	GNAT family N-acetyltransferase
EIH10_RS19215	hypothetical protein
RALGR_RS01790	nucleotidyl transferase AbiEii/AbiGii toxin family protein
EIH10_RS23425	hypothetical protein
EIH10_RS08080	pilus assembly protein
EIH10_RS23010	hypothetical protein
RALGR_RS02475	hypothetical protein
RALGR_RS02550	hypothetical protein
RALGR_RS25890	hypothetical protein
AQR21_RS20155	tartrate dehydrogenase
EIH10_RS21390	hypothetical protein
AQR21_RS25795	hypothetical protein
AQR21_RS16215	DNA-binding protein
RALGR_RS05020	hypothetical protein
EIH12_RS11875	hypothetical protein

Moko typical	
**Gene**	**Annotation**
EIH12_RS11870	hypothetical protein
AQR21_RS03900	hypothetical protein
AQR21_RS18645	helix-turn-helix domain-containing protein
EIH10_RS18860	hypothetical protein
AQR21_RS24810	hypothetical protein
EIH12_RS05275	hypothetical protein
EIH10_RS18840	DDE-type integrase/transposase/recombinase
EIH12_RS02725	ATP-binding protein
RALGR_RS06965	hypothetical protein
EIH10_RS18825	hypothetical protein
EIH12_RS02710	hypothetical protein
EIH12_RS02700	hypothetical protein
EIH12_RS02695	hypothetical protein
EIH12_RS02690	hypothetical protein
AQR21_RS18700	DUF3164 family protein
EIH10_RS08405	site-specific DNA-methyltransferase
AQR21_RS16995	hypothetical protein
EIH10_RS08380	DUF3489 domain-containing protein
EIH10_RS08375	hypothetical protein
AQR21_RS16960	hypothetical protein
EIH12_RS19625	calcium-binding protein
RALGR_RS09200	site-specific integrase
EIH12_RS16065	type III effector protein
RALGR_RS09740	hypothetical protein
AQR21_RS15290	hypothetical protein

Moko typical	
**Gene**	**Annotation**
RALGR_RS09750	hypothetical protein
AQR21_RS24705	helix-turn-helix domain-containing protein
EIH12_RS13340	cysteine desulfurase
EIH10_RS21295	class I SAM-dependent methyltransferase
EIH12_RS15535	MFS transporter
EIH10_RS09010	hypothetical protein
EIH10_RS23495	hypothetical protein
EIH12_RS06690	DUF4276 family protein
RALGR_RS11935	hypothetical protein
EIH10_RS13100	hypothetical protein
RALGR_RS24375	2OG-Fe(II) oxygenase
AQR21_RS00145	LysR family transcriptional regulator
EIH10_RS22445	glutamine amidotransferase
EIH10_RS03915	hypothetical protein
EIH12_RS18425	hypothetical protein
EIH12_RS15415	recombinase family protein
RALGR_RS13980	helix-turn-helix transcriptional regulator
RALGR_RS14255	hypothetical protein
EIH12_RS19115	hypothetical protein
RALGR_RS15325	DUF1444 family protein
AQR21_RS06445	YigZ family protein
RALGR_RS15535	glycoside hydrolase family 5 protein
RALGR_RS25105	hypothetical protein
AQR21_RS25780	hypothetical protein
AQR21_RS06580	hypothetical protein

Moko typical	
**Gene**	**Annotation**
RALGR_RS16960	hypothetical protein
EIH12_RS05905	CPBP family intramembrane metalloprotease
AQR21_RS04225	hypothetical protein
RALGR_RS25170	hypothetical protein
EIH12_RS21855	replication-associated recombination protein A
AQR21_RS11530	hypothetical protein
EIH12_RS15045	DUF1484 domain-containing protein
AQR21_RS15390	ImmA/IrrE family metallo-endopeptidase
RALGR_RS25195	DUF4411 family protein
EIH10_RS22470	DUF3018 family protein
EIH12_RS15155	DUF3489 domain-containing protein
RALGR_RS26175	hypothetical protein
AQR21_RS13800	hypothetical protein
RALGR_RS18420	DUF3375 domain-containing protein
EIH12_RS15140	DUF4194 domain-containing protein
EIH10_RS15820	ATP-dependent exonuclease SbcCD, C subunit-like protein
RALGR_RS18435	hypothetical protein
RALGR_RS18830	hypothetical protein
EIH10_RS21690	acyl carrier protein
RALGR_RS25305	hypothetical protein
AQR21_RS12335	DUF5076 domain-containing protein
RALGR_RS20290	pilin
RALGR_RS20355	site-specific integrase
EIH10_RS18685	hypothetical protein
RALGR_RS26245	hypothetical protein

Moko typical	
**Gene**	**Annotation**
EIH10_RS14135	hypothetical protein
EIH10_RS14130	hypothetical protein
EIH10_RS19475	hypothetical protein
EIH10_RS10545	hypothetical protein
EIH12_RS23280	BON domain-containing protein
EIH12_RS09410	hypothetical protein
EIH12_RS09405	DUF4123 domain-containing protein
RALB5_RS19645	hypothetical protein
EIH10_RS22145	DUF769 domain-containing protein
RALGR_RS25445	hypothetical protein
EIH10_RS22370	hypothetical protein
EIH10_RS22375	hypothetical protein
RALGR_RS22810	DUF3304 domain-containing protein
EIH10_RS11420	hypothetical protein
AQR21_RS22105	hypothetical protein
EIH10_RS23095	hypothetical protein
EIH10_RS23090	hypothetical protein
EIH12_RS23830	hypothetical protein
EIH12_RS23775	zinc-binding dehydrogenase
EIH10_RS19405	hypothetical protein
group_7305	IS66 family insertion sequence element accessory protein TnpB
RALFB_RS08535	tail protein X
EIH11_RS19405	hypothetical protein
EIH10_RS19520	hypothetical protein
EIH10_RS22475	ATP-binding protein

Moko typical	
**Gene**	**Annotation**
EIH10_RS17995	hypothetical protein
RALFB_RS27390	IS5 family transposase
RSPO_RS23755	IS5 family transposase
EIH11_RS10160	phage terminase large subunit family protein
AQR21_RS23515	IS701 family transposase
RALB5_RS24570	IS701 family transposase
AQR21_RS18005	hypothetical protein
HXP35_12855	VUT family protein
HXP34_14455	calcium-binding protein
HXP34_03055	type VI secretion system tip protein VgrG
vgrG	type VI secretion system tip protein VgrG
HXP36_07705	hypothetical protein
RSPO_RS01110	hypothetical protein
RALGR_RS23820	hypothetical protein
EIH10_RS18710	membrane protein
RALB5_RS13980	ABC transporter substrate-binding protein
AQR21_RS23835	BON domain-containing protein
AQR21_RS25345	IS630 family transposase
AQR24_RS25355	tail protein
RALW1_RS14400	AAA domain-containing protein
EIH10_RS19760	hypothetical protein
AQR21_RS03800	hypothetical protein
AQR21_RS03865	phage head morphogenesis protein
AQR21_RS00160	hypothetical protein
EIH12_RS22425	hypothetical protein

Moko typical	
**Gene**	**Annotation**
AQR21_RS22110	hypothetical protein
EIH12_RS13320	DUF3304 domain-containing protein
AQR21_RS24825	hypothetical protein
HXP34_01520	site-specific DNA-methyltransferase
HXP34_18740	HAMP domain-containing protein
HXP35_03080	sel1 repeat family protein
HXP34_13675	SPOR domain-containing protein
AQR21_RS00130	tartrate dehydrogenase
RALB5_RS24260	DUF3274 domain-containing protein
AQR24_RS25795	hypothetical protein
RALB5_RS09260	methyltransferase
HXP36_07750	DUF2303 family protein
HXP36_07835	hypothetical protein
HXP36_07855	phage gp6-like head-tail connector protein
AQR24_RS24905	tail fiber protein
EIH10_RS15765	hypothetical protein
EIH14_RS13500	hypothetical protein
RALGR_RS21950	hypothetical protein
EIH14_RS09465	acyl-CoA/acyl-ACP dehydrogenase
EIH11_RS20645	hypothetical protein
HXP36_25100	hypothetical protein
RALB5_RS23515	DUF3459 domain-containing protein
AQR24_RS03180	hypothetical protein
virB11	P-type DNA transfer ATPase VirB11
AQR24_RS03215	type IV secretion system protein

Moko typical	
**Gene**	**Annotation**
AQR24_RS23675	S8 family serine peptidase
UW163_RS24080	hypothetical protein
UW163_RS24070	ParB/RepB/Spo0J family partition protein
AQR24_RS00120	NAD(P)-binding domain-containing protein
RALB5_RS19505	hypothetical protein
RALB5_RS21730	hypothetical protein
RALB5_RS22715	hypothetical protein
RALB5_RS26775	trypsin-like peptidase domain-containing protein
RALB5_RS23775	site-specific DNA-methyltransferase
AQR24_RS24850	hypothetical protein
RALB5_RS24485	hypothetical protein
RALB5_RS24665	glycoside hydrolase family protein
AQR24_RS25870	PRTRC system ThiF family protein
AQR21_RS24180	hypothetical protein
RALGR_RS05490	hypothetical protein
EIH12_RS22835	hypothetical protein
AQR21_RS16900	DUF1998 domain-containing protein
AQR24_RS25300	IS3 family transposase
EIH12_RS12370	hypothetical protein
UW163_RS23690	hypothetical protein
RALGR_RS26170	hypothetical protein
EIH12_RS23490	hypothetical protein
AQR21_RS00165	type II toxin-antitoxin system prevent-host-death family antitoxin
RALGR_RS26275	hypothetical protein
RALGR_RS26285	hypothetical protein

Moko typical	
**Gene**	**Annotation**
RALGR_RS26310	hypothetical protein
EIH12_RS09675	hypothetical protein
EIH12_RS15255	hypothetical protein
EIH10_RS19340	terminase
AQR21_RS23405	hypothetical protein
EIH14_RS22635	head completion/stabilization protein
EIH11_RS19415	DUF3380 domain-containing protein
RALFB_RS08560	phage tail protein
RALFB_RS08580	baseplate J-like protein
EIH10_RS15770	hypothetical protein
EIH14_RS05620	type III effector protein
RALGR_RS24530	H-NS histone family protein
AQR24_RS27280	hypothetical protein
HXP36_13600	DUF1484 domain-containing protein
AQR24_RS24325	LysR family transcriptional regulator
HXP35_11270	hypothetical protein
AQR24_RS17095	glycine hydroxymethyltransferase
RALW1_RS17355	EamA family transporter
HXP34_16125	hypothetical protein
HXP34_16150	IS5 family transposase
HXP35_16510	pilus assembly protein PilN
HXP34_01660	hypothetical protein
HXP34_17285	LysR family transcriptional regulator
AQR24_RS26590	hypothetical protein
AQR24_RS14080	erythromycin esterase family protein

Moko typical	
**Gene**	**Annotation**
EIH10_RS21370	type III effector protein
AQR24_RS22845	hypothetical protein
HXP37_05250	type II toxin-antitoxin system ParD family antitoxin
UW163_RS05290	type II toxin-antitoxin system RelE/ParE family toxin
RALB5_RS22305	hypothetical protein
AQR24_RS23295	deoxynucleotide monophosphate kinase
HXP36_07845	HK97 family phage prohead protease
HXP36_07850	phage major capsid protein
EIH14_RS06425	phage head closure protein
HXP36_07865	hypothetical protein
HXP36_07955	hypothetical protein
C2I33_RS25260	biotin/lipoyl-binding protein
EIH11_RS19205	hypothetical protein
RALW1_RS07020	AMP-binding protein
RALFB_RS00085	porin
AQR24_RS07750	hypothetical protein
RALB5_RS08790	DUF2235 domain-containing protein
AQR24_RS03165	hypothetical protein
RALB5_RS14010	hypothetical protein
AQR24_RS03185	type IV secretory system conjugative DNA transfer family protein
AQR24_RS03190	hypothetical protein
UW163_RS23860	TrbG/VirB9 family P-type conjugative transfer protein
RALB5_RS14055	type IV secretion system protein
virB5	P-type DNA transfer protein VirB5
RALGR_RS00115	hypothetical protein

Moko typical	
**Gene**	**Annotation**
RALB5_RS15555	DUF1911 domain-containing protein
group_6687	DNA repair protein RadC
RALB5_RS26545	4'-phosphopantetheinyl transferase superfamily protein
EIH12_RS15125	filamentous hemagglutinin N-terminal domain-containing protein
RALB5_RS22880	phage major capsid protein, P2 family
AQR21_RS25410	hypothetical protein
RALB5_RS23135	phage tail protein I
AQR24_RS17425	AbrB/MazE/SpoVT family DNA-binding domain-containing protein
RALB5_RS23440	type II toxin-antitoxin system VapC family toxin
RALB5_RS23445	hypothetical protein
RALB5_RS23450	hypothetical protein
RALB5_RS26795	Com family DNA-binding transcriptional regulator
RALFB_RS08615	GpE family phage tail protein
EIH14_RS04340	GALA protein
EIH09_RS00170	adenylate/guanylate cyclase domain-containing protein
EIH12_RS23425	DUF4276 family protein
RALGR_RS25860	hypothetical protein
AQR21_RS24760	hypothetical protein
RALGR_RS01855	AAA family ATPase
RALGR_RS01870	ATP-dependent DNA ligase
EIH12_RS13955	metal-dependent phosphohydrolase
EIH12_RS04195	hypothetical protein
EIH12_RS04200	hypothetical protein
RALGR_RS25905	hypothetical protein
RALGR_RS03170	VOC family protein

Moko typical	
**Gene**	**Annotation**
AQR21_RS24390	hypothetical protein
EIH12_RS04220	MBL fold metallo-hydrolase
AQR21_RS09130	hypothetical protein
EIH12_RS04240	hypothetical protein
AQR21_RS09120	metallophosphoesterase
EIH12_RS04290	hypothetical protein
AQR21_RS03785	hypothetical protein
RALGR_RS24345	LamG domain-containing protein
RALGR_RS05520	hypothetical protein
EIH12_RS11950	tape measure protein
EIH12_RS11945	hypothetical protein
AQR21_RS03820	hypothetical protein
AQR21_RS03825	hypothetical protein
RALGR_RS05545	hypothetical protein
RALGR_RS05550	DUF1320 domain-containing protein
EIH12_RS11920	hypothetical protein
RALGR_RS05565	hypothetical protein
RALGR_RS05590	hypothetical protein
RALGR_RS05595	DUF1804 family protein
EIH12_RS11865	hypothetical protein
EIH12_RS11860	hypothetical protein
RALGR_RS05625	hypothetical protein
RALGR_RS05630	regulatory protein GemA
AQR21_RS03915	hypothetical protein
RALGR_RS05640	hypothetical protein

Moko typical	
**Gene**	**Annotation**
UW163_RS23715	hypothetical protein
RALGR_RS26000	hypothetical protein
AQR21_RS02125	helix-turn-helix transcriptional regulator
RALGR_RS09725	DEAD/DEAH box helicase
EIH12_RS22975	MFS transporter
AQR21_RS17995	hypothetical protein
RALGR_RS26045	hypothetical protein
UW163_RS23705	DUF4338 domain-containing protein
AQR21_RS24530	site-specific integrase
RALGR_RS18445	tail assembly protein
EIH12_RS13480	C40 family peptidase
RALGR_RS20190	tyrosine-type recombinase/integrase
RALGR_RS25345	phage tail protein
EIH10_RS19285	phage tail tape measure protein
EIH12_RS21485	hypothetical protein
EIH12_RS21480	DUF4035 domain-containing protein
RALGR_RS20565	hypothetical protein
RALGR_RS20570	phage major tail 2 family protein
RALGR_RS20575	DUF3168 domain-containing protein
EIH12_RS21460	phage head closure protein
EIH10_RS19320	phage gp6-like head-tail connector protein
EIH10_RS19325	phage portal protein
EIH10_RS19465	hypothetical protein
EIH12_RS23415	protein phosphatase
AQR21_RS25475	hypothetical protein

Moko typical	
**Gene**	**Annotation**
EIH12_RS22415	hypothetical protein
EIH12_RS22410	transposase
EIH12_RS14065	hypothetical protein
EIH12_RS14030	DUF2059 domain-containing protein
EIH12_RS14025	lysozyme
AQR21_RS21165	hypothetical protein
RALGR_RS21040	hypothetical protein
EIH12_RS14010	hypothetical protein
EIH12_RS14005	hypothetical protein
AQR21_RS21185	DNA-dependent RNA polymerase
RALGR_RS21060	hypothetical protein
AQR21_RS21195	hypothetical protein
AQR21_RS24885	hypothetical protein
RALGR_RS21085	phosphodiesterase
AQR21_RS19195	hypothetical protein
RALGR_RS21480	hypothetical protein
RALGR_RS21555	DUF1484 family protein
RALGR_RS21560	helix-turn-helix transcriptional regulator
EIH12_RS23800	hypothetical protein
EIH10_RS22260	IS5-like element IS1021 family transposase
AQR21_RS21900	ankyrin repeat domain-containing protein
EIH12_RS15245	hypothetical protein
AQR21_RS23490	DNA-binding protein
EIH12_RS23725	hypothetical protein
RALGR_RS26365	hypothetical protein

Moko typical	
**Gene**	**Annotation**
EIH10_RS19525	Sel1 repeat protein
AQR21_RS24720	DUF746 domain-containing protein
AQR21_RS22095	DUF4411 family protein
EIH12_RS21710	IS630 family transposase
RALGR_RS26395	hypothetical protein
EIH12_RS21440	peptidase U35
AQR21_RS26020	AAA family ATPase
AQR21_RS15755	hypothetical protein
EIH12_RS24180	hypothetical protein
AQR21_RS23400	hypothetical protein
HXP35_19910	type III effector protein
AQR21_RS24665	preprotein translocase subunit SecF
AQR21_RS18210	hypothetical protein
EIH12_RS23395	hypothetical protein
AQR21_RS25905	hypothetical protein
AQR21_RS23150	hypothetical protein
EIH12_RS15170	DNA-binding protein
AQR21_RS16895	hypothetical protein
AQR24_RS03205	hypothetical protein
AQR21_RS23360	glutamate/aspartate ABC transporter substrate-binding protein
EIH13_RS05295	hypothetical protein
UW163_RS05850	AAA family ATPase
UW163_RS09545	AAA family ATPase
EIH09_RS23450	IS21 family transposase
EIH13_RS17250	IS21 family transposase

Moko typical	
**Gene**	**Annotation**
EIH13_RS22220	IS3 family transposase
AQR24_RS27100	SDR family NAD(P)-dependent oxidoreductase
RALGR_RS25980	hypothetical protein
EIH10_RS19515	peptidyl-prolyl cis-trans isomerase
HXP34_16805	DcrB-related protein
HXP36_21580	S-layer family protein
HXP35_03090	phage portal protein
HXP34_20625	phage portal protein
EIH14_RS22650	phage portal protein
HXP35_03095	oxidoreductase
HXP35_20790	oxidoreductase
HXP34_20465	TetR/AcrR family transcriptional regulator
HXP34_17055	PAAR domain-containing protein
RALGR_RS20540	phage minor tail protein L
RALGR_RS23965	host specificity protein J
EIH12_RS23155	host specificity protein J
HXP35_18950	PAAR domain-containing protein
RALB5_RS25040	hypothetical protein
HXP36_14390	M48 family metallopeptidase
AQR21_RS15330	hypothetical protein
UW163_RS16210	hypothetical protein
HXP37_05465	hypothetical protein
AQR24_RS27485	IS701 family transposase
EIH12_RS12355	DEAD/DEAH box helicase
EIH12_RS23300	DUF559 domain-containing protein

Moko typical	
**Gene**	**Annotation**
EIH10_RS23620	hypothetical protein
EIH12_RS23285	hypothetical protein
EIH12_RS18300	hypothetical protein
HXP35_16965	hypothetical protein
UW163_RS05355	phage portal protein
RALFB_RS25790	transposase
HXP34_00495	hypothetical protein
HXP37_05305	hypothetical protein
AQR24_RS27225	IS5 family transposase
EIH12_RS16885	hypothetical protein
HXP34_10055	lysis protein
HXP35_17745	DUF2345 domain-containing protein
EIH10_RS12535	SDR family NAD(P)-dependent oxidoreductase
HXP35_20620	IS3 family transposase
EIH10_RS15755	hypothetical protein
HXP36_18735	class II aldolase/adducin family protein
RALW1_RS02040	LysR family transcriptional regulator
UW163_RS11525	hypothetical protein
UW163_RS11575	VCBS repeat-containing protein
HXP37_11610	helix-turn-helix transcriptional regulator
UW163_RS25795	hypothetical protein
AQR24_RS25075	phage major tail tube protein
AQR24_RS14890	glycosyltransferase family 2 protein
RALB5_RS25705	WecB/TagA/CpsF family glycosyltransferase
AQR24_RS25265	IS5 family transposase

Moko typical	
**Gene**	**Annotation**
RALB5_RS11520	filamentous hemagglutinin N-terminal domain-containing protein
RALB5_RS19755	hypothetical protein
AQR24_RS18595	hypothetical protein
RALB5_RS19775	hypothetical protein
AQR24_RS24275	hypothetical protein
RALB5_RS20795	hypothetical protein
RALB5_RS22430	DDE-type integrase/transposase/recombinase
AQR24_RS03255	AAA family ATPase
AQR24_RS23555	AAA family ATPase
AQR24_RS23100	tail assembly protein
RALB5_RS23615	IS4 family transposase
RALB5_RS23660	terminase endonuclease subunit
group_3846	group II intron reverse transcriptase/maturase
AQR24_RS18490	hypothetical protein
RALB5_RS24010	recombinase family protein
AQR24_RS03245	recombinase family protein
RALB5_RS24290	DDE-type integrase/transposase/recombinase
AQR24_RS24845	DUF1484 domain-containing protein
RALB5_RS24470	DUF3304 domain-containing protein
RALB5_RS24500	hypothetical protein
AQR24_RS24420	IS4 family transposase
AQR24_RS25080	phage tail assembly protein
AQR24_RS25800	hypothetical protein
RALB5_RS25230	DUF1484 domain-containing protein
AQR24_RS00300	phage holin family protein

Moko typical	
**Gene**	**Annotation**
RALB5_RS25425	DUF1983 domain-containing protein
RALB5_RS27175	DGQHR domain-containing protein
RALW1_RS05320	DUF853 family protein
AQR21_RS12775	hypothetical protein
RALGR_RS07540	hypothetical protein
AQR21_RS22900	N-6 DNA methylase
AQR21_RS23330	sel1 repeat family protein
RALGR_RS25595	MBL fold metallo-hydrolase
RALGR_RS25690	hypothetical protein
AQR21_RS25065	TetR family transcriptional regulator
RALFB_RS27430	hypothetical protein
AQR24_RS03235	transporter
EIH10_RS15760	hypothetical protein
AQR21_RS24395	hypothetical protein
AQR21_RS17940	MFS transporter
AQR21_RS19200	DNA polymerase A family protein
UW163_RS23810	helix-turn-helix transcriptional regulator
UW163_RS23805	M81 family metallopeptidase
AQR21_RS25940	hypothetical protein
EIH10_RS07640	hypothetical protein
EIH12_RS24165	hypothetical protein
EIH09_RS24155	hypothetical protein
EIH14_RS00500	DUF596 domain-containing protein
EIH11_RS01570	hypothetical protein
HXP34_16795	hypothetical protein

Moko typical	
**Gene**	**Annotation**
HXP35_01150	HNH endonuclease
AQR24_RS27895	hypothetical protein
HXP35_03075	phosphatidylserine/phosphatidylglycerophosphate/ cardiolipin synthase family protein
HXP34_03070	PAAR domain-containing protein
EIH11_RS19225	hypothetical protein
HXP34_06825	hypothetical protein
AQR21_RS24040	H-NS histone family protein
HXP34_07230	toprim domain-containing protein
HXP34_07260	hypothetical protein
HXP34_20525	hypothetical protein
HXP35_20675	DUF3313 domain-containing protein
AQR24_RS23755	phage virion morphogenesis protein
HXP35_08180	hypothetical protein
HXP34_08230	hypothetical protein
HXP34_08240	hypothetical protein
HXP34_08280	hypothetical protein
HXP35_08255	hypothetical protein
AQR24_RS26080	phage virion morphogenesis protein
AQR24_RS25280	phage virion morphogenesis protein
HXP35_16945	hypothetical protein
HXP34_09845	hypothetical protein
HXP34_16110	filamentous hemagglutinin N-terminal domain-containing protein
HXP34_10135	ISAs1 family transposase
HXP34_11105	MFS transporter
HXP34_16130	filamentous hemagglutinin N-terminal domain-containing protein

Moko typical	
**Gene**	**Annotation**
HXP35_13765	hypothetical protein
HXP34_13975	hypothetical protein
HXP35_16210	hypothetical protein
EIH09_RS23795	head decoration protein
HXP34_16265	hypothetical protein
HXP34_16305	conjugal transfer protein TrbJ
group_4558	type IV pilus biogenesis protein PilP
HXP35_17750	DUF4123 domain-containing protein
HXP35_17755	DUF3304 domain-containing protein
HXP35_17760	DUF2235 domain-containing protein
HXP35_17825	hypothetical protein
HXP34_18480	Cro/Cl family transcriptional regulator
RALGR_RS05695	sigma-70 family RNA polymerase sigma factor
AQR21_RS18955	PAS domain-containing protein
HXP35_20490	phosphatidylserine/phosphatidylglycerophosphate/ cardiolipin synthase family protein
HXP34_20330	sel1 repeat family protein
HXP34_20405	hypothetical protein
HXP35_21705	hypothetical protein
HXP36_07755	hypothetical protein
RALFB_RS27445	ATP-binding cassette domain-containing protein
RALB5_RS13230	AMP-binding protein
HXP34_01755	filamentous hemagglutinin N-terminal domain-containing protein
EIH13_RS10645	hypothetical protein
HXP36_18730	MFS transporter
HXP36_18740	LysR family transcriptional regulator

Moko typical	
**Gene**	**Annotation**
UW163_RS01300	hypothetical protein
UW163_RS05190	N-6 DNA methylase
HXP37_05150	hypothetical protein
HXP37_05155	site-specific recombinase resolvase
popP3	type III secretion system YopJ family effector PopP3
HXP37_05200	helix-turn-helix domain-containing protein
HXP37_05205	ATP-binding protein
HXP37_05270	type III secretion system effector protein
HXP37_05275	hypothetical protein
HXP37_05280	hypothetical protein
HXP37_05285	DUF3489 domain-containing protein
UW163_RS05345	hypothetical protein
UW163_RS25630	hypothetical protein
HXP37_05520	hypothetical protein
UW163_RS22555	IS1595 family transposase
UW163_RS25725	SLATT domain-containing protein
HXP37_11570	hypothetical protein
HXP37_11575	hypothetical protein
HXP37_11580	hypothetical protein
UW163_RS11555	hypothetical protein
HXP37_11590	hypothetical protein
HXP37_11605	hypothetical protein
HXP37_15615	site-specific integrase
HXP37_15620	hypothetical protein
HXP37_15625	GFA family protein

Moko typical	
**Gene**	**Annotation**
UW163_RS15625	hypothetical protein
UW163_RS15630	H-NS histone family protein
HXP37_15640	hypothetical protein
HXP37_15645	hypothetical protein
UW163_RS15645	hypothetical protein
UW163_RS25810	hypothetical protein
HXP37_15665	hypothetical protein
HXP37_15670	hypothetical protein
UW163_RS15670	hypothetical protein
UW163_RS15675	DNA-deoxyinosine glycosylase
HXP34_16830	hypothetical protein
HXP34_17465	type VI secretion system lipoprotein TssJ
HXP34_18770	(2Fe-2S)-binding protein
UW163_RS16185	DUF1484 family protein
UW163_RS26305	hypothetical protein
RALGR_RS23815	filamentous hemagglutinin N-terminal domain-containing protein
RSPO_RS25735	hypothetical protein
AQR24_RS27435	ISAs1 family transposase
AQR21_RS23695	ABC transporter substrate-binding protein
AQR24_RS00065	transcriptional regulator
AQR24_RS26355	hypothetical protein
AQR24_RS00140	LD-carboxypeptidase
RALB5_RS27275	IS5 family transposase
group_6526	type VI secretion system tip protein VgrG
RALB5_RS25965	DUF4158 domain-containing protein

Moko typical	
**Gene**	**Annotation**
RALB5_RS00375	hypothetical protein
AQR24_RS22980	hypothetical protein
RALB5_RS03220	glycosyltransferase family 4 protein
AQR24_RS14900	SpoIIE family protein phosphatase
AQR24_RS14905	amino acid ABC transporter substrate-binding protein
RALB5_RS03235	hypothetical protein
RALB5_RS03240	oligosaccharide flippase family protein
AQR24_RS14920	O-antigen ligase domain-containing protein
RALB5_RS03250	hypothetical protein
AQR24_RS14930	hypothetical protein
AQR24_RS14935	hypothetical protein
AQR24_RS14940	STAS domain-containing protein
RALB5_RS03270	hypothetical protein
AQR24_RS14950	response regulator
RALB5_RS03280	response regulator
AQR24_RS14960	sugar transferase
RALB5_RS27345	hypothetical protein
AQR24_RS24810	hypothetical protein
RALB5_RS03945	hypothetical protein
RALB5_RS03950	hypothetical protein
AQR24_RS28445	hypothetical protein
AQR24_RS24795	hypothetical protein
RALB5_RS03960	hypothetical protein
RALB5_RS03965	hypothetical protein
RALB5_RS05655	hypothetical protein

Moko typical	
**Gene**	**Annotation**
AQR24_RS03005	hypothetical protein
RALB5_RS06930	hypothetical protein
AQR24_RS02995	hypothetical protein
AQR24_RS02990	hypothetical protein
AQR24_RS14850	response regulator transcription factor
AQR24_RS14855	serine acetyltransferase
AQR24_RS14860	glycosyltransferase family 2 protein
RALB5_RS07135	glycosyltransferase
RALB5_RS07140	acyltransferase
RALB5_RS07145	glycosyltransferase
RALB5_RS07150	glycosyltransferase
RALB5_RS07155	acyltransferase
AQR24_RS27905	hypothetical protein
AQR24_RS03100	excisionase family DNA-binding protein
RALB5_RS07965	IS110 family transposase
RALB5_RS07970	acyltransferase family protein
RALB5_RS09990	HlyD family type I secretion periplasmic adaptor subunit
RALB5_RS10005	hypothetical protein
RALB5_RS10015	hypothetical protein
RALB5_RS27455	hypothetical protein
AQR24_RS04055	hypothetical protein
AQR24_RS27940	hypothetical protein
RALB5_RS27465	hypothetical protein
AQR24_RS26575	DUF2335 domain-containing protein
RALB5_RS27470	hypothetical protein

Moko typical	
**Gene**	**Annotation**
AQR24_RS04040	LexA family transcriptional regulator
RALB5_RS26275	hypothetical protein
RALB5_RS10105	hypothetical protein
AQR24_RS27925	hypothetical protein
AQR24_RS04030	hypothetical protein
AQR24_RS04025	hypothetical protein
RALB5_RS10125	hypothetical protein
RALB5_RS10130	hypothetical protein
AQR24_RS04010	hypothetical protein
AQR24_RS04005	phage terminase large subunit family protein
AQR24_RS27920	hypothetical protein
RALB5_RS10150	phage portal protein
RALB5_RS10155	hypothetical protein
RALB5_RS10160	DUF2190 family protein
AQR24_RS03980	hypothetical protein
RALB5_RS10170	hypothetical protein
RALB5_RS10175	hypothetical protein
RALB5_RS10180	hypothetical protein
AQR24_RS03960	hypothetical protein
RALB5_RS10190	phage tail tape measure protein
AQR24_RS03950	phage tail protein
AQR24_RS03945	tail fiber protein
RALB5_RS10205	tail assembly protein
AQR24_RS03935	phage minor tail protein L
RALB5_RS11350	hypothetical protein

Moko typical	
**Gene**	**Annotation**
AQR24_RS10815	site-specific integrase
AQR24_RS10810	hypothetical protein
RALB5_RS11450	hypothetical protein
RALB5_RS27510	hypothetical protein
RALB5_RS25735	hypothetical protein
RALB5_RS27520	hypothetical protein
AQR24_RS19640	type III effector protein with ppr repeats
AQR24_RS00130	sce7726 family protein
AQR24_RS11680	GALA protein
RALB5_RS13105	LysR family transcriptional regulator
mdcA	malonate decarboxylase subunit alpha
AQR24_RS11715	triphosphoribosyl-dephospho-CoA synthase
AQR24_RS11720	malonate decarboxylase subunit delta
AQR24_RS11725	biotin-independent malonate decarboxylase subunit beta
mdcE	biotin-independent malonate decarboxylase subunit gamma
madL	malonate transporter subunit MadL
madM	malonate transporter subunit MadM
RALB5_RS13145	malonate decarboxylase holo-ACP synthase
mdcH	malonate decarboxylase subunit epsilon
RALB5_RS13155	LysR family transcriptional regulator
RALB5_RS13160	carbon-nitrogen hydrolase family protein
AQR24_RS11765	molybdopterin-dependent oxidoreductase
RALB5_RS13170	cystathionine beta-lyase
RALB5_RS13985	hypothetical protein
RALB5_RS13990	hypothetical protein

Moko typical	
**Gene**	**Annotation**
RALB5_RS25750	hypothetical protein
AQR24_RS03170	hypothetical protein
AQR24_RS03200	TrbI/VirB10 family protein
RALB5_RS14060	hypothetical protein
RALB5_RS14095	hypothetical protein
group_6663	group II intron reverse transcriptase/maturase
group_6670	allantoicase
group_6672	type VI secretion system tip protein VgrG
RALB5_RS27615	hypothetical protein
AQR24_RS28385	hypothetical protein
AQR24_RS23785	MFS transporter
RALB5_RS19760	hypothetical protein
AQR24_RS23075	RES family NAD+ phosphorylase
AQR24_RS23070	trypsin-like peptidase domain-containing protein
AQR24_RS18025	H-NS histone family protein
RALB5_RS19785	hypothetical protein
AQR24_RS18010	hypothetical protein
RALB5_RS19800	DNA-binding protein
RALB5_RS19805	DUF1778 domain-containing protein
AQR24_RS17995	GNAT family N-acetyltransferase
RALB5_RS19815	AAA family ATPase
AQR24_RS17985	hypothetical protein
RALB5_RS19825	HORMA domain containing protein
AQR24_RS17975	nucleotidyltransferase
RALB5_RS19835	hypothetical protein

Moko typical	
**Gene**	**Annotation**
AQR24_RS17965	hypothetical protein
RALB5_RS19845	hypothetical protein
AQR24_RS17955	hypothetical protein
AQR24_RS17950	hypothetical protein
RALB5_RS19860	hypothetical protein
AQR24_RS17940	DUF4123 domain-containing protein
RALB5_RS19870	PAAR domain-containing protein
RALB5_RS19875	hypothetical protein
RALB5_RS25785	hypothetical protein
AQR24_RS21270	hypothetical protein
AQR24_RS28300	hypothetical protein
RALB5_RS20085	GGDEF domain-containing protein
AQR24_RS12425	hypothetical protein
AQR24_RS12420	hypothetical protein
RALB5_RS20505	helix-turn-helix transcriptional regulator
AQR24_RS18615	RepB family plasmid replication initiator protein
AQR24_RS18610	ParB/RepB/Spo0J family partition protein
RALB5_RS20725	ParA family protein
AQR24_RS18600	tyrosine-type recombinase/integrase
RALB5_RS20740	hypothetical protein
RALB5_RS20745	RES family NAD+ phosphorylase
RALB5_RS26605	tyrosine-type recombinase/integrase
RALB5_RS20800	DDE-type integrase/transposase/recombinase
RALB5_RS25550	Tn3 family transposase
RALB5_RS20805	ATP-binding protein

Moko typical	
**Gene**	**Annotation**
AQR24_RS28155	hypothetical protein
RALB5_RS20810	tyrosine-type recombinase/integrase
RALB5_RS20815	DEAD/DEAH box helicase
RALB5_RS20825	hypothetical protein
RALB5_RS26615	DUF4158 domain-containing protein
RALB5_RS21025	GALA protein
AQR24_RS28235	hypothetical protein
AQR24_RS23505	ImmA/IrrE family metallo-endopeptidase
RALB5_RS21375	hypothetical protein
RALB5_RS21380	Tn3 family transposase
RALB5_RS21385	hypothetical protein
AQR24_RS23480	hypothetical protein
AQR24_RS23470	hypothetical protein
AQR24_RS24605	hypothetical protein
AQR24_RS24610	N-6 DNA methylase
RALB5_RS21415	XamI family restriction endonuclease
EIH12_RS23500	hypothetical protein
AQR24_RS23145	hypothetical protein
AQR24_RS23150	hypothetical protein
AQR24_RS23155	DUF4238 domain-containing protein
RALB5_RS21700	hypothetical protein
AQR24_RS23165	hypothetical protein
RALB5_RS21710	helix-turn-helix domain-containing protein
RALB5_RS21715	hypothetical protein
AQR24_RS23180	ATP-binding protein

Moko typical	
**Gene**	**Annotation**
RALB5_RS21760	hypothetical protein
RALB5_RS21765	hypothetical protein
EIH12_RS23485	hypothetical protein
RALB5_RS22295	hypothetical protein
RALB5_RS22300	hypothetical protein
AQR24_RS23240	aldo/keto reductase
RALB5_RS22540	MarR family transcriptional regulator
AQR24_RS23230	metal-dependent hydrolase
RALB5_RS22550	SDR family NAD(P)-dependent oxidoreductase
EIH10_RS21865	hypothetical protein
AQR24_RS23620	hypothetical protein
AQR24_RS23625	tail assembly protein
RALB5_RS22705	hypothetical protein
RALB5_RS22710	hypothetical protein
RALB5_RS22720	hypothetical protein
AQR24_RS20715	hypothetical protein
AQR24_RS00080	IS4 family transposase
RALB5_RS22915	hypothetical protein
RALB5_RS22920	hypothetical protein
AQR24_RS22200	hypothetical protein
AQR24_RS23430	DUF1186 domain-containing protein
AQR24_RS23435	DUF3489 domain-containing protein
RALB5_RS27830	hypothetical protein
RALB5_RS23565	NAD(P)-dependent alcohol dehydrogenase
RALB5_RS23570	TetR family transcriptional regulator

Moko typical	
**Gene**	**Annotation**
AQR24_RS23565	phage major capsid protein, P2 family
AQR24_RS23560	phage capsid scaffolding protein
group_6808	group II intron reverse transcriptase/maturase
AQR24_RS12590	hypothetical protein
AQR24_RS12595	hypothetical protein
RALB5_RS23745	ogr/Delta-like zinc finger family protein
AQR24_RS20680	transcriptional regulator
AQR24_RS28275	hypothetical protein
AQR24_RS20675	helix-turn-helix transcriptional regulator
AQR24_RS20670	hypothetical protein
AQR24_RS27115	hypothetical protein
RALB5_RS27850	hypothetical protein
RALB5_RS23880	hypothetical protein
RALB5_RS23890	site-specific integrase
RALB5_RS23895	hypothetical protein
AQR24_RS24530	hypothetical protein
AQR24_RS28215	hypothetical protein
RALB5_RS27865	hypothetical protein
AQR24_RS23540	SDR family oxidoreductase
RALB5_RS24190	hypothetical protein
RALB5_RS24195	type II toxin-antitoxin system RelE/ParE family toxin
AQR24_RS23950	ribbon-helix-helix protein, CopG family
AQR24_RS23955	DUF2807 domain-containing protein
AQR24_RS23960	hypothetical protein
RALB5_RS24265	DUF3304 domain-containing protein

Moko typical	
**Gene**	**Annotation**
RALB5_RS24270	DUF4123 domain-containing protein
RALB5_RS24305	phage tail protein
AQR24_RS28480	hypothetical protein
AQR24_RS28460	hypothetical protein
AQR24_RS00075	MipA/OmpV family protein
RALB5_RS24480	hypothetical protein
AQR24_RS24980	hypothetical protein
AQR24_RS25435	Lrp/AsnC family transcriptional regulator
RALB5_RS24580	hypothetical protein
AQR24_RS24910	tail assembly protein
AQR24_RS25380	hypothetical protein
AQR24_RS24025	hypothetical protein
AQR24_RS28650	hypothetical protein
RALB5_RS27910	hypothetical protein
RALB5_RS24795	membrane protein
AQR24_RS03240	hypothetical protein
AQR24_RS28520	hypothetical protein
AQR24_RS28735	hypothetical protein
RALB5_RS26970	hypothetical protein
AQR24_RS28530	hypothetical protein
RALB5_RS25020	hypothetical protein
RALB5_RS27020	hypothetical protein
RALB5_RS25025	hypothetical protein
RALB5_RS27040	DUF2345 domain-containing protein
AQR24_RS27480	Dam family site-specific DNA-(adenine-N6)-methyltransferase

Moko typical	
**Gene**	**Annotation**
RALB5_RS27975	hypothetical protein
RALB5_RS28020	sce7725 family protein
AQR24_RS12110	hypothetical protein
RALB5_RS28035	hypothetical protein
AQR24_RS26050	hypothetical protein
AQR24_RS25790	hypothetical protein
RALB5_RS28040	hypothetical protein
RALB5_RS27145	DUF596 domain-containing protein
AQR24_RS28720	hypothetical protein
RALB5_RS28120	hypothetical protein
AQR21_RS11225	DUF1488 domain-containing protein
AQR21_RS10475	hypothetical protein
AQR21_RS11210	hypothetical protein
RALGR_RS25840	hypothetical protein
AQR21_RS11220	hypothetical protein
AQR21_RS25395	hypothetical protein
RALGR_RS01860	hypothetical protein
AQR21_RS25685	hypothetical protein
RALGR_RS25950	hypothetical protein
EIH12_RS22850	hypothetical protein
RALGR_RS09715	hypothetical protein
RALGR_RS10485	iron-containing alcohol dehydrogenase
RALGR_RS10495	hypothetical protein
RALGR_RS24950	hypothetical protein
AQR21_RS24235	hypothetical protein

Moko typical	
**Gene**	**Annotation**
AQR21_RS25430	hypothetical protein
RALGR_RS26090	hypothetical protein
AQR21_RS24240	hypothetical protein
RALGR_RS25005	hypothetical protein
AQR21_RS24250	hypothetical protein
AQR21_RS09225	hypothetical protein
RALGR_RS26155	hypothetical protein
RALGR_RS25180	transposase
RALGR_RS26160	hypothetical protein
RALGR_RS18675	hypothetical protein
RALGR_RS26180	hypothetical protein
RALGR_RS25255	hypothetical protein
AQR21_RS25020	hypothetical protein
AQR21_RS25015	isochorismatase
AQR21_RS22390	cold-shock protein
AQR21_RS22385	hypothetical protein
AQR21_RS22380	hypothetical protein
AQR21_RS22375	hypothetical protein
AQR21_RS25720	hypothetical protein
AQR21_RS25715	hypothetical protein
AQR21_RS19730	hypothetical protein
RALGR_RS20185	hypothetical protein
RALGR_RS20195	hypothetical protein
AQR21_RS22695	hypothetical protein
RALGR_RS21075	RNase H superfamily protein

Moko typical	
**Gene**	**Annotation**
RALGR_RS26290	hypothetical protein
AQR21_RS16455	calcium-binding protein
RALGR_RS25405	HNH endonuclease
RALGR_RS25465	hypothetical protein
RALGR_RS26325	hypothetical protein
AQR21_RS03135	AAA family ATPase
AQR21_RS04315	amino acid permease
HXP35_07585	hypothetical protein
AQR21_RS22300	hypothetical protein
AQR21_RS22305	helix-turn-helix domain-containing protein
RALGR_RS23085	ImmA/IrrE family metallo-endopeptidase
RALGR_RS25560	tyrosine protein phosphatase
RALGR_RS23475	tyrosine-type recombinase/integrase
RALGR_RS26400	hypothetical protein
AQR21_RS25950	hypothetical protein
AQR21_RS00170	YOPP/AvrRxv family protein
AQR21_RS00180	hypothetical protein
RALGR_RS24050	AAA family ATPase
AQR21_RS17930	ParB/RepB/Spo0J family partition protein
AQR21_RS24845	RepA replicase
RALGR_RS24250	carboxypeptidase regulatory-like domain-containing protein
AQR21_RS11205	hypothetical protein
RALGR_RS24260	IS66 family transposase
C2I33_RS12790	hypothetical protein
AQR24_RS17400	hypothetical protein

Moko typical	
**Gene**	**Annotation**
AQR21_RS22470	ABC transporter permease subunit
EIH10_RS19540	hypothetical protein
AQR21_RS17935	LysR family transcriptional regulator
AQR21_RS25740	hypothetical protein
EIH10_RS23345	hypothetical protein
EIH10_RS04130	type III effector protein
EIH12_RS24145	hypothetical protein
EIH10_RS13090	type III effector protein skwp2
EIH12_RS24015	hypothetical protein
EIH10_RS23460	hypothetical protein
EIH10_RS15690	hypothetical protein
EIH12_RS18345	helix-turn-helix domain-containing protein
EIH10_RS15700	hypothetical protein
EIH10_RS15705	DUF2274 domain-containing protein
EIH10_RS15710	TrbI/VirB10 family protein
group_7806	P-type conjugative transfer protein TrbG
EIH10_RS15720	conjugal transfer protein TrbF
group_7808	P-type conjugative transfer protein TrbL
group_7809	P-type conjugative transfer protein TrbJ
EIH12_RS24075	hypothetical protein
EIH12_RS15165	DUF1484 domain-containing protein
EIH12_RS19680	S-layer family protein
EIH12_RS22105	transposase
EIH12_RS21445	phage major capsid protein
EIH10_RS23545	hypothetical protein

Moko typical	
**Gene**	**Annotation**
EIH10_RS19795	hypothetical protein
EIH10_RS21740	phage minor tail protein L
EIH12_RS12020	hypothetical protein
EIH10_RS22540	hypothetical protein
EIH12_RS18290	AlpA family phage regulatory protein
EIH12_RS18295	hypothetical protein
EIH14_RS01105	hypothetical protein
EIH11_RS05450	DUF1911 domain-containing protein
EIH11_RS05475	hypothetical protein
EIH14_RS01160	DUF769 domain-containing protein
EIH11_RS05495	hypothetical protein
EIH14_RS01175	hypothetical protein
EIH14_RS01330	Hpt domain-containing protein
EIH14_RS01425	hypothetical protein
EIH14_RS01580	hypothetical protein
HXP34_20520	Type III effector protein (Skwp 4)
EIH11_RS11215	SDR family NAD(P)-dependent oxidoreductase
EIH11_RS16445	hypothetical protein
EIH11_RS16375	hypothetical protein
EIH14_RS04655	hypothetical protein
EIH14_RS04685	hypothetical protein
EIH11_RS22230	DDE-type integrase/transposase/recombinase
EIH14_RS05765	hypothetical protein
EIH11_RS14545	GALA protein
EIH14_RS06140	hypothetical protein

Moko typical	
**Gene**	**Annotation**
EIH14_RS06365	lysis protein
EIH14_RS06370	hypothetical protein
EIH11_RS13925	hypothetical protein
EIH11_RS13840	hypothetical protein
EIH14_RS06500	hypothetical protein
EIH11_RS13830	type II toxin-antitoxin system MqsA family antitoxin
EIH11_RS17830	recombinase family protein
EIH11_RS17825	DUF2726 domain-containing protein
EIH14_RS07715	hypothetical protein
EIH14_RS23135	hypothetical protein
EIH14_RS07720	hypothetical protein
EIH14_RS07725	RNA-directed DNA polymerase
EIH14_RS07730	hypothetical protein
EIH11_RS01790	GALA protein
EIH14_RS08315	SDR family oxidoreductase
EIH14_RS08380	hypothetical protein
EIH14_RS23160	hypothetical protein
EIH11_RS02675	replication initiation protein
EIH14_RS08840	hypothetical protein
EIH14_RS08845	hypothetical protein
EIH14_RS08855	hypothetical protein
EIH11_RS02700	hypothetical protein
EIH14_RS08865	hypothetical protein
EIH11_RS02710	hypothetical protein
EIH14_RS08875	hypothetical protein

Moko typical	
**Gene**	**Annotation**
EIH11_RS02720	hypothetical protein
EIH14_RS08885	hypothetical protein
EIH11_RS02975	MFS transporter
EIH11_RS03180	hypothetical protein
HXP35_20670	MCE family protein
EIH11_RS03355	hypothetical protein
group_7901	adenosylmethionine decarboxylase
EIH11_RS04075	hypothetical protein
EIH11_RS23160	hypothetical protein
EIH11_RS06475	hypothetical protein
EIH14_RS12075	DNA (cytosine-5-)-methyltransferase
EIH11_RS06465	Eco29kI family restriction endonuclease
vsr	DNA mismatch endonuclease Vsr
EIH11_RS06455	ATP-binding protein
EIH14_RS12095	hypothetical protein
EIH11_RS06445	hypothetical protein
EIH11_RS06440	hypothetical protein
EIH14_RS12110	DNA-binding protein
EIH14_RS12115	HTH domain-containing protein
EIH11_RS06425	hypothetical protein
EIH14_RS12125	site-specific integrase
EIH14_RS12525	hypothetical protein
EIH14_RS13740	hypothetical protein
EIH11_RS10310	DUF2188 domain-containing protein
EIH14_RS14560	hypothetical protein

Moko typical	
**Gene**	**Annotation**
EIH14_RS14565	hypothetical protein
EIH11_RS10295	helix-turn-helix transcriptional regulator
EIH14_RS14575	ImmA/IrrE family metallo-endopeptidase
EIH14_RS14580	DUF262 domain-containing protein
EIH11_RS10280	hypothetical protein
EIH14_RS14590	hypothetical protein
EIH14_RS14595	DUF2924 domain-containing protein
EIH14_RS14600	recombinase family protein
EIH14_RS14605	hypothetical protein
EIH14_RS14610	helix-turn-helix domain-containing protein
EIH11_RS10250	hypothetical protein
EIH14_RS14620	hypothetical protein
EIH11_RS10240	hypothetical protein
EIH14_RS14630	ATP-binding protein
EIH11_RS10230	hypothetical protein
EIH11_RS10225	hypothetical protein
EIH14_RS14645	hypothetical protein
EIH14_RS14650	helix-turn-helix domain-containing protein
EIH11_RS10210	hypothetical protein
EIH11_RS10205	hypothetical protein
EIH14_RS14665	hypothetical protein
EIH11_RS10195	helix-turn-helix domain-containing protein
EIH14_RS14675	site-specific DNA-methyltransferase
EIH14_RS14680	site-specific DNA-methyltransferase
EIH14_RS14685	hypothetical protein

Moko typical	
**Gene**	**Annotation**
EIH14_RS14690	hypothetical protein
EIH11_RS10170	DUF3489 domain-containing protein
EIH11_RS10165	elements of external origin
EIH11_RS10155	hypothetical protein
EIH14_RS14715	phage portal protein
EIH11_RS10145	S49 family peptidase
EIH14_RS14725	head decoration protein
EIH11_RS10135	major capsid protein
EIH11_RS10130	hypothetical protein
EIH14_RS14740	hypothetical protein
EIH14_RS14745	hypothetical protein
EIH14_RS14750	hypothetical protein
EIH11_RS10110	hypothetical protein
EIH14_RS14760	hypothetical protein
EIH11_RS10100	hypothetical protein
EIH11_RS10095	phage tail tape measure protein
EIH14_RS14775	hypothetical protein
EIH11_RS10085	hypothetical protein
EIH11_RS10080	hypothetical protein
EIH14_RS14790	hypothetical protein
EIH14_RS14795	hypothetical protein
EIH11_RS10065	hypothetical protein
EIH11_RS10060	hypothetical protein
EIH14_RS14810	hypothetical protein
EIH14_RS14815	hypothetical protein

Moko typical	
**Gene**	**Annotation**
EIH14_RS14820	lysozyme
EIH11_RS10040	hypothetical protein
EIH14_RS14830	helix-turn-helix transcriptional regulator
EIH11_RS10030	phage Gp37/Gp68 family protein
tcmP	three-Cys-motif partner protein TcmP
EIH14_RS14845	toll/interleukin-1 receptor domain-containing protein
EIH14_RS23210	hypothetical protein
EIH14_RS14850	hypothetical protein
EIH11_RS10010	DEAD/DEAH box helicase family protein
EIH11_RS10005	hypothetical protein
EIH11_RS10000	DUF1156 domain-containing protein
EIH14_RS14870	hypothetical protein
EIH14_RS14875	DUF499 domain-containing protein
EIH14_RS14880	helix-turn-helix domain-containing protein
EIH14_RS14885	hypothetical protein
EIH14_RS14890	ParB N-terminal domain-containing protein
EIH14_RS14895	ParB/RepB/Spo0J family partition protein
EIH14_RS14900	recombinase family protein
EIH11_RS09960	hypothetical protein
EIH14_RS14910	transcriptional regulator
EIH11_RS09950	nucleotidyl transferase AbiEii/AbiGii toxin family protein
EIH14_RS14920	hypothetical protein
EIH14_RS14925	hypothetical protein
EIH14_RS14930	hypothetical protein
EIH14_RS14940	hypothetical protein

Moko typical	
**Gene**	**Annotation**
EIH14_RS14945	hypothetical protein
EIH14_RS14950	site-specific recombinase resolvase
EIH11_RS09865	type II toxin-antitoxin system RelE/ParE family toxin
EIH11_RS09860	hypothetical protein
EIH14_RS15010	DUF1484 family protein
EIH14_RS15015	DUF3489 domain-containing protein
EIH14_RS15545	hypothetical protein
EIH11_RS07610	DNA adenine methylase
EIH11_RS07855	hypothetical protein
EIH14_RS16335	hypothetical protein
EIH14_RS16450	hypothetical protein
EIH11_RS00765	hypothetical protein
EIH14_RS17665	hypothetical protein
EIH14_RS17675	TonB-dependent siderophore receptor
EIH11_RS00205	hypothetical protein
EIH14_RS18480	YncE family protein
EIH14_RS18545	autotransporter domain-containing protein
EIH11_RS09070	hypothetical protein
EIH14_RS20275	hypothetical protein
EIH14_RS20425	hypothetical protein
EIH11_RS20130	hypothetical protein
EIH14_RS22430	transcriptional repressor
EIH14_RS23295	hypothetical protein
EIH11_RS19440	hypothetical protein
EIH11_RS19435	hypothetical protein

Moko typical	
**Gene**	**Annotation**
EIH11_RS19420	hypothetical protein
EIH11_RS19375	PAAR domain-containing protein
HXP34_08800	PilZ domain-containing protein
EIH11_RS22715	ATP-binding protein
EIH14_RS22935	hypothetical protein
EIH14_RS22940	hypothetical protein
RSPO_RS07370	AAA family ATPase
RSPO_RS17895	AAA family ATPase
UW163_RS18310	AAA family ATPase
UW163_RS20520	AAA family ATPase
RSPO_RS22290	AAA family ATPase
EIH14_RS22805	AAA family ATPase
UW163_RS15585	IS3 family transposase
RALW1_RS14225	IS3 family transposase
RSPO_RS24680	IS5 family transposase
UW163_RS25250	IS5 family transposase
UW163_RS15600	IS21 family transposase
UW163_RS15235	IS21 family transposase
RSPO_RS05350	IS21 family transposase
RSPO_RS17890	IS21 family transposase
UW163_RS18305	IS21 family transposase
RSPO_RS20245	IS21 family transposase
RSPO_RS22285	IS21 family transposase
EIH10_RS11755	hypothetical protein
C2I33_RS08810	membrane protein

Moko typical	
**Gene**	**Annotation**
RALW1_RS16860	MMPL family transporter
AQR24_RS09230	type I secretion system permease/ATPase
AQR24_RS26090	DUF3757 domain-containing protein
RALB5_RS00235	AMP-binding protein
HXP37_11360	IS5 family transposase
HXP37_22690	IS5 family transposase
RALFB_RS27365	DUF2924 domain-containing protein
RALFB_RS27225	DUF2924 domain-containing protein
UW163_RS05335	phage terminase large subunit family protein
RALFB_RS22685	phage terminase large subunit family protein
group_1084	elongation factor Tu
RALGR_RS03935	acyl carrier protein
C2I33_RS25935	AMP-binding protein
AQR21_RS23765	transposase
C2I33_RS25715	RHS repeat protein
AQR24_RS10625	amino acid adenylation domain-containing protein
EIH10_RS14260	hypothetical protein
RALFB_RS27490	hypothetical protein
UW163_RS00060	S49 family peptidase
C2I33_RS25980	AMP-binding protein
RALFB_RS18265	terminase ATPase subunit family protein
EIH10_RS04235	hypothetical protein
EIH10_RS17435	IS5-like element IS1420 family transposase
EIH10_RS23605	hypothetical protein
RALGR_RS19375	DUF3293 domain-containing protein

Moko typical	
**Gene**	**Annotation**
C2I33_RS15610	iron chelate uptake ABC transporter family permease subunit
RALW1_RS00220	AMP-binding protein
HXP34_21480	hypothetical protein
HXP36_23890	MCP four helix bundle domain-containing protein
HXP36_23885	GGDEF domain-containing protein
HXP36_23880	response regulator transcription factor
HXP36_23860	peroxidase-related enzyme
HXP36_23855	acyl-CoA thioesterase/BAAT N-terminal domain-containing protein
HXP36_23835	ABC transporter permease
HXP36_23825	NAD(P)/FAD-dependent oxidoreductase
HXP36_23625	universal stress protein
HXP36_23620	zinc-dependent alcohol dehydrogenase family protein
group_1339	hydroxyacylglutathione hydrolase
HXP36_23450	sulfite exporter TauE/SafE family protein
HXP34_20505	TetR/AcrR family transcriptional regulator
HXP36_23390	TetR/AcrR family transcriptional regulator
HXP35_20665	TetR/AcrR family transcriptional regulator
HXP36_23370	metallophosphoesterase
HXP34_20485	metallophosphoesterase
HXP35_20645	metallophosphoesterase
HXP35_20635	efflux RND transporter periplasmic adaptor subunit
HXP34_20475	efflux RND transporter periplasmic adaptor subunit
HXP36_23360	efflux RND transporter periplasmic adaptor subunit
HXP36_23350	TetR/AcrR family transcriptional regulator
RALW1_RS26445	AMP-binding protein

Moko typical	
**Gene**	**Annotation**
C2I33_RS25930	transposase
RALW1_RS24770	hypothetical protein
RALW1_RS26440	glycoside hydrolase family protein
EIH10_RS19500	lysozyme
group_162	type VI secretion system tip protein VgrG
RALB5_RS25615	hypothetical protein
RALGR_RS21175	EAL domain-containing protein
C2I33_RS19460	hypothetical protein
group_1763	type VI secretion system tip protein VgrG
HXP36_23895	hypothetical protein
HXP36_23660	DUF3761 domain-containing protein
HXP36_23645	tyrosinase family protein
RALFB_RS25370	MFS transporter
HXP36_23635	SCO family protein
HXP36_14385	DUF1016 domain-containing protein
UW163_RS00040	hypothetical protein
RALW1_RS23815	hypothetical protein
AQR21_RS00045	AMP-binding protein
HXP36_14055	hypothetical protein
UW163_RS00050	major capsid protein
RALW1_RS26390	hypothetical protein
RALGR_RS21920	RNA ligase family protein
UW163_RS16205	membrane protein
RALB5_RS00275	AMP-binding protein
EIH10_RS23290	hypothetical protein

Moko typical	
**Gene**	**Annotation**
EIH10_RS23295	hypothetical protein
RALB5_RS22750	MBL fold metallo-hydrolase
AQR24_RS25985	IS701 family transposase
EIH10_RS19450	hypothetical protein
EIH10_RS23315	hypothetical protein
RALGR_RS11805	acyltransferase domain-containing protein
EIH10_RS23030	hypothetical protein
EIH12_RS15565	DUF3304 domain-containing protein
EIH12_RS23315	hypothetical protein
EIH10_RS19205	hypothetical protein
AQR21_RS23810	transposase
AQR21_RS00260	transposase
AQR24_RS00205	IS5 family transposase
UW163_RS26160	hypothetical protein
UW163_RS26220	hypothetical protein
UW163_RS26165	hypothetical protein
EIH10_RS22555	hypothetical protein
C2I33_RS25495	ShlB/FhaC/HecB family hemolysin secretion/activation protein
RALB5_RS22780	WGR domain-containing protein
RALB5_RS21505	DUF4011 domain-containing protein
C2I33_RS21725	acyl carrier protein
RALGR_RS23740	Bcr/CflA family efflux MFS transporter
AQR24_RS24975	DUF2875 family protein
RALGR_RS25655	hypothetical protein
RALGR_RS25640	hypothetical protein

Moko typical	
**Gene**	**Annotation**
RALGR_RS25635	hypothetical protein
RALB5_RS19010	IS3 family transposase
AQR21_RS19910	DUF2063 domain-containing protein
HXP34_21345	GntR family transcriptional regulator
group_2429	tRNA pseudouridine(38-40) synthase TruA
EIH10_RS06340	DUF2063 domain-containing protein
HXP34_21415	phosphoribosylanthranilate isomerase
group_2433	hemin uptake protein HemP
HXP36_23815	ABC transporter substrate-binding protein
HXP36_23800	hypothetical protein
HXP36_23790	DUF4743 domain-containing protein
group_2447	DNA polymerase III subunit epsilon
HXP36_23615	DUF2892 domain-containing protein
HXP36_01875	putative DNA-binding domain-containing protein
HXP36_23590	class I SAM-dependent methyltransferase
group_2453	glutamine-hydrolyzing carbamoyl-phosphate synthase small subunit
HXP36_23550	DUF4149 domain-containing protein
group_2457	dihydropteroate synthase
group_2459	phosphate ABC transporter permease PstA
HXP36_23475	GNAT family N-acetyltransferase
group_2463	phosphohistidine phosphatase SixA
HXP36_23435	MBL fold metallo-hydrolase
HXP35_20660	hypothetical protein
EIH09_RS21775	hypothetical protein
HXP36_23385	hypothetical protein

Moko typical	
**Gene**	**Annotation**
HXP34_20500	hypothetical protein
HXP34_20480	efflux RND transporter permease subunit
HXP35_20640	efflux RND transporter permease subunit
HXP36_23365	efflux RND transporter permease subunit
RALW1_RS00145	hypothetical protein
RALW1_RS00210	H-NS histone family protein
RALW1_RS24255	IS3 family transposase
RALB5_RS24475	DUF748 domain-containing protein
EIH12_RS17115	IS5 family transposase
RALB5_RS28135	hypothetical protein
RALB5_RS27025	DUF2345 domain-containing protein
EIH12_RS01150	transposase
C2I33_RS25535	porin
HXP36_17815	hypothetical protein
RALFB_RS28355	AMP-binding protein
EIH10_RS10520	IS3 family transposase
AQR24_RS17765	KR domain-containing protein
EIH10_RS13035	transposase
AQR24_RS24000	methyltransferase
EIH10_RS22385	transposase
RALW1_RS00190	hypothetical protein
HXP36_02325	phage portal protein
HXP36_02415	phage portal protein
AQR21_RS25200	LysR family transcriptional regulator
group_3279	bifunctional tetrahydrofolate synthase/dihydrofolate synthase

Moko typical	
**Gene**	**Annotation**
HXP36_23910	GNAT family N-acetyltransferase
HXP36_23900	helix-turn-helix transcriptional regulator
HXP36_23870	(2Fe-2S)-binding protein
HXP36_23640	hypothetical protein
HXP36_23460	acyltransferase
HXP34_07415	IS3 family transposase
HXP36_09660	RHS repeat protein
HXP36_23200	hypothetical protein
EIH10_RS07585	transposase
EIH10_RS18570	transposase
RALB5_RS27140	DUF1983 domain-containing protein
RALB5_RS27990	DUF1983 domain-containing protein
EIH10_RS22020	IS5 family transposase
UW163_RS16220	hypothetical protein
UW163_RS16200	hypothetical protein
RALGR_RS23445	NAD(P)-binding protein
AQR21_RS23415	hypothetical protein
EIH10_RS08285	transposase
UW163_RS25440	hypothetical protein
UW163_RS26150	hypothetical protein
RALB5_RS00175	aminotransferase class I/II-fold pyridoxal phosphate-dependent enzyme
EIH10_RS23625	transposase
AQR24_RS06835	RraA family protein
RALB5_RS07240	RraA family protein
EIH10_RS23560	hypothetical protein

Moko typical	
**Gene**	**Annotation**
UW163_RS26170	hypothetical protein
UW163_RS00030	hypothetical protein
HXP36_02360	hypothetical protein
RALW1_RS23880	hypothetical protein
UW163_RS24250	hypothetical protein
RALW1_RS23730	hypothetical protein
RALB5_RS25815	hypothetical protein
RALFB_RS28375	AMP-binding protein
RALGR_RS25245	IS5 family transposase
RALB5_RS21735	phospholipase
C2I33_RS19435	hypothetical protein
RALGR_RS24275	hypothetical protein
AQR24_RS00315	filamentous hemagglutinin N-terminal domain-containing protein
HXP34_21405	FimV family protein
HXP35_20760	type VI secretion system tip protein VgrG
HXP34_21335	CoA ester lyase
HXP34_21395	hypothetical protein
HXP34_21445	SPOR domain-containing protein
HXP34_07150	energy transducer TonB
AQR24_RS26465	hypothetical protein
group_4208	leucine efflux protein LeuE
RALB5_RS26715	hypothetical protein
HXP36_23400	DUF3313 domain-containing protein
HXP36_23375	hypothetical protein
HXP35_20650	hypothetical protein

Moko typical	
**Gene**	**Annotation**
HXP34_20490	hypothetical protein
HXP34_20470	hypothetical protein
AQR24_RS15900	O-acetylserine/cysteine exporter
HXP34_15600	hypothetical protein
HXP36_21890	(2Fe-2S)-binding protein
EIH09_RS21765	hypothetical protein
EIH10_RS19825	filamentous hemagglutinin N-terminal domain-containing protein
UW163_RS00055	head decoration protein
HXP36_02335	head decoration protein
HXP34_16140	ShlB/FhaC/HecB family hemolysin secretion/activation protein
RALFB_RS23960	cytochrome C
EIH10_RS19815	filamentous hemagglutinin N-terminal domain-containing protein
RALW1_RS22685	ATP-binding cassette domain-containing protein
RALGR_RS00140	filamentous hemagglutinin N-terminal domain-containing protein
RALGR_RS22715	PAS domain-containing protein
HXP36_00070	hypothetical protein
HXP36_00090	zinc-ribbon domain-containing protein
HXP36_00125	TonB-dependent receptor
HXP36_00130	FecR domain-containing protein
HXP36_00135	RNA polymerase sigma factor
HXP36_00145	SLATT domain-containing protein
HXP36_00150	nucleotidyltransferase
HXP36_00155	DUF3800 domain-containing protein
HXP36_00160	YkgJ family cysteine cluster protein
HXP36_00165	hypothetical protein

Moko typical	
**Gene**	**Annotation**
HXP36_00170	restriction endonuclease
HXP36_00175	helix-turn-helix transcriptional regulator
HXP36_00265	RHS repeat protein
HXP36_00275	immunity protein 58
HXP36_00280	hypothetical protein
HXP36_00355	signal peptidase
HXP36_00455	RNA polymerase subunit sigma-70
HXP36_00520	hypothetical protein
HXP36_00570	hypothetical protein
HXP36_01120	SAM-dependent DNA methyltransferase
HXP36_01125	restriction endonuclease subunit S
HXP36_01130	DUF262 domain-containing protein
HXP36_01135	DEAD/DEAH box helicase family protein
HXP36_01140	hypothetical protein
HXP36_01145	helix-turn-helix transcriptional regulator
HXP36_01150	ImmA/IrrE family metallo-endopeptidase
HXP36_01155	C1 family peptidase
HXP36_01160	hypothetical protein
HXP36_01165	uracil-DNA glycosylase
HXP36_01175	nucleoside triphosphate pyrophosphohydrolase family protein
HXP36_01180	DUF4031 domain-containing protein
HXP36_01185	helix-turn-helix transcriptional regulator
HXP36_01190	hypothetical protein
HXP36_01230	PAAR domain-containing protein
HXP36_01240	hypothetical protein

Moko typical	
**Gene**	**Annotation**
HXP36_01255	hypothetical protein
HXP36_01260	hypothetical protein
HXP36_01265	DUF695 domain-containing protein
HXP36_01285	restriction endonuclease
HXP36_01670	HD domain-containing protein
HXP36_02125	TIGR04255 family protein
HXP36_02130	hypothetical protein
HXP36_02135	hypothetical protein
HXP36_02140	restriction endonuclease
HXP36_02145	hypothetical protein
HXP36_02250	site-specific DNA-methyltransferase
HXP36_02255	site-specific DNA-methyltransferase
HXP36_02260	hypothetical protein
HXP36_02265	hypothetical protein
HXP36_02285	DUF3489 domain-containing protein
HXP36_02290	hypothetical protein
HXP36_02315	hypothetical protein
HXP36_02525	hypothetical protein
HXP36_02535	SAM-dependent methyltransferase
HXP36_02540	hypothetical protein
HXP36_02565	hypothetical protein
HXP36_02570	hypothetical protein
HXP36_02580	hypothetical protein
HXP36_02585	hypothetical protein
HXP36_02590	DUF1851 domain-containing protein

Moko typical	
**Gene**	**Annotation**
HXP36_02605	nucleotide-binding protein
HXP36_02610	hypothetical protein
HXP36_02630	RHS repeat protein
HXP36_02635	RHS repeat protein
HXP36_02645	hypothetical protein
HXP36_02650	hypothetical protein
HXP36_02655	hypothetical protein
HXP36_02660	hypothetical protein
HXP36_02685	RICIN domain-containing protein
HXP36_02800	hypothetical protein
HXP36_02810	type III effector
HXP36_02815	hypothetical protein
HXP36_02965	preprotein translocase subunit SecF
HXP36_02970	hypothetical protein
HXP36_03155	hypothetical protein
HXP36_04045	integrase arm-type DNA-binding domain-containing protein
HXP36_04050	hypothetical protein
HXP36_04055	AlpA family phage regulatory protein
HXP36_04060	AAA family ATPase
HXP36_04065	hypothetical protein
HXP36_04070	inovirus-type Gp2 protein
HXP36_04075	AAA family ATPase
HXP36_04080	ATP-dependent helicase
HXP36_04085	DEAD/DEAH box helicase family protein
HXP36_04090	site-specific DNA-methyltransferase

Moko typical	
**Gene**	**Annotation**
HXP36_04095	PHA-granule associated protein 4
HXP36_04100	HU family DNA-binding protein
HXP36_04105	hypothetical protein
HXP36_04110	DUF932 domain-containing protein
HXP36_04115	YqaJ viral recombinase family protein
HXP36_04120	phage capsid protein
HXP36_04125	hypothetical protein
HXP36_04130	hypothetical protein
HXP36_04135	glycine zipper 2TM domain-containing protein
HXP36_04140	plasmid related protein
C2I33_RS05230	group II truncated hemoglobin
HXP36_04145	helix-turn-helix transcriptional regulator
HXP36_04150	hypothetical protein
HXP36_04155	cell wall anchor protein
HXP36_04160	toxin-antitoxin system YwqK family antitoxin
HXP36_04165	site-specific integrase
HXP36_04170	hypothetical protein
HXP36_04175	AAA family ATPase
HXP36_04180	hypothetical protein
HXP36_04185	hypothetical protein
HXP36_04190	hypothetical protein
HXP36_04195	type VI secretion system tip protein VgrG
HXP36_04200	hypothetical protein
HXP36_04205	DUF1911 domain-containing protein
HXP36_05305	chromate resistance protein

Moko typical	
**Gene**	**Annotation**
HXP36_05350	AAA family ATPase
HXP36_05820	chalcone isomerase family protein
HXP36_06060	MliC family protein
HXP36_06095	hypothetical protein
HXP36_06560	DUF3306 domain-containing protein
HXP36_06775	DUF3987 domain-containing protein
HXP34_21475	phasin family protein
HXP36_06805	flavin reductase
HXP34_21485	hypothetical protein
HXP36_06955	hypothetical protein
HXP36_06980	type II secretion system protein
HXP36_06985	type II secretion system protein
HXP36_06990	type II secretion system protein
HXP36_06995	multicopper oxidase family protein
HXP36_07000	multicopper oxidase family protein
HXP36_07010	hypothetical protein
HXP36_07015	outer membrane lipoprotein-sorting protein
HXP36_07020	hypothetical protein
HXP36_07035	lytic transglycosylase domain-containing protein
HXP36_07380	hsdR
HXP36_07385	hypothetical protein
HXP36_07390	hypothetical protein
HXP36_07395	hypothetical protein
HXP36_07400	hypothetical protein
HXP36_23865	xanthine dehydrogenase family protein molybdopterin-binding subunit

Moko typical	
**Gene**	**Annotation**
HXP36_07405	hypothetical protein
HXP36_07410	hypothetical protein
HXP36_07505	hypothetical protein
HXP36_07685	hypothetical protein
HXP36_07690	hypothetical protein
HXP36_07695	hypothetical protein
HXP36_07715	AlpA family phage regulatory protein
HXP36_07720	DUF1488 domain-containing protein
HXP36_07725	phosphoadenosine phosphosulfate reductase family protein
C2I33_RS25760	molybdopterin-dependent oxidoreductase
HXP36_07730	DNA cytosine methyltransferase
HXP36_07735	HD family hydrolase
HXP36_07760	hypothetical protein
HXP36_07765	hypothetical protein
HXP36_07770	hypothetical protein
HXP36_07790	hypothetical protein
HXP36_07795	hypothetical protein
HXP36_07805	hypothetical protein
HXP36_07810	hypothetical protein
HXP36_07815	hypothetical protein
HXP36_07820	HNH endonuclease
HXP36_07825	hypothetical protein
HXP36_07830	terminase large subunit
HXP36_07840	phage portal protein
HXP36_23845	ATP-binding cassette domain-containing protein

Moko typical	
**Gene**	**Annotation**
HXP36_07875	hypothetical protein
HXP36_07885	hypothetical protein
HXP36_07920	C40 family peptidase
HXP36_07945	glycoside hydrolase family protein
HXP36_07975	integrase arm-type DNA-binding domain-containing protein
HXP36_08120	hypothetical protein
HXP36_08610	hypothetical protein
HXP36_08615	AAA family ATPase
HXP36_08920	hypothetical protein
HXP36_08930	RHS repeat protein
HXP36_08935	RHS repeat protein
HXP36_08945	SMI1/KNR4 family protein
HXP36_23915	SMI1/KNR4 family protein
HXP36_08955	hypothetical protein
HXP36_23905	hypothetical protein
HXP36_23875	hypothetical protein
HXP36_09090	integrase arm-type DNA-binding domain-containing protein
HXP36_23770	integrase arm-type DNA-binding domain-containing protein
HXP36_09095	hypothetical protein
HXP36_23765	hypothetical protein
HXP36_09100	AlpA family phage regulatory protein
HXP36_23760	AlpA family phage regulatory protein
HXP36_09105	hypothetical protein
HXP36_23755	hypothetical protein
HXP36_09110	hypothetical protein

Moko typical	
**Gene**	**Annotation**
HXP36_23750	hypothetical protein
HXP36_09115	hypothetical protein
HXP36_23745	hypothetical protein
group_509	propionate--CoA ligase
HXP36_09120	hypothetical protein
HXP36_23740	hypothetical protein
HXP36_09125	hypothetical protein
HXP36_23735	hypothetical protein
HXP36_09140	hypothetical protein
HXP36_23720	hypothetical protein
HXP36_09145	hypothetical protein
HXP36_23715	hypothetical protein
HXP36_09150	phage tail protein
HXP36_23710	phage tail protein
HXP36_09155	hypothetical protein
HXP36_23705	hypothetical protein
HXP36_09160	hypothetical protein
HXP36_23700	hypothetical protein
HXP36_09165	hypothetical protein
HXP36_23695	hypothetical protein
HXP36_09170	hypothetical protein
HXP36_23690	hypothetical protein
HXP36_09175	hypothetical protein
HXP36_23685	hypothetical protein
HXP36_23380	DUF2145 domain-containing protein

Moko typical	
**Gene**	**Annotation**
HXP36_09180	hypothetical protein
HXP36_23680	hypothetical protein
HXP36_09185	hypothetical protein
HXP36_23675	hypothetical protein
HXP36_09190	hypothetical protein
HXP36_23670	hypothetical protein
HXP36_09195	DUF1488 family protein
HXP36_23665	DUF1488 family protein
HXP36_09205	hypothetical protein
HXP36_23655	hypothetical protein
HXP34_20495	DUF2145 domain-containing protein
HXP36_09210	excisionase
HXP36_23650	excisionase
HXP36_09230	hypothetical protein
HXP36_23630	hypothetical protein
HXP36_23455	hypothetical protein
HXP36_23425	aminotransferase class III-fold pyridoxal phosphate-dependent enzyme
HXP36_23420	cupin-like domain-containing protein
HXP35_20655	DUF2145 domain-containing protein
HXP36_23415	hypothetical protein
HXP36_09450	hypothetical protein
HXP36_23410	hypothetical protein
HXP36_09515	UDP-glucuronosyltransferase
HXP36_23345	UDP-glucuronosyltransferase
group_5136	flagellar type III secretion system protein FliQ

Moko typical	
**Gene**	**Annotation**
group_5137	flagellar type III secretion system protein FliQ
HXP36_09525	flagellar biosynthetic protein FliR
HXP36_23335	flagellar biosynthetic protein FliR
HXP36_09530	EscU/YscU/HrcU family type III secretion system export apparatus switch protein
HXP36_23330	EscU/YscU/HrcU family type III secretion system export apparatus switch protein
group_5142	flagellar biosynthesis protein FlhA
group_5143	flagellar biosynthesis protein FlhA
HXP36_09540	flagellar hook-basal body protein
HXP36_23320	flagellar hook-basal body protein
group_5146	flagellar basal-body rod protein FlgG
group_5147	flagellar basal-body rod protein FlgG
HXP36_09550	flagella basal body P-ring formation protein FlgA
HXP36_23310	flagella basal body P-ring formation protein FlgA
HXP36_09555	flagellar basal body L-ring protein FlgH
HXP36_23305	flagellar basal body L-ring protein FlgH
HXP36_09565	flagellar basal body protein
HXP36_23295	flagellar basal body protein
group_5154	flagellar basal body rod protein FlgC
group_5155	flagellar basal body rod protein FlgC
group_5156	flagellar hook-basal body complex protein FliE
group_5157	flagellar hook-basal body complex protein FliE
group_5158	flagellar M-ring protein FliF
group_5159	flagellar M-ring protein FliF
HXP36_09585	hypothetical protein
HXP36_23275	hypothetical protein

Moko typical	
**Gene**	**Annotation**
HXP36_09590	hypothetical protein
HXP36_23270	hypothetical protein
HXP36_09595	FliI/YscN family ATPase
HXP36_23265	FliI/YscN family ATPase
HXP36_09600	hypothetical protein
HXP36_23260	hypothetical protein
HXP36_09605	hypothetical protein
HXP36_23255	hypothetical protein
HXP36_09610	flagellar hook capping protein
HXP36_23250	flagellar hook capping protein
HXP36_09615	flagellar hook-basal body complex protein
HXP36_23245	flagellar hook-basal body complex protein
HXP36_09620	FliM/FliN family flagellar motor switch protein
HXP36_23240	FliM/FliN family flagellar motor switch protein
HXP36_09625	FliM/FliN family flagellar motor switch protein
HXP36_23235	FliM/FliN family flagellar motor switch protein
HXP36_09630	flagellar biosynthetic protein FliO
HXP36_23230	flagellar biosynthetic protein FliO
group_5180	flagellar type III secretion system pore protein FliP
group_5181	flagellar type III secretion system pore protein FliP
HXP36_09640	sigma-70 family RNA polymerase sigma factor
HXP36_23220	sigma-70 family RNA polymerase sigma factor
HXP36_09645	tetratricopeptide repeat protein
HXP36_23215	tetratricopeptide repeat protein
HXP36_09650	hypothetical protein

Moko typical	
**Gene**	**Annotation**
HXP36_23210	hypothetical protein
HXP36_09655	tetratricopeptide repeat protein
HXP36_23205	tetratricopeptide repeat protein
HXP36_09665	hypothetical protein
HXP36_09670	hypothetical protein
HXP36_09675	hypothetical protein
HXP36_09680	hypothetical protein
HXP36_09685	IS4 family transposase
HXP36_09690	hypothetical protein
HXP36_09695	hypothetical protein
HXP36_09700	DUF2846 domain-containing protein
HXP36_09710	hypothetical protein
HXP36_09715	2OG-Fe(II) oxygenase
HXP36_09720	hypothetical protein
HXP36_09725	YcaO-like family protein
HXP36_10455	septation protein A
HXP36_10740	ISNCY family transposase
HXP36_10745	hypothetical protein
HXP36_10750	glycosyltransferase
HXP36_10755	hypothetical protein
HXP36_10760	hypothetical protein
HXP36_10765	hypothetical protein
HXP36_10770	hypothetical protein
HXP36_10775	Mov34/MPN/PAD-1 family protein
HXP36_10780	ThiF family adenylyltransferase

Moko typical	
**Gene**	**Annotation**
HXP36_10785	WYL domain-containing protein
HXP36_10790	hypothetical protein
HXP36_10795	hypothetical protein
HXP36_10800	hypothetical protein
HXP36_10805	hypothetical protein
HXP36_10810	hypothetical protein
HXP36_10815	hypothetical protein
HXP36_10820	hypothetical protein
HXP36_10825	site-specific integrase
HXP36_10830	tyrosine-type recombinase/integrase
HXP36_10840	hypothetical protein
HXP36_10850	PLP-dependent aminotransferase family protein
group_5238	ubiquinol oxidase subunit II
group_5239	cytochrome o ubiquinol oxidase subunit I
group_5240	cytochrome o ubiquinol oxidase subunit III
cyoD	cytochrome o ubiquinol oxidase subunit IV
HXP36_10875	nitrite/sulfite reductase
HXP36_10880	DUF934 domain-containing protein
HXP36_10995	type III effector protein
HXP36_11555	GALA protein
HXP36_11560	GALA protein
HXP36_11835	mannose-6-phosphate isomerase
HXP36_12690	hypothetical protein
HXP36_12920	hypothetical protein
HXP36_12925	hypothetical protein

Moko typical	
**Gene**	**Annotation**
HXP36_13590	tyrosine-type recombinase/integrase
HXP36_13605	hypothetical protein
HXP36_13610	ogr/Delta-like zinc finger family protein
HXP36_13615	hypothetical protein
HXP36_13620	hypothetical protein
HXP36_13630	hypothetical protein
HXP36_13635	hypothetical protein
HXP36_13640	TIGR04255 family protein
HXP36_13945	recombinase family protein
HXP36_14015	class I SAM-dependent methyltransferase
HXP36_14020	NAD(P)/FAD-dependent oxidoreductase
HXP36_14025	TetR/AcrR family transcriptional regulator
HXP36_14035	hypothetical protein
HXP36_14070	hypothetical protein
HXP36_14080	DUF1484 domain-containing protein
UW163_RS16190	hypothetical protein
HXP36_14090	hypothetical protein
group_5360	group II intron reverse transcriptase/maturase
HXP36_14200	hypothetical protein
HXP36_14205	hypothetical protein
HXP36_14210	hypothetical protein
HXP36_14215	glycine hydroxymethyltransferase
HXP36_14265	hypothetical protein
HXP36_14640	acyl-CoA dehydrogenase family protein
RALGR_RS24280	DUF3999 family protein

Moko typical	
**Gene**	**Annotation**
HXP36_15145	hypothetical protein
HXP36_15180	transposase
HXP36_15200	hypothetical protein
HXP36_15520	pilin
HXP36_15700	hypothetical protein
HXP36_15945	tartrate dehydrogenase
HXP36_16275	hypothetical protein
HXP36_16785	hypothetical protein
HXP36_16800	hypothetical protein
HXP36_16895	hypothetical protein
HXP36_17175	hypothetical protein
HXP36_17180	hypothetical protein
HXP36_17185	calcium-binding protein
HXP36_17595	hypothetical protein
HXP36_17625	signal peptidase
HXP36_17715	hypothetical protein
HXP36_17765	hypothetical protein
HXP36_17835	thioredoxin family protein
HXP36_17845	hypothetical protein
HXP36_17860	response regulator
HXP36_17905	hypothetical protein
HXP36_17915	ShET2/EspL2 family type III secretion system effector toxin
HXP36_17955	type III effector protein
HXP36_17980	hypothetical protein
HXP36_18075	hypothetical protein

Moko typical	
**Gene**	**Annotation**
HXP36_18220	hypothetical protein
HXP36_18230	hypothetical protein
HXP36_18235	hypothetical protein
HXP36_18240	tetratricopeptide repeat protein
HXP36_18250	hypothetical protein
HXP36_18255	hypothetical protein
HXP36_18260	hypothetical protein
HXP36_18265	hypothetical protein
HXP36_18270	replication initiation protein
HXP36_18275	hypothetical protein
HXP36_18395	hypothetical protein
HXP36_18440	hypothetical protein
HXP36_18530	hypothetical protein
HXP36_18695	hypothetical protein
HXP36_18700	HD domain-containing protein
HXP36_18705	hypothetical protein
HXP36_18710	helix-turn-helix transcriptional regulator
HXP36_18715	glyoxalase/bleomycin resistance/dioxygenase family protein
HXP36_18720	arsenate reductase ArsC
HXP36_18725	aquaporin family protein
HXP36_18770	hypothetical protein
HXP36_18775	hypothetical protein
HXP36_18780	hypothetical protein
HXP36_18785	hypothetical protein
HXP36_18790	hypothetical protein

Moko typical	
**Gene**	**Annotation**
HXP36_18795	hypothetical protein
HXP36_18800	DUF2314 domain-containing protein
HXP36_18805	immunity 22 family protein
HXP36_18810	hypothetical protein
HXP36_18840	hypothetical protein
HXP36_18845	hypothetical protein
HXP36_18860	hypothetical protein
HXP36_18875	fucose-binding lectin protein
HXP36_18910	hypothetical protein
HXP36_18915	hypothetical protein
HXP36_18925	hypothetical protein
HXP36_18930	KAP family NTPase
HXP36_18965	TolC family protein
HXP36_18970	TetR/AcrR family transcriptional regulator
HXP36_18975	LysR family transcriptional regulator
HXP36_18980	phosphonopyruvate decarboxylase
HXP36_18985	aldehyde dehydrogenase
HXP36_18990	aldehyde dehydrogenase
RALW1_RS22110	filamentous hemagglutinin
HXP36_19005	CoA transferase
HXP36_19010	CoA ester lyase
HXP36_19015	type III effector protein
HXP36_19020	MaoC family dehydratase N-terminal domain-containing protein
HXP36_19030	hypothetical protein
HXP36_19055	hypothetical protein

Moko typical	
**Gene**	**Annotation**
HXP36_19060	hypothetical protein
HXP36_19070	EndoU domain-containing protein
HXP36_19075	hypothetical protein
HXP36_19080	hypothetical protein
HXP36_19085	hypothetical protein
HXP36_19095	hypothetical protein
HXP36_19100	hypothetical protein
HXP36_19125	YafY family transcriptional regulator
HXP36_19130	hypothetical protein
HXP36_19180	hypothetical protein
HXP36_19200	hypothetical protein
HXP36_19205	S8 family serine peptidase
HXP36_19235	type III effector
HXP36_19250	hypothetical protein
HXP36_19260	hypothetical protein
HXP36_19815	MFS transporter
HXP36_19870	DUF1911 domain-containing protein
HXP36_19875	hypothetical protein
HXP36_19880	type VI secretion system tip protein VgrG
HXP36_19950	hypothetical protein
HXP36_19955	hypothetical protein
HXP36_19965	hypothetical protein
HXP36_20235	hypothetical protein
HXP36_20510	leucine-rich repeat protein
HXP36_20520	hypothetical protein

Moko typical	
**Gene**	**Annotation**
HXP36_20530	hypothetical protein
HXP36_20550	nuclear transport factor 2 family protein
HXP36_20565	type III effector protein
HXP36_20595	type III effector protein
HXP36_20630	hypothetical protein
HXP36_20820	hypothetical protein
HXP36_20845	hypothetical protein
HXP36_20875	hypothetical protein
HXP36_20880	helix-turn-helix transcriptional regulator
HXP36_21225	type III effector protein
RALW1_RS25670	filamentous hemagglutinin N-terminal domain-containing protein
HXP36_21495	type III effector protein
HXP36_21570	hypothetical protein
HXP36_21575	hypothetical protein
HXP36_21585	hypothetical protein
HXP36_21595	DUF596 domain-containing protein
HXP36_21615	hypothetical protein
HXP36_21620	hypothetical protein
HXP36_21895	xanthine dehydrogenase family protein molybdopterin-binding subunit
HXP36_22015	DUF3175 domain-containing protein
HXP36_22030	BapA prefix-like domain-containing protein
HXP36_22145	oligosaccharide repeat unit polymerase
HXP36_22200	type III effector protein
HXP36_22230	hypothetical protein
HXP36_22235	hypothetical protein

Moko typical	
**Gene**	**Annotation**
HXP36_22240	hypothetical protein
HXP36_22245	DUF1911 domain-containing protein
HXP36_22355	type III effector protein
HXP36_22765	F-box domain-containing protein
HXP36_22775	transposase
HXP36_22780	hypothetical protein
HXP36_22785	hypothetical protein
HXP36_22815	type III effector protein
HXP36_22880	DNA-binding protein
HXP36_23090	hypothetical protein
HXP36_23095	hypothetical protein
HXP36_23110	type III effector protein
HXP36_23115	hypothetical protein
HXP36_23160	hypothetical protein
HXP36_23190	hypothetical protein
HXP36_23920	hypothetical protein
HXP36_23935	hypothetical protein
HXP36_24040	efflux RND transporter periplasmic adaptor subunit
HXP36_24110	calcium-binding protein
HXP36_24195	EAL domain-containing protein
HXP36_24265	nuclear transport factor 2 family protein
HXP36_24270	DUF4437 domain-containing protein
HXP36_24275	DsbA family oxidoreductase
HXP36_24280	LysR family transcriptional regulator
HXP36_24285	FAD-dependent monooxygenase

Moko typical	
**Gene**	**Annotation**
HXP36_24290	DsbA family protein
HXP36_24295	Lrp/AsnC family transcriptional regulator
HXP36_24300	AzlC family ABC transporter permease
HXP36_24305	AzlD family protein
HXP36_24325	hypothetical protein
HXP36_24330	carboxymuconolactone decarboxylase family protein
HXP36_24355	LysR family transcriptional regulator
HXP36_24360	pyridoxal phosphate-dependent aminotransferase
HXP36_24365	amino acid permease
HXP36_24370	GMC family oxidoreductase
HXP36_24375	GMC family oxidoreductase
RALB5_RS27170	filamentous hemagglutinin N-terminal domain-containing protein
HXP36_24640	hypothetical protein
HXP36_24645	hypothetical protein
HXP36_24650	SUMF1/EgtB/PvdO family nonheme iron enzyme
HXP36_24655	hypothetical protein
HXP36_24740	acyl carrier protein
HXP36_24795	hypothetical protein
HXP36_24870	hypothetical protein
HXP36_24935	type III effector protein
HXP36_24940	hypothetical protein
HXP36_24945	hypothetical protein
HXP36_24950	hypothetical protein
HXP36_24955	hypothetical protein
HXP36_24960	hypothetical protein

Moko typical	
**Gene**	**Annotation**
HXP36_25070	toll/interleukin-1 receptor domain-containing protein
HXP36_25075	hypothetical protein
HXP36_25280	hypothetical protein
UW163_RS00075	hypothetical protein
RALW1_RS21930	MFS transporter
HXP37_11355	response regulator transcription factor
HXP37_11540	hypothetical protein
HXP37_11595	hypothetical protein
HXP36_23610	hypothetical protein
HXP37_16185	XRE family transcriptional regulator
HXP37_20290	tartrate dehydrogenase
HXP37_20355	(2Fe-2S)-binding protein
C2I33_RS25520	RHS repeat protein
C2I33_RS25780	RHS repeat protein
RALFB_RS27435	hypothetical protein
HXP36_00270	RHS repeat protein
HXP35_08560	DUF4102 domain-containing protein
HXP35_16295	IS5 family transposase
HXP35_21810	hypothetical protein
UW163_RS00045	hypothetical protein
HXP36_02545	DUF1484 family protein
C2I33_RS24945	RHS repeat protein
C2I33_RS25810	RHS repeat protein
C2I33_RS25800	RHS repeat protein
C2I33_RS20360	RHS repeat protein

Moko typical	
**Gene**	**Annotation**
HXP36_02640	RHS repeat protein
AQR21_RS23350	filamentous hemagglutinin N-terminal domain-containing protein
EIH11_RS23195	hypothetical protein
UW163_RS11520	IS3 family transposase
UW163_RS11565	IS3 family transposase
C2I33_RS24855	NTP transferase domain-containing protein
RALB5_RS00005	hypothetical protein
RALB5_RS00015	acyltransferase family protein
RALB5_RS27270	hypothetical protein
RALB5_RS27350	hypothetical protein
RALB5_RS04110	efflux transporter outer membrane subunit
RALB5_RS05865	TOBE domain-containing protein
RALB5_RS26180	SDR family oxidoreductase
RALFB_RS25510	TSUP family transporter
RALB5_RS27420	hypothetical protein
RALB5_RS27450	hypothetical protein
RALGR_RS26485	filamentous hemagglutinin N-terminal domain-containing protein
RALB5_RS26295	hypothetical protein
RALB5_RS11715	ABC transporter permease
RALB5_RS27590	hypothetical protein
RALB5_RS27600	hypothetical protein
RALB5_RS16480	FAD-dependent oxidoreductase
RALB5_RS17535	MFS transporter
RALB5_RS19510	hypothetical protein
C2I33_RS21835	hypothetical protein

Moko typical	
**Gene**	**Annotation**
RALB5_RS00245	Tn3 family transposase
RALB5_RS20410	IS21 family transposase
group_6739	MprA protease, GlyGly-CTERM protein-sorting domain-containing form
AQR24_RS26035	Tn3 family transposase
RALB5_RS21315	hypothetical protein
RALB5_RS27755	hypothetical protein
RALB5_RS27795	hypothetical protein
RALB5_RS27805	hypothetical protein
C2I33_RS25645	hypothetical protein
RALB5_RS23840	hypothetical protein
RALB5_RS27895	hypothetical protein
RALB5_RS24440	TolB family protein
RALB5_RS24525	hypothetical protein
RALB5_RS24785	head completion/stabilization protein
RALB5_RS27035	hypothetical protein
RALB5_RS27965	AMP-binding protein
RALB5_RS27070	DUF2345 domain-containing protein
RALB5_RS25095	phage baseplate assembly protein V
RALB5_RS28000	hypothetical protein
RALB5_RS25115	phage minor tail protein L
RALB5_RS28025	hypothetical protein
RALB5_RS25345	helix-turn-helix domain-containing protein
RALB5_RS28060	head completion/stabilization protein
RALB5_RS25380	phage minor tail protein L
RALB5_RS28105	aminotransferase class III-fold pyridoxal phosphate-dependent enzyme

Moko typical	
**Gene**	**Annotation**
RALB5_RS28140	hypothetical protein
group_6890	bifunctional hydroxymethylpyrimidine kinase/phosphomethylpyrimidine kinase
RALW1_RS25780	transposase
RALW1_RS24510	DUF1484 family protein
RALW1_RS24540	transposase
RALW1_RS25800	IS3 family transposase
RALW1_RS25860	hypothetical protein
RALW1_RS25900	hypothetical protein
RALW1_RS25910	twin-arginine translocation signal domain-containing protein
C2I33_RS18955	filamentous hemagglutinin N-terminal domain-containing protein
RALW1_RS05165	EamA family transporter
RALW1_RS25955	hypothetical protein
RALW1_RS25985	hypothetical protein
RALW1_RS26085	hypothetical protein
RALW1_RS25190	hypothetical protein
RALW1_RS26135	hypothetical protein
RALW1_RS26140	hypothetical protein
RALW1_RS26150	hypothetical protein
RALW1_RS26155	hypothetical protein
RALW1_RS18075	hypothetical protein
RALW1_RS26160	hypothetical protein
UW163_RS23635	AraC family transcriptional regulator
RALW1_RS18865	AI-2E family transporter
RALW1_RS18915	peptidase
group_6917	S-formylglutathione hydrolase

Moko typical	
**Gene**	**Annotation**
RALW1_RS20290	hypothetical protein
RALW1_RS26225	hypothetical protein
RALW1_RS21085	MFS transporter
RALW1_RS26270	hypothetical protein
RALW1_RS22825	amino acid permease
RALW1_RS22950	ABC transporter permease subunit
RALW1_RS23200	amino acid ABC transporter permease
RALW1_RS23380	BamA/TamA family outer membrane protein
RALW1_RS23450	arginase
RALW1_RS26360	hypothetical protein
RALW1_RS26365	hypothetical protein
RALW1_RS26370	hypothetical protein
RALW1_RS23810	DUF3489 domain-containing protein
RALW1_RS23855	IS3 family transposase
RALW1_RS26395	hypothetical protein
RALW1_RS24075	HpcH/HpaI aldolase/citrate lyase family protein
RALW1_RS26425	IS3 family transposase
group_6936	peptidoglycan editing factor PgeF
RALW1_RS24250	H-NS histone family protein
RALW1_RS26455	hypothetical protein
group_694	trifunctional transcriptional regulator/proline dehydrogenase/L-glutamate gamma-semialdehyde dehydrogenase
RALGR_RS25780	hypothetical protein
RALGR_RS25785	hypothetical protein
RALGR_RS25790	hypothetical protein
RALGR_RS00195	hypothetical protein

Moko typical	
**Gene**	**Annotation**
RALGR_RS25815	hypothetical protein
RALGR_RS25820	hypothetical protein
group_7020	type VI secretion system tip protein VgrG
RALGR_RS25940	hypothetical protein
HXP37_04240	ATP-binding protein
UW163_RS26245	S8 family serine peptidase
RALGR_RS11170	DEAD/DEAH box helicase
RALGR_RS12040	tetratricopeptide repeat protein
RALGR_RS26065	hypothetical protein
RALGR_RS26080	hypothetical protein
RALGR_RS26100	hypothetical protein
RALGR_RS14075	hypothetical protein
RALGR_RS25025	hypothetical protein
RALGR_RS16040	porin
RALGR_RS16255	MMPL family transporter
RALGR_RS26125	hypothetical protein
RALGR_RS18695	DUF1059 domain-containing protein
RALGR_RS26255	hypothetical protein
UW163_RS23675	DNA-binding protein
HXP35_03065	hypothetical protein
RALGR_RS26350	hypothetical protein
RALGR_RS26355	hypothetical protein
RALGR_RS26360	hypothetical protein
RALGR_RS22990	hypothetical protein
RALGR_RS23120	hypothetical protein

Moko typical	
**Gene**	**Annotation**
RALGR_RS23405	RHS repeat protein
RALGR_RS23930	AMP-binding protein
RALGR_RS25670	IS3 family transposase
RALGR_RS26465	SMEK domain-containing protein
RALGR_RS26470	hypothetical protein
RALGR_RS25740	hypothetical protein
RALGR_RS24295	DUF2345 domain-containing protein
RALFB_RS27535	hypothetical protein
RALFB_RS00050	copper-binding protein
RALFB_RS26185	IS3 family transposase
group_7316	copper homeostasis periplasmic binding protein CopC
RALFB_RS01825	copper resistance protein CopB
RALFB_RS01830	copper resistance system multicopper oxidase
RALFB_RS01835	heavy metal response regulator transcription factor
RALFB_RS01840	heavy metal sensor histidine kinase
RALFB_RS01845	oleate hydratase
RALFB_RS26240	heavy-metal-associated domain-containing protein
RALFB_RS01850	hypothetical protein
RALFB_RS01855	YHS domain-containing protein
RALFB_RS01860	DUF2933 domain-containing protein
RALFB_RS01865	isoprenylcysteine carboxylmethyltransferase family protein
RALFB_RS01870	MBL fold metallo-hydrolase
RALFB_RS01875	thymidine phosphorylase family protein
group_7329	ribose-phosphate pyrophosphokinase
RALFB_RS27600	hypothetical protein

Moko typical	
**Gene**	**Annotation**
RALFB_RS03140	efflux RND transporter periplasmic adaptor subunit
RALFB_RS03145	TolC family protein
RALFB_RS03150	hypothetical protein
RALFB_RS03155	hypothetical protein
RALFB_RS03160	heavy metal translocating P-type ATPase
RALFB_RS03165	hypothetical protein
RALFB_RS26275	YHS domain-containing protein
RALFB_RS03170	DUF2933 domain-containing protein
RALFB_RS03175	isoprenylcysteine carboxylmethyltransferase family protein
RALFB_RS03180	hypothetical protein
RALFB_RS03185	copper resistance protein B
RALFB_RS03190	copper resistance system multicopper oxidase
RALFB_RS03195	DUF3141 domain-containing protein
RALFB_RS03200	hypothetical protein
RALFB_RS26280	heavy-metal-associated domain-containing protein
RALFB_RS03205	hypothetical protein
RALFB_RS03210	hypothetical protein
RALFB_RS03220	ABC transporter ATP-binding protein
RALFB_RS03225	ABC transporter permease
RALFB_RS03230	hypothetical protein
RALFB_RS03235	AarF/ABC1/UbiB kinase family protein
RALFB_RS03245	LemA family protein
RALFB_RS03250	YgcG family protein
RALFB_RS03255	membrane protein
RALFB_RS03260	hypothetical protein

Moko typical	
**Gene**	**Annotation**
RALFB_RS03265	hypothetical protein
RALFB_RS03270	hypothetical protein
RALFB_RS03275	MFS transporter
RALFB_RS03280	heavy metal translocating P-type ATPase
RALFB_RS03285	hypothetical protein
RALFB_RS04250	AAA family ATPase
RALFB_RS04265	sel1 repeat family protein
RALFB_RS06060	hypothetical protein
RALFB_RS06065	hypothetical protein
RALFB_RS06070	hypothetical protein
group_7367	single-stranded DNA-binding protein
RALFB_RS06080	zinc-binding protein
RALFB_RS06085	hypothetical protein
RALFB_RS06090	hypothetical protein
RALFB_RS06095	PRTRC system protein D
RALFB_RS06100	tyrosine-type recombinase/integrase
RALFB_RS06105	hypothetical protein
RALFB_RS06110	DNA/RNA non-specific endonuclease
RALFB_RS06115	helix-turn-helix transcriptional regulator
RALFB_RS06120	hypothetical protein
RALFB_RS06125	phage Gp37/Gp68 family protein
RALFB_RS27745	hypothetical protein
RALFB_RS27750	DUF4031 domain-containing protein
RALFB_RS06130	hypothetical protein
RALFB_RS26405	hypothetical protein

Moko typical	
**Gene**	**Annotation**
RALFB_RS26410	hypothetical protein
RALFB_RS06145	hypothetical protein
RALFB_RS06150	hypothetical protein
RALFB_RS06155	DUF1173 family protein
RALFB_RS06160	ATP-dependent helicase
RALFB_RS06165	hypothetical protein
RALFB_RS06170	hypothetical protein
RALFB_RS06175	hypothetical protein
RALFB_RS06180	N-6 DNA methylase
RALFB_RS06185	PRTRC system ParB family protein
RALFB_RS06190	PRTRC system protein E
RALFB_RS06195	PRTRC system protein C
RALFB_RS06200	PRTRC system protein F
RALFB_RS06205	PRTRC system protein B
RALFB_RS06210	PRTRC system protein A
RALFB_RS06220	hypothetical protein
RALFB_RS06910	mannose-1-phosphate guanylyltransferase
RALFB_RS08125	branched-chain amino acid ABC transporter permease
RALFB_RS08545	phage holin family protein
RALFB_RS08555	hypothetical protein
RALFB_RS27805	hypothetical protein
RALFB_RS08590	tail protein
RALFB_RS08595	hypothetical protein
RALFB_RS26525	hypothetical protein
RALFB_RS08600	phage tail sheath protein

Moko typical	
**Gene**	**Annotation**
RALFB_RS08605	phage major tail tube protein
RALFB_RS08610	phage tail assembly protein
RALFB_RS08620	hypothetical protein
RALFB_RS08625	phage tail protein
RALFB_RS26535	hypothetical protein
RALFB_RS08645	helix-turn-helix transcriptional regulator
RALFB_RS26545	hypothetical protein
RALFB_RS08655	hypothetical protein
RALFB_RS08660	ogr/Delta-like zinc finger family protein
RALFB_RS08665	hypothetical protein
RALFB_RS08670	hypothetical protein
RALFB_RS08680	hypothetical protein
RALFB_RS08685	hypothetical protein
RALFB_RS08690	hypothetical protein
RALFB_RS08695	hypothetical protein
RALFB_RS08700	hypothetical protein
RALFB_RS08705	hypothetical protein
RALFB_RS08715	site-specific integrase
RALFB_RS10220	hypothetical protein
RALFB_RS10225	hypothetical protein
RALFB_RS27820	hypothetical protein
RALFB_RS10260	hypothetical protein
RALFB_RS10265	hypothetical protein
RALFB_RS27825	hypothetical protein
RALFB_RS10275	hypothetical protein

Moko typical	
**Gene**	**Annotation**
RALFB_RS10280	TrbG/VirB9 family P-type conjugative transfer protein
RALFB_RS10285	hypothetical protein
RALFB_RS10290	N-acetylmuramoyl-L-alanine amidase
RALFB_RS27830	hypothetical protein
RALFB_RS10300	hypothetical protein
RALFB_RS10305	hypothetical protein
RALFB_RS10310	hypothetical protein
RALFB_RS10315	hypothetical protein
RALFB_RS10320	hypothetical protein
RALFB_RS10325	hypothetical protein
RALFB_RS10330	hypothetical protein
RALFB_RS10335	hypothetical protein
RALFB_RS10340	hypothetical protein
RALFB_RS10345	glycine zipper 2TM domain-containing protein
RALFB_RS10350	hypothetical protein
RALFB_RS21570	DUF3313 domain-containing protein
RALFB_RS10355	hypothetical protein
RALFB_RS10360	hypothetical protein
RALFB_RS10365	hypothetical protein
RALFB_RS10370	hypothetical protein
RALFB_RS10380	hypothetical protein
RALFB_RS10385	ATP-binding protein
RALFB_RS10390	hypothetical protein
RALFB_RS10395	hypothetical protein
RALFB_RS10400	hypothetical protein

Moko typical	
**Gene**	**Annotation**
RALFB_RS10410	hypothetical protein
RALFB_RS27835	hypothetical protein
RALFB_RS26650	hypothetical protein
RALFB_RS26015	hypothetical protein
RALFB_RS10425	hypothetical protein
RALFB_RS10430	polymer-forming cytoskeletal protein
RALFB_RS26655	hypothetical protein
RALFB_RS10435	hypothetical protein
RALFB_RS26660	hypothetical protein
RALFB_RS10440	hypothetical protein
RALFB_RS10445	DNA repair exonuclease
RALFB_RS10450	hypothetical protein
RALFB_RS10455	AAA family ATPase
RALFB_RS10460	hypothetical protein
RALFB_RS10465	hypothetical protein
RALFB_RS27840	hypothetical protein
RALFB_RS10475	hypothetical protein
RALFB_RS26670	hypothetical protein
RALFB_RS10480	Tn3 family transposase
RALFB_RS10485	hypothetical protein
RALFB_RS12145	integrase family protein
RALFB_RS12150	hypothetical protein
RALFB_RS27890	hypothetical protein
RALFB_RS27975	hypothetical protein
RALFB_RS14370	phosphomannomutase/phosphoglucomutase

Moko typical	
**Gene**	**Annotation**
RALFB_RS14930	arginase
RALFB_RS28030	hypothetical protein
RALFB_RS28035	hypothetical protein
RALFB_RS28065	hypothetical protein
RALFB_RS28085	Tn3 family transposase
RALFB_RS17640	hypothetical protein
RALFB_RS18260	GPO family capsid scaffolding protein
RALFB_RS18590	universal stress protein
RALFB_RS28110	hypothetical protein
RALFB_RS19055	Hsp70 family protein
RALFB_RS19640	lauroyl acyltransferase
RALFB_RS28165	hypothetical protein
RALFB_RS27120	hypothetical protein
RALFB_RS20885	hypothetical protein
RALFB_RS22950	DMT family transporter
RALFB_RS23355	hypothetical protein
group_7500	copper homeostasis membrane protein CopD
RALFB_RS23970	metal-binding protein
ppk2	polyphosphate kinase 2
group_7503	ATP-dependent metallopeptidase FtsH/Yme1/Tma family protein
RALFB_RS23985	DNA-binding protein
RALFB_RS24410	TonB-dependent receptor
RALFB_RS24790	HIT domain-containing protein
RALFB_RS28275	hypothetical protein
RALFB_RS24880	twin-arginine translocation signal domain-containing protein

Moko typical	
**Gene**	**Annotation**
RALFB_RS24920	ATP-binding cassette domain-containing protein
RALFB_RS25010	AAA family ATPase
RALFB_RS25015	hypothetical protein
RALFB_RS27340	hypothetical protein
RALFB_RS28290	hypothetical protein
RALFB_RS25435	ABC transporter permease subunit
RALFB_RS28320	hypothetical protein
RALFB_RS25605	aminotransferase class I/II-fold pyridoxal phosphate-dependent enzyme
RALFB_RS25630	EamA family transporter
RALFB_RS28335	AMP-binding protein
RALFB_RS27440	hypothetical protein
RALFB_RS28340	DDE-type integrase/transposase/recombinase
RALFB_RS25805	hypothetical protein
RALFB_RS28350	hypothetical protein
RALFB_RS27470	hypothetical protein
group_7524	4-hydroxy-2-oxoheptanedioate aldolase
AQR24_RS26410	inovirus-type Gp2 protein
AQR24_RS00040	DNA-binding protein
AQR24_RS00050	DUF2523 domain-containing protein
AQR24_RS27810	hypothetical protein
AQR24_RS00145	hypothetical protein
AQR24_RS00150	hypothetical protein
AQR24_RS26435	phage-like protein
AQR24_RS26440	DUF4124 domain-containing protein
AQR24_RS27830	hypothetical protein

Moko typical	
**Gene**	**Annotation**
AQR24_RS27835	hypothetical protein
AQR24_RS27845	hypothetical protein
AQR24_RS27850	hypothetical protein
AQR24_RS27870	hypothetical protein
AQR24_RS04060	hypothetical protein
AQR24_RS04065	hypothetical protein
AQR24_RS27960	hypothetical protein
AQR24_RS27965	hypothetical protein
AQR24_RS13005	hypothetical protein
AQR24_RS15750	response regulator transcription factor
AQR24_RS28185	hypothetical protein
AQR24_RS28245	hypothetical protein
AQR24_RS20690	Bro-N domain-containing protein
AQR24_RS28325	hypothetical protein
AQR24_RS22940	J domain-containing protein
AQR24_RS27260	hypothetical protein
AQR24_RS23105	host specificity protein J
AQR24_RS23245	mannose-1-phosphate guanylyltransferase
AQR24_RS23870	MATE family efflux transporter
AQR24_RS24385	DNA cytosine methyltransferase
group_7557	prolyl aminopeptidase
AQR24_RS28435	hypothetical protein
AQR24_RS24745	EAL domain-containing protein
AQR24_RS28455	hypothetical protein
AQR24_RS25785	alpha/beta fold hydrolase

Moko typical	
**Gene**	**Annotation**
AQR24_RS28515	hypothetical protein
AQR24_RS28535	hypothetical protein
AQR24_RS28560	DUF3274 domain-containing protein
AQR24_RS25910	hypothetical protein
AQR24_RS28585	hypothetical protein
AQR24_RS28635	hypothetical protein
AQR24_RS28640	hypothetical protein
AQR24_RS28695	MFS transporter
AQR24_RS28710	GpE family phage tail protein
AQR24_RS26315	IS3 family transposase
group_7572	IS3 family transposase
AQR24_RS28750	hypothetical protein
AQR21_RS23980	hypothetical protein
AQR21_RS25315	hypothetical protein
AQR21_RS03765	hypothetical protein
AQR21_RS04635	hypothetical protein
AQR21_RS05510	hypothetical protein
AQR21_RS25455	hypothetical protein
AQR21_RS25540	hypothetical protein
AQR21_RS25550	hypothetical protein
AQR21_RS11315	Type III effector protein (Skwp5)
AQR21_RS12655	hypothetical protein
AQR21_RS25595	hypothetical protein
AQR21_RS12890	2-hydroxychromene-2-carboxylate isomerase
AQR21_RS24725	restriction endonuclease subunit S

Moko typical	
**Gene**	**Annotation**
AQR21_RS15340	hypothetical protein
AQR21_RS15345	type I restriction endonuclease subunit R
AQR21_RS25680	hypothetical protein
AQR21_RS16210	hypothetical protein
AQR21_RS17125	MFS transporter
UW163_RS26420	hypothetical protein
AQR21_RS25760	hypothetical protein
AQR21_RS18395	SAM-dependent DNA methyltransferase
AQR21_RS18595	arginase
AQR21_RS25785	hypothetical protein
AQR21_RS25790	hypothetical protein
AQR21_RS20650	ATP-dependent DNA helicase RecG
AQR21_RS20655	hypothetical protein
AQR21_RS20660	hypothetical protein
AQR21_RS25835	hypothetical protein
AQR21_RS24965	hypothetical protein
group_7608	P-type conjugative transfer protein TrbL
AQR21_RS21725	type I toxin-antitoxin system ptaRNA1 family toxin
AQR21_RS21730	hypothetical protein
AQR21_RS25885	hypothetical protein
AQR21_RS22315	hypothetical protein
traJ	conjugal transfer transcriptional regulator TraJ
group_7614	P-type conjugative transfer protein TrbJ
AQR21_RS22840	TIM barrel protein
AQR21_RS22915	transposase

Moko typical	
**Gene**	**Annotation**
AQR21_RS23045	EamA family transporter
AQR21_RS26000	hypothetical protein
AQR21_RS23785	hypothetical protein
AQR21_RS26025	hypothetical protein
AQR21_RS23845	hypothetical protein
AQR21_RS25235	glycoside hydrolase family protein
AQR21_RS23895	AMP-binding protein
AQR21_RS25260	transposase
AQR21_RS26100	DUF2345 domain-containing protein
UW163_RS06935	HAMP domain-containing histidine kinase
UW163_RS23630	hypothetical protein
UW163_RS26405	hypothetical protein
UW163_RS23695	hypothetical protein
UW163_RS24255	hypothetical protein
UW163_RS23740	hypothetical protein
UW163_RS23745	type II toxin-antitoxin system VapC family toxin
UW163_RS23760	hypothetical protein
UW163_RS23770	hypothetical protein
UW163_RS23775	DUF3800 domain-containing protein
UW163_RS23780	ParB/RepB/Spo0J family partition protein
UW163_RS23785	AAA family ATPase
UW163_RS23790	replication initiation protein
UW163_RS23815	hypothetical protein
UW163_RS23820	lytic transglycosylase domain-containing protein
UW163_RS23825	hypothetical protein

Moko typical	
**Gene**	**Annotation**
UW163_RS23830	type IV secretory pathway VirB3 family protein
UW163_RS23845	hypothetical protein
UW163_RS23870	TrbI/VirB10 family protein
UW163_RS23900	hypothetical protein
UW163_RS23910	hypothetical protein
UW163_RS23915	hypothetical protein
UW163_RS23920	hypothetical protein
UW163_RS23935	hypothetical protein
UW163_RS23945	DUF4123 domain-containing protein
UW163_RS23950	DUF3304 domain-containing protein
UW163_RS23955	DUF2235 domain-containing protein
UW163_RS23960	hypothetical protein
UW163_RS24000	hypothetical protein
UW163_RS24005	GMC family oxidoreductase
UW163_RS24010	hypothetical protein
UW163_RS24015	S1/P1 nuclease
UW163_RS24025	hypothetical protein
UW163_RS24030	hypothetical protein
UW163_RS24035	hypothetical protein
UW163_RS24040	hypothetical protein
UW163_RS26415	hypothetical protein
UW163_RS24055	relaxase/mobilization nuclease domain-containing protein
UW163_RS24060	DUF2807 domain-containing protein
UW163_RS24065	ribbon-helix-helix protein, CopG family
UW163_RS24085	DNA-binding protein

Moko typical	
**Gene**	**Annotation**
UW163_RS24090	H-NS histone family protein
UW163_RS26200	hypothetical protein
UW163_RS24095	S8/S53 family peptidase
UW163_RS24105	hypothetical protein
UW163_RS24110	hypothetical protein
UW163_RS24115	hypothetical protein
UW163_RS24120	YqaE/Pmp3 family membrane protein
UW163_RS24125	hypothetical protein
UW163_RS24130	hypothetical protein
UW163_RS26205	hypothetical protein
UW163_RS24145	hypothetical protein
UW163_RS24150	hypothetical protein
UW163_RS24155	hypothetical protein
UW163_RS25505	hypothetical protein
UW163_RS24170	hypothetical protein
UW163_RS24175	hypothetical protein
UW163_RS24180	hypothetical protein
UW163_RS24185	hypothetical protein
UW163_RS26210	hypothetical protein
UW163_RS24190	hypothetical protein
UW163_RS24195	hypothetical protein
UW163_RS24200	H-NS histone family protein
UW163_RS24205	hypothetical protein
UW163_RS26225	hypothetical protein
UW163_RS26235	hypothetical protein

Moko typical	
**Gene**	**Annotation**
UW163_RS26240	hypothetical protein
EIH10_RS13820	efflux RND transporter periplasmic adaptor subunit
HXP34_21490	YdcF family protein
EIH10_RS23505	hypothetical protein
EIH10_RS23535	hypothetical protein
EIH10_RS20530	hypothetical protein
HXP36_23445	NAD(P)/FAD-dependent oxidoreductase
EIH10_RS22435	DUF3304 domain-containing protein
EIH10_RS23600	DUF4238 domain-containing protein
EIH10_RS23610	hypothetical protein
EIH10_RS23100	hypothetical protein
EIH10_RS23260	hypothetical protein
EIH10_RS23640	hypothetical protein
EIH12_RS02570	tripartite tricarboxylate transporter TctB family protein
EIH12_RS24025	hypothetical protein
EIH12_RS24055	hypothetical protein
EIH12_RS15925	hypothetical protein
EIH12_RS24090	hypothetical protein
EIH12_RS23290	N-6 DNA methylase
EIH12_RS23420	hypothetical protein
HXP36_23430	EAL domain-containing protein
EIH12_RS23515	hypothetical protein
EIH12_RS23850	hypothetical protein
EIH13_RS13080	transposase
EIH13_RS16930	hemagglutinin

Moko typical	
**Gene**	**Annotation**
EIH13_RS24215	SDR family NAD(P)-dependent oxidoreductase
EIH14_RS00450	LLM class flavin-dependent oxidoreductase
EIH14_RS02515	amino acid ABC transporter ATP-binding protein
HXP36_23405	Type III effector protein (Skwp 4)
HXP36_23395	MCE family protein
EIH14_RS14935	DUF4747 family protein
RALB5_RS01985	pilus assembly protein
EIH10_RS22870	pilus assembly protein
EIH14_RS22900	DUF4268 domain-containing protein
EIH09_RS20135	endonuclease
EIH09_RS22150	integrase arm-type DNA-binding domain-containing protein
EIH11_RS01620	MoxR family ATPase
C2I33_RS19010	hypothetical protein
C2I33_RS19245	hypothetical protein
C2I33_RS04485	hypothetical protein
C2I33_RS19945	hypothetical protein
EIH12_RS20215	pilus assembly protein
C2I33_RS20340	hypothetical protein
C2I33_RS20595	hypothetical protein
C2I33_RS20725	hypothetical protein
C2I33_RS20880	hypothetical protein
C2I33_RS21100	hypothetical protein
C2I33_RS21125	hypothetical protein
C2I33_RS04865	hypothetical protein
C2I33_RS04950	hypothetical protein

Moko typical	
**Gene**	**Annotation**
C2I33_RS05045	transporter
C2I33_RS21325	hypothetical protein
C2I33_RS21660	integrase
C2I33_RS05430	hypothetical protein
C2I33_RS22365	aldehyde dehydrogenase family protein
C2I33_RS22580	hypothetical protein
C2I33_RS05990	hypothetical protein
C2I33_RS23255	hypothetical protein
C2I33_RS23270	hypothetical protein
C2I33_RS23325	hypothetical protein
C2I33_RS23540	hypothetical protein
C2I33_RS24010	alpha-E domain-containing protein
RALGR_RS18295	pilus assembly protein
C2I33_RS00260	hypothetical protein
C2I33_RS00765	EamA family transporter
C2I33_RS24535	hypothetical protein
C2I33_RS24850	hypothetical protein
C2I33_RS07385	hypothetical protein
group_8065	septum site-determining protein MinC
C2I33_RS07695	hypothetical protein
C2I33_RS07820	hypothetical protein
C2I33_RS25200	hypothetical protein
C2I33_RS25240	amidohydrolase family protein
C2I33_RS25270	dihydrolipoyllysine-residue acetyltransferase
C2I33_RS25310	hypothetical protein

Moko typical	
**Gene**	**Annotation**
C2I33_RS08185	hypothetical protein
C2I33_RS08210	hypothetical protein
C2I33_RS08420	hypothetical protein
C2I33_RS08730	hypothetical protein
C2I33_RS25615	RHS repeat protein
C2I33_RS00770	DUF2339 domain-containing protein
C2I33_RS01205	hypothetical protein
C2I33_RS25640	RHS repeat protein
C2I33_RS25695	RHS repeat protein
C2I33_RS25835	filamentous hemagglutinin N-terminal domain-containing protein
C2I33_RS10710	RHS repeat protein
C2I33_RS10895	transposase
C2I33_RS10930	hypothetical protein
C2I33_RS01610	hypothetical protein
C2I33_RS11715	hypothetical protein
C2I33_RS12545	hypothetical protein
C2I33_RS12600	hypothetical protein
C2I33_RS12730	hypothetical protein
C2I33_RS12825	hypothetical protein
C2I33_RS13200	hypothetical protein
C2I33_RS13560	hypothetical protein
C2I33_RS02525	hypothetical protein
C2I33_RS14570	hypothetical protein
C2I33_RS14870	hypothetical protein
C2I33_RS14880	hypothetical protein

Moko typical	
**Gene**	**Annotation**
C2I33_RS15470	hypothetical protein
C2I33_RS15480	hypothetical protein
C2I33_RS02915	hypothetical protein
C2I33_RS16385	hypothetical protein
C2I33_RS16460	hypothetical protein
C2I33_RS16605	hypothetical protein
C2I33_RS16930	hypothetical protein
C2I33_RS16945	hypothetical protein
C2I33_RS17085	Pr2TM family membrane protein
C2I33_RS18235	hypothetical protein
C2I33_RS18730	hypothetical protein
C2I33_RS18830	hypothetical protein
C2I33_RS18850	transglycosylase SLT domain-containing protein
C2I33_RS18895	DUF3005 domain-containing protein
C2I33_RS03845	hypothetical protein
UW163_RS01525	AAA family ATPase
UW163_RS24050	AAA family ATPase
UW163_RS01580	AAA family ATPase
HXP36_23810	hypothetical protein
HXP36_23535	ATP-dependent metallopeptidase FtsH/Yme1/Tma family protein
group_8231	phosphate regulon transcriptional regulator PhoB
group_8237	tryptophan synthase subunit beta
group_8245	phosphate signaling complex protein PhoU
group_8273	phosphate ABC transporter permease PstC
HXP34_21430	tryptophan synthase subunit alpha

Moko typical	
**Gene**	**Annotation**
group_8320	3-isopropylmalate dehydratase small subunit
group_8353	3-isopropylmalate dehydratase large subunit
HXP34_21370	succinate dehydrogenase assembly factor 2
group_8378	phosphoglucosamine mutase
group_8380	formyltetrahydrofolate deformylase
group_8390	exopolyphosphatase
HXP34_21495	heme-binding protein
RSPO_RS07340	IS3 family transposase
group_8414	succinate dehydrogenase, hydrophobic membrane anchor protein
HXP36_23600	DUF1488 domain-containing protein
group_8430	3-isopropylmalate dehydrogenase
group_8439	carbamoyl-phosphate synthase large subunit
HXP34_21360	succinate dehydrogenase flavoprotein subunit
UW163_RS25420	IS5 family transposase
group_8556	aspartate-semialdehyde dehydrogenase
HXP34_21365	succinate dehydrogenase iron-sulfur subunit
HXP34_21435	acetyl-CoA carboxylase carboxyltransferase subunit beta
UW163_RS01575	IS21 family transposase
UW163_RS04280	IS21 family transposase
UW163_RS06930	IS21 family transposase
EIH10_RS23635	calcium-binding protein
UW163_RS15290	IS21 family transposase
UW163_RS24215	IS21 family transposase
RSPO_RS16380	IS21 family transposase
UW163_RS01520	IS21 family transposase

Moko typical	
**Gene**	**Annotation**
UW163_RS24045	IS21 family transposase
RALW1_RS23340	ATP-binding protein
group_8606	lipid A hydroxylase LpxO
HXP34_21340	malate dehydrogenase
group_8622	citrate (Si)-synthase
group_8630	phosphate ABC transporter substrate-binding protein PstS
group_8642	transcription elongation factor GreA
HXP34_21450	CvpA family protein
group_8658	polyphosphate kinase 1
group_8675	methylisocitrate lyase
HXP34_21460	ABC transporter permease
HXP34_21510	MotA/TolQ/ExbB proton channel family protein
HXP36_23580	transglycosylase SLT domain-containing protein
RALW1_RS23470	MFS transporter
HXP36_23850	ABC transporter substrate-binding protein
HXP34_21505	biopolymer transporter ExbD
group_8837	phosphate regulon sensor histidine kinase PhoR
HXP34_21425	site-specific DNA-methyltransferase
HXP36_23540	RlmE family RNA methyltransferase
group_8880	phosphate ABC transporter ATP-binding protein PstB
group_8893	ribonuclease HI
RALW1_RS25685	tape measure protein
group_8923	amidophosphoribosyltransferase
HXP36_23605	TraR/DksA family transcriptional regulator
HXP34_21470	glycine zipper 2TM domain-containing protein

Moko typical	
**Gene**	**Annotation**
HXP36_23440	helix-turn-helix transcriptional regulator
HXP36_23545	YhbY family RNA-binding protein
HXP36_23840	ABC transporter ATP-binding protein
AQR21_RS22475	ABC transporter ATP-binding protein
HXP36_23795	hypothetical protein
UW163_RS01175	AAA family ATPase
UW163_RS04285	AAA family ATPase
UW163_RS06925	AAA family ATPase
UW163_RS15295	AAA family ATPase
UW163_RS24210	AAA family ATPase
HXP36_23830	ABC transporter permease
HXP36_23820	LysR family transcriptional regulator
group_9139	nodulation factor ABC transporter ATP-binding protein NodI
EIH14_RS23140	transposase
HXP36_23355	efflux transporter outer membrane subunit
**Sergipe facies**	
Gene	Annotation
C2L97_RS05445	IS21 family transposase
C2L97_RS07510	IS21 family transposase
C2L97_RS07875	IS21 family transposase
C2I38_RS20825	IS21 family transposase
C2I38_RS09790	ATP-binding cassette domain-containing protein
C2L97_RS01245	hypothetical protein
C2I38_RS02125	hypothetical protein
C2I38_RS17810	hypothetical protein
C2I38_RS18335	integrase

Moko typical	
**Gene**	**Annotation**
C2L97_RS23970	hypothetical protein
C2L97_RS00060	hypothetical protein
C2I38_RS24315	hypothetical protein
C2L97_RS01160	hypothetical protein
C2I38_RS01185	hypothetical protein
C2L97_RS01240	hypothetical protein
C2I38_RS01355	hypothetical protein
C2I38_RS01765	hypothetical protein
C2L97_RS02990	hypothetical protein
C2I38_RS03505	collagen-like triple helix repeat-containing protein
C2L97_RS04560	hypothetical protein
group_7717	type I-E CRISPR-associated protein Cse2/CasB
C2I38_RS24395	hypothetical protein
C2I38_RS08940	FAD-dependent oxidoreductase
C2L97_RS09670	GALA protein
C2I38_RS09750	type III effector protein
C2L97_RS11705	hypothetical protein
C2L97_RS16460	hypothetical protein
C2I38_RS16580	GALA protein
C2L97_RS16515	GNAT family N-acetyltransferase
C2L97_RS16995	hypothetical protein
C2L97_RS17080	4'-phosphopantetheinyl transferase superfamily protein
C2I38_RS24630	hypothetical protein
C2L97_RS17150	hypothetical protein
C2L97_RS18055	metallophosphoesterase
C2I38_RS24665	hypothetical protein
C2I38_RS18450	hypothetical protein
C2I38_RS20055	type III effector protein
C2L97_RS20110	type III effector protein

Moko typical	
**Gene**	**Annotation**
C2L97_RS20400	NUDIX hydrolase
C2I38_RS20670	collagen-like triple helix repeat-containing protein
C2I38_RS20920	hypothetical protein
C2I38_RS24755	hypothetical protein
C2I38_RS21875	hypothetical protein
C2L97_RS21925	hypothetical protein
C2I38_RS22115	type III effector protein
C2I38_RS22580	type III effector protein
C2L97_RS22615	Hpt domain-containing protein
C2L97_RS22695	HAD family hydrolase
C2I38_RS22905	Lrp/AsnC ligand binding domain-containing protein
C2L97_RS22815	hypothetical protein
C2L97_RS22830	amino acid (threonine) efflux protein
C2I38_RS23140	hypothetical protein
C2I38_RS24050	type I addiction module toxin, SymE family
C2I38_RS24815	hypothetical protein
C2L97_RS24100	hypothetical protein
C2I38_RS21910	type III effector protein
C2L97_RS07490	IS3 family transposase
C2L97_RS18380	IS5 family transposase
C2L97_RS07505	ATP-binding protein
C2L97_RS05450	ATP-binding protein
C2L97_RS16635	IS21 family transposase
C2L97_RS18205	IS21 family transposase
C2L97_RS18895	IS21 family transposase
C2L97_RS20700	IS21 family transposase
C2L97_RS22775	IS21 family transposase
C2I38_RS18290	IS21 family transposase
C2I38_RS18995	IS21 family transposase

Moko typical	
**Gene**	**Annotation**
C2I38_RS22930	IS21 family transposase
C2L97_RS12350	IS21 family transposase
C2I38_RS16715	IS21 family transposase
C2L97_RS12150	hypothetical protein
C2L97_RS20695	AAA family ATPase
C2L97_RS16630	AAA family ATPase
C2L97_RS18210	AAA family ATPase
C2L97_RS18890	AAA family ATPase
C2L97_RS12260	hypothetical protein
C2L97_RS12240	hypothetical protein
C2L97_RS12080	hypothetical protein
C2I38_RS01595	hypothetical protein
C2I38_RS04415	aculeacin A acylase
C2I38_RS05620	hypothetical protein
C2I38_RS24425	hypothetical protein
C2I38_RS06935	LysR family transcriptional regulator
C2I38_RS11060	hypothetical protein
C2I38_RS24500	hypothetical protein
C2I38_RS18305	hypothetical protein
C2L97_RS01155	hypothetical protein
C2L97_RS12265	hypothetical protein
C2L97_RS11015	hypothetical protein
C2L97_RS12035	phosphatidylserine/phosphatidylglycerophosphate/ cardiolipin synthase family protein
C2L97_RS12130	hypothetical protein
C2L97_RS12245	DUF3489 domain-containing protein
C2L97_RS13395	type III effector protein
C2L97_RS13405	hypothetical protein
C2L97_RS13450	hypothetical protein
C2L97_RS13755	O-antigen ligase C-terminal domain-containing protein

Moko typical	
**Gene**	**Annotation**
C2L97_RS13845	hypothetical protein
C2L97_RS13925	AMP-binding protein
C2L97_RS14195	tartrate dehydrogenase
C2L97_RS15135	hypothetical protein
C2L97_RS15210	DUF1328 domain-containing protein
C2L97_RS15470	hypothetical protein
C2L97_RS15490	zinc-ribbon domain-containing protein
C2L97_RS16425	hypothetical protein
C2L97_RS17655	hypothetical protein
C2L97_RS18410	hypothetical protein
C2L97_RS19205	hypothetical protein
C2L97_RS19260	hypothetical protein
C2L97_RS19420	hypothetical protein
C2L97_RS20725	IS5 family transposase
C2I38_RS18295	ATP-binding protein
C2I38_RS18990	ATP-binding protein
C2I38_RS22935	ATP-binding protein
**Bugtok disease**	
Gene	Annotation
RSMK_RS03090	DUF4158 domain-containing protein
RALCI_RS23880	IS3 family transposase
RSMK_RS26545	hypothetical protein
RSMK_RS25490	IS5 family transposase
RSMK_RS26235	IS3 family transposase
RSMK_RS18445	DDE-type integrase/transposase/recombinase
RSMK_RS26180	IS3 family transposase
RSMK_RS19425	hypothetical protein
group_1082	elongation factor Tu
RSMK_RS18000	response regulator

Moko typical	
**Gene**	**Annotation**
RALCI_RS26030	Minor spike protein
RALCI_RS26785	AMP-binding protein
RALCI_RS24200	AMP-binding protein
group_1204	DNA-directed RNA polymerase subunit alpha
RSMK_RS02275	IS21 family transposase
RSMK_RS09180	helix-turn-helix domain-containing protein
RSMK_RS07445	IS21-like element ISRso19 family transposase
RSMK_RS08550	TetR/AcrR family transcriptional regulator
RSMK_RS19840	glycine zipper 2TM domain-containing protein
RSMK_RS05930	helix-turn-helix domain-containing protein
RSMK_RS26250	helix-turn-helix domain-containing protein
RSMK_RS10855	IS256 family transposase
RSMK_RS21370	IS256 family transposase
RSMK_RS05980	PaaI family thioesterase
RSMK_RS15445	hypothetical protein
RSMK_RS20210	DUF2924 domain-containing protein
RSMK_RS09420	AAA domain-containing protein
RSMK_RS04415	aldolase
RSMK_RS25095	histone
RSMK_RS20205	NAD(P)-binding protein
RSMK_RS07820	hypothetical protein
RSMK_RS07395	Tn3 family transposase
RALCI_RS23350	Tn3 family transposase
RSMK_RS04420	IucA/IucC family siderophore biosynthesis protein
RALCI_RS25895	LysR family transcriptional regulator
RSMK_RS04435	IS3 family transposase
RSMK_RS10130	IS3 family transposase
RSMK_RS14520	transposase
RSMK_RS14660	IS3 family transposase

Moko typical	
**Gene**	**Annotation**
RSMK_RS19405	transposase
RSMK_RS08030	hypothetical protein
RSMK_RS20315	transposase
group_343	IS3 family transposase
RSMK_RS01985	hypothetical protein
RSMK_RS08490	hypothetical protein
RSMK_RS07900	LysR family transcriptional regulator
RALCI_RS24325	AMP-binding protein
RALCI_RS26780	AMP-binding protein
RSMK_RS08535	hypothetical protein
RSMK_RS25150	tRNA-guanine transglycosylase
RSMK_RS12005	serine/threonine-protein phosphatase
RSMK_RS00900	collagen-like triple helix repeat-containing protein
RALCI_RS19175	TOBE domain-containing protein
RSMK_RS07895	cysteine synthase A
RALCI_RS25870	hypothetical protein
RALCI_RS25880	hypothetical protein
RSMK_RS00470	hybrid sensor histidine kinase/response regulator
RSMK_RS00475	DUF1800 family protein
RSMK_RS26400	hypothetical protein
RSMK_RS26230	hypothetical protein
RSMK_RS20220	hypothetical protein
RSMK_RS20215	hypothetical protein
RSMK_RS26225	hypothetical protein
RSMK_RS05065	hypothetical protein
RSMK_RS26475	hypothetical protein
RSMK_RS06735	DUF4129 domain-containing protein
RSMK_RS26575	hypothetical protein
RSMK_RS25440	hypothetical protein

Moko typical	
**Gene**	**Annotation**
group_6266	5-dehydro-2-deoxygluconokinase
RSMK_RS25485	IS5 family transposase
RSMK_RS08920	DUF2090 domain-containing protein
RSMK_RS09680	DMT family transporter
RSMK_RS02000	methylated-DNA--[protein]-cysteine S-methyltransferase
RSMK_RS11870	EamA family transporter
group_6330	phosphate ABC transporter permease PstC
RSMK_RS25740	ankyrin repeat domain-containing protein
RSMK_RS14650	type III effector
RSMK_RS14845	AMP-binding protein
RSMK_RS15520	S-layer family protein
RSMK_RS15560	TOBE domain-containing protein
RSMK_RS16460	type III effector protein
RSMK_RS17020	efflux RND transporter periplasmic adaptor subunit
RSMK_RS17030	MMPL family transporter
RSMK_RS17975	porin
RSMK_RS18590	EamA family transporter
RSMK_RS26900	hypothetical protein
RSMK_RS19695	FAD-dependent oxidoreductase
RSMK_RS26375	excinuclease, ATPase subunit precursor
RALCI_RS00035	filamentous hemagglutinin N-terminal domain-containing protein
RALCI_RS01975	c-type cytochrome
RALCI_RS02325	ABC transporter permease subunit
RALCI_RS26185	hypothetical protein
RALCI_RS13665	dipeptide ABC transporter ATP-binding protein
RALCI_RS19865	HAD-IA family hydrolase
RALCI_RS22615	MFS transporter
RALCI_RS26635	hypothetical protein
RALCI_RS26640	hypothetical protein

Moko typical	
**Gene**	**Annotation**
RALCI_RS22980	hemagglutinin
RALCI_RS23200	VWA domain-containing protein
RALCI_RS26670	hypothetical protein
RALCI_RS23395	MFS transporter
RALCI_RS24230	AMP-binding protein
RALCI_RS24365	hypothetical protein
RALCI_RS25860	hypothetical protein
RALCI_RS26770	AMP-binding protein
RALCI_RS26800	hypothetical protein
RALCI_RS25900	hypothetical protein
RALCI_RS24530	DUF2427 domain-containing protein
RALCI_RS26820	LysR family transcriptional regulator
RALCI_RS25995	AAA domain-containing protein
RSMK_RS01995	GNAT family N-acetyltransferase
RSMK_RS04040	ABC transporter permease subunit
RSMK_RS10010	ATP-binding protein
RSMK_RS15245	ATP-binding protein
RSMK_RS21225	ATP-binding protein
RSMK_RS01410	ATP-binding protein
RSMK_RS18300	ATP-binding protein
RSMK_RS07915	carboxymuconolactone decarboxylase family protein
RSMK_RS07865	ATP-binding protein
RSMK_RS14570	AAA family ATPase
RSMK_RS23320	AAA family ATPase
RSMK_RS25240	IS5 family transposase
RALCI_RS24535	tRNA (cytidine(34)-2'-O)-methyltransferase
RSMK_RS10895	phosphoribosylglycinamide formyltransferase
RSMK_RS08500	DUF427 domain-containing protein
RSMK_RS10890	RsmB/NOP family class I SAM-dependent RNA methyltransferase

Moko typical	
**Gene**	**Annotation**
RSMK_RS19395	AAA family ATPase
RSMK_RS20775	AAA family ATPase
RSMK_RS16875	membrane protein
RALCI_RS20190	membrane protein
RSMK_RS07920	diguanylate cyclase
RSMK_RS14140	IS3 family transposase
RSMK_RS16075	IS3 family transposase
RSMK_RS23010	IS3 family transposase
RSMK_RS25185	IS3 family transposase
**Bugtok disease and Sergipe Facies**	
Gene	Annotation
RSMK_RS07995	ATP-binding protein
RSMK_RS18140	filamentous hemagglutinin N-terminal domain-containing protein
**Moko typical and Sergipe facies**	
Gene	Annotation
mreC	rod shape-determining protein MreC
HXP34_06785	urease accessory protein UreD
ureE	urease accessory protein UreE
EIH14_RS11240	hypothetical protein
HXP35_06850	phosphotransferase
RALB5_RS07750	MaoC family dehydratase
AQR24_RS14460	MaoC family dehydratase
UW163_RS10890	urease accessory protein UreJ
ureC	urease subunit alpha
RALFB_RS09595	fatty acid desaturase
RALFB_RS07625	efflux transporter outer membrane subunit
HXP36_08525	urease accessory protein UreF
urtA	urea ABC transporter substrate-binding protein

Moko typical	
**Gene**	**Annotation**
urtE	urea ABC transporter ATP-binding subunit UrtE
ureA	urease subunit gamma
EIH12_RS03390	urease subunit beta
ureG	urease accessory protein UreG
EIH13_RS02590	amino acid transporter
RALB5_RS17710	thiol:disulfide oxidoreductase
AQR24_RS24075	acyl-CoA dehydrogenase
EIH10_RS06240	enoyl-CoA hydratase
urtD	urea ABC transporter ATP-binding protein UrtD
EIH11_RS07875	hypothetical protein
AQR21_RS06400	ABC transporter substrate-binding protein
AQR21_RS21540	MFS transporter
EIH10_RS04745	exodeoxyribonuclease VII large subunit
HXP36_06490	cobalamin biosynthesis protein
EIH12_RS03435	isoprenylcysteine carboxylmethyltransferase family protein
UW163_RS01475	YheT family hydrolase
AQR21_RS04555	hypothetical protein
RALW1_RS16515	LysR family transcriptional regulator
urtC	urea ABC transporter permease subunit UrtC
C2I38_RS14030	PhoH family protein
C2L97_RS24035	metallophosphoesterase
leuA	2-isopropylmalate synthase
EIH14_RS15250	PLP-dependent aminotransferase family protein
UW163_RS20750	efflux RND transporter periplasmic adaptor subunit
EIH11_RS05370	membrane protein
EIH09_RS17260	PaaI family thioesterase
HXP35_00735	CoA transferase
AQR21_RS23460	SgcJ/EcaC family oxidoreductase
HXP35_02525	DUF2300 domain-containing protein

Moko typical	
**Gene**	**Annotation**
urtB	urea ABC transporter permease subunit UrtB
RALGR_RS23110	CBS domain-containing protein
RALB5_RS12840	rhomboid family intramembrane serine protease
EIH11_RS16530	EAL domain-containing protein
RSPO_RS21630	TlpA family protein disulfide reductase
EIH14_RS11635	type 1 glutamine amidotransferase domain-containing protein
HXP34_07640	DUF2892 domain-containing protein
AQR21_RS15450	metal-binding protein
C2L97_RS13050	chain-length determining protein
EIH13_RS19200	hypothetical protein
C2L97_RS20415	arsenate reductase ArsC
HXP35_21295	hypothetical protein
HXP34_12900	methyltransferase domain-containing protein
RALB5_RS18575	hypothetical protein
EIH13_RS01750	hypothetical protein
group_4582	dicarboxylate/amino acid:cation symporter
AQR21_RS09170	tartrate dehydrogenase
HXP37_18470	LysR family transcriptional regulator
UW163_RS05000	GNAT family N-acetyltransferase
HXP34_02335	two-component sensor histidine kinase
C2I38_RS03975	hypothetical protein
AQR21_RS13930	3-hydroxyacyl-CoA dehydrogenase
HXP37_18455	glycerate kinase
EIH09_RS00290	hypothetical protein
RSPO_RS06340	amidohydrolase
HXP35_14425	hypothetical protein
EIH14_RS04290	NADH:flavin oxidoreductase
EIH13_RS15555	FAD-dependent oxidoreductase
RALW1_RS08905	MHS family MFS transporter

Moko typical	
**Gene**	**Annotation**
HXP37_08080	PilT/PilU family type 4a pilus ATPase
EIH12_RS23930	hypothetical protein
AQR24_RS09290	hybrid sensor histidine kinase/response regulator
RALGR_RS00960	2-hydroxyacid dehydrogenase
EIH12_RS01455	DNA-deoxyinosine glycosylase
HXP36_00120	membrane protein
EIH09_RS03070	acyl-CoA-binding protein
AQR24_RS24095	histidine phosphatase family protein
HXP34_16870	hypothetical protein
C2L97_RS00385	sel1 repeat family protein
group_1977	ACP S-malonyltransferase
group_285	pyruvate kinase
C2I38_RS22540	Gfo/Idh/MocA family oxidoreductase
RSPO_RS19965	helix-turn-helix transcriptional regulator
EIH10_RS11815	RraA family protein
AQR24_RS03590	DUF853 domain-containing protein
AQR21_RS07365	collagen-like triple helix repeat-containing protein
HXP36_25090	transcriptional repressor
RALB5_RS21515	DcrB-related protein
EIH11_RS14605	DUF817 domain-containing protein
RALB5_RS08765	NAD(P)-dependent alcohol dehydrogenase
C2L97_RS19080	hypothetical protein
EIH11_RS15840	cobaltochelatase subunit CobN
RSPO_RS17080	hypothetical protein
RALW1_RS04670	DUF393 domain-containing protein
EIH14_RS16760	serine hydrolase
RALB5_RS08785	helix-turn-helix transcriptional regulator
HXP36_13970	hypothetical protein
C2I38_RS19240	carboxymuconolactone decarboxylase family protein

Moko typical	
**Gene**	**Annotation**
RSPO_RS18740	LysR family transcriptional regulator
AQR21_RS02820	HTTM domain-containing protein
RALGR_RS24385	hypothetical protein
HXP35_00505	hypothetical protein
RALB5_RS26515	hypothetical protein
UW163_RS16155	LysR family transcriptional regulator
AQR21_RS09115	hypothetical protein
HXP37_06925	LysR family transcriptional regulator
C2I38_RS01560	VOC family protein
UW163_RS05770	NYN domain-containing protein
UW163_RS05450	hypothetical protein
HXP36_02235	helix-turn-helix domain-containing protein
EIH10_RS20465	hypothetical protein
HXP37_22250	amino acid permease
EIH12_RS04300	aldo/keto reductase
EIH12_RS07920	hypothetical protein
EIH13_RS17500	hypothetical protein
AQR21_RS20645	DDE transposase family protein
RSPO_RS16395	type II toxin-antitoxin system RelE/ParE family toxin
EIH13_RS22080	choline dehydrogenase
C2I38_RS01075	DNA-binding protein
EIH09_RS00085	hypothetical protein
HXP36_02230	hypothetical protein
HXP37_16145	sel1 repeat family protein
HXP35_01460	hypothetical protein
RALB5_RS16100	helix-turn-helix domain-containing protein
C2L97_RS01080	hypothetical protein
HXP34_01480	hypothetical protein
UW163_RS05250	hypothetical protein

Moko typical	
**Gene**	**Annotation**
EIH13_RS06975	hypothetical protein
RALW1_RS14725	hypothetical protein
EIH10_RS21780	hypothetical protein
HXP34_16915	hypothetical protein
EIH11_RS15610	DHA2 family efflux MFS transporter permease subunit
EIH13_RS06985	hypothetical protein
RSPO_RS00075	hypothetical protein
EIH10_RS17365	MFS transporter
AQR24_RS06520	YOPP/AvrRxv family protein
AQR24_RS13855	recombinase family protein
EIH09_RS00150	DUF2924 domain-containing protein
C2L97_RS01410	DUF746 domain-containing protein
HXP37_05225	hypothetical protein
EIH09_RS21735	S-layer family protein
casA	type I-E CRISPR-associated protein Cse1/CasA
UW163_RS16135	PAAR domain-containing protein
EIH11_RS23005	hypothetical protein
EIH09_RS21105	DUF1641 domain-containing protein
C2L97_RS23850	NAD(P)/FAD-dependent oxidoreductase
C2L97_RS18375	hypothetical protein
EIH14_RS09370	hypothetical protein
C2L97_RS02810	alpha/beta hydrolase
EIH10_RS23515	hypothetical protein
EIH09_RS08805	AlpA family phage regulatory protein
cas5e	type I-E CRISPR-associated protein Cas5/CasD
cas6e	type I-E CRISPR-associated protein Cas6/Cse3/CasE
cas1e	type I-E CRISPR-associated endonuclease Cas1
cas2	type I-E CRISPR-associated endoribonuclease Cas2
HXP35_08865	hypothetical protein

Moko typical	
**Gene**	**Annotation**
UW163_RS00690	pilus assembly protein
RALW1_RS17750	zinc ribbon domain-containing protein
cas7e	type I-E CRISPR-associated protein Cas7/Cse4/CasC
group_4354	type IV pilus modification protein PilV
RALB5_RS11160	hypothetical protein
EIH14_RS23095	hypothetical protein
RSPO_RS12005	hypothetical protein
RALFB_RS09550	glyoxalase
AQR21_RS20545	hypothetical protein
C2I38_RS18480	DUF4265 domain-containing protein
group_165	type VI secretion system tip protein VgrG
EIH14_RS23065	hypothetical protein
EIH13_RS10435	phospholipase
UW163_RS19690	hypothetical protein
C2I38_RS01060	hypothetical protein
EIH13_RS22475	hypothetical protein
C2I38_RS12925	PilW family protein
HXP36_17950	HNH endonuclease
EIH11_RS16380	type VI secretion system tip protein VgrG
EIH14_RS15820	hypothetical protein
EIH09_RS15055	SDR family NAD(P)-dependent oxidoreductase
EIH13_RS09570	hypothetical protein
UW163_RS12875	putative addiction module antidote protein
UW163_RS18370	hypothetical protein
UW163_RS26355	hypothetical protein
UW163_RS19700	DMT family transporter
RALGR_RS01325	membrane protein
UW163_RS20300	DUF4865 family protein
RSPO_RS18860	nucleoside hydrolase

Moko typical	
**Gene**	**Annotation**
RALW1_RS16905	hypothetical protein
HXP37_17380	hypothetical protein
C2L97_RS18960	hypothetical protein
UW163_RS24450	hypothetical protein
RSPO_RS01040	ATP-binding protein
C2L97_RS01260	DNA cytosine methyltransferase
EIH09_RS20270	hypothetical protein
EIH13_RS22470	hypothetical protein
AQR24_RS12520	SPOR domain-containing protein
AQR24_RS05645	hypothetical protein
HXP36_17645	hypothetical protein
EIH12_RS24000	hypothetical protein
HXP36_01250	bifunctional (p)ppGpp synthetase/guanosine-3',5'-bis(diphosphate) 3'-pyrophosphohydrolase
C2I38_RS12075	hypothetical protein
RSPO_RS24975	PaaI family thioesterase
HXP36_19275	TetR family transcriptional regulator
RSPO_RS21470	LysR family transcriptional regulator
EIH11_RS11305	hypothetical protein
RSPO_RS24380	hypothetical protein
UW163_RS19310	putative 2-aminoethylphosphonate ABC transporter ATP-binding protein
UW163_RS26110	polyketide synthase RhiF
C2I38_RS08835	pilus assembly protein
RSPO_RS24870	tyrosine-type recombinase/integrase
EIH11_RS04410	DDE-type integrase/transposase/recombinase
RSPO_RS18050	peptidyl-prolyl cis-trans isomerase
EIH13_RS06905	DUF3489 domain-containing protein
RALW1_RS23775	hypothetical protein
HXP34_02060	DUF4153 domain-containing protein
HXP35_04520	methylated-DNA--[protein]-cysteine S-methyltransferase

Moko typical	
**Gene**	**Annotation**
AQR24_RS25475	membrane protein
EIH10_RS04880	hypothetical protein
cas3	CRISPR-associated helicase/endonuclease Cas3
EIH12_RS03050	hypothetical protein
AQR21_RS15590	hypothetical protein
EIH10_RS18450	universal stress protein
HXP34_14955	hypothetical protein
RALB5_RS12490	4-oxalocrotonate tautomerase family protein
RALB5_RS08910	hypothetical protein
AQR24_RS11535	DUF2063 domain-containing protein
HXP35_19675	DUF333 domain-containing protein
AQR21_RS20830	hypothetical protein
group_1773	type VI secretion system tip protein VgrG
HXP36_05225	hypothetical protein
HXP37_00175	hypothetical protein
C2L97_RS01145	site-specific DNA-methyltransferase
EIH14_RS15125	hypothetical protein
C2I38_RS07815	helix-turn-helix domain-containing protein
C2I38_RS12050	recombinase family protein
UW163_RS17880	DNA helicase
C2L97_RS21630	hypothetical protein
RSPO_RS23685	type III effector protein, AvrPphF family
UW163_RS25970	hypothetical protein
EIH10_RS17770	hypothetical protein
EIH13_RS06950	type II toxin-antitoxin system RelE/ParE family toxin
AQR24_RS07710	hypothetical protein
HXP35_04715	site-2 protease family protein
EIH10_RS04890	hypothetical protein
RALB5_RS02070	DUF3306 domain-containing protein

Moko typical	
**Gene**	**Annotation**
AQR24_RS09505	DUF1178 family protein
group_2427	succinate dehydrogenase, cytochrome b556 subunit
HXP35_07870	thioredoxin family protein
C2I38_RS01260	hypothetical protein
HXP36_02375	H-NS histone family protein
C2I38_RS10340	xylose isomerase
HXP34_01665	hypothetical protein
C2I38_RS10910	thiol-disulfide oxidoreductase DCC family protein
EIH10_RS16290	glutaredoxin family protein
RALB5_RS04680	ShET2/EspL2 family type III secretion system effector toxin
C2L97_RS23440	hypothetical protein
AQR21_RS18415	hypothetical protein
HXP35_16820	flavoprotein
C2I38_RS20925	MFS transporter
RALB5_RS06125	hypothetical protein
group_3009	type III secretion system cytoplasmic ring protein SctQ
AQR21_RS21620	leucine-rich repeat domain-containing protein
HXP35_18215	nuclear transport factor 2 family protein
group_3057	Flp pilus assembly protein CpaB
HXP35_19295	collagen-like triple helix repeat-containing protein
EIH10_RS11370	alpha/beta hydrolase
C2I38_RS20205	DUF2239 family protein
C2L97_RS01235	hypothetical protein
C2I38_RS17795	hypothetical protein
HXP34_20945	hypothetical protein
HXP37_21605	type III effector protein
EIH09_RS24305	hypothetical protein
RALW1_RS15640	pilus assembly protein
EIH13_RS22915	ImmA/IrrE family metallo-endopeptidase

Moko typical	
**Gene**	**Annotation**
HXP37_02860	hypothetical protein
EIH14_RS15855	hypothetical protein
C2I38_RS00110	hypothetical protein
EIH13_RS20240	hypothetical protein
UW163_RS07975	UDP-glucuronosyltransferase
EIH13_RS05670	type II toxin-antitoxin system HipA family toxin
EIH11_RS06885	hypothetical protein
C2L97_RS09250	ribbon-helix-helix protein, CopG family
UW163_RS15335	PAS domain-containing sensor histidine kinase
EIH14_RS08500	hypothetical protein
EIH11_RS02375	hypothetical protein
EIH11_RS02380	MFS transporter
C2I38_RS23590	calcium-binding protein
EIH09_RS07555	hypothetical protein
RALW1_RS24825	hypothetical protein
EIH09_RS24295	hypothetical protein
EIH09_RS07015	AraC family transcriptional regulator
EIH14_RS09315	MarR family transcriptional regulator
RALW1_RS10450	hypothetical protein
RSPO_RS20495	hypothetical protein
UW163_RS25955	hypothetical protein
EIH14_RS03825	DJ-1/PfpI family protein
EIH13_RS02920	glycoside hydrolase family 28 protein
C2L97_RS22335	hypothetical protein
EIH13_RS12670	antibiotic biosynthesis monooxygenase
RSPO_RS19590	hypothetical protein
C2L97_RS19555	hypothetical protein
C2L97_RS19520	type III effector protein
EIH14_RS09605	hypothetical protein

Moko typical	
**Gene**	**Annotation**
RALW1_RS25555	hypothetical protein
RALW1_RS14905	hypothetical protein
C2I38_RS17660	GNAT family N-acetyltransferase
EIH11_RS01080	hypothetical protein
RSPO_RS19545	RraA family protein
EIH11_RS09895	type II toxin-antitoxin system ParD family antitoxin
RALW1_RS04755	amino acid ABC transporter permease
RALB5_RS18845	SAM-dependent DNA methyltransferase
HXP35_00615	DEAD/DEAH box helicase family protein
AQR21_RS16675	hypothetical protein
AQR21_RS09070	c-type cytochrome
EIH10_RS02240	ABC transporter substrate-binding protein
EIH10_RS12510	hypothetical protein
RALGR_RS17335	hypothetical protein
EIH12_RS14590	response regulator transcription factor
RALB5_RS05155	CDGSH iron-sulfur domain-containing protein
RALB5_RS05070	hypothetical protein
EIH10_RS04530	hypothetical protein
AQR21_RS23020	hypothetical protein
HXP34_05290	hypothetical protein
C2I38_RS05465	hypothetical protein
AQR24_RS05455	class I SAM-dependent methyltransferase
C2I38_RS05975	TetR/AcrR family transcriptional regulator
AQR24_RS03620	DUF1232 domain-containing protein
EIH10_RS08890	antibiotic biosynthesis monooxygenase
HXP35_06535	DUF3106 domain-containing protein
RALGR_RS06640	hypothetical protein
AQR24_RS20455	(2Fe-2S)-binding protein
RALGR_RS06785	hypothetical protein

Moko typical	
**Gene**	**Annotation**
HXP35_07595	universal stress protein
RALB5_RS12650	MerR family transcriptional regulator
group_4227	homoprotocatechuate degradation operon regulator HpaR
AQR21_RS09985	nitroreductase
C2L97_RS08265	hypothetical protein
pncA	bifunctional nicotinamidase/pyrazinamidase
group_4268	ergothioneine biosynthesis protein EgtB
EIH10_RS14005	nitroreductase family protein
EIH10_RS13995	hypothetical protein
RALGR_RS19875	membrane protein
RALGR_RS06270	hypothetical protein
HXP34_14610	GNAT family N-acetyltransferase
EIH10_RS17660	DUF1328 domain-containing protein
HXP35_15110	hypothetical protein
RALGR_RS21415	transcriptional regulator
C2I38_RS24100	hypothetical protein
HXP35_15375	GNAT family N-acetyltransferase
EIH12_RS20735	cupin domain-containing protein
AQR21_RS11990	hypothetical protein
EIH10_RS11265	hypothetical protein
C2I38_RS23575	GNAT family N-acetyltransferase
RALB5_RS05460	nuclear transport factor 2 family protein
HXP34_15705	nucleotidyltransferase family protein
RALB5_RS01200	DUF2894 domain-containing protein
HXP35_18040	SDR family oxidoreductase
RALB5_RS06090	LuxR family transcriptional regulator
RALGR_RS07045	hypothetical protein
AQR21_RS16240	type III effector protein
C2L97_RS22660	cold-shock protein

Moko typical	
**Gene**	**Annotation**
HXP35_19215	Gfo/Idh/MocA family oxidoreductase
group_4708	trehalose-phosphatase
RALB5_RS01585	aquaporin family protein
HXP35_19455	response regulator transcription factor
EIH12_RS04740	helix-turn-helix transcriptional regulator
AQR21_RS10365	hypothetical protein
EIH10_RS03650	hypothetical protein
C2L97_RS17620	hypothetical protein
EIH12_RS21040	NAD(P)-binding protein
HXP35_21290	siderophore-interacting protein
AQR24_RS18805	hypothetical protein
EIH10_RS21340	MbtH family protein
AQR24_RS05435	hypothetical protein
EIH10_RS14745	DUF3426 domain-containing protein
EIH09_RS13520	pilin
RALW1_RS25920	hypothetical protein
RALW1_RS06670	SRPBCC domain-containing protein
EIH11_RS07815	hypothetical protein
RSPO_RS16000	hypothetical protein
EIH09_RS11610	HD domain-containing protein
EIH11_RS12995	hypothetical protein
EIH09_RS15115	hypothetical protein
C2I38_RS03900	glycosyltransferase
HXP37_07990	hypothetical protein
HXP37_09045	hypothetical protein
C2I38_RS04985	cytochrome c4
UW163_RS09655	lytic transglycosylase domain-containing protein
UW163_RS10085	hypothetical protein
EIH11_RS06880	hypothetical protein

Moko typical	
**Gene**	**Annotation**
EIH11_RS17505	threonine synthase
UW163_RS15345	TraR/DksA family transcriptional regulator
EIH14_RS07175	hypothetical protein
EIH11_RS02290	ParB-like nuclease domain-containing protein
RALW1_RS21505	FAD-binding oxidoreductase
C2L97_RS23710	RidA family protein
EIH09_RS03465	helix-turn-helix domain-containing protein
C2I38_RS24805	hypothetical protein
EIH11_RS00850	hypothetical protein
C2I38_RS23490	hypothetical protein
UW163_RS24875	hypothetical protein
EIH14_RS17315	hypothetical protein
EIH14_RS17590	hypothetical protein
C2I38_RS18900	YqaJ viral recombinase family protein
EIH11_RS03225	hypothetical protein
EIH14_RS04265	LysR family transcriptional regulator
C2I38_RS21460	hypothetical protein
C2L97_RS21650	SMP-30/gluconolactonase/LRE family protein
HXP37_19920	hypothetical protein
RALW1_RS13290	hypothetical protein
RSPO_RS21650	copper uptake system-associated protein
UW163_RS21190	hypothetical protein
RSPO_RS19245	hypothetical protein
C2I38_RS19755	chemotaxis protein
UW163_RS21635	hypothetical protein
UW163_RS26025	hypothetical protein
EIH11_RS22255	heavy metal sensor histidine kinase
C2L97_RS18315	heavy metal response regulator transcription factor
EIH09_RS08180	TIGR02677 family protein

Moko typical	
**Gene**	**Annotation**
C2L97_RS17580	TIGR02678 family protein
EIH13_RS01345	TIGR02680 family protein
RALW1_RS13030	hypothetical protein
EIH13_RS01330	hypothetical protein
UW163_RS23005	hypothetical protein
C2L97_RS17205	hypothetical protein
EIH13_RS18195	type III effector protein
RSPO_RS16485	SRPBCC family protein
RALB5_RS04675	metallophosphoesterase
EIH10_RS20875	response regulator transcription factor
EIH09_RS21700	hypothetical protein
AQR21_RS05690	rRNA pseudouridine synthase
HXP34_02250	cytochrome c5 family protein
EIH10_RS06190	hypothetical protein
RALB5_RS23125	sulfotransferase
EIH11_RS22200	1,4-beta-cellobiosidase
HXP37_15320	Type III effector protein (Skwp 4)
UW163_RS18385	DUF1801 domain-containing protein
C2L97_RS01280	phage tail tape measure protein
prpC	2-methylcitrate synthase
group_91	2-methylcitrate synthase
HXP34_18675	LysE family translocator
RALB5_RS26425	hypothetical protein
EIH10_RS08180	NUDIX hydrolase
C2I38_RS01350	lysozyme
EIH10_RS17405	AWR family protein
EIH12_RS20285	hypothetical protein
RSPO_RS25005	hypothetical protein
RALB5_RS16630	hypothetical protein

Moko typical	
**Gene**	**Annotation**
RSPO_RS18705	prolyl oligopeptidase family serine peptidase
RALW1_RS23900	hypothetical protein
C2I38_RS01200	hypothetical protein
group_2551	pca operon transcription factor PcaQ
AQR24_RS02665	helix-turn-helix domain-containing protein
HXP34_11610	type IV pilin protein
HXP34_11760	META domain-containing protein
RALGR_RS21135	hypothetical protein
EIH12_RS07565	hypothetical protein
HXP35_13245	hypothetical protein
AQR21_RS09670	acylphosphatase
C2I38_RS16800	hypothetical protein
EIH10_RS11545	hypothetical protein
RALGR_RS17580	type III effector protein
RALB5_RS16945	GNAT family N-acetyltransferase
RALW1_RS24225	hypothetical protein
UW163_RS12880	type II toxin-antitoxin system RelE/ParE family toxin
UW163_RS18145	hypothetical protein
C2I38_RS19190	metallophosphoesterase
EIH13_RS15940	alkene reductase
cfa	cyclopropane fatty acyl phospholipid synthase
EIH10_RS22410	SRPBCC domain-containing protein
UW163_RS25530	hypothetical protein
RSPO_RS15275	acylphosphatase
RALGR_RS24120	hypothetical protein
C2I38_RS19810	hypothetical protein
C2L97_RS18775	DUF3348 domain-containing protein
AQR24_RS00420	LysE family translocator
HXP34_01890	tryptophan 2-monooxygenase oxidoreductase

Moko typical	
**Gene**	**Annotation**
RALB5_RS24120	lipoprotein
HXP35_06525	triacylglycerol lipase
HXP35_08735	GNAT family N-acetyltransferase
AQR24_RS00815	pirin family protein
EIH10_RS03145	bacteriohemerythrin
AQR21_RS05990	PilW family protein
EIH12_RS11210	pilus assembly protein PilY
HXP35_11790	type IV pilin protein
RALGR_RS02175	prepilin-type N-terminal cleavage/methylation domain-containing protein
RALB5_RS17615	pilus assembly protein
RALGR_RS25140	DUF4288 domain-containing protein
AQR24_RS21645	paraquat-inducible membrane protein A
HXP34_12455	hypothetical protein
AQR21_RS14420	FmdB family transcriptional regulator
RALGR_RS03995	gluconokinase
C2L97_RS14460	acyl carrier protein
AQR24_RS04290	acyl-CoA synthetase
AQR24_RS04150	hypothetical protein
RALB5_RS12245	ABC transporter
EIH12_RS19160	DUF4430 domain-containing protein
C2L97_RS15750	GYD domain-containing protein
AQR21_RS11380	TfoX/Sxy family protein
HXP34_14900	hypothetical protein
AQR24_RS27995	hypothetical protein
AQR21_RS07865	hypothetical protein
EIH10_RS07300	alanine-rich signal peptide protein
C2I38_RS21090	YciI family protein
AQR21_RS05065	ABC transporter ATP-binding protein
C2L97_RS19800	FMN-dependent NADH-azoreductase

Moko typical	
**Gene**	**Annotation**
RALB5_RS20970	TetR/AcrR family transcriptional regulator
C2L97_RS17875	hypothetical protein
HXP34_20760	DUF3005 domain-containing protein
AQR24_RS09935	hypothetical protein
RALGR_RS02815	hypothetical protein
EIH12_RS18695	hypothetical protein
C2I38_RS20165	filamentous hemagglutinin N-terminal domain-containing protein
RALW1_RS11235	NAD(P)H-binding protein
C2I38_RS19265	hypothetical protein
EIH09_RS11210	hypothetical protein
RSPO_RS12700	PilW family protein
EIH14_RS03160	hypothetical protein
EIH09_RS18560	hypothetical protein
EIH11_RS12330	polymerase
EIH13_RS16165	beta-ketothiolase
RALW1_RS08960	type III effector protein
EIH11_RS16835	ankyrin repeat domain-containing protein
RSPO_RS15235	hypothetical protein
UW163_RS03280	hypothetical protein
UW163_RS03465	hypothetical protein
RSPO_RS25255	hypothetical protein
HXP37_09675	hypothetical protein
C2L97_RS07725	hypothetical protein
HXP37_21735	hypothetical protein
HXP37_21775	F-box protein
C2I38_RS19355	TetR/AcrR family transcriptional regulator
EIH11_RS00125	hypothetical protein
EIH10_RS12765	phosphatidate cytidylyltransferase
HXP34_13725	DUF3108 domain-containing protein

Moko typical	
**Gene**	**Annotation**
UW163_RS16160	MFS transporter
AQR24_RS02785	helix-hairpin-helix domain-containing protein
C2I38_RS02000	response regulator
C2L97_RS00075	hypothetical protein
RALW1_RS21465	AAA family ATPase
RALW1_RS26110	hypothetical protein
HXP34_01560	phage terminase large subunit family protein
RALFB_RS21510	flavin reductase family protein
C2I38_RS13815	AAA family ATPase
RALGR_RS21310	ankyrin repeat domain-containing protein
EIH10_RS11520	GNAT family N-acetyltransferase
AQR21_RS01065	hypothetical protein
UW163_RS00110	hypothetical protein
HXP37_16180	hypothetical protein
RALW1_RS00105	hypothetical protein
C2L97_RS01325	membrane protein
EIH12_RS04515	hypothetical protein
RALW1_RS24620	hypothetical protein
EIH10_RS08780	uracil-DNA glycosylase
UW163_RS05405	H-NS histone family protein
EIH12_RS18800	cytochrome c
EIH10_RS13765	hypothetical protein
C2I38_RS17930	hypothetical protein
C2L97_RS12275	hypothetical protein
EIH09_RS11405	hypothetical protein
EIH13_RS14415	hypothetical protein
EIH09_RS24150	hypothetical protein
UW163_RS01660	recombinase
UW163_RS04240	sce7725 family protein

Moko typical	
**Gene**	**Annotation**
C2L97_RS00055	sce7726 family protein
C2I38_RS12220	YOPP/AvrRxv family protein
C2I38_RS01375	ABC transporter substrate-binding protein
RALW1_RS10680	hypothetical protein
EIH13_RS10530	queuosine precursor transporter
EIH09_RS11760	phosphohydrolase
RSPO_RS07280	DotA/TraY family protein
EIH13_RS21000	hypothetical protein
UW163_RS17795	hypothetical protein
RSPO_RS25540	hypothetical protein
RSPO_RS20260	alpha/beta hydrolase
EIH13_RS01955	AAA family ATPase
UW163_RS24985	hypothetical protein
UW163_RS22030	TolC family protein
EIH09_RS19495	hypothetical protein
C2I38_RS17330	hypothetical protein
HXP37_23385	hypothetical protein
C2I38_RS24615	hypothetical protein
EIH09_RS19860	hypothetical protein
UW163_RS23230	helix-turn-helix transcriptional regulator
UW163_RS01515	DEAD/DEAH box helicase
C2L97_RS24050	hypothetical protein
EIH12_RS09640	type III effector protein (Skwp5)
C2L97_RS08930	hypothetical protein
RALB5_RS27640	hypothetical protein
C2I38_RS20535	glyoxalase/bleomycin resistance/dioxygenase family protein
RALB5_RS05015	DUF1311 domain-containing protein
EIH12_RS10285	hypothetical protein
HXP35_01595	head decoration protein

Moko typical	
**Gene**	**Annotation**
EIH12_RS09725	hypothetical protein
HXP34_19950	hypothetical protein
RALW1_RS22515	hypothetical protein
EIH09_RS22185	hypothetical protein
C2I38_RS12435	glycine hydroxymethyltransferase
popP1	type III secretion system YopJ family effector PopP1
C2I38_RS13415	hypothetical protein
RALW1_RS18690	hypothetical protein
RSPO_RS25200	hypothetical protein
EIH09_RS23030	hypothetical protein
C2L97_RS13715	recombinase family protein
EIH09_RS23040	hypothetical protein
C2I38_RS24550	hypothetical protein
C2L97_RS13740	hypothetical protein
C2L97_RS13745	hypothetical protein
EIH09_RS13585	site-specific integrase
C2L97_RS13835	hypothetical protein
C2I38_RS13915	helix-turn-helix transcriptional regulator
C2L97_RS13850	hypothetical protein
HXP37_01595	hypothetical protein
C2I38_RS16320	phospholipase
EIH09_RS06560	SAVED domain-containing protein
EIH09_RS06565	nucleotidyltransferase
HXP37_04175	ThiF family adenylyltransferase
EIH09_RS06575	hypothetical protein
EIH09_RS06580	3'-5' exonuclease
EIH13_RS10535	helix-turn-helix domain-containing protein
UW163_RS05530	hypothetical protein
C2I38_RS01430	hypothetical protein

Moko typical	
**Gene**	**Annotation**
HXP37_05555	hypothetical protein
EIH09_RS24455	hypothetical protein
C2L97_RS01570	hypothetical protein
EIH13_RS09625	tyrosine-type recombinase/integrase
RSPO_RS04595	hypothetical protein
C2L97_RS04695	DUF3631 domain-containing protein
UW163_RS08795	hypothetical protein
EIH09_RS08790	hypothetical protein
EIH09_RS08775	hypothetical protein
C2L97_RS05480	hypothetical protein
C2I38_RS05540	hypothetical protein
C2I38_RS05580	hypothetical protein
RALW1_RS05355	cysteine hydrolase
UW163_RS10175	MFS transporter
C2I38_RS06145	GntR family transcriptional regulator
C2I38_RS06265	DUF1851 domain-containing protein
RALW1_RS08290	tyrosine-type recombinase/integrase
EIH13_RS19630	hypothetical protein
EIH13_RS19635	AlpA family phage regulatory protein
RSPO_RS23940	hypothetical protein
UW163_RS10330	hypothetical protein
UW163_RS10335	hypothetical protein
RALW1_RS08320	hypothetical protein
RALW1_RS08325	hypothetical protein
EIH13_RS19665	hypothetical protein
RALW1_RS20730	hypothetical protein
UW163_RS11435	site-specific integrase
RSPO_RS07250	hypothetical protein
HXP37_11485	hypothetical protein

Moko typical	
**Gene**	**Annotation**
EIH09_RS20510	hypothetical protein
UW163_RS11465	hypothetical protein
EIH13_RS22675	hypothetical protein
HXP37_11520	hypothetical protein
C2L97_RS07445	exonuclease domain-containing protein
HXP37_11530	hypothetical protein
RALW1_RS07380	Fis family transcriptional regulator
RSPO_RS25110	hypothetical protein
RSPO_RS11875	ABC transporter substrate-binding protein
UW163_RS24765	phosphoribosyltransferase
EIH09_RS20535	hypothetical protein
HXP37_16205	hypothetical protein
EIH09_RS21090	DUF4935 domain-containing protein
EIH09_RS22270	DHA2 family efflux MFS transporter permease subunit
RALW1_RS23235	non-ribosomal peptide synthetase
RSPO_RS25655	hypothetical protein
RSPO_RS18445	hypothetical protein
EIH13_RS01940	START domain-containing protein
C2I38_RS18935	oxidoreductase
C2L97_RS18840	hypothetical protein
C2I38_RS18950	hypothetical protein
EIH09_RS07090	HEAT repeat domain-containing protein
RSPO_RS18505	deaminase reductase
C2L97_RS18940	hypothetical protein
RALW1_RS01405	SDR family oxidoreductase
HXP37_18480	flavodoxin
C2L97_RS19040	alpha/beta hydrolase
C2L97_RS19045	alpha/beta fold hydrolase
EIH09_RS23705	cupin domain-containing protein

Moko typical	
**Gene**	**Annotation**
RSPO_RS18645	MFS transporter
HXP37_18505	aldo/keto reductase
HXP37_18510	LysR family transcriptional regulator
EIH09_RS23670	hypothetical protein
EIH13_RS22065	hypothetical protein
EIH13_RS15980	PAAR domain-containing protein
HXP37_19870	hypothetical protein
C2I38_RS22220	hypothetical protein
C2I38_RS20800	hypothetical protein
EIH09_RS21740	hypothetical protein
EIH13_RS12700	DUF1911 domain-containing protein
HXP37_21235	hypothetical protein
C2L97_RS20075	hypothetical protein
UW163_RS26000	hypothetical protein
RALW1_RS25560	DUF2933 domain-containing protein
EIH09_RS23270	hypothetical protein
EIH09_RS23260	hypothetical protein
RSPO_RS17980	copper resistance system multicopper oxidase
EIH09_RS23235	copper resistance protein B
copC	copper homeostasis periplasmic binding protein CopC
copD	copper homeostasis membrane protein CopD
RALW1_RS21975	MFS transporter
EIH13_RS18950	oxidoreductase
EIH09_RS23200	TetR/AcrR family transcriptional regulator
C2L97_RS18260	hypothetical protein
EIH13_RS05290	hypothetical protein
C2L97_RS18070	DUF4279 domain-containing protein
EIH09_RS19500	hypothetical protein
RSPO_RS24355	hypothetical protein

Moko typical	
**Gene**	**Annotation**
UW163_RS22530	UDP-glucuronosyltransferase
C2L97_RS17570	TIGR02679 family protein
C2I38_RS24640	hypothetical protein
EIH09_RS19845	hypothetical protein
RALW1_RS26175	hypothetical protein
C2I38_RS17115	hypothetical protein
group_6099	site-specific tyrosine recombinase XerC
EIH09_RS19880	toprim domain-containing protein
HXP37_23440	hypothetical protein
C2I38_RS17090	hypothetical protein
EIH13_RS18120	hypothetical protein
HXP37_23565	hypothetical protein
HXP37_23570	hypothetical protein
C2L97_RS16875	hypothetical protein
RALW1_RS25465	tyrosine-type recombinase/integrase
HXP37_23775	hypothetical protein
EIH09_RS24450	hypothetical protein
RSPO_RS18715	putative type III effector protein
C2I38_RS19260	hypothetical protein
EIH12_RS02765	HAMP domain-containing histidine kinase
RALB5_RS11420	dipeptidase
EIH09_RS23300	IS3 family transposase
RALGR_RS13455	flavodoxin family protein
UW163_RS17805	AraC family transcriptional regulator
RALB5_RS11355	C40 family peptidase
RSPO_RS07325	N-6 DNA methylase
EIH10_RS18505	flagellar hook-length control protein FliK
UW163_RS23590	hypothetical protein
HXP37_22155	copper-translocating P-type ATPase

Moko typical	
**Gene**	**Annotation**
RALW1_RS25580	barnase inhibitor
group_8481	elongation factor Tu
group_8482	elongation factor Tu
UW163_RS16195	lysozyme
EIH10_RS04535	metal-binding protein
EIH13_RS22590	hypothetical protein
RSPO_RS12010	major capsid protein
EIH12_RS10150	hypothetical protein
RALB5_RS13085	leucine-rich repeat protein
C2L97_RS00585	hypothetical protein
C2L97_RS17495	diguanylate cyclase
HXP35_08000	hypothetical protein
group_284	pyruvate kinase
HXP34_17555	hypothetical protein
C2L97_RS21440	hypothetical protein
EIH09_RS00160	hypothetical protein
EIH13_RS06910	hypothetical protein
EIH14_RS23180	hypothetical protein
C2I38_RS13490	hypothetical protein
RSPO_RS25225	hypothetical protein
EIH09_RS00050	site-specific DNA-methyltransferase
C2I38_RS18985	hypothetical protein
HXP37_19345	hypothetical protein
C2I38_RS13765	tyrosine-type recombinase/integrase
C2I38_RS19290	hypothetical protein
C2L97_RS17195	FimV family protein
EIH12_RS21000	H-NS histone family protein
HXP37_22695	hemolysin-type protein
RSPO_RS12085	site-specific DNA-methyltransferase

Moko typical	
**Gene**	**Annotation**
RSPO_RS13205	type III effector protein
HXP37_01580	hypothetical protein
HXP37_01935	tartrate dehydrogenase
RSPO_RS11985	hypothetical protein
EIH13_RS22555	hypothetical protein
RSPO_RS07265	hypothetical protein
HXP37_11510	hypothetical protein
HXP37_16960	hypothetical protein
C2L97_RS18220	hypothetical protein
C2I38_RS24620	hypothetical protein
UW163_RS05490	DUF1484 family protein
HXP36_14075	helix-turn-helix transcriptional regulator
EIH13_RS10470	helix-turn-helix transcriptional regulator
RALW1_RS26355	hypothetical protein
EIH11_RS03360	protein phosphatase
HXP37_22275	hypothetical protein
UW163_RS05545	ShlB/FhaC/HecB family hemolysin secretion/activation protein
HXP37_22105	hypothetical protein
RALB5_RS17490	LysR family transcriptional regulator
EIH10_RS23615	hypothetical protein
EIH12_RS22250	acyltransferase domain-containing protein
EIH12_RS12160	GNAT family N-acetyltransferase
HXP35_18650	hypothetical protein
HXP34_19595	phospholipase D family protein
RALGR_RS26050	hypothetical protein
UW163_RS00115	hypothetical protein
HXP36_02595	ShlB/FhaC/HecB family hemolysin secretion/activation protein
C2L97_RS13320	IS3 family transposase
HXP36_20040	hypothetical protein

Moko typical	
**Gene**	**Annotation**
HXP35_11550	glycoside hydrolase family 3 protein
C2I38_RS12305	hypothetical protein
HXP37_21740	type III effector protein skwp2
RSPO_RS01260	hypothetical protein
C2I38_RS12175	hypothetical protein
RSPO_RS24940	hypothetical protein
EIH09_RS16135	hypothetical protein
UW163_RS24725	helix-turn-helix domain-containing protein
RALW1_RS25705	DUF1484 domain-containing protein
C2I38_RS23485	hypothetical protein
EIH12_RS13345	hypothetical protein
AQR21_RS07545	molecular chaperone DnaJ
HXP36_14100	hypothetical protein
RALB5_RS27495	hypothetical protein
C2L97_RS01860	cellulose 1,4-beta-cellobiosidase
HXP35_02235	hypothetical protein
RALB5_RS27760	hypothetical protein
EIH12_RS03620	FimV family protein
HXP35_07000	hypothetical protein
HXP34_14770	spherulation-specific family 4 protein
AQR24_RS05360	methyltransferase domain-containing protein
C2I38_RS06240	filamentous hemagglutinin, protein
UW163_RS00100	DUF3489 domain-containing protein
UW163_RS26265	hypothetical protein
HXP37_16170	hypothetical protein
EIH09_RS00185	hypothetical protein
RALW1_RS25015	AAA family ATPase
RALW1_RS12375	cyclic nucleotide-binding domain-containing protein
EIH09_RS00165	hypothetical protein

Moko typical	
**Gene**	**Annotation**
EIH09_RS00040	hypothetical protein
EIH13_RS10550	hypothetical protein
C2I38_RS07530	hypothetical protein
RALB5_RS07950	YeiH family putative sulfate export transporter
EIH09_RS24135	ParB N-terminal domain-containing protein
C2L97_RS17610	anti-ECFsigma factor, ChrR
RALGR_RS02845	HutD family protein
C2L97_RS12120	hypothetical protein
C2L97_RS21740	hypothetical protein
C2L97_RS12050	MFS transporter
AQR24_RS21660	membrane integrity-associated transporter subunit PqiC
AQR24_RS17755	SDR family NAD(P)-dependent oxidoreductase
C2I38_RS01165	hypothetical protein
C2L97_RS01285	hypothetical protein
RALB5_RS03930	hypothetical protein
C2L97_RS12975	carbon-nitrogen hydrolase family protein
AQR21_RS05325	hypothetical protein
AQR21_RS13120	hypothetical protein
EIH12_RS13915	hypothetical protein
RALGR_RS20680	hypothetical protein
C2L97_RS12215	hypothetical protein
RALW1_RS13610	hemagglutinin repeat-containing protein
C2L97_RS02530	IS1595 family transposase
EIH13_RS24435	hypothetical protein
C2I38_RS01235	hypothetical protein
C2L97_RS19195	hypothetical protein
AQR21_RS25815	hypothetical protein
C2I38_RS24575	hypothetical protein
C2L97_RS13735	IS3 family transposase

Moko typical	
**Gene**	**Annotation**
RSPO_RS19575	IS3 family transposase
C2I38_RS01445	IS5 family transposase
HXP37_18555	IS5 family transposase
C2I38_RS12275	phage portal protein
RALGR_RS19180	hypothetical protein
RALGR_RS19185	SCO family protein
C2L97_RS20690	hypothetical protein
HXP35_15160	hypothetical protein
UW163_RS11675	IS1595 family transposase
C2L97_RS17130	IS1595 family transposase
C2L97_RS06205	IS3 family transposase
RSPO_RS13210	IS3 family transposase
EIH09_RS11805	hypothetical protein
RALGR_RS12495	2-dehydropantoate 2-reductase
C2I38_RS18170	chromate resistance protein
C2L97_RS13680	IS3 family transposase
UW163_RS00185	IS3 family transposase
C2I38_RS19095	IS3 family transposase
C2L97_RS16865	IS3 family transposase
UW163_RS24635	IS5 family transposase
C2I38_RS09110	IS5 family transposase
UW163_RS24855	IS5 family transposase
C2L97_RS23425	IS5 family transposase
RSPO_RS24365	IS5 family transposase
C2I38_RS16960	IS5 family transposase
RSPO_RS07725	ATP-binding protein
RALB5_RS06630	SDR family NAD(P)-dependent oxidoreductase
C2L97_RS12220	hypothetical protein
HXP37_10360	hypothetical protein

Moko typical	
**Gene**	**Annotation**
AQR21_RS22000	transposase
C2L97_RS07410	IS3 family transposase
C2I38_RS13780	IS3 family transposase
C2L97_RS20685	hemagglutinin repeat-containing protein
C2I38_RS12270	S49 family peptidase
C2L97_RS07405	hypothetical protein
C2L97_RS17775	hypothetical protein
C2L97_RS22780	ATP-binding protein
C2L97_RS05675	glutamate/aspartate ABC transporter substrate-binding protein
C2I38_RS08105	hypothetical protein
C2L97_RS05955	hypothetical protein
EIH10_RS23565	hypothetical protein
C2I38_RS01435	IS3 family transposase
C2L97_RS00455	IS5 family transposase
RSPO_RS24550	IS5 family transposase
C2L97_RS00070	IS5 family transposase
C2I38_RS06315	IS5 family transposase
RSPO_RS24110	IS5 family transposase
C2L97_RS17970	IS5 family transposase
RSPO_RS24485	IS5 family transposase
RSPO_RS24655	IS5 family transposase
UW163_RS01180	IS21 family transposase
RSPO_RS12155	AAA family ATPase
group_164	type VI secretion system tip protein VgrG
UW163_RS10350	hypothetical protein
UW163_RS23645	RNA ligase family protein
C2L97_RS20065	hypothetical protein
RALW1_RS24665	hemolysin-type protein
C2L97_RS19455	YOPP/AvrRxv family protein

Moko typical	
**Gene**	**Annotation**
C2I38_RS13445	AAA family ATPase
AQR21_RS13035	DUF898 domain-containing protein
UW163_RS11530	IS3 family transposase
RSPO_RS24135	IS5 family transposase
RSPO_RS12160	IS21 family transposase
C2I38_RS16710	AAA family ATPase

Moko and Bugtok disease	
Gene	Annotation
HXP35_00525	cellobiose phosphorylase
AQR24_RS19785	diguanylate cyclase
HXP35_14755	TonB-dependent receptor
RALFB_RS07690	tyrosine-type recombinase/integrase
RSPO_RS19700	GGDEF domain-containing protein
RALFB_RS02865	hypothetical protein
C2I33_RS23970	TonB-dependent receptor
EIH11_RS06270	LysR family transcriptional regulator
RSPO_RS08930	MoxR family ATPase
RSMK_RS24590	xanthine dehydrogenase family protein molybdopterin-binding subunit
EIH12_RS04460	nicotinate phosphoribosyltransferase
AQR24_RS11595	EAL domain-containing protein
RALW1_RS18940	type II toxin-antitoxin system HipA family toxin
AQR21_RS11265	ABC transporter substrate-binding protein
HXP37_09800	hypothetical protein
EIH10_RS06805	Patatin
EIH14_RS15940	hypothetical protein
yidD	membrane protein insertion efficiency factor YidD
AQR21_RS07320	MFS transporter
EIH12_RS09045	TetR family transcriptional regulator
C2I33_RS02495	efflux RND transporter periplasmic adaptor subunit

Moko typical	
**Gene**	**Annotation**
EIH11_RS07690	efflux transporter outer membrane subunit
rpsU	30S ribosomal protein S21
EIH11_RS07670	phosphotyrosine protein phosphatase
rnpA	ribonuclease P protein component
rpmH	50S ribosomal protein L34
pelG	exopolysaccharide Pel transporter PelG
RALW1_RS10060	hypothetical protein
AQR21_RS08495	diguanylate cyclase
treS	maltose alpha-D-glucosyltransferase
UW163_RS23555	LysR family transcriptional regulator
EIH12_RS15545	TrbI/VirB10 family protein
mnmE	tRNA uridine-5-carboxymethylaminomethyl(34) synthesis GTPase MnmE
HXP36_19790	porin
EIH12_RS12220	gamma-glutamylcyclotransferase
putA	trifunctional transcriptional regulator/proline dehydrogenase/L-glutamate gamma-semialdehyde dehydrogenase
fliF	flagellar basal body M-ring protein FliF
EIH10_RS21110	hypothetical protein
UW163_RS13170	enoyl-CoA hydratase/isomerase family protein
tssB	type VI secretion system contractile sheath small subunit
HXP34_00820	OmpW family protein
EIH13_RS16980	DUF1175 domain-containing protein
C2I33_RS20495	hypothetical protein
HXP36_16515	OsmC family protein
EIH11_RS08485	DUF2167 domain-containing protein
EIH13_RS12945	multidrug efflux RND transporter permease subunit
C2I33_RS16645	response regulator transcription factor
HXP36_24445	diguanylate cyclase
EIH12_RS16800	cation acetate symporter
tssK	type VI secretion system baseplate subunit TssK

Moko typical	
**Gene**	**Annotation**
RALB5_RS20565	HlyD family efflux transporter periplasmic adaptor subunit
RALGR_RS09520	iron-containing alcohol dehydrogenase
sdhC	succinate dehydrogenase, cytochrome b556 subunit
RALFB_RS25025	glutamate/aspartate ABC transporter substrate-binding protein
EIH14_RS08185	hypothetical protein
EIH14_RS03590	hypothetical protein
EIH14_RS16940	GNAT family N-acetyltransferase
AQR24_RS08440	aldehyde dehydrogenase
RALB5_RS03420	sugar ABC transporter ATP-binding protein
group_1477	4-hydroxy-tetrahydrodipicolinate synthase
C2I33_RS10010	LysR family transcriptional regulator
EIH10_RS11785	PIN domain-containing protein
RALFB_RS06240	DUF4153 domain-containing protein
C2I33_RS09800	HutD family protein
RSPO_RS05975	DUF1232 domain-containing protein
C2I33_RS11080	MerR family transcriptional regulator
C2I33_RS05705	GNAT family N-acetyltransferase
RALW1_RS14065	C40 family peptidase
HXP36_12750	thiol-disulfide oxidoreductase DCC family protein
RALCI_RS08940	YeiH family putative sulfate export transporter
C2I33_RS23565	transcriptional regulator
RSMK_RS14675	MFS transporter
EIH09_RS05080	hypothetical protein
HXP37_20200	ABC transporter ATP-binding protein
EIH14_RS01070	Gfo/Idh/MocA family oxidoreductase
RSPO_RS25490	hypothetical protein
EIH09_RS12755	hypothetical protein
RALCI_RS02110	DUF333 domain-containing protein
EIH11_RS04050	2-dehydropantoate 2-reductase

Moko typical	
**Gene**	**Annotation**
C2I33_RS10565	ABC transporter ATPase
EIH09_RS08205	NAD(P)-binding protein
C2I33_RS22120	hypothetical protein
RALFB_RS22720	4'-phosphopantetheinyl transferase superfamily protein
RALFB_RS08440	type III effector protein
RSPO_RS20100	collagen-like triple helix repeat-containing protein
HXP34_04160	dihydrodipicolinate synthase family protein
RALB5_RS11710	DUF4382 domain-containing protein
HXP36_17695	NAD(P)H-binding protein
EIH10_RS13230	ribbon-helix-helix protein, CopG family
AQR24_RS16855	type II toxin-antitoxin system RelE/ParE family toxin
RALCI_RS00065	exo-alpha-sialidase
pgaC	poly-beta-1,6 N-acetyl-D-glucosamine synthase
pgaB	poly-beta-1,6-N-acetyl-D-glucosamine N-deacetylase PgaB
EIH12_RS21160	hypothetical protein
EIH12_RS23330	AAA family ATPase
EIH13_RS19525	SAM-dependent DNA methyltransferase
HXP37_05100	c-type cytochrome
HXP37_06045	hypothetical protein
EIH11_RS21535	hypothetical protein
EIH09_RS08880	hypothetical protein
EIH09_RS16490	cob(I)yrinic acid a,c-diamide adenosyltransferase
EIH09_RS18755	SAM-dependent methyltransferase
RALCI_RS03840	antibiotic biosynthesis monooxygenase
group_3300	ergothioneine biosynthesis protein EgtB
C2I33_RS20225	hypothetical protein
EIH13_RS10800	ATP-binding cassette domain-containing protein
RSPO_RS09770	dipeptidase
RSMK_RS08755	helix-turn-helix domain-containing protein

Moko typical	
**Gene**	**Annotation**
RALCI_RS09745	hypothetical protein
EIH13_RS21180	hypothetical protein
EIH11_RS02260	cupin domain-containing protein
EIH11_RS02460	hypothetical protein
EIH14_RS04450	YciI family protein
RALCI_RS11935	LysR family transcriptional regulator
RALCI_RS01375	cold-shock protein
HXP36_21875	LuxR family transcriptional regulator
RALCI_RS04100	nuclear transport factor 2 family protein
RALCI_RS07845	siderophore-interacting protein
EIH09_RS19790	sulfotransferase
RALW1_RS20390	MbtH family protein
C2I33_RS18360	hypothetical protein
RSMK_RS12255	DUF3106 domain-containing protein
EIH14_RS09275	DUF3348 domain-containing protein
HXP37_20925	response regulator transcription factor
EIH10_RS11810	RraA family protein
EIH13_RS19080	membrane protein
HXP35_04135	GntR family transcriptional regulator
HXP36_05270	FAD-binding oxidoreductase
AQR24_RS08450	MFS transporter
C2I33_RS19535	FimV family protein
RALFB_RS20170	pyridoxamine 5'-phosphate oxidase family protein
AQR24_RS04525	hypothetical protein
EIH14_RS17565	GGDEF domain-containing protein
C2I33_RS19630	glycoside hydrolase family 28 protein
RALCI_RS22680	response regulator transcription factor
RALW1_RS12105	lipoprotein
EIH14_RS13750	CDGSH iron-sulfur domain-containing protein

Moko typical	
**Gene**	**Annotation**
UW163_RS07070	hypothetical protein
EIH11_RS09145	hypothetical protein
EIH14_RS11340	DUF1178 family protein
hpaR	homoprotocatechuate degradation operon regulator HpaR
RALFB_RS15485	nitroreductase
EIH14_RS18885	metal-dependent hydrolase
C2I33_RS21645	phosphatidate cytidylyltransferase
HXP36_11950	membrane protein
RSPO_RS11050	glutaredoxin family protein
UW163_RS16400	hypothetical protein
RSMK_RS06315	hypothetical protein
RALW1_RS20075	DUF1328 domain-containing protein
EIH14_RS04150	hypothetical protein
HXP37_19820	type III effector protein
group_5566	type I glutamate--ammonia ligase
EIH10_RS12560	L-threonine 3-dehydrogenase
kbl	glycine C-acetyltransferase
AQR21_RS01630	helix-turn-helix transcriptional regulator
RSMK_RS16360	LysE family translocator
RALW1_RS14950	GNAT family N-acetyltransferase
HXP37_13550	uracil-DNA glycosylase
RSPO_RS07420	hypothetical protein
EIH12_RS08615	hypothetical protein
EIH09_RS09215	methylated-DNA--[protein]-cysteine S-methyltransferase
RSPO_RS21070	LysR family transcriptional regulator
EIH12_RS17750	hypothetical protein
EIH14_RS03605	LuxR family transcriptional regulator
EIH10_RS19460	ShlB/FhaC/HecB family hemolysin secretion/activation protein
HXP36_05295	porin

Moko typical	
**Gene**	**Annotation**
HXP37_19285	hypothetical protein
EIH09_RS06505	isoprenylcysteine carboxylmethyltransferase family protein
EIH13_RS05480	hypothetical protein
EIH11_RS08310	ser/threonine protein phosphatase
RALCI_RS01450	DUF2063 domain-containing protein
RSMK_RS06670	hypothetical protein
EIH09_RS03860	hypothetical protein
EIH13_RS03175	nuclear transport factor 2 family protein
AQR21_RS22510	RES family NAD+ phosphorylase
AQR21_RS22500	hypothetical protein
HXP34_21020	transcriptional regulator
EIH10_RS19225	M20/M25/M40 family metallo-hydrolase
HXP36_03160	hypothetical protein
EIH14_RS15465	cytochrome c5 family protein
RALW1_RS03385	(2Fe-2S)-binding protein
EIH09_RS04695	thioredoxin family protein
otsB	trehalose-phosphatase
EIH09_RS12865	alpha/beta hydrolase
RALW1_RS10815	hypothetical protein
C2I33_RS24645	hypothetical protein
HXP37_11200	SPOR domain-containing protein
EIH14_RS08175	membrane protein
RSPO_RS22475	hypothetical protein
EIH09_RS21535	type III effector protein
RALFB_RS18790	hypothetical protein
RALCI_RS07920	hypothetical protein
EIH12_RS15030	hypothetical protein
AQR21_RS22515	hypothetical protein
RALGR_RS02485	hypothetical protein

Moko typical	
**Gene**	**Annotation**
RALFB_RS25735	hypothetical protein
HXP34_19110	pilus assembly protein
EIH09_RS10025	collagen-like triple helix repeat-containing protein
RALW1_RS01165	hypothetical protein
EIH09_RS19085	TetR/AcrR family transcriptional regulator
RALFB_RS28170	hypothetical protein
RALW1_RS11345	hypothetical protein
RALCI_RS13455	membrane protein
RSPO_RS23195	HNH endonuclease
UW163_RS18990	hypothetical protein
RALFB_RS02820	lysozyme
RSMK_RS06700	hypothetical protein
RALFB_RS09865	triacylglycerol lipase
RALW1_RS08890	hypothetical protein
UW163_RS19520	SDR family oxidoreductase
EIH09_RS19560	J domain-containing protein
HXP37_22510	DUF2875 family protein
RALCI_RS24970	hypothetical protein
HXP37_14080	nitroreductase
pcaQ	pca operon transcription factor PcaQ
RALFB_RS19835	PilW family protein
EIH14_RS13015	pilus assembly protein
EIH13_RS15500	GNAT family N-acetyltransferase
RSMK_RS20945	GMC family oxidoreductase
yidC	membrane protein insertase YidC
AQR21_RS03980	threonine synthase
RALCI_RS12890	rRNA pseudouridine synthase
RSPO_RS11445	patatin-like phospholipase domain-containing protein
group_5373	type IV pilus modification protein PilV

Moko typical	
**Gene**	**Annotation**
EIH11_RS22270	chemotaxis protein CheV
AQR21_RS19650	hypothetical protein
EIH10_RS20835	hypothetical protein
HXP37_10485	porin
sctQ	type III secretion system cytoplasmic ring protein SctQ
RALFB_RS08435	hypothetical protein
RALFB_RS13215	PRTRC system protein C
RSMK_RS13350	PRTRC system protein E
pgaA	poly-beta-1,6 N-acetyl-D-glucosamine export porin PgaA
HXP34_21630	hypothetical protein
EIH12_RS20430	type II secretion system protein
RALCI_RS22410	DUF1993 family protein
EIH11_RS05550	type III effector protein
UW163_RS17625	flagellar hook-length control protein FliK
UW163_RS24395	calcium-binding protein
RALB5_RS08305	PRTRC system protein B
RALFB_RS13210	PRTRC system protein F
HXP35_21695	type IV pilus secretin PilQ
group_3167	type II secretion system major pseudopilin GspG
HXP36_18210	hypothetical protein
HXP37_19275	hypothetical protein
RSPO_RS23680	hypothetical protein
EIH13_RS17180	GNAT family N-acetyltransferase
HXP37_20845	alpha/beta hydrolase
RALW1_RS08245	SCO family protein
RALFB_RS19140	chromate resistance protein
HXP37_22425	phosphohydrolase
RALGR_RS12370	hypothetical protein
HXP34_08185	PRTRC system protein A

Moko typical	
**Gene**	**Annotation**
RALW1_RS11155	DUF3426 domain-containing protein
RSPO_RS25705	hypothetical protein
UW163_RS17370	nucleotidyltransferase family protein
AQR24_RS12360	LysM peptidoglycan-binding domain-containing protein
C2I33_RS12785	hypothetical protein
RALFB_RS10955	type I polyketide synthase
EIH14_RS00195	response regulator transcription factor
RSMK_RS13475	phage tail protein
RALFB_RS08370	polymerase
RSMK_RS13555	hypothetical protein
RALFB_RS20275	ABC transporter substrate-binding protein
RALGR_RS19410	hypothetical protein
C2I33_RS12625	class I SAM-dependent methyltransferase
HXP37_22365	hypothetical protein
RALW1_RS04535	hypothetical protein
RALCI_RS08645	type IV pilus secretin PilQ
HXP34_21640	type II secretion system protein
RALCI_RS16180	prepilin-type N-terminal cleavage/methylation domain-containing protein
RALB5_RS17985	type II secretion system F family protein
RALFB_RS16810	HAMP domain-containing histidine kinase
RALFB_RS13340	DUF3168 domain-containing protein
AQR24_RS23440	hypothetical protein
EIH10_RS20830	porin
RALCI_RS18870	hypothetical protein
AQR24_RS25480	glycoside hydrolase family protein
RALFB_RS08320	hypothetical protein
AQR24_RS05675	site-specific DNA-methyltransferase
HXP35_08190	hypothetical protein
AQR24_RS04365	hypothetical protein

Moko typical	
**Gene**	**Annotation**
RALCI_RS05245	DUF4400 domain-containing protein
RALFB_RS04440	hypothetical protein
traD	conjugative transfer system coupling protein TraD
AQR24_RS24920	host specificity protein J
AQR21_RS17805	HrgA protein
EIH10_RS08395	hypothetical protein
EIH10_RS18785	H-NS histone family protein
RALCI_RS07620	hypothetical protein
RSMK_RS06900	hypothetical protein
AQR24_RS19440	hypothetical protein
RALFB_RS04520	hypothetical protein
RALCI_RS14250	DUF853 domain-containing protein
RSPO_RS23650	hypothetical protein
C2I33_RS24245	phosphoribulokinase
AQR21_RS24025	Minor spike protein
EIH12_RS23335	IS256 family transposase
C2I33_RS04280	hypothetical protein
RALCI_RS07660	hypothetical protein
HXP36_07900	hypothetical protein
RALCI_RS18835	phage minor tail protein L
RALFB_RS17225	DUF2059 domain-containing protein
HXP36_18285	hypothetical protein
group_1962	2,3-diaminopropionate biosynthesis protein SbnA
RALCI_RS16205	hypothetical protein
RALFB_RS11125	PilT/PilU family type 4a pilus ATPase
group_2988	type VI secretion system tip protein VgrG
RSMK_RS14090	type II/IV secretion system protein
RALCI_RS07740	DUF1799 domain-containing protein
C2I33_RS03070	hypothetical protein

Moko typical	
**Gene**	**Annotation**
C2I33_RS19470	hypothetical protein
RALFB_RS26140	phage capsid protein
RALB5_RS25910	DNA replication protein
RALB5_RS25920	hypothetical protein
RALFB_RS14805	hypothetical protein
RALCI_RS22155	PHB depolymerase family esterase
EIH13_RS03615	MFS transporter
RALB5_RS25895	DNA-binding protein
RALW1_RS24490	scaffolding protein D
AQR21_RS24000	phage DNA packaging protein C
C2I33_RS23460	recombinase family protein
EIH14_RS07755	collagen-like triple helix repeat-containing protein
RALB5_RS18450	IS110 family transposase
RALGR_RS17830	M20/M25/M40 family metallo-hydrolase
group_1953	polyribonucleotide nucleotidyltransferase
C2I33_RS21380	hypothetical protein
C2I33_RS14755	hypothetical protein
RALGR_RS26185	hypothetical protein
C2I33_RS12660	DNA-binding protein
RALB5_RS19980	LysR family transcriptional regulator
RALCI_RS18890	hypothetical protein
RALGR_RS11310	PAS domain-containing protein
C2I33_RS00745	hypothetical protein
C2I33_RS03085	DUF2939 domain-containing protein
UW163_RS05455	hypothetical protein
EIH12_RS23760	pyridoxal phosphate-dependent aminotransferase
AQR24_RS18015	DNA-binding protein
RALFB_RS13290	hypothetical protein
RALFB_RS27940	hypothetical protein

Moko typical	
**Gene**	**Annotation**
RALFB_RS13805	type II toxin-antitoxin system HicA family toxin
RALFB_RS09140	hypothetical protein
RALB5_RS14445	hypothetical protein
C2I33_RS14740	hypothetical protein
RALFB_RS13235	hypothetical protein
RALCI_RS10545	pilus assembly protein
RSMK_RS05185	YaiI/YqxD family protein
C2I33_RS03080	class I SAM-dependent methyltransferase
RSMK_RS19435	hypothetical protein
RSMK_RS13355	hypothetical protein
AQR24_RS01435	hypothetical protein
C2I33_RS21135	HNH endonuclease
AQR24_RS01405	terminase large subunit
AQR24_RS01400	phage portal protein
C2I33_RS08700	Clp protease ClpP
RSMK_RS13450	phage major capsid protein
RALB5_RS00420	hypothetical protein
C2I33_RS08680	phage head closure protein
RALCI_RS07720	hypothetical protein
RALFB_RS13350	hypothetical protein
C2I33_RS08620	tail assembly protein
RALFB_RS13810	type II toxin-antitoxin system HicB family antitoxin
C2I33_RS08890	hypothetical protein
RSMK_RS25985	hypothetical protein
RALCI_RS19330	response regulator transcription factor
RSMK_RS07840	PaaI family thioesterase
RALCI_RS04320	CoA transferase
C2I33_RS20310	DUF2924 domain-containing protein
C2I33_RS08650	phage tail tape measure protein

Moko typical	
**Gene**	**Annotation**
RALCI_RS00910	hypothetical protein
C2I33_RS21895	hypothetical protein
HXP36_19450	aminotransferase class III-fold pyridoxal phosphate-dependent enzyme
RSMK_RS16000	hypothetical protein
UW163_RS20605	lipoprotein
RSMK_RS18125	ShlB/FhaC/HecB family hemolysin secretion/activation protein
RALFB_RS27460	avrbs3 family type III effector protein
C2I33_RS20550	acylphosphatase
RSMK_RS08145	hypothetical protein
RALFB_RS19700	hypothetical protein
RSMK_RS03450	zn-finger domain associated with topoisomerase typeIprotein
HXP36_01535	sel1 repeat family protein
RSMK_RS26435	hypothetical protein
HXP36_02975	hypothetical protein
RSMK_RS03010	HAMP domain-containing protein
RALCI_RS18590	HlyD family type I secretion periplasmic adaptor subunit
RALCI_RS25160	hypothetical protein
RALFB_RS06820	deoxynucleoside kinase
HXP36_06850	aminotransferase class I/II-fold pyridoxal phosphate-dependent enzyme
C2I33_RS06490	phosphotransferase
RALCI_RS09115	hypothetical protein
HXP36_06895	aspartate aminotransferase family protein
RALCI_RS09080	acyl-CoA/acyl-ACP dehydrogenase
RALFB_RS15795	hypothetical protein
RSMK_RS25705	XRE family transcriptional regulator
RALFB_RS13250	hypothetical protein
HXP36_07785	helix-turn-helix transcriptional regulator
RSMK_RS10495	GALA protein
RSMK_RS10480	FAD-dependent oxidoreductase

Moko typical	
**Gene**	**Annotation**
HXP36_12900	hypothetical protein
RALCI_RS02215	carbon-nitrogen hydrolase family protein
RALFB_RS07395	hypothetical protein
RSMK_RS23315	type III effector protein
C2I33_RS17455	avrd-related protein
RALFB_RS19245	acetyl-CoA C-acetyltransferase
C2I33_RS13140	LLM class flavin-dependent oxidoreductase
RALCI_RS20995	type III effector protein
RSMK_RS06435	hypothetical protein
RALFB_RS14125	hypothetical protein
HXP36_17675	hypothetical protein
RSMK_RS06025	hypothetical protein
group_3415	type VI secretion system tip protein VgrG
RSMK_RS06015	hypothetical protein
RALFB_RS22405	hypothetical protein
C2I33_RS20270	hypothetical protein
RSMK_RS05965	nuclear transport factor 2 family protein
HXP36_18080	OmpA family protein
RALCI_RS26170	hypothetical protein
RALFB_RS14535	DUF2894 domain-containing protein
C2I33_RS00325	hypothetical protein
RSMK_RS14975	lrr-gala family type III effector protein (gala 2)
C2I33_RS13515	hypothetical protein
C2I33_RS19410	hypothetical protein
HXP36_20895	TlpA family protein disulfide reductase
C2I33_RS24035	hypothetical protein
RALFB_RS04295	Hpt domain-containing protein
RALCI_RS10870	antibiotic biosynthesis monooxygenase
HXP36_21735	collagen-like triple helix repeat-containing protein

Moko typical	
**Gene**	**Annotation**
C2I33_RS18160	NUDIX hydrolase
RALFB_RS07560	hypothetical protein
C2I33_RS14520	hypothetical protein
RALFB_RS12755	hypothetical protein
C2I33_RS06790	hypothetical protein
RALFB_RS01445	hypothetical protein
HXP36_22985	4-oxalomesaconate tautomerase
C2I33_RS23375	hypothetical protein
HXP36_24010	hypothetical protein
HXP36_24185	hypothetical protein
RALCI_RS10740	hypothetical protein
RALCI_RS15295	mandelate racemase
HXP35_11005	PA0069 family radical SAM protein
RALCI_RS07910	anti-ECFsigma factor, ChrR
C2I33_RS18620	hypothetical protein
RSMK_RS04970	hypothetical protein
RALCI_RS20910	hypothetical protein
EIH12_RS13865	flavin-dependent oxidoreductase
RALFB_RS27900	hypothetical protein
RALFB_RS18185	hypothetical protein
UW163_RS26185	hypothetical protein
RSMK_RS14960	efflux RND transporter periplasmic adaptor subunit
UW163_RS25490	DUF746 domain-containing protein
HXP36_20025	hypothetical protein
RSMK_RS18130	filamentous hemagglutinin N-terminal domain-containing protein
RALFB_RS17450	hypothetical protein
RALCI_RS12290	SRPBCC domain-containing protein
HXP36_01270	nucleotide pyrophosphohydrolase
HXP36_01655	hypothetical protein

Moko typical	
**Gene**	**Annotation**
HXP36_02020	type III effector protein
RALFB_RS20250	recombinase family protein
RALFB_RS13475	VOC family protein
HXP36_02835	hypothetical protein
HXP36_03060	YcxB family protein
HXP36_03150	hypothetical protein
RSMK_RS03025	hypothetical protein
RSMK_RS02955	hypothetical protein
RALFB_RS12890	hypothetical protein
C2I33_RS16800	hypothetical protein
C2I33_RS23420	hypothetical protein
C2I33_RS23430	TolC family protein
RALCI_RS02350	hypothetical protein
RALFB_RS11015	hypothetical protein
HXP36_06020	hypothetical protein
RSMK_RS11125	TolC family protein
RALFB_RS06770	efflux RND transporter periplasmic adaptor subunit
RSMK_RS11135	efflux RND transporter permease subunit
RALFB_RS06795	tryptophan synthase subunit alpha
group_4984	tryptophan synthase subunit beta
RALFB_RS06805	amidohydrolase
HXP36_06835	aminotransferase class I/II-fold pyridoxal phosphate-dependent enzyme
RSMK_RS11175	hypothetical protein
C2I33_RS06495	glucose 1-dehydrogenase
RALCI_RS09110	Bcr/CflA family efflux MFS transporter
RALFB_RS06850	NAD-dependent epimerase/dehydratase family protein
RALFB_RS06855	hypothetical protein
RALFB_RS06860	hypothetical protein
RSMK_RS11225	polyketide cyclase/dehydrase

Moko typical	
**Gene**	**Annotation**
HXP36_06915	NAD(P)H-dependent oxidoreductase
RSMK_RS11260	alpha/beta hydrolase
RSMK_RS11265	LysR family transcriptional regulator
C2I33_RS05285	hypothetical protein
RSMK_RS12615	hypothetical protein
C2I33_RS13385	hypothetical protein
HXP36_09435	aminotransferase class III-fold pyridoxal phosphate-dependent enzyme
HXP36_09440	cupin-like domain-containing protein
RALFB_RS19605	hypothetical protein
RALFB_RS16660	hypothetical protein
RALCI_RS07235	hypothetical protein
group_5212	bifunctional nicotinamidase/pyrazinamidase
RALCI_RS22635	acyl-CoA-binding protein
RALCI_RS26350	hypothetical protein
RALFB_RS20335	metal-binding protein
RSMK_RS19675	hypothetical protein
RALFB_RS02320	cytochrome c
RALFB_RS02330	membrane protein
RALFB_RS19805	PilW family protein
RALFB_RS19800	pilus assembly protein
RALFB_RS24655	chain-length determining protein
HXP36_15300	hypothetical protein
RSMK_RS23145	HAD-IA family hydrolase
RSMK_RS22890	hypothetical protein
RALCI_RS03300	type II toxin-antitoxin system HigB family toxin
C2I33_RS11895	hypothetical protein
RSMK_RS22455	methyltransferase domain-containing protein
RALFB_RS01160	hypothetical protein
RSMK_RS21745	type III effector protein

Moko typical	
**Gene**	**Annotation**
RALFB_RS18095	phospholipase C, phosphocholine-specific
RALCI_RS23565	hypothetical protein
HXP36_17290	GNAT family N-acetyltransferase
RALFB_RS14985	4-oxalocrotonate tautomerase family protein
RALFB_RS25100	hypothetical protein
RSMK_RS06135	undecaprenyl-diphosphate phosphatase
RALCI_RS15470	DUF4124 domain-containing protein
C2I33_RS10115	ankyrin repeat domain-containing protein
RSMK_RS06010	DUF2169 domain-containing protein
RALFB_RS22325	hypothetical protein
RALFB_RS22320	DUF4150 domain-containing protein
C2I33_RS10150	hypothetical protein
RALFB_RS22425	EAL domain-containing protein
RALFB_RS16130	GNAT family N-acetyltransferase
HXP36_18305	hypothetical protein
C2I33_RS14860	amino acid permease
HXP36_19145	TetR/AcrR family transcriptional regulator
RSMK_RS14590	TolC family protein
RALFB_RS04950	oxidoreductase
RALCI_RS13165	cyclic peptide export ABC transporter
C2I33_RS07525	MBL fold metallo-hydrolase
RALCI_RS13155	MbtH family protein
RALCI_RS04130	hypothetical protein
C2I33_RS24715	efflux RND transporter permease subunit
RALCI_RS02990	hypothetical protein
RALFB_RS27380	hypothetical protein
RALFB_RS21135	hypothetical protein
RALFB_RS17660	YukJ family protein
RSMK_RS16125	CPBP family intramembrane metalloprotease

Moko typical	
**Gene**	**Annotation**
C2I33_RS06970	AAA family ATPase
HXP36_20915	copper uptake system-associated protein
RALCI_RS26235	hypothetical protein
HXP36_21185	thiazolinyl imide reductase
RALFB_RS09295	DUF596 domain-containing protein
C2I33_RS08865	hypothetical protein
group_5618	Flp pilus assembly protein CpaB
RALCI_RS20670	DUF2145 domain-containing protein
RALCI_RS09515	hypothetical protein
RALCI_RS09535	helix-turn-helix domain-containing protein
HXP36_22360	hypothetical protein
C2I33_RS06125	hypothetical protein
RSMK_RS18065	gallate dioxygenase
C2I33_RS19070	aromatic acid/H+ symport family MFS transporter
RALFB_RS24145	NAD(P)-dependent oxidoreductase
C2I33_RS15455	TetR/AcrR family transcriptional regulator
HXP36_24130	hypothetical protein
RALCI_RS10750	hypothetical protein
RALFB_RS07245	MFS transporter
HXP36_24430	hypothetical protein
RSMK_RS04695	heme ABC transporter ATP-binding protein
RSMK_RS04850	hypothetical protein
C2I33_RS22145	hypothetical protein
RALW1_RS24955	hypothetical protein
RSMK_RS11145	hypothetical protein
RSMK_RS11825	hypothetical protein
RSMK_RS15555	ATP-binding cassette domain-containing protein
RALB5_RS00390	hypothetical protein
RALFB_RS26050	hypothetical protein

Moko typical	
**Gene**	**Annotation**
RSMK_RS18120	hypothetical protein
EIH14_RS08905	peptidyl-prolyl cis-trans isomerase
AQR21_RS23920	AMP-binding protein
RALFB_RS23035	hypothetical protein
RALFB_RS17815	hypothetical protein
RSMK_RS25450	AAA family ATPase
trbL	P-type conjugative transfer protein TrbL
RALCI_RS11965	DUF1911 domain-containing protein
RALFB_RS13380	hypothetical protein
RSMK_RS18085	porin
HXP36_04855	hemagglutinin
RSMK_RS11315	hypothetical protein
RALCI_RS02905	DUF4123 domain-containing protein
RALFB_RS17665	Dyp-type peroxidase
RALCI_RS26085	hypothetical protein
RALFB_RS14000	hypothetical protein
RSMK_RS11115	hypothetical protein
C2I33_RS08565	hypothetical protein
RALCI_RS25470	hypothetical protein
RSMK_RS26690	hypothetical protein
RALFB_RS18765	hypothetical protein
RALCI_RS24720	hypothetical protein
RSMK_RS26870	hypothetical protein
RALFB_RS11595	AAA family ATPase
RALFB_RS27895	hypothetical protein
C2I33_RS21410	DUF3800 domain-containing protein
RALCI_RS10085	putative metallophosphoesterase protein
RALCI_RS10735	hypothetical protein
RSMK_RS03310	tryptophan 2-monooxygenase oxidoreductase

Moko typical	
**Gene**	**Annotation**
C2I33_RS12815	SgcJ/EcaC family oxidoreductase
RSMK_RS02060	DUF1311 domain-containing protein
C2I33_RS08635	tail assembly protein
RALCI_RS26180	hypothetical protein
RSMK_RS12735	hypothetical protein
RALCI_RS11515	hypothetical protein
RALCI_RS26580	hypothetical protein
RALCI_RS16570	methyltransferase domain-containing protein
HXP36_17875	hypothetical protein
RALFB_RS16395	type III effector protein
HXP36_21110	hypothetical protein
RALCI_RS09440	DUF2239 family protein
RALFB_RS05810	LysR family transcriptional regulator
HXP36_22990	amidohydrolase
ligK	4-carboxy-4-hydroxy-2-oxoadipate aldolase/oxaloacetate decarboxylase
HXP36_24500	ABC transporter substrate-binding protein
RSMK_RS04690	iron ABC transporter permease
HXP36_24695	amino acid ABC transporter permease
AQR24_RS16540	PAS domain-containing methyl-accepting chemotaxis protein
RSMK_RS00100	DUF3306 domain-containing protein
C2I33_RS23315	hypothetical protein
ltrA	group II intron reverse transcriptase/maturase
RALCI_RS08825	hypothetical protein
RALFB_RS16960	metal-binding protein
RALCI_RS25480	hypothetical protein
C2I33_RS06920	hypothetical protein
RALFB_RS17820	DUF2778 domain-containing protein
RALCI_RS00565	hypothetical protein
C2I33_RS12870	hypothetical protein

Moko typical	
**Gene**	**Annotation**
C2I33_RS13950	hypothetical protein
RALCI_RS16090	hypothetical protein
RSMK_RS04065	hypothetical protein
RALFB_RS27075	hypothetical protein
RSMK_RS25220	ImmA/IrrE family metallo-endopeptidase
RALCI_RS25930	DUF4411 family protein
RALCI_RS07935	nucleotidyl transferase AbiEii/AbiGii toxin family protein
RALFB_RS27665	hypothetical protein
RALCI_RS14350	hypothetical protein
RSMK_RS26460	hypothetical protein
RALCI_RS00250	nuclear transport factor 2 family protein
RSMK_RS05140	YafY family transcriptional regulator
RALFB_RS14610	flavoprotein
C2I33_RS02780	hypothetical protein
C2I33_RS14855	FAD-dependent oxidoreductase
RSMK_RS05580	hypothetical protein
RALFB_RS28145	hypothetical protein
RALCI_RS01820	hypothetical protein
RALCI_RS01780	ShET2/EspL2 family type III secretion system effector toxin
RALCI_RS16825	hypothetical protein
RSMK_RS06040	hypothetical protein
RSMK_RS25310	metallophosphoesterase
RSMK_RS26510	hypothetical protein
RALFB_RS22380	type I toxin-antitoxin system SymE family toxin
RALCI_RS21935	hypothetical protein
RALCI_RS16600	hypothetical protein
RALFB_RS14960	hypothetical protein
RALFB_RS02560	type II toxin-antitoxin system RelE/ParE family toxin
C2I33_RS01785	hypothetical protein

Moko typical	
**Gene**	**Annotation**
RALCI_RS05755	hypothetical protein
RALCI_RS26215	hypothetical protein
RALFB_RS27105	hypothetical protein
RALCI_RS13130	hypothetical protein
C2I33_RS04210	DUF3800 domain-containing protein
C2I33_RS04215	hypothetical protein
C2I33_RS04225	DNA-binding protein
RALCI_RS16930	hypothetical protein
RALFB_RS04565	hypothetical protein
RALCI_RS05345	hypothetical protein
C2I33_RS04260	hypothetical protein
RSMK_RS06905	hypothetical protein
C2I33_RS04315	tyrosine-type recombinase/integrase
RALFB_RS04480	ParB/RepB/Spo0J family partition protein
RSMK_RS06965	RepB family plasmid replication initiator protein
RALFB_RS04470	ATP-dependent helicase
RSMK_RS06975	hypothetical protein
RALFB_RS04460	flagellar transcriptional regulator FlhD
RSMK_RS06985	hypothetical protein
C2I33_RS04360	hypothetical protein
C2I33_RS04375	type VI secretion protein
RALFB_RS04425	conjugal transfer protein TrbL
RSMK_RS07020	hypothetical protein
RALFB_RS04415	conjugal transfer protein TrbE
RALFB_RS04410	conjugal transfer protein
RALCI_RS05200	hypothetical protein
RALCI_RS05195	CpaF family protein
RALCI_RS05190	type IV secretion system protein
C2I33_RS04415	TrbG/VirB9 family P-type conjugative transfer protein

Moko typical	
**Gene**	**Annotation**
C2I33_RS04420	TrbI/VirB10 family protein
C2I33_RS04425	hypothetical protein
RALCI_RS05170	TrbG/VirB9 family P-type conjugative transfer protein
RSMK_RS07070	lytic transglycosylase domain-containing protein
RALCI_RS05160	pilus assembly protein
RSMK_RS07080	hypothetical protein
pilP	type IV pilus biogenesis protein PilP
pilO2	type 4b pilus protein PilO2
RSMK_RS07095	Flp pilus assembly complex ATPase component
RSMK_RS07100	type II secretion system protein
C2I33_RS04470	hypothetical protein
RALFB_RS26345	hypothetical protein
group_6238	shufflon system plasmid conjugative transfer pilus tip adhesin PilV
RALFB_RS04325	CAP domain-containing protein
RSMK_RS07120	hypothetical protein
RALFB_RS26335	hypothetical protein
RALCI_RS05110	hypothetical protein
RSMK_RS07130	hypothetical protein
RALCI_RS25190	preprotein translocase subunit SecF
C2I33_RS19320	hypothetical protein
RALFB_RS27285	hypothetical protein
RALCI_RS22700	hypothetical protein
C2I33_RS21680	filamentous hemagglutinin N-terminal domain-containing protein
RALFB_RS23520	hypothetical protein
RALCI_RS25690	replication initiation protein
RALCI_RS21120	hypothetical protein
RSMK_RS26580	hypothetical protein
RALFB_RS04620	PAAR domain-containing protein
C2I33_RS04185	DUF4123 domain-containing protein

Moko typical	
**Gene**	**Annotation**
C2I33_RS04190	hypothetical protein
RSMK_RS07470	hypothetical protein
RALFB_RS26355	H-NS histone family protein
RALFB_RS22410	thioredoxin family protein
RSMK_RS26220	hypothetical protein
RSMK_RS26600	hypothetical protein
C2I33_RS00010	AAA family ATPase
RSMK_RS08135	ankyrin repeat domain-containing protein
RSMK_RS08555	hypothetical protein
RALCI_RS13405	hypothetical protein
RSMK_RS09495	type III effector protein
RALCI_RS19355	GALA protein
RALFB_RS13940	site-specific integrase
RALCI_RS00325	hypothetical protein
C2I33_RS08090	hypothetical protein
RALFB_RS13955	AlpA family phage regulatory protein
C2I33_RS08100	hypothetical protein
RSMK_RS09910	hypothetical protein
RALCI_RS00300	hypothetical protein
C2I33_RS08125	hypothetical protein
C2I33_RS08130	DUF945 domain-containing protein
RALFB_RS13990	ParB N-terminal domain-containing protein
RALCI_RS00265	DUF736 domain-containing protein
C2I33_RS08160	helix-turn-helix transcriptional regulator
C2I33_RS08170	DUF2285 domain-containing protein
RALCI_RS17250	helix-turn-helix domain-containing protein
C2I33_RS08180	replication initiator protein A
RALCI_RS17240	AAA family ATPase
RALFB_RS17770	hypothetical protein

Moko typical	
**Gene**	**Annotation**
C2I33_RS08200	DUF2840 domain-containing protein
RSMK_RS09990	peptidase
RSMK_RS09995	relaxase/mobilization nuclease and DUF3363 domain-containing protein
RALFB_RS17750	TetR/AcrR family transcriptional regulator
RALCI_RS17210	hypothetical protein
RALCI_RS25620	hypothetical protein
traG	conjugal transfer protein TraG
RALFB_RS21220	ribbon-helix-helix protein, CopG family
RALCI_RS18565	conjugal transfer protein TrbC
RSMK_RS10045	conjugal transfer protein TrbD
trbJ	P-type conjugative transfer protein TrbJ
C2I33_RS21315	conjugal transfer protein TrbF
trbG	P-type conjugative transfer protein TrbG
RALFB_RS21265	TrbI/VirB10 family protein
RALCI_RS25615	DUF2274 domain-containing protein
RSMK_RS10085	efflux transporter outer membrane subunit
RALCI_RS22835	efflux RND transporter periplasmic adaptor subunit
RSMK_RS10100	response regulator transcription factor
RALCI_RS25280	DUF934 domain-containing protein
RALFB_RS28180	hypothetical protein
RSMK_RS10460	putative tyrosinase domain protein
RSMK_RS10465	hypothetical protein
RALCI_RS09580	DUF2182 domain-containing protein
RALCI_RS25165	hypothetical protein
RALFB_RS06890	DUF262 domain-containing protein
C2I33_RS21805	hypothetical protein
C2I33_RS11670	hypothetical protein
RALCI_RS05895	lytic transglycosylase domain-containing protein
RALCI_RS03855	hypothetical protein

Moko typical	
**Gene**	**Annotation**
RSMK_RS12845	DUF1488 domain-containing protein
RALCI_RS01035	hypothetical protein
RALGR_RS05475	ATP-binding cassette domain-containing protein
RALCI_RS13400	chromosome partitioning protein ParA
RALCI_RS13395	hypothetical protein
C2I33_RS11090	AAA family ATPase
RALFB_RS26575	hypothetical protein
RALFB_RS08960	tyrosine-type recombinase/integrase
RSMK_RS25695	DUF4224 domain-containing protein
C2I33_RS21075	hypothetical protein
C2I33_RS21105	hypothetical protein
RALFB_RS13280	hypothetical protein
RALFB_RS13835	tail fiber protein
RALFB_RS13795	hypothetical protein
C2I33_RS08580	hypothetical protein
C2I33_RS08570	hypothetical protein
RALCI_RS18895	hypothetical protein
RALCI_RS25465	DUF4304 domain-containing protein
RSMK_RS26685	hypothetical protein
RALFB_RS18810	hypothetical protein
RALCI_RS11650	hypothetical protein
RSMK_RS14285	hypothetical protein
RALFB_RS08165	hypothetical protein
RALCI_RS25510	hypothetical protein
RALFB_RS22785	hypothetical protein
RSMK_RS14540	hypothetical protein
RALCI_RS23330	LysR family transcriptional regulator
RALCI_RS23325	DMT family transporter
RALCI_RS26690	hypothetical protein

Moko typical	
**Gene**	**Annotation**
RALCI_RS19240	hypothetical protein
RALCI_RS25345	hypothetical protein
RALCI_RS11970	hypothetical protein
RSMK_RS25890	hypothetical protein
RALFB_RS27260	hypothetical protein
RALCI_RS26645	hypothetical protein
C2I33_RS21770	hypothetical protein
RALFB_RS09040	hypothetical protein
RSMK_RS26760	hypothetical protein
RALFB_RS27180	hypothetical protein
RSMK_RS16255	membrane protein
RALCI_RS01480	DUF4865 family protein
RALFB_RS08300	IS3 family transposase
C2I33_RS08875	DUF596 domain-containing protein
RSMK_RS17185	DUF3175 domain-containing protein
RALCI_RS09425	hypothetical protein
C2I33_RS06000	barnase inhibitor
RSMK_RS26810	hypothetical protein
RSMK_RS26045	hypothetical protein
RSMK_RS26835	hypothetical protein
RALCI_RS13535	F-box protein
RALCI_RS13570	type III effector protein
C2I33_RS22930	hypothetical protein
C2I33_RS22925	DUF2247 family protein
RALCI_RS25400	hypothetical protein
RALCI_RS26375	hypothetical protein
RSMK_RS18210	type III effector protein
RSMK_RS18235	hypothetical protein
RALFB_RS27545	hypothetical protein

Moko typical	
**Gene**	**Annotation**
RALCI_RS26685	hypothetical protein
RSMK_RS18390	hypothetical protein
RALCI_RS25940	DUF4279 domain-containing protein
RALCI_RS25910	DUF416 family protein
C2I33_RS23685	hypothetical protein
RSMK_RS18755	hypothetical protein
RSMK_RS26885	hypothetical protein
C2I33_RS19955	single-stranded DNA-binding protein
C2I33_RS17175	hypothetical protein
RALFB_RS11585	hypothetical protein
RALCI_RS25525	hypothetical protein
C2I33_RS17190	hypothetical protein
RALCI_RS16630	HigA family addiction module antidote protein
C2I33_RS17200	Killer protein
RALFB_RS11565	integrase family protein
RALFB_RS05110	helix-turn-helix transcriptional regulator
C2I33_RS24750	hypothetical protein
RSMK_RS19400	VCBS repeat-containing protein
RSMK_RS19420	DNA-deoxyinosine glycosylase
RALCI_RS25680	hypothetical protein
RALCI_RS21100	hypothetical protein
RSMK_RS19890	hypothetical protein
C2I33_RS00320	hypothetical protein
RALFB_RS14870	DUF1484 domain-containing protein
RALFB_RS02770	hypothetical protein
RSMK_RS19990	DNA cytosine methyltransferase
C2I33_RS00175	hypothetical protein
RALCI_RS05080	hypothetical protein
RSMK_RS20100	hypothetical protein

Moko typical	
**Gene**	**Annotation**
RALCI_RS12350	hypothetical protein
C2I33_RS00040	hypothetical protein
RSMK_RS20160	hypothetical protein
RALCI_RS12385	hypothetical protein
RALFB_RS14860	DUF3732 domain-containing protein
RSMK_RS20245	RHS repeat protein
C2I33_RS21450	hypothetical protein
RALCI_RS23930	DUF1484 domain-containing protein
RALFB_RS25170	DUF3489 domain-containing protein
RALFB_RS23480	hypothetical protein
RALFB_RS23470	AAA family ATPase
RALCI_RS22395	helix-turn-helix domain-containing protein
RALCI_RS25420	hypothetical protein
RSMK_RS20380	hypothetical protein
RSMK_RS20605	hypothetical protein
RALCI_RS19685	DUF433 domain-containing protein
RSMK_RS21275	hypothetical protein
C2I33_RS20895	hypothetical protein
RALCI_RS10165	hypothetical protein
RALFB_RS00540	hypothetical protein
C2I33_RS17550	tartrate dehydrogenase
C2I33_RS23795	pilin
C2I33_RS14420	DDE-type integrase/transposase/recombinase
RSMK_RS24065	GMC family oxidoreductase
RALCI_RS25245	hypothetical protein
RALFB_RS10635	calcium-binding protein
RSMK_RS24470	hypothetical protein
C2I33_RS16520	BON domain-containing protein
RSMK_RS24885	hypothetical protein

Moko typical	
**Gene**	**Annotation**
RALFB_RS18110	hypothetical protein
RALFB_RS24325	AMP-binding protein
HXP37_06935	AAA family ATPase
RSMK_RS01400	hypothetical protein
C2I33_RS02490	multidrug efflux RND transporter permease subunit
RALFB_RS25535	peptidyl-prolyl cis-trans isomerase
group_1999	4-alpha-glucanotransferase
RALFB_RS25470	FimV family protein
RALB5_RS23790	DUF2300 domain-containing protein
group_264	elongation factor G
C2I33_RS20245	hypothetical protein
C2I33_RS24520	TonB-dependent receptor
RALFB_RS20225	hypothetical protein
RSMK_RS04080	hypothetical protein
RALCI_RS24960	hypothetical protein
RSMK_RS26535	hypothetical protein
mreB	rod shape-determining protein MreB
RALCI_RS26435	hypothetical protein
RALFB_RS27355	DUF2380 domain-containing protein
RALFB_RS26095	hypothetical protein
C2I33_RS00120	hypothetical protein
HXP36_01355	hypothetical protein
RALCI_RS21190	amino acid permease
RSMK_RS24075	AsmA family protein
C2I33_RS01700	formylglycine-generating enzyme family protein
RALFB_RS28280	hypothetical protein
RALFB_RS13470	hypothetical protein
RALFB_RS27070	hypothetical protein
RSMK_RS04055	AlpA family phage regulatory protein

Moko typical	
**Gene**	**Annotation**
RALFB_RS27760	hypothetical protein
RSMK_RS05075	hypothetical protein
RSMK_RS05455	hypothetical protein
RSMK_RS26500	hypothetical protein
RSMK_RS05865	hypothetical protein
C2I33_RS20450	hypothetical protein
RSMK_RS06885	hypothetical protein
RALFB_RS04485	ParA family protein
RALCI_RS26620	hypothetical protein
C2I33_RS21675	ShlB/FhaC/HecB family hemolysin secretion/activation protein
RALFB_RS18345	ankyrin repeat domain-containing protein
RALFB_RS27935	hypothetical protein
RALFB_RS27790	hypothetical protein
RALFB_RS09070	hypothetical protein
RALCI_RS26395	hypothetical protein
RSMK_RS26805	hypothetical protein
RALFB_RS17245	type III effector protein
RALCI_RS25905	DUF4279 domain-containing protein
RALCI_RS08965	hypothetical protein
RALCI_RS26480	hypothetical protein
RSMK_RS26935	hypothetical protein
RALFB_RS27980	hypothetical protein
RALFB_RS13995	hypothetical protein
RALCI_RS01940	hypothetical protein
RALCI_RS26305	excinuclease, ATPase subunit precursor
RALFB_RS28175	hypothetical protein
RALCI_RS26370	hypothetical protein
RALFB_RS04600	hypothetical protein
RALCI_RS19160	pyrimidine reductase

Moko typical	
**Gene**	**Annotation**
RALCI_RS26625	hypothetical protein
RALFB_RS18860	HAMP domain-containing histidine kinase
RSMK_RS00010	MFS transporter
RSMK_RS01415	type I secretion system permease/ATPase
RALFB_RS25940	AMP-binding protein
EIH13_RS17035	elongation factor Tu
RALCI_RS24750	amino acid adenylation domain-containing protein
RALCI_RS04680	AAA domain-containing protein
group_1425	Fe-S protein assembly chaperone HscA
C2I33_RS11975	transposase
RALGR_RS22015	MFS transporter
RALW1_RS07430	ferritin-like domain-containing protein
RALCI_RS24775	transposase
C2I33_RS20255	hypothetical protein
RALCI_RS15425	hypothetical protein
C2I33_RS06925	Tn3 family transposase
RALFB_RS22190	DUF4158 domain-containing protein
HXP36_01275	hypothetical protein
RALFB_RS16005	hemagglutinin
C2I33_RS25475	transposase
RALFB_RS25620	IS3 family transposase
RSMK_RS03520	type III effector protein (skwp 1)
RSMK_RS03845	RHS repeat protein
RSMK_RS20230	hypothetical protein
C2I33_RS19745	hypothetical protein
RALCI_RS26155	hypothetical protein
RALCI_RS25570	LysR family transcriptional regulator
RALCI_RS05075	hypothetical protein
group_475	tRNA-guanine transglycosylase

Moko typical	
**Gene**	**Annotation**
HXP36_23170	hypothetical protein
RSMK_RS18385	type IV pilus secretin PilQ
RALCI_RS26765	hypothetical protein
RSMK_RS20225	AlpA family phage regulatory protein
C2I33_RS02960	hypothetical protein
C2I33_RS08165	DUF2958 domain-containing protein
C2I33_RS15380	filamentous hemagglutinin N-terminal domain-containing protein
C2I33_RS11110	hypothetical protein
RALFB_RS18385	DUF3005 domain-containing protein
RSMK_RS26825	hypothetical protein
C2I33_RS00290	hypothetical protein
RSMK_RS20240	RHS repeat protein
RALFB_RS00115	hypothetical protein
RALCI_RS04125	alpha/beta fold hydrolase
RALCI_RS26220	hypothetical protein
RALFB_RS27660	hypothetical protein
group_6952	dethiobiotin synthase
RALFB_RS27800	hypothetical protein
RALCI_RS21375	efflux RND transporter periplasmic adaptor subunit
RALFB_RS28295	hypothetical protein
RSMK_RS00465	response regulator transcription factor
RALFB_RS25495	AMP-binding protein
HXP36_19565	efflux transporter outer membrane subunit
EIH14_RS23045	IS3 family transposase
C2I33_RS25480	IS3 family transposase
