# Supplementary material for: Comparative genomics and phylogenomics of the Ralstonia solanacearum Moko ecotype and its symptomatological variants
Source: Genet Mol Biol. 2022 Dec 2;45(4):e20220038. doi: 10.1590/1678-4685-GMB-2022-0038 (PMC9731368; doi:10.1590/1678-4685-GMB-2022-0038)
Supplement: Table S3 - [file 1415-4757-GMB-45-4-e20220038-s3.pdf]

Genetics and Molecular Biology
https://doi.org/10.1590/1678-4685-GMB-2022-0038

Supplementary material to “Comparative genomics and phylogenomics of the *Ralstonia solanacearum* Moko ecotype and its symptomatological variants”

Table S3 - Exclusive and shared gene clusters obtained by pan-genome analysis of the *Ralstonia solanacearum* Moko ecotype and its symptomatological variants.

**Sergipe facies**			
**Gene**	**Annotation**	**Function**	**Description**
C2L97_RS17080	4'-phosphopantetheinyl transferase superfamily protein	Molecular function	Enzyme that transfers a chemical group
C2L97_RS22830	amino acid (threonine) efflux protein	Biological process	Conducts the efflux of threonine
C2I38_RS09790	ATP-binding cassette domain-containing protein	Molecular function, Biological process and Ligand	
C2L97_RS07505	ATP-binding protein	Ligand	Protein which binds adenosine 5'-triphosphate (ATP), a ribonucleotide adenosine (a purine base adenine linked to the sugar D-ribofuranose) that carries three phosphate groups esterified to the sugar moiety. It is the cell's source for energy and phosphate.
C2L97_RS05450	ATP-binding protein	Ligand	Protein which binds adenosine 5'-triphosphate (ATP), a ribonucleotide adenosine (a purine base adenine linked to the sugar D-ribofuranose) that carries three phosphate groups esterified to the sugar moiety. It is the cell's source for energy and phosphate.
C2I38_RS03505	collagen-like triple helix repeat-containing protein	Molecular function and Biological process	May act as a negative regulator of collagen matrix deposition.
C2I38_RS20670	collagen-like triple helix repeat-containing protein	Molecular function and Biological process	
C2I38_RS08940	FAD-dependent oxidoreductase	Molecular function	oxidoreductase activity, acting on CH-OH group of donors
C2L97_RS09670	GALA protein	Type three secretion system	
C2I38_RS16580	GALA protein	Type three secretion system	
C2L97_RS16515	GNAT family N-acetyltransferase	Molecular Function	Enzyme that transfers a chemical group
C2L97_RS22695	HAD family hydrolase	Molecular Function	Catalysis of the hydrolysis of various bonds, e.g. C-O, C-N, C-C, phosphoric anhydride bonds
C2L97_RS22615	Hpt domain-containing protein	Molecular function and Biological process	

Sergipe facies			
**Gene**	**Annotation**	**Function**	**Description**
C2L97_RS01245	hypothetical protein	Unknown	
C2I38_RS02125	hypothetical protein	Unknown	
C2I38_RS17810	hypothetical protein	Unknown	
C2L97_RS23970	hypothetical protein	Unknown	
C2L97_RS00060	hypothetical protein	Unknown	
C2I38_RS24315	hypothetical protein	Unknown	
C2L97_RS01160	hypothetical protein	Unknown	
C2I38_RS01185	hypothetical protein	Unknown	
C2L97_RS01240	hypothetical protein	Unknown	
C2I38_RS01355	hypothetical protein	Unknown	
C2I38_RS01765	hypothetical protein	Unknown	
C2L97_RS02990	hypothetical protein	Unknown	
C2L97_RS04560	hypothetical protein	Unknown	
C2I38_RS24395	hypothetical protein	Unknown	
C2L97_RS11705	hypothetical protein	Unknown	
C2L97_RS16460	hypothetical protein	Unknown	
C2L97_RS16995	hypothetical protein	Unknown	
C2I38_RS24630	hypothetical protein	Unknown	
C2L97_RS17150	hypothetical protein	Unknown	
C2I38_RS24665	hypothetical protein	Unknown	
C2I38_RS18450	hypothetical protein	Unknown	
C2I38_RS20920	hypothetical protein	Unknown	
C2I38_RS24755	hypothetical protein	Unknown	
C2I38_RS21875	hypothetical protein	Unknown	

Sergipe facies			
**Gene**	**Annotation**	**Function**	**Description**
C2L97_RS21925	hypothetical protein	Unknown	
C2L97_RS22815	hypothetical protein	Unknown	
C2I38_RS23140	hypothetical protein	Unknown	
C2I38_RS24815	hypothetical protein	Unknown	
C2L97_RS24100	hypothetical protein	Unknown	
C2I38_RS18335	integrase		
C2L97_RS05445	IS21 family transposase	Deletions, inversions and gene amplification (multifold)	
C2L97_RS07510	IS21 family transposase	Deletions, inversions and gene amplification (multifold)	
C2L97_RS07875	IS21 family transposase	Deletions, inversions and gene amplification (multifold)	
C2I38_RS20825	IS21 family transposase	Deletions, inversions and gene amplification (multifold)	
C2L97_RS07490	IS3 family transposase	Deletions, inversions and gene amplification (multifold)	
C2L97_RS18380	IS5 family transposase	Deletions, inversions and gene amplification (multifold)	
C2I38_RS22905	Lrp/AsnC ligand binding domain-containing protein	Molecular function and Biological process	
C2L97_RS18055	metallophosphoesterase	Molecular function, Biological process and Ligand	Metallophosphoesterase required for transport of GPI-anchor proteins from the endoplasmic reticulum to the Golgi. Acts in lipid remodeling steps of GPI-anchor maturation by mediating the removal of a side-chain ethanolamine-phosphate (EtNP) from the second Man (Man2) of the GPI intermediate, an essential step for efficient transport of GPI-anchor proteins.
C2L97_RS20400	NUDIX hydrolase	Molecular function and Biological process	Mediates the hydrolysis of some nucleoside diphosphate derivatives. Can use FAD and ADP-ribose as substrates
C2I38_RS24050	type I addiction module toxin, SymE family	Type one secretion system	
group_7717	type I-E CRISPR-associated protein Cse2/CasB	CRISPR	
C2I38_RS09750	type III effector protein	Type three secretion system	
C2I38_RS20055	type III effector protein	Type three secretion system	

Gene	Annotation	Function	Description
**Sergipe facies**			
**Gene**	**Annotation**	**Function**	**Description**
C2L97_RS20110	type III effector protein	Type three secretion system	
C2I38_RS22115	type III effector protein	Type three secretion system	
C2I38_RS22580	type III effector protein	Type three secretion system	
C2I38_RS21910	type III effector protein	Type three secretion system	
**Bugtok disease**			
Gene	Annotation	Function	
RSMK_RS03090	DUF4158 domain-containing protein	Unknown	
RALCI_RS23880	IS3 family transposase	Deletions, inversions and gene amplification (multifold)	
RSMK_RS26545	hypothetical protein	Unknown	
RSMK_RS25490	IS5 family transposase	Deletions, inversions and gene amplification (multifold)	
**Moko typical and Sergipe facies**			
Gene	Annotation	Function	Description
mreC	rod shape-determining protein MreC	Molecular function and Biological process	Involved in formation and maintenance of cell shape. Responsible for formation of rod shape. May also contribute to regulation of formation of penicillin-binding proteins
**Moko typical and Bugtok disease**			
Gene	Annotation	Function	Description
AQR21_RS11265	ABC transporter substrate-binding protein	Biological process	Protein involved in the transport of amino acids
HXP35_00525	cellobiose phosphorylase	Molecular function and Biological process	
AQR24_RS19785	diguanylate cyclase	Molecular function and Biological process	Catalyzes the synthesis of cyclic-di-GMP via the condensation of 2 GTP molecules
AQR24_RS11595	EAL domain-containing protein	100% identity with GGDEF	
RSPO_RS19700	GGDEF domain-containing protein	Cellular component	
RALFB_RS02865	hypothetical protein	Unknown	
HXP37_09800	hypothetical protein	Unknown	

Sergipe facies			
**Gene**	**Annotation**	**Function**	**Description**
EIH11_RS06270	LysR family transcriptional regulator	Molecular function	Any molecular function by which a gene product interacts selectivelys and non-covalently with DNA (deoxyribonucleic acid)
RSPO_RS08930	MoxR family ATPase	Molecular function	
EIH12_RS04460	nicotinate phosphoribosyltransferase	Molecular function and Biological process	Catalyzes the first step in the biosynthesis of NAD from nicotinic acid, the ATP-dependent synthesis of betanicotinate D-ribonucleotide from nicotinate and 5-phospho-D-ribose 1-phosphate. Helps prevent cellular oxidative stress via its role in NAD biosynthesis.
HXP35_14755	TonB-dependent receptor	Molecular function	Signaling receptor activity
C2I33_RS23970	TonB-dependent receptor	Molecular function	Signaling receptor activity
RALW1_RS18940	type II toxin-antitoxin system HipA family toxin	Molecular function and Biological process	
RALFB_RS07690	tyrosine-type recombinase/integrase	Molecular function and Biological process	
