# Supplementary material for: Comparative genomics and phylogenomics of the Ralstonia solanacearum Moko ecotype and its symptomatological variants
Source: Genet Mol Biol. 2022 Dec 2;45(4):e20220038. doi: 10.1590/1678-4685-GMB-2022-0038 (PMC9731368; doi:10.1590/1678-4685-GMB-2022-0038)
Supplement: Figure S1 - [file 1415-4757-GMB-45-4-e20220038-s4.pdf]

Genetics and Molecular Biology
https://doi.org/10.1590/1678-4685-GMB-2022-0038

Supplementary material to "Comparative genomics and phylogenomics of the *Ralstonia solanacearum* Moko ecotype and its symptomatological variants"

Phylotype	Strain	CIP417	CFBP1416	Molk2	10314	CCRMRsB7	UA-1579	Po82	CCRMRs287	UA-1617	UA-1591	UA-1609	UW179	UW163	CCRMRs277	CCRMRs304	IBSBF1900	B50	Grenada91	UW181	UA-1612	UA-1611	SFC	IBSBF2570
Phylotype IIB-α	CIP417		99.80	99.70	99.70	86.40	78.20	77.40	77.60	77.60	77.60	78.30	77.80	77.30	67.70	67.70	67.20	67.20	68.20	68.10	68.60	68.30	67.80	68.10
	CFBP1416	99.98		99.60	99.50	86.10	78.10	77.10	77.30	77.30	77.20	78.00	77.50	77.00	67.40	67.40	67.00	66.90	68.00	67.90	68.40	68.10	67.50	67.80
	Molk2	99.96	99.95		99.50	85.80	77.80	77.00	77.20	77.20	77.20	77.80	77.40	76.90	67.30	67.20	66.90	66.80	67.70	67.70	68.10	67.80	67.40	67.60
	10314	99.94	99.94	99.92		85.80	77.90	77.10	77.30	77.30	77.20	77.90	77.40	77.00	67.40	67.30	66.90	66.80	67.70	67.70	68.10	67.90	67.40	67.60
	CCRMRsB7	98.55	98.55	98.51	98.51		77.50	76.70	76.70	76.70	76.70	77.50	76.80	76.60	68.40	68.40	68.00	67.90	68.30	68.10	68.50	68.20	68.10	68.50
Phylotype IIB-β	UA-1579	97.58	97.57	97.55	97.54	97.52		95.40	95.70	95.70	95.70	100.00	95.80	95.40	66.90	66.80	66.40	66.40	66.90	66.70	67.10	67.00	66.30	66.10
	Po82	97.53	97.52	97.49	97.49	97.45	99.53		99.40	98.50	97.70	95.30	99.90	99.20	67.10	67.10	66.80	66.80	66.30	66.30	66.80	66.70	66.30	66.70
	CCRMRs287	97.53	97.52	97.50	97.49	97.44	99.52	99.92		99.40	99.40	95.70	99.40	99.90	67.10	67.00	66.80	66.70	66.60	66.40	67.00	66.90	66.40	66.80
	UA-1617	97.54	97.53	97.50	97.50	97.45	99.54	100.00	99.93		99.90	95.60	99.90	99.20	67.10	67.10	66.90	66.80	66.50	66.40	67.00	66.90	66.40	66.80
	UA-1591	97.54	97.53	97.50	97.50	97.45	99.54	99.99	99.93	99.99		95.70	99.90	98.50	67.00	67.00	66.80	66.80	66.50	66.30	67.00	66.80	66.40	66.80
	UA-1609	97.58	97.57	97.54	97.54	97.52	99.99	99.52	99.52	99.53	99.54		95.70	95.40	66.90	66.90	66.40	66.40	66.90	66.70	67.10	67.00	66.30	66.10
	UW179	97.55	97.54	97.51	97.51	97.47	99.54	99.98	99.92	99.98	99.98	99.54		99.20	67.30	67.20	67.00	67.00	66.70	66.60	67.40	67.10	66.50	66.90
	UW163	97.53	97.51	97.48	97.48	97.43	99.52	99.92	99.99	99.92	99.92	99.51	99.92		66.90	66.80	66.50	66.50	66.40	66.40	66.70	66.70	66.30	66.70
Phylotype IIA-α	CCRMRs277	96.27	96.26	96.24	96.24	96.41	96.16	96.19	96.19	96.20	96.20	96.17	96.20	96.18		99.90	99.50	99.50	91.30	91.10	91.20	91.30	89.30	89.50
	CCRMRs304	96.27	96.25	96.23	96.23	96.41	96.16	96.18	96.18	96.18	96.19	96.16	96.19	96.17	99.99		99.50	99.40	91.20	91.00	91.00	91.20	89.20	89.50
	IBSBF1900	96.25	96.24	96.21	96.21	96.37	96.11	96.15	96.15	96.16	96.16	96.11	96.17	96.14	99.97	99.97		99.90	91.00	90.80	91.00	91.10	89.20	89.30
	B50	96.24	96.23	96.20	96.20	96.37	96.10	96.15	96.15	96.16	96.16	96.11	96.17	96.14	99.97	99.97	99.97		90.80	90.70	90.80	91.00	88.90	89.10
Phylotype IIA-β	Grenada91	96.31	96.31	96.29	96.27	96.38	96.12	96.11	96.11	96.12	96.12	96.12	96.14	96.12	99.04	99.03	99.02	99.01		99.70	99.70	99.70	91.70	91.70
	UW181	96.31	96.31	96.28	96.27	96.35	96.11	96.10	96.11	96.11	96.11	96.11	96.13	96.12	99.02	99.02	99.01	99.01	99.98		99.70	99.70	91.60	91.60
	UA-1612	96.33	96.33	96.29	96.29	96.37	96.14	96.13	96.13	96.14	96.14	96.14	96.17	96.13	99.03	99.03	99.02	99.01	99.98	99.98		99.80	91.90	91.90
	UA-1611	96.32	96.32	96.29	96.28	96.36	96.13	96.13	96.14	96.14	96.14	96.13	96.16	96.14	99.04	99.04	99.02	99.01	99.98	99.97	99.98		91.60	91.70
	SFC	96.30	96.29	96.26	96.27	96.32	96.12	96.11	96.13	96.13	96.12	96.12	96.15	96.14	98.89	98.88	98.90	98.86	99.10	99.08	99.11	99.09		99.90
	IBSBF2570	96.32	96.31	96.29	96.29	96.36	96.13	96.14	96.16	96.16	96.15	96.14	96.17	96.15	98.91	98.90	98.91	98.86	99.11	99.09	99.11	99.09	100.00	

Figure S1 - Heatmap generated from in silico DNA-DNA hybridization and average nucleotide identity using the MUMmer algorithm of *Ralstonia solanacearum* Moko ecotype genomes and its symptomatological variants. *The upper triangle refers to the values of *is*DDH and the lower triangle to the values of ANIm.
